# The First 5′-Phosphorylated 1,2,3-Triazolyl Nucleoside Analogues with Uracil and Quinazoline-2,4-Dione Moieties: A Synthesis and Antiviral Evaluation

**DOI:** 10.3390/molecules27196214

**Published:** 2022-09-21

**Authors:** Dmitry A. Tatarinov, Bulat F. Garifullin, Mayya G. Belenok, Olga V. Andreeva, Irina Yu Strobykina, Anna V. Shepelina, Vladimir V. Zarubaev, Alexander V. Slita, Alexandrina S. Volobueva, Liliya F. Saifina, Marina M. Shulaeva, Vyacheslav E. Semenov, Vladimir E. Kataev

**Affiliations:** 1 Arbuzov Institute of Organic and Physical Chemistry, FRC Kazan Scientific Center, Russian Academy of Sciences, Arbuzov Str., 8, 420088 Kazan, Russia; 2 Department of Organic and Medicine Chemistry, Kazan Federal University, Kremlevskaya 18, 420008 Kazan, Russia; 3 Pasteur Institute of Epidemiology and Microbiology, Mira Str., 14, 197101 Saint Petersburg, Russia

**Keywords:** nucleoside analogues, nucleotides, 1,2,3-triazole, antivirals, influenza virus, click chemistry

## Abstract

A series of 5′-phosphorylated (dialkyl phosphates, diaryl phosphates, phosphoramidates, *H*-phosphonates, phosphates) 1,2,3-triazolyl nucleoside analogues in which the 1,2,3-triazole-4-yl-β-D-ribofuranose fragment is attached via a methylene group or a butylene chain to the *N*-1 atom of the heterocycle moiety (uracil or quinazoline-2,4-dione) was synthesized. All compounds were evaluated for antiviral activity against influenza virus A/PR/8/34/(H1N1). Antiviral assays revealed three compounds, **13b**, **14b,** and **17a**, which showed moderate activity against influenza virus A (H1N1) with IC_50_ values of 17.9 μM, 51 μM, and 25 μM, respectively. In the first two compounds, the quinazoline-2,4-dione moiety is attached via a methylene or a butylene linker, respectively, to the 1,2,3-triazole-4-yl-β-D-ribofuranosyl fragment possessing a 5′-diphenyl phosphate substituent. In compound **17a**, the uracil moiety is attached via the methylene unit to the 1,2,3-triazole-4-yl-β-D-ribofuranosyl fragment possessing a 5′-(phenyl methoxy-L-alaninyl)phosphate substituent. The remaining compounds appeared to be inactive against influenza virus A/PR/8/34/(H1N1). The results of molecular docking simulations indirectly confirmed the literature data that the inhibition of viral replication is carried out not by nucleoside analogues themselves, but by their 5′-triphosphate derivatives.

## 1. Introduction

**Figure 1:**
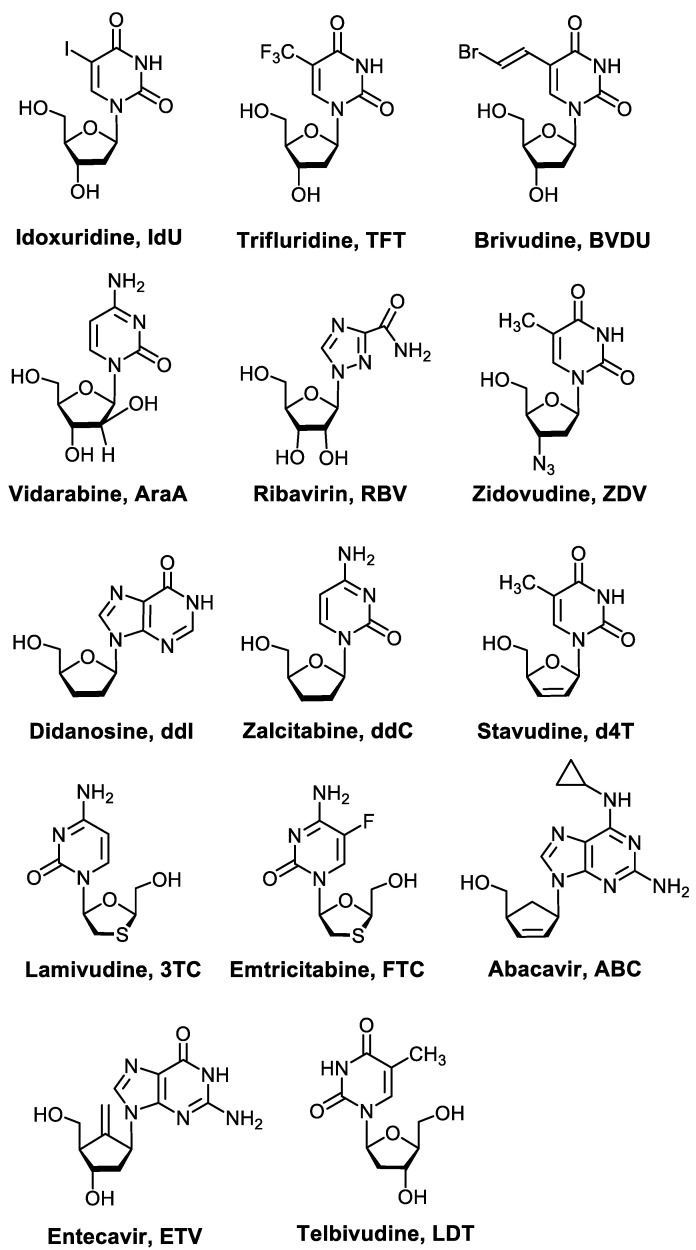
The most well-known antiviral cyclic nucleoside analogues.

The seven decades that have passed since the discovery of the structure of DNA have shown that analogues of natural nucleosides are the leading line of drugs used to fight infections caused by HIV, hepatitis B and C viruses, Dengue virus, Ebola virus, herpes virus, and others [1,2,3]. Moreover, it was representatives of nucleoside analogues that proved effective in COVID-19 chemotherapy [4]. The most well-known cyclic nucleoside analogues used in the treatment of various viral infections are shown in Figure 1. Chronologically, the first nucleoside analogue whose antiviral activity was detected was Idoxuridine (IdU), which was approved by the U.S. Food and Drug Administration (FDA) in 1963 for the treatment of herpes simplex virus (HSV) and varicella-zoster virus (VZV) [1] (Figure 1). Trifluoruridine (TFT) was also licensed for the treatment of herpes in 1980 [1]. Brivudine (BVDU) was approved by the FDA in 1980 against VZV [5]. Six years later, Vidarabine (AraA) was licensed for the treatment of HSV and VZV [1]. Ribavirin (RBV) is the first nucleoside analogue that has shown activity against several RNA viruses (hepatitis C, influenza A viruses, respiratory syncytial virus, etc.) [6]. In addition, Ribavirin is the only nucleoside analogue among all drugs approved by the FDA for the treatment of influenza [6]. Zidovudine (ZDV) (Figure 1) was not only the first drug approved for the treatment of HIV infection, but also the first nucleoside analogue for which a target was established—HIV reverse transcriptase (RNA-dependent DNA polymerase) [6]. Accordingly, Zidovudine was the first to be named a nucleoside reverse transcriptase inhibitor (NRTI). Didanosine (ddI) (Figure 1), synthesized in 1964 and approved by the FDA in 1991, appeared to be the second NRTI after Zidovudine for HIV treatment [2,6]. Zalcitabine (ddC) (Figure 1), synthesized in the late 1960s and released to the pharmaceutical market in 1992, became the third nucleoside analogue approved by the FDA for the treatment of HIV infection [2,6]. Stavudine (d4T) (Figure 1) was synthesized in the late 1960s, but its ability to inhibit HIV replication was discovered only in 1982 during the AIDS epidemic that began in New York City. Approved for use in 1994, Stavudine became the fourth drug among nucleoside analogues for the treatment of HIV infection [2,6]. Completing the list of NRTIs, Lamivudine (3TS), Abacavir (ABC), and Emtricitabine (FTC) were approved by the FDA for the treatment of HIV infection in 1996, 1998, and 2004, respectively [1,2,6] (Figure 1). Entecavir (ETV) and Telbivudine (LDT) (Figure 1) both were approved for the treatment of HBV infection in 2005 and 2006, respectively [3].

From 1986 to the 1990s, a series of studies showed that Zidovudine (ZDV) and Stavudine (d4T) in uninfected and HIV-infected cells were metabolized under the action of various cellular kinases (phosphotransferases) to their 5′-mono-, 5′-di-, and 5′-triphosphate derivatives [7]. It was later assumed that all nucleoside analogues are subjected to such metabolism and 5′-triphosphorylated derivatives formed in this way are their active forms. These 5′-triphosphate forms compete with natural nucleotides for binding to the active centers of viral RNA-dependent RNA polymerases (RdRp) and inhibit the synthesis of viral nucleic acids, which leads to blocking the replication of a virus [1,8,9].

Numerous studies have revealed the following features of the intracellular metabolism of nucleoside analogues affecting their antiviral activity. (i) Since nucleoside analogues (NA) are structurally different from natural nucleosides, their phosphorylation by nucleoside/nucleotide cellular kinases to generate the active NA-5′-triphosphate metabolites is often of limited efficiency [10,11,12]. Thus, for example, phosphorylation of Zidovudine monophosphate (ZDV-MP) to Zidovudine diphosphate (ZDV-DP) is very inefficient in lymphocytes and peripheral blood mononuclear (PBM) cells, and ZDV accumulates as a monophosphate representing approximately 95% of all its phosphorylated forms [10,12]. Consistent with the rate-limited formation of Stavudine monophosphate (d4T-MP) caused by poor recognition of d4T by cellular kinases, the majority of intracellular d4T remains in its unphosphorylated form [12]. Since Zidovudine and Stavudine are ineffectively phosphorylated by cellular kinases in resting PBM cells in vitro, they exhibit weak anti-HIV activity in them. In contrast, ddI, ddC, and 3TC (Figure 1) are phosphorylated quite efficiently in these types of cells in vitro and exhibit more potent anti-HIV activity [11]. (ii) The intracellular concentration of nucleoside analogue 5′-triphosphates is regulated by positive or negative feedback mechanisms on one or more enzymes in the phosphorylation pathway. Regulation of the pathway can be very complex and can be affected by other nucleosides (endogenous as well as exogenous) in multiple negative and positive feedback regulatory loops [12]. 

To overcome the problems of intracellular phosphorylation of nucleoside analogues caused by their poor recognition by cellular kinases (phosphotransferases), which leads to insignificant concentrations of active 5′-triphosphate forms and, consequently, to low antiviral activity, chemists focused on the synthesis of 5′-phosphorylated derivatives of nucleoside analogues. It was assumed that the introduction of synthesized 5′-mono-, 5′-di-, or 5′-triphosphate nucleoside analogues into an infected cell would avoid three, two, or even one stage of intracellular phosphorylation and deliver to a viral target already prepared active forms. The published various synthetic approaches for preparing 5′-mono-, 5′-di-, and 5′-triphosphate nucleoside analogues have been systematized and outlined in several reviews and books (see, for example, [13,14]). 

Note that in parallel, in principle different directions of synthesis of antiviral nucleoside analogues began to develop. The thing is, the data accumulated in the 1950s that the nucleotides of naturally-occurring pyrimidines and purines are poorly incorporated into cell nucleic acids caused serious doubts about their ability to penetrate cells through lipid-rich cell membranes due to negative charges in phosphate groups at physiological pH. A. Montgomery was the first to suggest in 1961 that this difficulty could be overcome if it were possible to prepare a nucleotide ester that could penetrate through the cell wall and then be metabolized to the nucleotide itself [15]. This idea was soon developed, and it was proposed to use so-called prodrugs representing 5′-monophosphates of nucleoside analogues in which the monophosphate group is masked by any easily hydrolysable groups, thereby making the molecule more lipophilic and hence improving its transport into cells. Upon cell entry, the masking groups are enzymatically cleaved off to release free 5′-monophosphate, which is further transformed by cellular kinases to the active 5′-triphosphate form of the nucleoside analogue [16]. 

**Figure 2:**
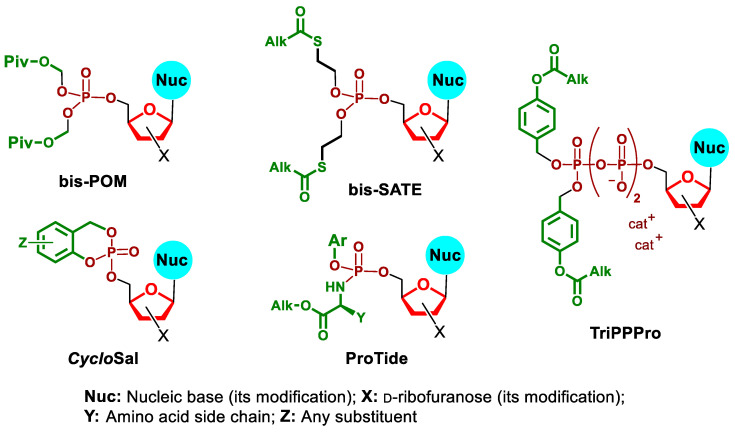
Schematic representation of the most known prodrugs.

Several prodrug approaches have been developed to date. The best known among them are the bis-POM, bis-SATE, *Cyclo*Sal, ProTide, and TriPPPro approaches. Chronologically, the first was the bis-POM approach, which uses bis-***p***ivaloyl***o***xy***m***ethyl (POM)-masking groups and was reported in 1983 by D. Farquhar [17] (Figure 2). This approach utilizes a carboxyesterase-catalyzed cleavage of the pivaloyl ester within the POM-masking group to yield the highly-reactive hydroxymethyl phosphotriester, which is converted into a mono(POM) phosphodiester, and, in turn, is hydrolyzed by a phosphodiesterase that affords the monophosphate of a parent nucleoside analogue [17]. Unfortunately, this approach cannot be considered successful because the delivery of one molecule of a parent nucleoside analogue in the form of a bis-POM prodrug results in the liberation of two equivalents of potentially toxic formaldehyde and pivalic acid. The following prodrug approach was reported by J. L. Imbach, who suggested to use bis(***S***-***a***cyl-2-***t***hio***e***thyl (bis-SATE) nucleotides as prodrugs [18] (Figure 2). The bis-SATE approach is also not optimal, since as a result of the cleavage of bis-SATE-masking groups, two equivalents of episulfide are chronically and acutely toxic in mice and rats [19], and are released besides the monophosphate of a parent nucleoside analogue [18]. 

In 1996, C. Meier designed a new and entirely different prodrug approach, named the *Cyclo*Sal approach, which uses salicyl alcohol as a cyclic bifunctional masking unit. Salicyl alcohol is attached to a phosphorus atom via a phenyl- and a benzyl ester bond, while a parent nucleoside analogue is attached through an alkyl ester bond (Figure 2) [20]. The introduction of these three different ester bonds allows sufficient discrimination in the process of enzymatic hydrolysis occurring by tandem (or cascade) mechanism. First, the phenolate is preferentially displaced at the phosphate group, and then spontaneous hydrolysis of the benzyl ester bond occurs, affording the 5′-monophosphate of the parent nucleoside analogue and salicylic alcohol, which is non-toxic [20]. The literature is full of examples of successful use of the *Cyclo*Sal approach. Thus, the functionalization of 2′,3′-dideoxyadenosine with the 5′-cyclosaligenyl moiety increased its anti-HIV activity by 100 times [21,22]. The 5′-*Cyclo*Sal derivative (prodrug) of 2′,3′-didehydro-2′,3′-dideoxyadenosine exceeded the anti-HIV activity of the parent nucleoside 400-fold [22]. The effectiveness of Abacavir (Figure 1) after its transformation into a 5′-cyclosaligenyl derivative improved 4 times [23], whereas the functionalization of Carbovir (6′-deoxy-2′,3′-didehydro-2′,3′-dideoxyguanosine) with the 5′-cyclosaligenyl moiety increased its anti-HIV activity by 30 times [23]. The functionalization of Brivudine (Figure 1) with a 5′-cyclosaligenyl moiety led to the appearance of its prodrug form of pronounced activity against the Herpes virus [24]. At the same time, the antiviral activity of Stavudine (Figure 1) and its 5′-cyclosaligenyl derivative appeared to be almost the same [25]. 

In the early 1990s, C. McGuigan proposed his prodrug approach, which he later called the ProTide approach [26]. In this approach, the 5′-phosphate substituent of nucleoside (nucleotide) analogues is masked by an aryl group and an amino acid ester residue. Such a phosphoramidate, which became known as ProTide (PROdrug + nucleoTIDE) [27] (Figure 2), is able to enter the cell via facilitated passive diffusion or by means of nucleoside transporters and afford a monophosphate form of a parent nucleoside analogue after cleavage of the masking groups via sequence enzyme-mediated and spontaneous steps, including the hydrolysis of the amino acid ester by carboxyesterase or cathepsin A, displacement of the phosphate phenol by the carboxylate anion, chemical hydrolysis, and cleavage of the amino acid moiety by the histidine triad nucleotide-binding protein [28]. Numerous studies have shown a clear correlation between the nature of aromatic and amino acid moieties and antiviral potency. ProTides with electron acceptor groups in the para-position of the aromatic moiety were the most active, while phosphoramidate with electron-donating groups showed the worst activity. ProTides possessing a phenyl moiety occupied an intermediate position [27]. As for the nature of the amino acid ester residue, alanine phosphoramidates proved to be strikingly more effective than other amino acids, for example, compared to leucine by 10 times and glycine by more than 100 times [27,29]. The literature provides many examples of the effectiveness of using the ProTide approach. For example, the 5′-(phenyl methoxy-L-alaninyl)phosphate of Zidovudine (ProTide-ZDV, Figure 2) showed more than 100-times better activity against HIV than the parent nucleoside analogue, Zidovudine (Figure 1) [27]. The ProTides of Stavudine and Abacavir (ProTide-d4T and ProTide-ABC, Figure 2) were more than 70-times and 20-times more active against HIV than their parent nucleoside analogues Stavudine and Abacavir, respectively (Figure 1) [27]. For 30 years, the ProTide approach has been applied to a wide range of nucleoside analogues. Dozens of phosphoramidate prodrugs have been synthesized, which have shown powerful antiviral activity against various RNA viruses [30,31,32]. These studies have paved the way for the discovery of such bright ProTides as Sofosbuvir (phosphoramidate of 2′-deoxy-2′-α-fluoro-2′-β-methyluridine) and Remdesivir (phosphoramidate 1′-cyano-7,9-dideaza-4-azaadenosine). Sofosbuvir was approved by the FDA in December 2013 for the treatment of HCV [33]. Remdesivir, which exhibits a wide range of activities against RNA viruses [4,28], is currently the only FDA-approved antiviral drug for the treatment of severe acute respiratory syndrome coronavirus 2 (SARS-CoV-2) infection [34]. 

The prodrugs mentioned above intracellularly release monophosphate forms that still need to undergo two additional phosphorylation steps into their corresponding triphosphates’ active metabolites. In 2015, C. Meier discovered the unique method of delivering bioactive triphosphate of nucleoside analogues in the cell, which is known as the TriPPPro approach [35]. This approach uses the functionalization of 5ʹ-triphosphate forms of nucleoside analogues by two masking alkoxycarbonyloxybenzyl groups (Figure 2). Although TriPPPro compounds are still charged at the phosphate groups, obviously the modification at the γ-phosphate groups by lipophilic bioreversible moieties gives the molecule sufficient lipophilicity to penetrate the cell membrane. Thus, unlike previous approaches, by using the TriPPPro approach, nucleoside analogues do not need any intracellular phosphorylation. The literature provides examples of the effectiveness of the TriPPPro approach. Both TriPPPro derivatives of Stavudine (TriPPPro-d4T, Figure 2) and TriPPPro derivatives of Abacavir (TriPPPro-ABC, Figure 2) are superior to their parent nucleoside analogues Stavudine (d4T, Figure 1) and Abacavir (ABC, Figure 1) in antiviral activity against HIV-1 and HIV-2—the first by 10 times [36] and the second by 3–6 times [37,38]. However, the antiviral activity of Zalcitabine (ddC, Figure 1) and its TriPPPro derivative against HIV-1 and HIV-2 are the same [37]. 

Surprisingly, despite many publications on the synthesis of 5′-phosphorylated derivatives of nucleoside analogues [13,14], we found in the literature just a few articles on the study of their antiviral activity. Firstly, in 1989, A. Kraevsky’s group synthesized a 5′-*H*-phosphonate derivative of Zidovudine, which demonstrated high anti-HIV activity [39]. In 1999, this prodrug form of Zidovudine was registered in the Russian Federation as an antiviral agent for combined antiretroviral therapy against HIV infection called Nicavir [39,40]. Secondly, in 2007, D. Liotta’s group reported that the 5′-triphosphate of 5-fluoro-1-[*cis*-3-(hydroxymethyl)-cyclobutyl]-cytosine showed in vitro anti-HIV activity against recombinant HIV reverse transcriptases (RT) and wild-type HIV RT (IC_50_ = 4.7 and 6.9 μM, respectively), whereas the parent nucleoside was inactive up to 100 μM. The same high activity was demonstrated by the 5′-triphosphate of Lamivudine (3TC, Figure 1) [41]. Third, four synthesized phosphoramidate prodrugs of β-D-2′-deoxy-2′-fluoro-2′-C-methyl-7-deazapurine nucleoside analogues showed no anti-HCV activity, whereas appropriate 5′-triphosphates of parent nucleosides demonstrated potent inhibitory effects against individual wild-type and S282T mutant HCV polymerases. Cellular pharmacology studies in Huh-7 cells revealed that 5′-triphosphates were not formed at significant levels from these phosphoramidate prodrugs, indicating that insufficient cellular phosphorylation was responsible for the lack of their anti-HCV activity [42]. Fourth, it was found that while 2′,3′-dideoxy-β-D-apio-D-furanonucleosides failed to show anti-HIV activity, their 5′-triphosphate prodrugs were readily accepted by a viral DNA polymerase to act as a DNA chain terminator [43]. Fifth, the ability of the 5′-triphosphates of four HIV RT inhibitors, Zidovudine, Lamivudine, Emtricitabine, and Abacavir (Figure 1), to be incorporated by the RNA-dependent RNA polymerase (RdRp) of SARS-CoV, wherein they also terminated further polymerase extension—thereby inhibiting virus replication—was demonstrated [44]. Of all the five cases listed above, only the groups of A. Kraevsky [39,40] and D. Liotta [41] incubated triphosphates of nucleoside analogues in infected cells (in other cases, experiments were conducted with individual viral RdRp) and found their antiviral activity. In our opinion, this is enough to cast doubt on the statement made in the 1950s that negatively-charged 5′-triphosphate nucleoside analogues cannot penetrate into cells through lipid-rich cell membranes [15]. Indeed, during 2000–2010, integral proteins were identified in human cells, of which not only nucleoside analogues, but also a variety of organic anions, penetrate cells [9,45]. The above leads to the idea of the expediency of continuing the synthesis and comparison of the antiviral activity of the three groups of compounds: nucleoside analogues, their 5′-phosphorylated derivatives, and their various prodrug forms. Moreover, it is interesting to consider not only the nucleoside analogues already used in clinical settings, but also other antiviral nucleoside analogues with an unusual structure.

Recently, we synthesized a series of 1,2,3-triazolyl nucleoside analogues in which a nucleic base (uracil or thymine) or its derivative (6-methyluracil or quinazoline-2,4-dione) was connected to the D-ribofuranose residue via a 1,2,3-triazolyl bridge and an alkyl (methylene, propylene or butylene) linker [46,47]. The evaluation of antiviral potency revealed compounds that showed moderate (IC_50_ = 30 and 42 μM) in vitro activity against influenza A (H1N1) virus [47]. Considering the literature data presented above, it was of interest to compare the antiviral activity of these 1,2,3-triazolyl nucleoside analogues [47] with the antiviral activity of their 5′-phosphorylated derivatives, which both possess a negatively-charged phosphate or *H*-phosphonate group and neutral ether or phosphoramidate group. In our opinion, this would be the first comparison of the antiviral activity of a nucleoside analogue, its negatively-charged 5′-monophosphate and 5′-*H*-phosphonate derivative, and its 5′-phosphorylated derivatives with a masked phosphate group (i.e., prodrug forms), which would allow, at least indirectly, the assessment of whether a synthetic nucleotide (i.e., prodrug forms) penetrates into the cell or not. Herein, we report on the synthesis and antiviral evaluation against influenza A (H1N1) virus of a series of 5′-phosphorylated (dialkyl phosphates, diaryl phosphates, phosphoramidates, *H*-phosphonates, phosphates) derivatives of 1,2,3-triazolyl nucleoside analogues in which the uracil or quinazoline-2,4-dione moiety is attached to the D-ribofuranose residue by means of a 1,2,3-triazolyl bridge and the methylene or butylene linker.

## 2. Results and Discussion

### 2.1. Chemistry

#### 2.1.1. Synthesis of 1,2,3-Triazolyl Nucleoside Analogues

**Scheme 1:**
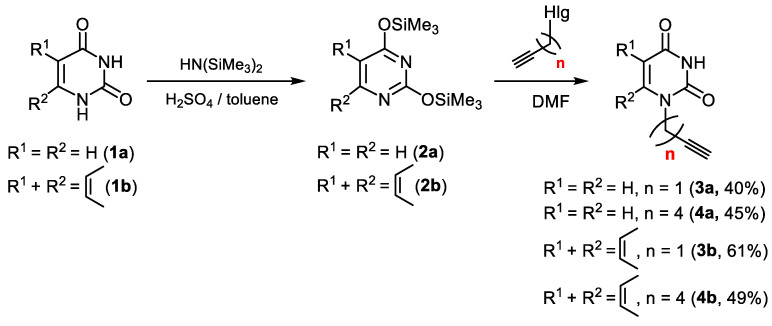
Synthesis of pyrimidine derivatives containing a ω-alkyne substituent at the *N-*1 atom.

The synthesis of starting 1,2,3-triazolyl nucleoside analogues was carried out in three stages in accordance with the described methods [46,47]. During the first stage (Scheme 1), uracil **1a** and quinazoline-2,4-dione **1b** were converted into 2,4-bistrimethylsilyl esters **2a** and **2b** by reflux with an excess of hexamethyldisilazane in toluene in the presence of H_2_SO_4_. Then, these esters were selectively alkylated in situ with propargyl bromide and 6-iodo-1-hexine in DMF to afford 1-alkyne derivatives of uracil **3a** and **4a** and quinazoline-2,4-dione **3b** and **4b** at 40–61% yields.

**Scheme 2:**
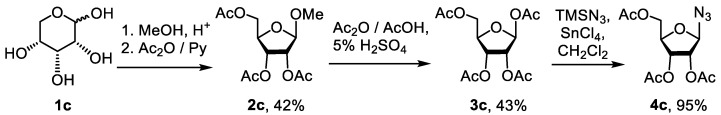
Synthesis of 2,3,5-tri-*O*-acetyl-β-D-ribofuranosyl azide **4c**.

During the second stage (Scheme 2), commercial D-ribose **1c** was converted by interaction with MeOH into methyl-D-α/β-ribofuranoside, which was acylated in situ to obtain methyl-2,3,5-tri-*O*-acetyl-β-D-ribofuranoside **2c**. 

Then, by reaction of monosaccharide **2c** with a mixture of glacial AcOH and Ac_2_O in the presence of H_2_SO_4_, tetra-acetylated β-D-ribofuranose **3c** was obtained, the interaction of which with trimethylsilylazide (TMSN_3_) in the presence of tin tetrachloride led to 2,3,5-tri-*O*-acetyl-β-D-ribofuranosylazide **4c** at a 95% yield (Scheme 2).

**Scheme 3:**
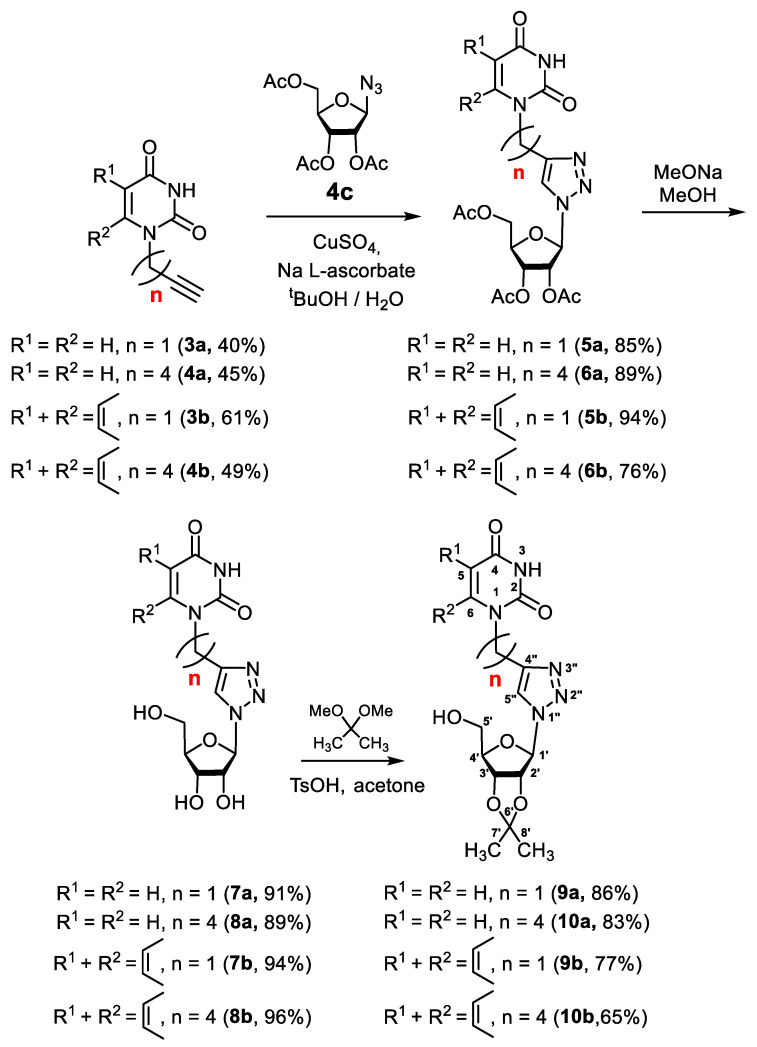
Synthesis of 1,2,3-triazolyl nucleoside analogues.

During the third stage (Scheme 3), 1-alkyne derivatives of uracil **3a** and **4a** and quinazoline-2,4-dione **3b** and **4b** were involved in the copper sulfate-catalyzed reaction of azide-alkyne cycloaddition (CuAAC) with 2,3,5-tri-*O*-acetyl-β-D-ribofuranosylazide **4c**. 

The reaction was carried out in a mixture of t-BuOH/H_2_O (1:1), using equimolecular amounts of azide and alkyne components, CuSO_4_·5H_2_O (10 mol%), and sodium ascorbate (20 mol%) according to the procedure previously described [46]. The 1,2,3-triazolyl nucleoside analogues with acylated hydroxyl groups **5a**, **6a**, **5b**, and **6b** were obtained at 76–89% yields (Scheme 3). The formation of a 1,2,3-triazole ring was confirmed by the appearance of a signal of the triazolyl proton, C-5′′-H, within the range of 7.50–7.97 ppm in the ^1^H NMR spectra of compounds **5a**, **6a**, **5b**, and **6b**. The triazolyl carbons, C-4″, in the ^13^C NMR spectra resonated within the range of 142.85–147.68 ppm, and signals of the triazolyl carbons, C-5″, were observed within the range of 120.10–123.54 ppm. All these facts fully corresponded to the characteristic features of 1,2,3-triazoles in ^1^H and ^13^C NMR spectra described in the literature [48,49,50,51]. Compounds **5a**, **6a**, **5b**, and **6b** were obtained as β-anomers. This was evidenced by the resonance of the anomeric protons of acetylated D-ribofuranose residues as doublets within the range of 5.98–6.13 ppm with vicinal coupling constants within the range of 3.5–4.2 Hz, which corresponded to the literature data [52].

For further phosphorylation of 1,2,3-triazolyl nucleoside analogues by very reactive phosphorus-containing reagents—for example, *H*-phosphonates—it was necessary to remove the protection of the hydroxyl group at the C-5′ atom, leaving the protection of the hydroxyl groups at the C-2′ and C-3′ atoms. For this purpose, first, the acetyl protection of the sugar residue of compounds **5a**, **6a**, **5b**, and **6b** was removed with a solution of sodium methylate in methanol, and then compounds **7a**, **8a**, **7b**, and **8b** were obtained at 89–91% yields and were converted to the 1,2,3-triazolyl nucleoside analogues **9a**, **10a**, **9b**, and **10b** with isopropylidene protection of hydroxyl groups at the C-2′ and C-3′ atoms and a free hydroxyl group at the C-5′ atom by interaction with 2,2-dimethoxypropane in acetone in the presence of para-toluene sulfonic acid (Scheme 3).

#### 2.1.2. Synthesis of 5′-Phosphorylated Derivatives of 1,2,3-Triazolyl Nucleoside Analogues

**Scheme 4:**
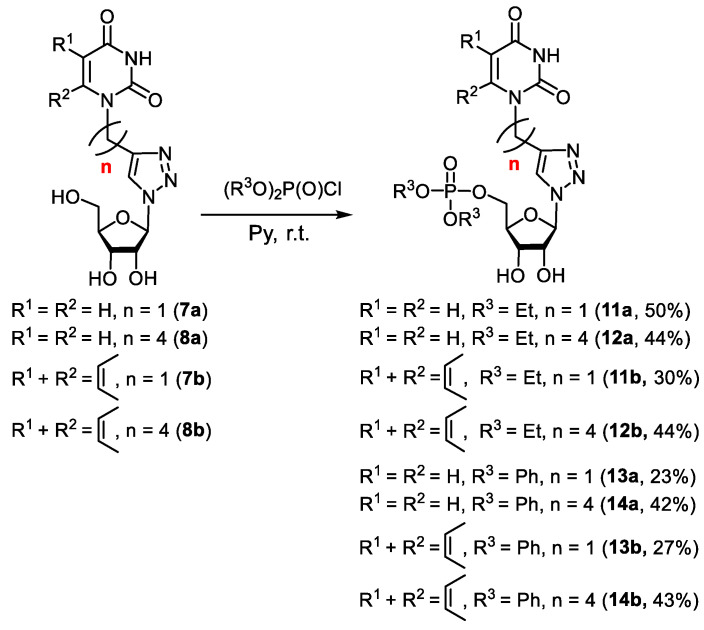
Synthesis of 5′-diethyl- and 5′-diphenylphosphates of 1,2,3-triazolyl nucleoside analogues.

The synthesis of 1,2,3-triazolyl nucleotide analogues with 5′-dialkyl- and 5′-diphenyl phosphate moieties at the C-5′ atom was carried out by analogy with the known method of phosphorylation of nucleoside analogues with D-arabinofuranose residue [53]. Compounds **7a**, **8a**, **7b**, and **8b** with unprotected hydroxyl groups were involved in a reaction with diethyl phosphorochloridate or diphenyl phosphorochloridate in pyridine at room temperature to afford 5′-diethyl phosphates **11a**, **12a**, **11b**, and **12b,** and 5′-diphenyl phosphates **13a**, **14a**, **13b**, and **14b** (Scheme 4). These compounds were isolated by flash chromatography on silica gel at 16–42% yields, respectively.

The appearance of a signal at −1.5 ppm in the ^31^P NMR spectra of compounds **11a**,**b** and **12a**,**b** and a signal of −12.1 ppm in the ^31^P NMR spectra of compounds **13a**,**b** and **14a**,**b** confirmed the addition of a diethyl phosphate or diphenyl phosphate group to the C-5′ atom of the initial nucleosides **7a**,**b** and **8a**,**b**. In general, a multiplicity, chemical shifts, and an integral intensity of signals in the ^1^H NMR spectra of synthesized diethyl phosphates **11a**,**b** and **12a**,**b** as well as diphenyl phosphates **13a**,**b** and **14a**,**b** are consistent with the structures shown in Scheme 4. 

**Scheme 5:**
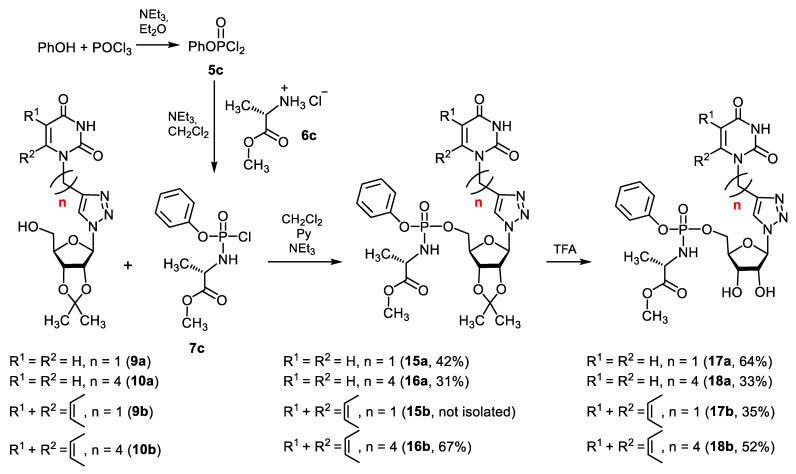
Synthesis of 5′-phosphoramidates of 1,2,3-triazolyl nucleoside analogues.

The synthesis of 1,2,3-triazolyl nucleoside analogues with a 5′-phosphoramidate moiety was carried out in three stages (Scheme 5) by analogy with the procedure developed in 1993 for the synthesis of a prodrug form of the antiretroviral drug Zidovudine [26]. According to the literature data [27,29], a phenyl group was selected as the aromatic moiety of the phosphoramidate fragment and an alanine residue was selected as the amino acid moiety of the phosphoramidate fragment. At the first stage, phenyl phosphorodichloridate **5c** was prepared by the reaction of phenol with phosphorus oxychloride. During the second stage, condensation of phenyl phosphorodichloridate **5c** with L-alanine methyl ether hydrochloride **6c** in the presence of triethylamine at −78 °C gave phenyl methoxyalaninyl phosphorochloridate **7c** a yield of more than 97%. During the third stage, amidophosphorochloridate **7c** was involved in a reaction with 2′,3′-*O*-isopropylidene-protected nucleosides **9a**,**b** and **10a**,**b** in dichloromethane. A pyridine mixture in the presence of triethylamine to afford 5′-phosphoramidates **15a**,**b** and **16a**,**b** were isolated by flash chromatography as a mixture of two diastereomers at 31–67% yields. Then, their 2,3-*O*-isopropylidene protection was removed with a 50% aqueous solution of trifluoroacetic acid (TFA) to obtain the target phosphoramidates **17a**,**b** and **18a**,**b** at 33–64% yields (Scheme 5).

**Scheme 6:**
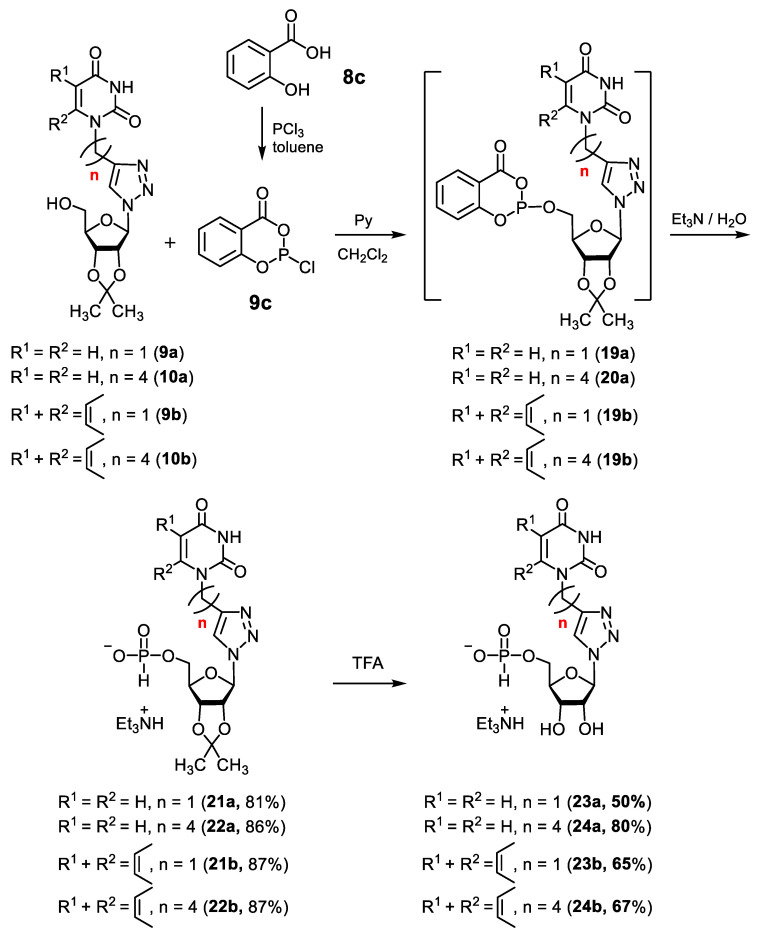
Synthesis of 5′-*H*-phosphonates of 1,2,3-triazolyl nucleoside analogues.

The synthesis of 1,2,3-triazolyl nucleoside analogues with a 5′-*H*-phosphonate moiety was performed similarly to the known procedure [54] by the interaction of 2′,3′-*O*-isopropylidene-protected nucleosides **9a**,**b** and **10a**,**b** with salicyl phosphorochloridate (2-chloro-1,3,2-benzodioxaphosphorin-4-one) **8c,** which was prepared by the reaction of salicylic acid with phosphorus trichloride as previously described [55] (Scheme 6). The completeness of the reaction was monitored by ^31^P NMR spectroscopy. The resulting salicyl phosphites **19a**,**b** and **20a**,**b** were manifested in the ^31^P{^1^H} NMR spectra in the form of two singlets near 125 ppm due to the formation of diastereomers differing in the configuration of the asymmetric phosphorus atom. In the ^31^P NMR spectra of **19a**,**b** and **20a**,**b,** these signals transformed to triplets with spin-coupling constants ^3^*J*_PH_ = 9.0 Hz. The resulting nucleoside 5′-salicyl phosphites **19a**,**b** and **20a**,**b** were hydrolyzed in situ to afford 2′,3′-*O*-isopropylidene-protected nucleoside 5′-*H*-phosphonate monoesters **21a**,**b** and **22a**,**b,** which were isolated by flash chromatography on silica gel at 31–64% yields (Scheme 6). The formation of the *H*-phosphonate group was confirmed by the ^31^P and ^31^P{^1^H} NMR spectra. The asymmetry of the phosphorus atom during the formation of the *H*-phosphonate anion disappeared, therefore the ^31^P {^1^H} NMR spectra of **21a**,**b** and **22a**,**b** showed a single singlet at 10 ppm and the ^31^P NMR spectra of **21a**,**b** and **22a**,**b** exhibited a doublet of triplets within the range of ^1^*J*_PH_ = 617–622 Hz. Treatment of 2′,3′-*O*-isopropylidene-protected nucleoside *H*-phosphonate monoesters **21a**,**b** and **22a**,**b** with aqueous TFA gave the desired unprotected nucleoside 5′-*H*-phosphonate monoesters **23a**,**b** and **24a**,**b** in 50–80% yields.

**Scheme 7:**
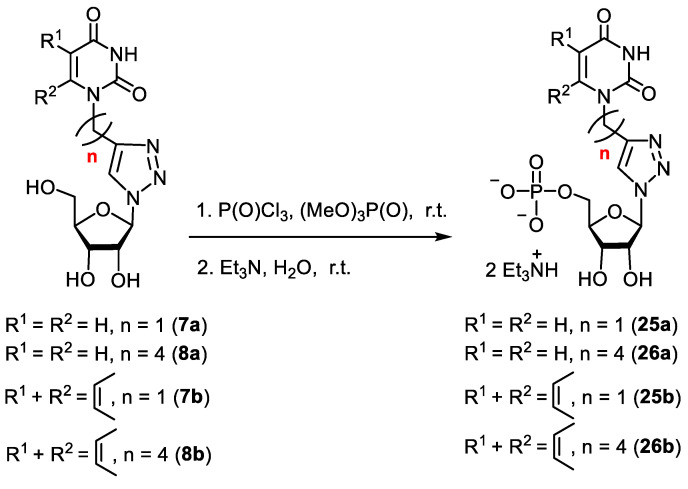

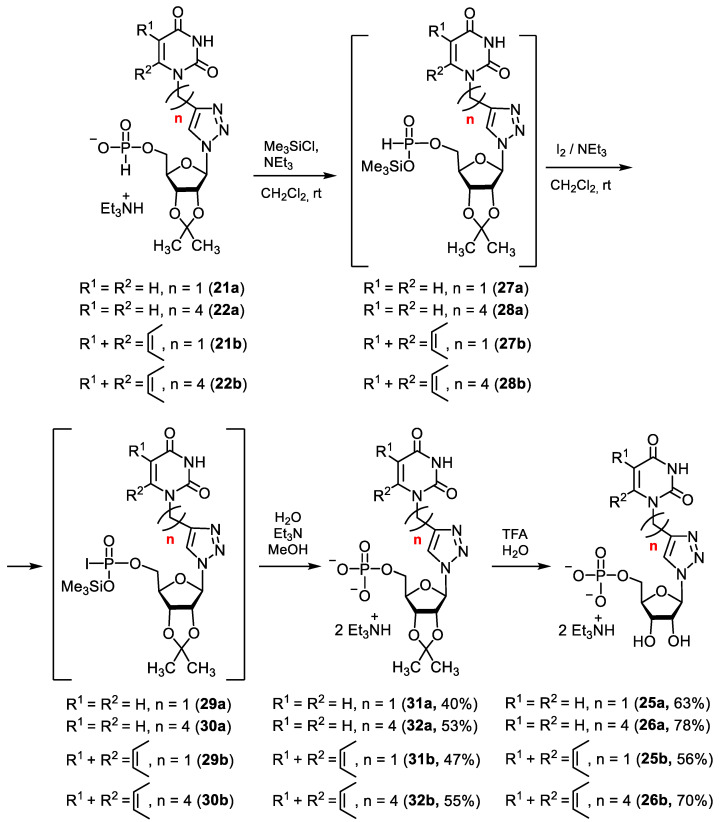
Synthesis of 5′- phosphates of 1,2,3-triazolyl nucleoside analogues.

The synthesis of 1,2,3-triazolyl nucleoside analogues with a 5′-phosphate moiety was initially carried out by analogy with the known methodology [56,57] by the interaction of nucleoside analogues **7a**,**b** and **8a**,**b** with phosphorus oxychloride in trimethyl phosphate, which was used as a solvent, followed by hydrolysis (Scheme 7). This methodology makes it possible to use nucleosides with unprotected hydroxyl groups of the ribofuranose residue as starting compounds, that is, it is characterized by the ease of implementation and, in addition, high conversion [56,57]. Indeed, according to TLC data, the interaction of 1,2,3-triazolyl nucleoside analogues **7a**,**b** and **8a**,**b** with phosphorus oxychloride proceeded with almost complete conversion of the initial nucleosides (>95%). The formation of target products **25a**,**b** and **26a**,**b** was confirmed by the ^31^P{^1^H} NMR spectra of the reaction mixtures, in which a singlet was present within the range of −0.5–3 ppm. Moreover, the ^31^P NMR spectra of the reaction mixtures exhibited a phosphorus atom of the C^5^^′^H_2_OP(O)O_2_^2−^ fragment as a triplet with a spin-coupling constant ^3^*J*_PH_ = 6.7 Hz.

Unfortunately, the isolation of 5′-phosphates **25a**,**b** and **26a**,**b** by chromatography methods turned out to be difficult due to the impurity of trimethylammonium salts of phosphoric acid, which could not be separated either by flash chromatography with gradient elution or chromatography on ion exchange resins. Having failed in the isolation of individual 5′-phosphates **25a**,**b** and **26a**,**b** synthesized by the known methodology [56,57], we used another approach based on the oxidation of 5′-trimethylsilyl-*H*-phosphonate monoesters with elemental iodine [58]. The 2′,3-*O*-isopropylidene-protected nucleoside *H*-phosphonate monoesters **21a**,**b** and **22a**,**b** were first converted into the trimethylsilyl *H*-phosphonate diesters **27a**,**b** and **28a**,**b** (using 3 mol. equiv. of TMSCl in CH_2_Cl_2_), which were oxidized in situ by elemental iodine to afford the halogenophosphates **29a**,**b** and **30a**,**b**—which were hydrolyzed in situ with aqueous triethylamine to give 2,3-*O*-isopropylidene-protected nucleoside 5′-phosphates **31a**,**b** and **32a**,**b,** which were isolated by flash chromatography on silica gel in 40–55% yields (Scheme 7). The formation of compounds **31a**,**b** and **32a**,**b** was confirmed by the appearance of a single singlet at –0.5 ppm in their ^31^P{1H} NMR spectra, which underwent a significant broadening in their ^31^P NMR spectra. The absence of the ^1^*J*_PH_ constant indicated complete conversion of the initial nucleoside *H*-phosphonate monoesters **21a**,**b** and **22a**,**b**. Then, the isopropylidene protection of 1,2,3-triazolyl nucleotide analogues **31a**,**b** and **32a**,**b** was removed with an aqueous solution of trifluoroacetic acid (TFA) to obtain target 5′-phosphates of 1,2,3-triazolyl nucleoside analogues **25a**,**b** and **26a**,**b** in 56–78% yields (Scheme 7).

### 2.2. Antiviral Evaluation

The in vitro antiviral activity of the synthesized compounds **11a**,**b**–**14a**,**b**; **17a**,**b**; **18a**,**b**; and **23a**,**b**–**26a**,**b** was evaluated regarding the A/Puerto Rico/8/34 (H1N1) strain of influenza virus. The resulting data expressed as virus-inhibiting activity (IC_50_), a medium cytotoxic concentration (CC_50_), and a selectivity index (SI), which is the CC_50_/IC_50_ ratio, are presented in Table 1. 

**Figure 3:**
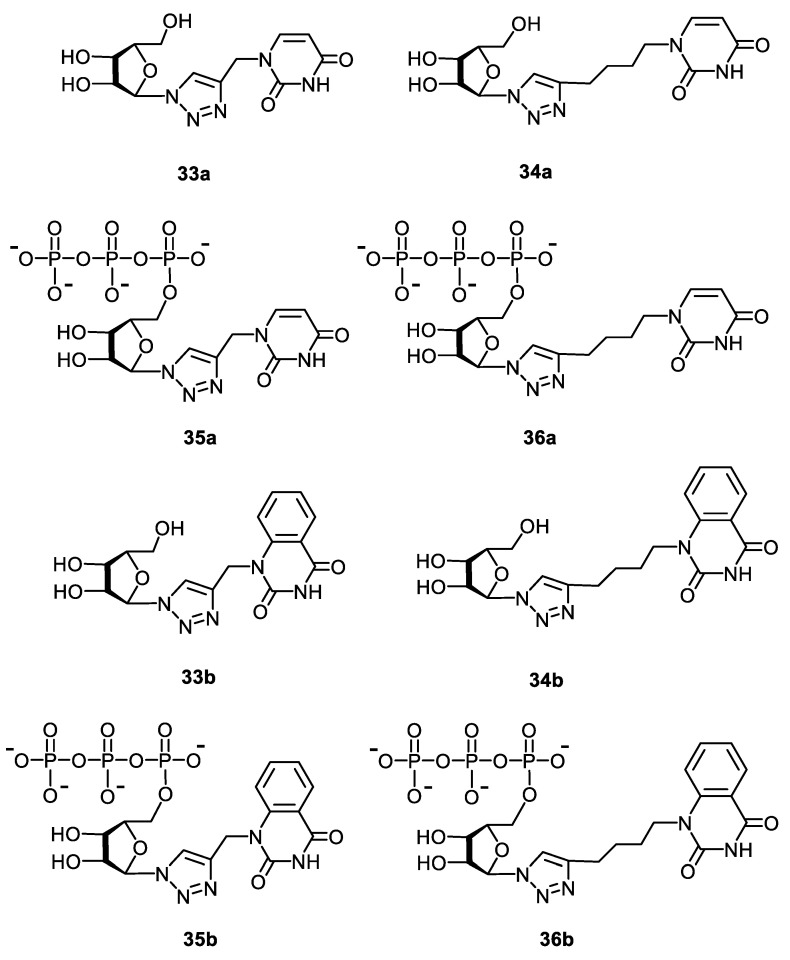
Parent 1,2,3-triazolyl nucleoside analogues **33a**,**b** and **34a**,**b** and their 5′-triphosphate derivatives **35a**,**b** and **36a**,**b**.

In addition, the data on the antiviral activity of the previously studied parent 1,2,3-triazolyl nucleoside analogues **33a**,**b** and **34a**,**b** (Figure 3) are also given in the Table. That is, in Table 1, one can compare the antiviral activity of the parent 1,2,3-triazolyl nucleoside analogues **33a**,**b** and **34a**,**b**; their prodrug forms, namely diethyl phosphates **11a**,**b** and **12a**,**b**, diphenyl phosphates **13a**,**b** and **14a**,**b**, phosphoramidates **17a**,**b** and **18a**,**b**; and their *H*-phosphonate derivatives **23a**,**b** and **24a**,**b**, and monophosphate derivatives **25a**,**b** and **26a**,**b**.

Note that among the parent nucleoside analogues **33a**,**b** and **34a**,**b** (Figure 3), 1,2,3-triazolyl derivatives of uridine **33a** and **34a** were inactive. Moderate activity against H1N1 (A/PR/8/34) influenza virus was demonstrated by compounds **33b** and **34b** (IC_50_ values are 42 and 30 μM, respectively), in which the uracil moiety was replaced by the quinazoline-2,4-dione moiety. Among all synthesized 5′-phosphorylated derivatives (i.e., prodrug forms) of nucleoside analogue **33b**, the only compound, namely diphenyl phosphate **13b**, showed antiviral activity, which, by the way, exceeded the activity of the parent compound **33b** by 2 times (IC_50_ = 17.9 vs. IC_50_ = 42 μM), and the selectivity index increased by 7 times (SI = 28 vs. SI = 3). Similarly, among all synthesized 5′-phosphorylated derivatives (i.e., prodrug forms) of the nucleoside analogue **34b** (Figure 3), antiviral activity was demonstrated by only one compound, namely diphenyl phosphate **14b**. Its activity approximately corresponded to the activity of the parent compound **34b** (IC_50_ = 51 μM vs. IC_50_ = 30 μM), although its cytotoxicity increased 7-fold (CC_50_ = 98 μM vs. CC_50_ = 710 μM). Regarding 1,2,3-triazolyl derivative of uridine **34a**, all its synthesized 5′-phosphorylated derivatives, namely prodrug forms **12a**, **14a**, and **18a,** and charged derivatives **24a** and **26a,** were inactive against H1N1 (A/PR/8/34) influenza virus. At the same time, to our surprise, the 5′-phosphoramidate derivative **17a** of the uridine analogue **33a** appeared to be moderately active against H1N1 (A/PR/8/34) influenza virus (IC_50_ = 25 μM) with a good selectivity index of S = 21, although its parent compound **33a** did not show antiviral activity against this virus.

By analyzing the results obtained from the point of view of the literature data presented in the Introduction section on the penetration of nucleoside analogues into the cell and their metabolism in the cell into active triphosphate forms [1,7,8,9], the following conclusions can be made. Firstly, the fact that all synthesized *H*-phosphonates **23a**,**b** and **24a**,**b** and phosphates **25a**,**b** and **26a**,**b** did no show antiviral activity is easily explained by the presence of negative charges preventing their penetration into the cell, and indicates that receptor proteins that are capable of transporting organic anions into the cell [9,45] were unsuitable for transporting *H*-phosphonates **23a**,**b** and **24a**,**b** and phosphates **25a**,**b** and **26a**,**b** into the cell. Secondly, according to the literature data discussed above [1,7,8,9], antiviral activity characterized by IC_50_ values of 42 μM and 30 μM was shown not by the parent nucleoside analogues **33b** and **34b**, but by their 5′-triphosphate derivatives **35b** and **36b** (Figure 3). That is, compounds **33b** and **34b** somehow (e.g., with the help of corresponding transport proteins) penetrated into the cell, wherein they were sequentially metabolized under the action of cellular kinases (phosphotransferases) first to their 5′-monophosphate derivatives **25b** and **26b**, then to their 5′-diphosphate derivatives, and finally, to 5′-triphosphate derivatives **35b** and **36b** (Figure 3), which somehow inhibited the replication of the virus [1,7,8,9]. Thirdly, according to the literature data [1,7,8,9], diphenyl phosphates **13b** and **14b** after penetration into the cell were enzymatically converted into monophosphates **25b** and **26b**, which were subsequently metabolized by cellular kinases into the corresponding active 5′-triphosphate forms **35b** and **36b** (Figure 3), which have shown antiviral activity with IC_50_ values of 17.9 and 51 μM. Therefore, it is not surprising that diphenyl phosphate **14b**, which is the prodrug form of the nucleoside analogue **34b**, showed approximately the same antiviral activity as the parent compound **34b** (IC_50_ = 51 μM vs. IC_50_ = 30 μM). In both cases, according to the literature data [1,7,8,9], virus replication was inhibited by the 5′-triphosphate derivative **36b,** which formed during intracellular metabolism (Figure 3).

It may seem strange that diphenyl phosphate **13b**, being the prodrug form of the nucleoside analogue **33b**, showed antiviral activity that was twice as high as the activity of the parent compound **33b** (IC_50_ = 17.9 μM vs. IC_50_ = 42 μM), although according to the literature data [1,7,8,9], in both cases, 5′-triphosphate **36b** was the active form. However, the literature has provided many examples of when the antiviral activity of a prodrug form—because of some unknown reason—significantly exceeded the antiviral activity of a parent nucleoside analogue [21,22,23,27,30,31,32], or even when a prodrug form showed antiviral activity that was absent from a parent nucleoside analogue [24]. Apparently, such a case can be observed for the 1,2,3-triazolyl analogue of uridine **33a** and its prodrug form, phosphoramidate **17a**. The 1,2,3-triazolyl analogue of uridine **33a** was completely inactive against H1N1 (A/PR/8/34) influenza virus, while its prodrug form, phosphoramidate **17a,** demonstrated moderate inhibitory activity against this virus (IC_50_ = 25 μM) and a good selectivity index (SI = 21). Taking into account the literature data on the metabolism of nucleoside analogues and their prodrug forms inside the cell [1,7,8,9], which were discussed in the Introduction section, we can assume the following reasons for the absence of antiviral activity for the 1,2,3-triazolyl analogue of uridine **33a** and moderate antiviral activity for its prodrug form, phosphoramidate **17a**: (i) the nucleoside analogue **33a** for some reason could not penetrate into the cell; (ii) the nucleoside analogue **33a** penetrated into the cell but was not recognized by cellular kinases, that is, the process of its phosphorylation to the active 5′-triphosphate form **35a** (Figure 3) did not start; (iii) phosphoramidate **17a**, in its differences from diethyl phosphate **11a** and diphenyl phosphate **13a,** somehow penetrated into the cell; and (iv) phosphoramidate **17a** was enzymatically hydrolyzed into monophosphate **25a**, which was then, according to the literature data [16], converted by cellular kinases into triphosphate **35a**, and it was triphosphate **35a** that showed antiviral activity against H1N1 (A/PR/8/34) influenza virus with the IC_50_ value of 25 μM and the SI value of 21 (Table 1).

As for the 1,2,3-triazolyl analogue of uridine **34a**, neither it nor its prodrug forms, namely diethyl phosphate **12a**, diphenyl phosphate **14a**, and phosphoramidate **18a**, have demonstrated any antiviral activity against H1N1 (A/PR/8/34) influenza virus. The reason can be assumed to be one single but significant difference in the structure of these compounds from the structure of the 1,2,3-triazolyl analogue of uridine **33a** and its prodrug forms **11a**, **13a**, and **17a**. This difference is the remoteness of the uracil moiety from the 1,2,3-triazolylribofuranosyl fragment by a considerable distance (butylene linker instead of methylene).

### 2.3. Molecular Docking

**Figure 4:**
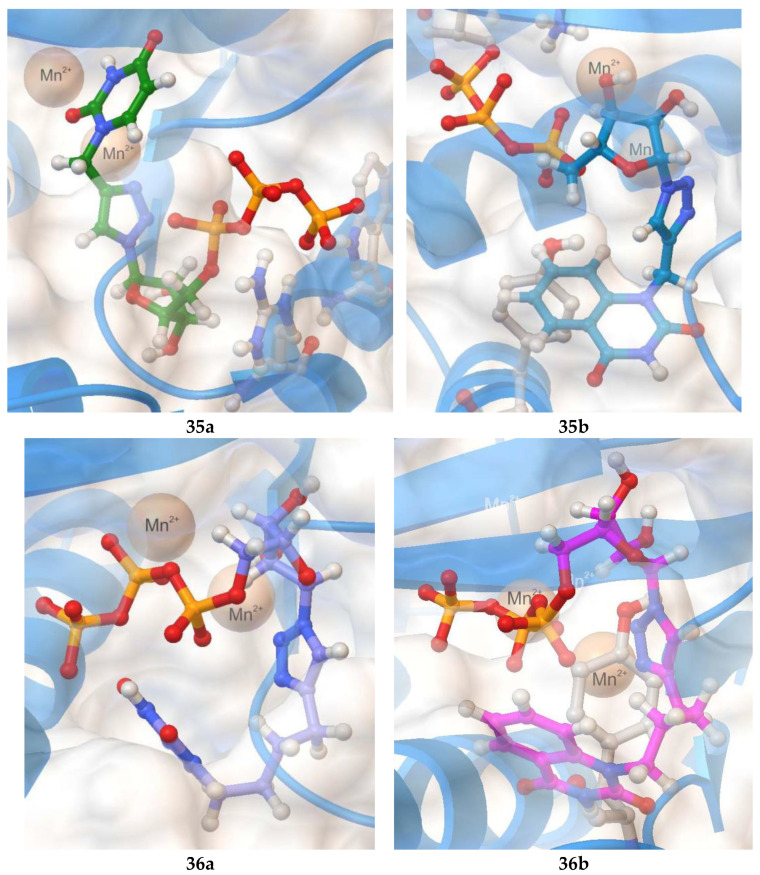
Molecular docking simulations of the optimized docking model of compounds **35a**,**b** and **36a**,**b** in the PA-Nter (PDB code 4AWK) active site of the RdRp of influenza virus A (H1N1) were obtained in the lowest-energy conformations.

Continuing the interpretation of the data obtained on the antiviral activity of the synthesized compounds (Table 1) from the point of view of the literature data, wherein despite whatever nucleoside analogues or their prodrug forms are introduced into the cell, it is their 5′-triphosphate derivatives that inhibit viral replication [1,7,8,9], we carried out molecular docking of triphosphates **35a**,**b** and **36a**,**b** (Figure 3). We chose the N-terminal endonuclease domain of the polymerase acidic polypeptide (PA-Nter) of the RNA-dependent RNA polymerase (RdRp) of influenza virus A (H1N1) as a drug target [59]. The PA-Nter domain has a cation-dependent endonuclease active-site core that is responsible for viral RNA replication (PDB code 4AWK [59]). A distinctive feature of this active site is that its structure and topology are approximately the same for all RNA viruses [60,61], including all influenza A subtypes and strains [62,63]. In addition, this site is not very selective to the structure of substrates [60,61]. All of the above makes the PA-Nter domain of RdRp an attractive drug target. Thus, we have evaluated the binding of triphosphates **35a**,**b** and **36a**,**b** to the PA-Nter endonuclease domain (PDB code 4AWK [59]). The positions of the optimized docking models of the compounds demonstrating the best binding energy in the PA-Nter active site are shown in Figure 4.

According to the molecular docking calculation, the compounds **35b**, **36b**, and **36a** are approximately located in one region of space and their triphosphate fragments are localized deep in the cavity of the P-Nter active site. On the contrary, compound **35a** is localized in such a way that its triphosphate fragment is located at the mouth of this cavity. This arrangement of the studied molecules seems surprising because compound **36a,** which possesses the uracil moiety, is not located in the same region of the active site in which compound **35a,** which also possesses the uracil moiety, but rather is located in the region in which compounds **35b** and **36b,** which possess the quinazoline-2,4-dione moiety, are located.

The reason can be assumed to be a significant structural difference between compounds **35a** and **36a**. If in the first compound only the methylene unit separates the uracil moiety from the 1,2,3-triazolylribofuranosyl fragment, then in the second one the uracil moiety is kept away from the 1,2,3-triazolylribofuranose fragment by the length of the butylene chain and the significantly increased volume of compound **36a,** which does not allow it to localize in the same area of space as compound **35a**. However, once in the same region of space as compounds **35b** and **36b**, compound **36a** did not find amino acid residues with which it could form hydrogen bonds.

As a result, compound **36a** is retained in the active site only due to van der Waals interactions and shows the lowest binding energy among the studied compounds (7.8 kcal/mol, Table 2). The compounds **35b** and **36b** are retained in the cavity of the active site by hydrogen bonding of their triphosphate fragments with the amino acid residue Val122 and the π-π stacking interaction of their quinazoline-2,4-dione moiety with the amino acid residue, Tyr24.

The hydrogen bonding of the triphosphate fragment of compound **35b** with the Lys134 amino acid residue provides a stronger retention of **35b** in the cavity of the P-Nter active site compared to **36b**. Accordingly, the binding energy of compound **35b** (9.8 kcal/mol) is higher than the binding energy of compound **36b** (8.9 kcal/mol, Table 2). The compound **35a**, being localized in another region of the cavity of the active P-Nter site, is well retained in it due to the hydrogen bonding of its triphosphate fragment with the amino acid residues, Arg84 and Trp88, as evidenced by the binding energy of 9.1 kcal/mol (Table 2).

Thus, according to the molecular docking calculation, triphosphate **35b** has the highest binding energy to the PA-Nter active site among the compounds studied (9.8 kcal/mol). The binding energies of **35a** and **36b** are worse (9.1 and 8.9 kcal/mol, respectively). Triphosphate **36a** has the lowest binding energy (7.8 kcal/mol). It can be seen that the trend of antiviral activity of prodrug forms **13b**, **14b**, and **17a** of the parent 1,2,3-triazolyl nucleoside analogues **33a**,**b** and **34a**,**b** (Figure 3) correlates with the trend of the binding energy of 5′-triphosphate derivatives **35a**,**b** and **36a**,**b** of the parent 1,2,3-triazolyl nucleoside analogues **33a**,**b** and **34a**,**b** with the active site of RdRp. Indeed, the decrease in antiviral activity during the transition from the lead compound diphenyl phosphate **13b** with maximum antiviral activity in the studied series of compounds (IC_50_ = 17.9 μM) to phosphoramidate **17a** with moderate antiviral activity (IC_50_ = 25 μM) correlates with the decrease of binding energy during the transition from the corresponding 5′-triphosphate **35b** (9.1 kcal/mol) to 5′-triphosphate **35a** (8.2 kcal/mol). Similarly, the transition from diphenyl phosphate **14b,** which showed moderate antiviral activity (IC_50_ = 51 μM), to the completely inactive parent 1,2,3-triazolyl nucleoside analog **34a** and its inactive prodrug forms **12a**, **14a**, and **18a** (IC_50_ > 180 μM) correlates with a significant decrease in the binding energy during the transition from the corresponding 5′-triphosphate **36b** (8.4 kcal/mol) to 5′-triphosphate **36a** (7.8 kcal/mol). Thus, in our opinion, the results of the molecular docking simulation indirectly confirm the literature data [1,7,8,9], which suggests that it is the 5′-triphosphate derivatives of nucleoside analogues that inhibit viral replication (See Supplementary Materials).

## 3. Conclusions

In summary, a series of prodrug forms of previously-studied [47] parent 1,2,3-triazolyl nucleoside analogues **33a**,**b** and **34a**,**b** (Figure 3) were synthesized. These prodrug forms are 5′-diethyl phosphates **11a**,**b** and **12a**,**b**; 5′-diphenyl phosphates **13a**,**b** and **14a**,**b**; and 5′-phosphoramidates **17a**,**b** and **18a**,**b**. In addition, 5′-*H*-phosphonate derivatives **23a**,**b** and **24a**,**b** and 5′-monophosphate derivatives **25a**,**b** and **26a**,**b** of parent nucleoside analogues **33a**,**b** and **34a**,**b** were synthesized. In all these compounds, the 5′-phosphorylated 1,2,3-triazole-4-yl-β-D-ribofuranose fragment is attached via the methylene unit or butylene chain to the *N*-1 atom of the heterocycle moiety (uracil or quinazoline-2,4-dione). Antiviral assays against H1N1 (A/PR/8/34) influenza virus revealed three prodrugs, namely 5′-diphenylphosphates **13b** and **14b** and 5′-phosphoramidates **17a,** which showed moderate activity against this virus with IC_50_ values of 17.9 µM, 51 µM, and 25 µM, respectively. Prodrug forms **13b** and **17a** showed good selectivity indices (28 and 21, respectively), while prodrug form **14b** demonstrated a low selectivity index (SI = 2) due to its high toxicity (CC_50_ = 98 µM). All other compounds appeared to be inactive against H1N1 (A/PR/8/34) influenza virus. Taking into account the literature data that virus replication is inhibited not by the nucleoside analogues themselves, but by their 5′-triphosphate derivatives [1,7,8,9], we carried out molecular docking simulations of 5′-triphosphate derivatives **35a**,**b** and **36a**,**b** of the parent nucleoside analogues **33a**,**b** and **34a**,**b** (Figure 3). The N-terminal endonuclease domain of the polymerase acidic polypeptide (PA-Nter) of the RNA-dependent RNA polymerase (RdRp) of the H1N1 (A/PR/8/34) influenza virus was used as a drug target. It was found that the antiviral activity of prodrug forms **13b**, **14b**, and **17a** in terms of IC_50_ values correlates with the binding energies of the corresponding triphosphates **35b**, **36b**, and **35a**. Thus, the maximum antiviral activity among the number of studied compounds (IC_50_ = 17.9 μM) shown by diphenyl phosphate **13b,** which possesses the quinazoline-2,4-dione moiety as a nucleic base, corresponds to the maximum binding energy (9.8 kcal/mol, Table 2) of the corresponding triphosphate **35b** with amino acid residues of the active site PA-Nter of the RdRp of H1N1 (A/PR/8/34) influenza virus. 

The absence of antiviral activity against influenza A (H1N1) virus (IC_50_ > 180 μM) of the parent nucleoside analogue **34a** with the uracil moiety as a nucleic base and its prodrug forms **12a**, **14a**, and **18a** correlates with the minimum binding energy (7.8 kcal/mol) of the corresponding triphosphate **36a** (Table 2). Thus, the results obtained indirectly confirm the literature data that the inhibition of viral replication is carried out not by nucleoside analogues themselves, but by their 5-triphosphate derivatives [1,7,8,9].

## Figures and Tables

**Table 1:** Antiviral activity against A/Puerto Rico/8/34 (H1N1) influenza virus and cytotoxicity of 5′-phosphorylated 1,2,3-triazolyl nucleoside analogues **11a**,**b**–**14a**,**b**; **17a**,**b**; **18a**,**b**; **23a**,**b**–**26a**,**b**; and their parent compounds **33a**,**b** and **34a**,**b**.

Compound	CC_50_^a^ (μM)	IC_50_^b^ (μM)	SI ^c^	Compound	CC_50_^a^ (μM)	IC_50_^b^ (μM)	SI ^c^
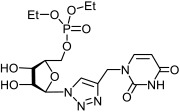 **11a**	>650	434 ± 39	2	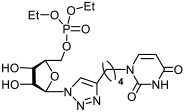 **12a**	>596	250 ± 22	2
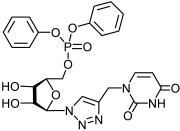 **13a**	>538	121 ± 7	4	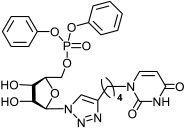 **14a**	>500	183 ± 15	3
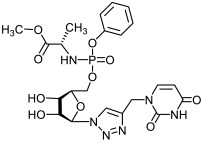 **17a**	>530	25 ± 4	21	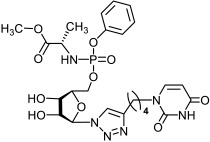 **18a**	>463	>463	1
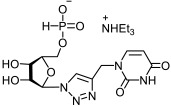 **23a**	>612	204 ± 28	3	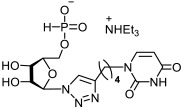 **24a**	>564	218 ± 32	3
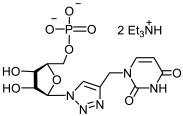 **25a**	>494	>494	1	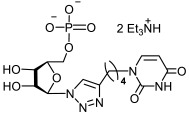 **26a**	>463	195 ± 26	2
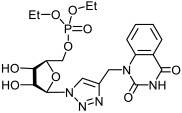 **11b**	>587	411 ± 51	1	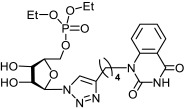 **12b**	>542	>542	1
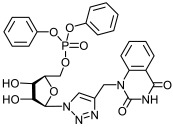 **13b**	>494	17.9 ± 3	28	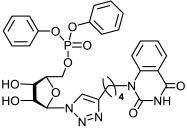 **14b**	98 ± 6	51 ± 4	2
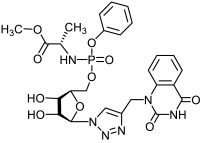 **17b**	>487	>487	1	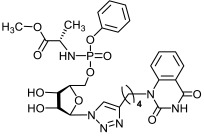 **18b**	>456	>456	1
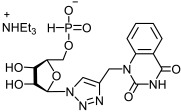 **23b**	>555	176 ± 21	3	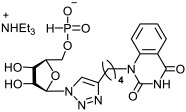 **24b**	>516	>516	1
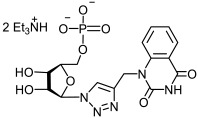 **25b**	>458	159 ± 19	3	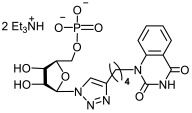 **26b**	>430	>430	1
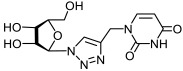 **33a**	>880 ^d^	>880 ^d^	1	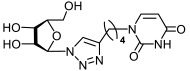 **34a**	>817 ^d^	343 ± 41	2
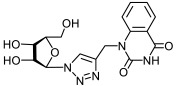 **33b**	132 ± 9 ^d^	42 ± 5 ^d^	3	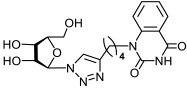 **34b**	>719 ^d^	30 ± 4 ^d^	24
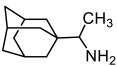							
Rimantadine	340 ± 16	77 ± 8	4				
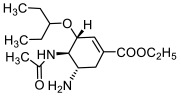 Ribavirin	94 ± 48	31 ± 9	3				
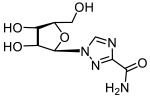 Oseltamivir carboxylate	>200	0.3 ± 0.06	>667				

^a^ CC_50_ is the median cytotoxic concentration, i.e., the concentration causing 50% cell death. ^b^ IC_50_ is the concentration causing 50% inhibition of virus replication. ^c^ SI is the selectivity index, which is the CC_50_/IC_50_ ratio. ^d^ Data from [47].

**Table 2:** Binding energies and ligand–protein interactions of 5′-triphosphates **35a,b** and **36a,b** obtained by molecular docking simulations.

Compound	−E_bind_ (kcal/mol)	Ligand-Protein Interactions
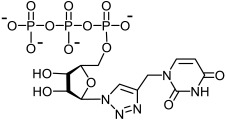 **35a**	9.1	H bonding: Arg84, Trp88
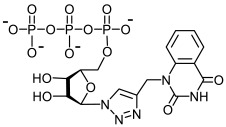 **35b**	9.8	H bonding: Val122, Lys134 π-π: Tyr24
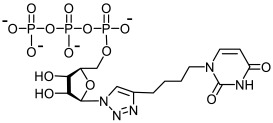 **36a**	7.8	no H bonding
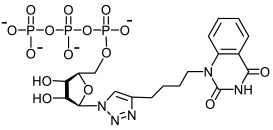 **36b**	8.9	H bonding: Glu80, Val122 π-π: Tyr24

## Data Availability

The data presented in this study are available on request from V.E.K.

## Supplementary Materials

The following supporting information can be downloaded at: https://www.mdpi.com/article/10.3390/molecules27196214/s1, general information (S2), procedures for the synthesis of the compounds (S3, S5, S10–S11, S17–S18, S23), characterization of the compounds synthesized (S3–S5, S5–S10, S11–S17, S18–S22, S23–S28) including their NMR spectra (Figures S1–S101), description of the antiviral assays (S130) and the molecular docking study (S130). References [46,64,65,66,67,68,69,70] are cited in the Supplementary data.

## Sample Availability

Samples of the compounds are not available.
