# Supplementary material for: The First 5′-Phosphorylated 1,2,3-Triazolyl Nucleoside Analogues with Uracil and Quinazoline-2,4-Dione Moieties: A Synthesis and Antiviral Evaluation
Source: Molecules. 2022 Sep 21;27(19):6214. doi: 10.3390/molecules27196214 (PMC9573387; doi:10.3390/molecules27196214)
Supplement: Supplementary file 1 [file molecules-27-06214-s001.zip › molecules-1901653-supplementary.pdf]

Supplementary data

The first 5′-phosphorylated 1,2,3-triazolyl nucleoside analogues with uracil and quinazoline-2,4-dione moieties. Synthesis and antiviral evaluation

Dmitriy A. Tatarinov[1,*], Bulat F. Garifullin[1], Mayya G. Belenok[1], Olga V. Andreeva[1], Irina Yu. Strobykina[1], Anna V. Shepelina[2], Vladimir V. Zarubaev[3], Alexander V. Slita[3], Alexandrina S. Volobueva[3], Liliya F. Saifina[1], Marina M. Shulaeva[1], Vyacheslav E. Semenov[1] and Vladimir E. Kataev[1]

[1]Arbuzov Institute of Organic and Physical Chemistry, FRC Kazan Scientific Center, Russian Academy of Sciences, Arbuzov Str., 8, 420088 Kazan, Russia

[2] Department of Organic and Medicine Chemistry, Kazan Federal University, Kremlevskaya 18, 420008 Kazan, Russia

[3]Pasteur Institute of Epidemiology and Microbiology, Mira Str., 14, 197101 Saint Petersburg, Russia

* Correspondence: datint@iopc.ru (Dmitriy A. Tatarinov)

Content:

1. General information …… 2

2. Characterization of 2′,3′-*O*-isopropylidene protected 1′′,2′′,3′′-triazolyl nucleoside analogues …… 3

3. General procedure for the synthesis of 5′-diethyl and 5′-diphenyl phosphates of 1′′,2′′,3′′-triazolyl nucleoside analogues …… 5

3.1 Characterization of compounds …… 5

4. General procedure for the synthesis of 5′-(phenyl methoxy-L-alaninyl)phosphates of 1′′,2′′,3′′-triazolyl nucleoside analogues …… 10

4.1 Characterization of compounds …… 11

5. General procedure for the synthesis of 5′-*H*-phosponates of 1′′,2′′,3′′-triazolyl nucleoside analogues …… 17

5.1 Characterization of compounds …… 18

6. General procedure for the synthesis of 5′- phosphates of 1′′,2′′,3′′-triazolyl nucleoside analogues …… 23

6.1 Characterization of compounds …… 23

7. NMR spectra …… 29

8. Antiviral assay …… 130

9. Molecular docking study …… 130

11. References …… 130

1. General information

^{1}H NMR spectra were recorded on 400 MHz and 600 MHz Brucker Advance. ^{13}C NMR spectra were obtained in the above instrument operating at 100.6 MHz. Mass spectra (MALDI) were recorded in a positive ion mode on a Bruker Ultraflex III TOF/TOF mass spectrometer for 10^{-3} mg/mL solutions in MeOH. The ESI MS measurements were performed using an AmazonX ion trap mass spectrometer (Bruker Daltonic GmbH, Germany) in the positive or negative mode in the mass range of 70–3000.The capillary voltage was 3500 V, nitrogen drying gas 10 L·min^{-1}, desolvation temperature 250°C. A methanol/water solution (70:30) was used as a mobile phase at a flow rate of 0.2 mL/min by binary pump (Agilent 1260 chromatograph, USA). The sample was dissolved in methanol to a concentration of 10^{-6} g·L^{-1}. Flash chromatography was performed on silica gel 60 (40-63 μm, Buchi, Sepacore). Thin-layer chromatography was carried out on plates with silica gel (Sorbfil, Russia). Spots of compounds were visualized by using ultraviolent fluorescence under a short wavelength (254 nm) followed by heating the plates (at ca. 150 °C) after immersion in a solution of 5% H_2SO_4 and 95% H_2O. All reactions sensitive to air and/or moisture were carried out under argon atmosphere with anhydrous solvents. Anhydrous solvents were purified and dried (where appropriate) according to standard procedures.

Uracil **1a**, quinazoline-2,4-dione **1b** and propargyl bromide were purchased from Sigma-Aldrich; D-ribose was purchased from Acros. 6-Iodo-hex-1-yn was prepared from purchased 6-chloro-1-hexyne (Sigma-Aldrich) by its reaction with sodium iodide in acetone. Compounds **3a,b**, **4a,b,c**, **5a,b**, **6a,b**, **7a,b**, **8a,b** were prepared as described earlier [1s]. Their spectral data were in keeping with published ones [1s]. Compounds **9a,b**, **10a,b** were synthesized by analogy with a procedure previously described [2s]. Their spectral data are presented below.

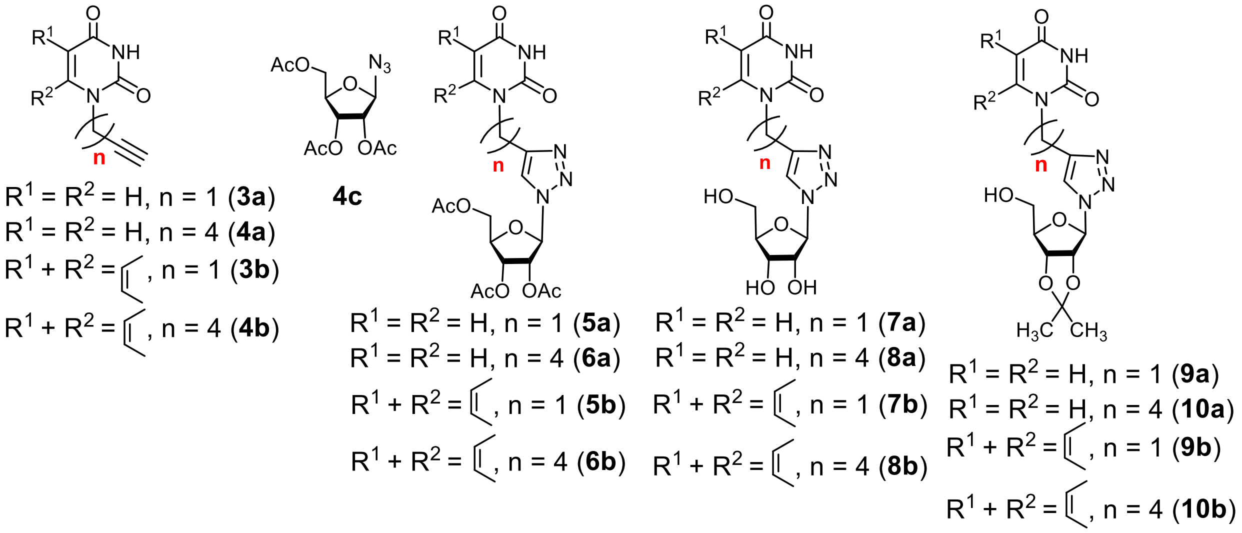

2.1 Characterization of 2′,3′-*O*-isopropylidene protected 1,2,3-triazolyl nucleoside analogues

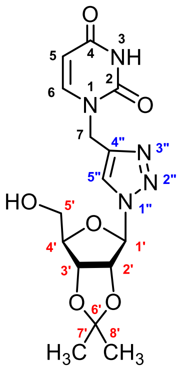

1′-[4″-(2,4-dioxo-pyrimidine-1-yl-methyl)-1″,2″,3″-triazol-1″-yl]-2′,3′-*O*-isopropylidene-β-D-ribofuranose (9a).

A white foam, 86% yield. ^{1}H NMR ($CDCl_3$, 400 MHz): δ 9.34 (s, 1H, NH), 8.11 (s, 1H, H-5″), 7.50 (d, 1H, J = 7.9 Hz, H-6), 6.13 (d, 1H, J = 2.5 Hz, H-1′), 5.72 (d, 1H, J = 7.9 Hz, H-5), 5.15 (dd, 1H, J = 5.9, 2.4 Hz, H-3′), 5.02 - 4.93 (m, 3H, H-2′, H-7), 4.55-4.52 (m, 1H, H-4′), 3.84 (d, 1H, J = 11.5 Hz, H-5a′), 3.72 - 3.61 (m, 2H, H-5b′, OH), 1.59 (s, 3H, H-7′), 1.36 (s, 3H, H-8′). ^{13}C NMR ($CDCl_3$, 100 MHz): δ 163.82 (s, C=O, C-4), 151.30 (s, C=O, C-2), 144.42 (s, C-4″), 141.79 (s, C-6), 123.81 (s, C-5″), 113.84 (s, C-6′), 102.83 (s, C-5), 95.31 (s, C-1′), 88.57 (s, C-4′), 85.64 (s, C-3′), 81.58 (s, C-2′), 62.84 (s, C-5′), 43.36 (s, C-7), 27.01, 25.11 (s, C-7′, C-8′). MALDI MS *m/z*: calcd. for $C_{15}H_{19}N_5O_6$ $[M+Na]^+$ 388.3; $[M+K]^+$ 404.4, found $[M+Na]^+$ 388.1, $[M+K]^+$ 404.1. Anal., %: C 49.28, H 5.33, N 19.14. $C_{15}H_{19}N_5O_6$. Calcd., %: C 49.31, H 5.24, N 19.17.

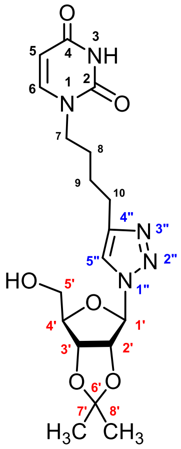

1′-[4″-(2,4-dioxo-pyrimidine-1-yl-butyl)-1″,2″,3″-triazol-1″-yl]-2′,3′-*O*-isopropylidene-β-D-ribofuranose (10a).

A white foam, 83% yield. ^{1}H NMR ($CDCl_3$, 400 MHz): δ 9.96 (s, 1H, NH), 7.61 (s, 1H, H-5″), 7.16 (d, 1H, J = 7.9 Hz, H-6), 6.05 (d, 1H, J = 2.0 Hz, H-1′), 5.61 (d, 1H, J = 7.9 Hz, H-5), 5.23 (dd, 1H, J = 6.0, 2.0 Hz, H-2′), 4.95 (dd, 1H, J = 6.0, 1.6 Hz, H-3′), 4.46-4.41 (m, 1H, H-4′), 3.72 – 3.64 (m, 3H, H-5a′, H-7), 3.54 (dd, 1H, J = 12.3, 4.4 Hz, H-5b′), 2.73-2.66 (m, 2H, H-10), 1.72-1.62 (m, 4H, H-8, H-9), 1.53 (s, 3H, H-7′), 1.32 (s, 3H, H-8′). ^{13}C NMR ($CDCl_3$, 100 MHz): δ 164.04 (s, C=O, C-4),

151.04 (s, C=O, C-2), 147.07 (s, C-4′′), 144.53 (s, C-6), 121.19 (s, C-5′′), 113.51 (s, C-6′), 102.07 (s, C-5), 94.55 (s, C-1′), 88.58 (s, C-4′), 85.12 (s, C-3′), 81.66 (s, C-2′), 62.70 (s, C-5′), 48.28 (s, C-7), 27.97 (s, C-10), 26.84, 24.99 (s, C-7′, C-8`), 25.70 (s, C-8), 24.52 (s, C-9). MALDI MS *m/z*: calcd. for $C_{18}H_{25}N_5O_6$ $[M+Na]^+$ 430.4; $[M+K]^+$ 446.5, found $[M+Na]^+$ 430.3, $[M+K]^+$ 446.3. Anal., %: C 52.99, H 6.22, N 17.17. $C_{18}H_{25}N_5O_6$. Calcd., %: C 53.06, H 6.19, N 17.19.

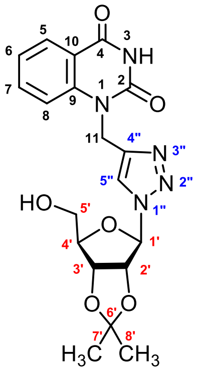

1′-[4′′-(2,4-dioxo-quinazoline-1-yl-methyl)-1′′,2′′,3′′-triazol-1′′-yl]-2′,3′-*O*-isopropylidene-β-D-ribofuranose (9b).

A white foam, 77% yield. ^{1}H NMR ($CDCl_3$, 400 MHz): *δ* 10.03 (s, 1H, NH), 8.17 (s, 1H, H-5′′), 8.11 (d, 1H, *J* = 7.9 Hz, H-ar), 7.76 (d, 1H, *J* = 8.4 Hz, H-ar), 7.72-7.66 (m, 1H, H-ar), 7.23 (t, 1H, *J* = 7.5 Hz, H-ar), 6.14 (d, 1H, *J* = 1.2 Hz, H-1′), 5.36 (q, 2H, *J* = 15.8 Hz, H-11), 5.14 (dd, 1H, *J* = 5.9, 2.3 Hz, H-3′), 4.99 (dd, 1H, *J* = 5.8, 1.7 Hz, H-2′), 4.53-4.49 (m, 1H, H-4′), 3.91-3.79 (m, 2H, H-5a′, OH), 3.72-3.63 (m, 1H, H-5b′), 1.57 (s, 3H, H-7′), 1.34 (s, 3H, H-8′). ^{13}C NMR (CD_3OD, 100 MHz): *δ* 164.19 (s, C=O, C-4), 152.35 (s, C=O, C-2), 144.72 (s, C-4′′), 142.35, 136.69, 129.17, 124.42, 124.35, 117.47, 116.16 (s, C-5, C-6, C-7, C-8, C-9, C-10, C-5′′), 114.91 (s, C-6′), 95.71 (s, C-1′), 90.05 (s, C-4′), 86.14 (s, C-3′), 83.29 (s, C-2′), 63.10 (s, C-5′), 39.17 (s, C-11), 27.28, 25.41 (s, C-7′, C-8′). MALDI MS *m/z*: calcd. for $C_{19}H_{21}N_5O_6$ $[M+Na]^+$ 438.4; found $[M+Na]^+$ 438.4. Anal., %: C 54.90, H 5.18, N 16.83. $C_{19}H_{21}N_5O_6$. Calcd., %: C 54.94, H 5.10, N 16.86.

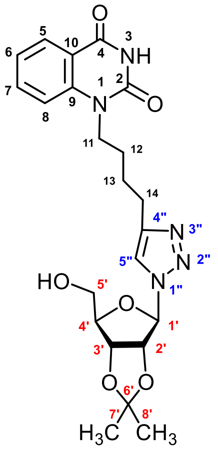

1′-[4″-(2,4-dioxo-quinazoline-1-yl-butyl)-1″,2″,3″-triazol-1″-yl]-2′,3′-*O*-isopropylidene-β-D-ribofuranose (10b).

A white foam, 65% yield. ^{1}H NMR ($CDCl_3$, 500 MHz): δ 9.18 (s, 1H, NH), 8.20 (dd, 1H, J = 7.8, 1.6 Hz, H-ar), 7.72-7.66 (m, 1H, H-ar), 7.26 (s, 1H, H-5″),7.28-7.19 (m, 2H, H-ar), 6.07 (d, 1H, J = 2.1 Hz, H-1′), 5.26 (dd, 1H, J = 5.9, 2.1 Hz, H-2′), 5.02 (dd, 1H, J = 6.0, 1.6 Hz, H-3′), 4.54-4.50 (m, 1H, H-4′), 4.14-4.09 (m, 2H, H-11), 3.88-3.76 (m, 2H, H-5a′, OH), 3.67-3.59 (m, 1H, H-5b′), 2.82 (t, 2H, J = 6.9 Hz, H-14), 1.88-1.73 (m, 4H, H-12, H-13), 1.58 (s, 3H, H-7′), 1.37 (s, 3H, H-8′). ^{13}C NMR (CD_3OD, 100 MHz): δ 161.87 (s, C=O, C-4), 150.46 (s, C=O, C-2), 147.30 (s, C-4″),140.80, 135.57, 128.88, 123.05, 121.42, 116.12, 114.03, 113.60 (s, C-5, C-6, C-7, C-8, C-9, C-10, C-5″, C-6′), 94.95 (s, C-1′), 88.91 (s, C-4′), 85.49 (s, C-3′), 81.83 (s, C-2′), 63.21 (s, C-5′), 42.48 (s, C-11), 26.97 (s, C-14), 26.27, 26.12, 25.08, 24.70 (s, C-12, C-13, C-7′, C-8′). MALDI MS *m/z*: calcd. for $C_{22}H_{27}N_5O_6$ $[M+Na]^+$ 480.5; found $[M+Na]^+$ 480.4. Anal., %: C 57.74, H 5.98, N 15.35. $C_{22}H_{27}N_5O_6$. Calcd., %: C 57.76, H 5.95, N 15.31.

3. General procedure for the synthesis of 5′-diethyl and 5′-diphenyl phosphates of 1″,2″,3″-triazolyl nucleoside analogues

Starting nucleoside **7a,b** or **8a,b** (1 eq) was heated *in vacuo* (60°C, 0.05 Torr) for 30 min, then the flask was filled with argon, and this operation was repeated 2 more times. The dried nucleoside was dissolved in 3–5 ml of freshly distilled (over CaH_2) pyridine, a flask was equipped with a rubber septum, argon inlet tube and cooled with a water bath (18–20°C). Then 2.2–2.5 eq of the corresponding chlorophosphate (diethyl or diphenylchlorophosphate) was added with a syringe under an argon atmosphere. The flask was tightly closed using a PTFE seal and left to stir for 12-24 h at room temperature. Upon completion of the reaction (TLC and ^{31}P NMR control), 1 ml of dry MeOH was added to the resulting mixture and left to stir for another 30 min, then all volatile components were removed using a water jet pump and dried *in vacuo* (40°C, 0.05 Torr). The residue was purified by flash chromatography to isolate the target product (eluent - a mixture of chloroform: ethanol 10 : 1).

3.1 Characterization of compounds

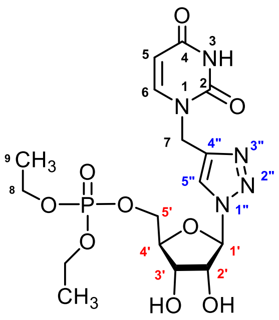

1′-[4″-(2,4-dioxo-pyrimidine-1-yl-methyl)-1″,2″,3″-triazol-1″-yl]-β-D-ribofuranose-5′-yl diethyl phosphate (11a).

Creamy powder. Yield 50%. ^{1}H NMR (MeOD, 400 MHz): δ 8.15 (s, 1H, H-5’’), 7.71 (d, *J* = 7.9 Hz, 1H, H-6), 6.04 (d, *J* = 3.3 Hz, 1H, H-1’), 5.68 (d, *J* = 7.9 Hz, 1H, H-5), 5.03 (s, 2H, H-7), 4.57 (dd, *J* = 5.0, 3.3 Hz, 1H, H-2’), 4.42 (t, *J* = 5.3 Hz, 1H, H-3’), 4.30 – 4.22 (m, 2H, H-5’), 4.22 – 4.13 (m, 1H, H-4’), 4.13 – 4.00 (m, 4H, H-8), 1.34 – 1.33 (m, 6H, H-9). ^{13}C NMR (MeOD, 126 MHz): δ 166.53 (s, C=O, C-4), 152.47 (s, C=O, C-2), 146.84 (s, C-6), 143.92 (s, C-4’’), 124.38 (s, C-5’’), 102.68 (s, C-5), 94.11 (s, C-1’), 84.36 (d, *J* = 7.8 Hz, C-4’), 76.29 (s, C-3’), 71.64 (s, C-2’), 68.03 (d, *J* = 5.8 Hz, C-8), 65.62 (dd, *J* = 6.1, 3.9 Hz, C-5’), 44.89 (s, C-7), 16.38 (d, *J* = 6.5 Hz, C-9). ^{31}P NMR (MeOD, 162 MHz) δ: -1.5. MALDI MS *m/z*: calcd. for $C_{16}H_{24}N_5O_9P$. [M]$^+$ 461.1; found [M]$^+$ 461.2. Anal., %: C 41.61; H 5.25; N 15.19; P 6.73. $C_{16}H_{24}N_5O_9P$. Calcd., %: C 41.65; H 5.24; N 15.18; P 6.71.

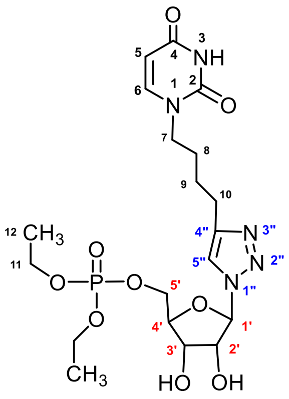

1′-[4′′-(2,4-dioxo-pyrimidine-1-yl-butyl)-1′′,2′′,3′′-triazol-1′′-yl]-β-D-ribofuranose-5′-yl diethyl phosphate (12a).

Light-red foam. Yield 44 %. ^{1}H NMR (MeOD, 600 MHz): δ 7.92 (s, 1H, H-5''), 7.57 (d, *J* = 7.8 Hz, 1H, H-6), 6.01 (d, *J* = 3.0 Hz, 1H, H-1'), 5.64 (d, *J* = 7.8 Hz, 1H, H-5), 4.53 (dd, *J* = 4.8, 3.3 Hz, 1H, H-4'), 4.40 (t, *J* = 5.1 Hz, 1H, H-3'), 4.30 – 4.23 (m, 2H, H-5'), 4.21 – 4.15 (m, 1H, H-2'), 4.12 – 4.03 (m, 4H, H-11), 3.78 (t, *J* = 6.6 Hz, 2H, H-7), 2.76 (t, *J* = 6.9 Hz, 2H, H-10), 1.77 – 1.68 (m, 4H, H-8,9), 1.29 (td, *J* = 6.9, 4.8 Hz, 6H, H-12). ^{13}C NMR(MeOD, 151 MHz): δ 166.70 (s, C-4), 152.79 (s, C-2), 148.79 (s, C-4''), 147.24 (s, C-6), 122.42 (s, C-5''), 102.23 (s, C-5), 94.13 (s, C-1'), 84.23 (d, *J* = 7.5 Hz, C-4'), 76.33 (s, C-3'), 71.66 (s, C-2'), 68.14 (d, *J* = 5.1 Hz, C-5'), 65.59 (d, *J* = 5.3 Hz, C-11), 49.22 (s, C-7), 29.32 (s, C-10), 27.20 (s, C-8), 25.71 (s, C-9), 16.39 (d, *J* = 6.3 Hz, C-12). ^{31}P NMR (MeOD, 162 MHz): δ -1.3. ESI-MS *m/z*: calcd. for $C_{19}H_{30}N_5O_9P$ [M+H]$^+$ 504.2; found [M+H]$^+$ 504.1. Anal., %: C 45.29, H 5.98, N 13.95, P 6.17. $C_{19}H_{30}N_5O_9P$. Calcd., %: C 45.33, H 6.01, N 13.91, P 6.15.

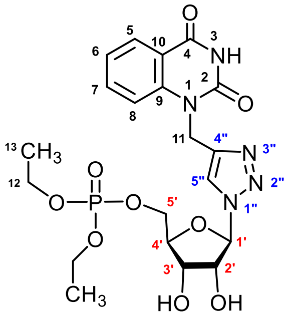

1′-[4″-(2,4-dioxo-quinazoline-1-yl-methyl)-1″,2″,3″-triazol-1″-yl]-β-D-ribofuranose-5′-yl diethyl phosphate (11b).

White foam. Yield 30 %. ^{1}H NMR (MeOD, 400 MHz): δ 8.11 (s, 1H, H-5"), 8.10 (d, *J* = 8.1 Hz, 1H, H-5), 7.71 (t, *J* = 7.2 Hz, 1H, H-7), 7.57 (d, *J* = 8.6 Hz, 1H, H-8), 7.27 (t, *J* = 7.5 Hz, 1H, H-6), 6.00 (d, *J* = 3.2 Hz, 1H, H-1'), 5.43 (s, 2H, H-11), 4.52 (dd, *J* = 4.9, 3.3 Hz, 1H, H-3'), 4.38 (t, *J* = 5.7, 5.2 Hz, 1H, H-2'), 4.25 – 4.23 (m, 1H, H-4'), 4.27 – 4.09 (m, 2H, H-5'), 4.07 – 3.94 (m, 4H, H-12), 1.23 (tdd, *J* = 7.0, 4.5, 1.0 Hz, 6H, H-13). ^{13}C NMR (MeOD, 101 MHz): δ 164.08 (s, C-4), 152.20 (s, C-2), 144.52 (s, C-4"), 142.27 (s, C-9), 136.64 (s, C-7), 129.10 (s, C-5), 124.27 (s, C-6), 124.01 (s, C-5"), 117.36 (s, C-10), 116.08 (s, C-8), 94.13 (s, C-1'), 84.38 (d, *J* = 7.7 Hz, C-4'), 76.29 (s, C-3'), 71.67 (s, C_2'), 68.01 (d, *J* = 5.6 Hz, C-5'), 65.59 (d, *J* = 5.8 Hz, C-12), 39.11 (s, C-11), 16.34 (d, *J* = 6.1 Hz, C-13). ^{31}P NMR (MeOD, 162 MHz): δ -1.5. ESI-MS *m/z*: calcd. for $C_{20}H_{26}N_5O_9P$ $[M+H]^+$ 512.2; found $[M+H]^+$ 512.1. Anal., %: C 46.99, H 5.10, N 13.71, P 6.08. $C_{20}H_{26}N_5O_9P$. Calcd., %: C 46.97, H 5.12, N 13.69, P 6.06.

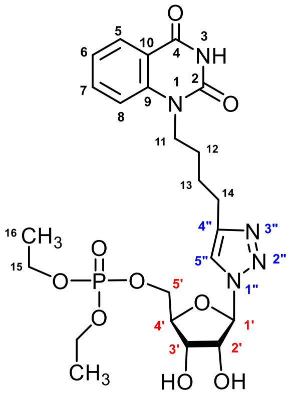

1′-[4″-(2,4-dioxo-quinazoline-1-yl-butyl)-1″,2″,3″-triazol-1″-yl]-β-D-ribofuranose-5′-yl diethyl phosphate (12b).

Transparent viscous oil. Yield 44 %. ^{1}H NMR ($CDCl_3$, 600 MHz): δ 9.96 (s, 1H, NH), 8.15 (d, *J* = 7.5 Hz, 1H, H-5), 7.72 (s, 1H, H-5"), 7.65 (t, *J* = 7.3 Hz, 1H, H-7), 7.23 – 7.17 (m, 2H, H-6,8), 6.07 (s, 1H, H-1'), 4.66 – 4.59 (m, 1H, H-4'), 4.52 (t, *J* = 5.3 Hz, 1H, H-3'), 4.36 – 4.27 (m, 2H, H-5'), 4.24 – 4.16 (m, 1H, H-2'), 4.12 – 4.00 (m, 6H, H-11, H-15), 2.80 – 2.70 (m, 2H, H-14), 1.81 – 1.68 (m, 4H, H-12,13), 1.31 – 1.21 (m, 6H, H-16). ^{13}C NMR($CDCl_3$, 151 MHz): δ 162.27 (s, C-4), 150.74 (s, C-2), 147.25 (s, C-4"), 140.97 (s, C-9), 135.66 (s, C-7), 128.93 (s, C-5), 123.15 (s, C-5"), 120.87 (s, C-6),

116.29 (s, C-10), 114.20 (s, C-8), 92.82 (s, C-1'), 82.95 (d, *J* = 7.3 Hz, C-4'), 75.48 (s, C-3'), 70.70 (s, C-2'), 67.08 (d, *J* = 4.6 Hz, C-5'), 64.41 (d, *J* = 5.4 Hz, C-15), 42.43 (s, C-11), 26.47 (s, C-14), 26.16 (s, C-13), 24.81 (s, C-12), 16.16 (d, *J* = 4.9 Hz, C-16). ^{31}P NMR ($CDCl_3$, 162 MHz): δ -0.8. ESI-MS *m/z*: calcd. for $C_{23}H_{32}N_5O_9P$ [*M*+H]$^+$ 554.2; found [*M*+H]$^+$ 554.1. Anal., %: C 49.93, H 5.81, N 12.63, P 5.57. $C_{20}H_{26}N_5O_9P$. Calcd., %: C 49.91, H 5.83, N 12.65, P 5.60.

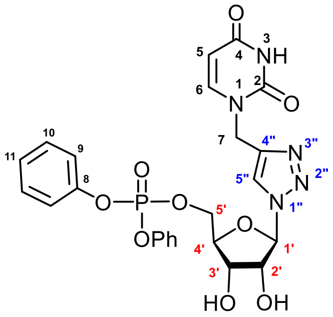

1′-[4″-(2,4-dioxo-pyrimidine-1-yl-methyl)-1″,2″,3″-triazol-1″-yl]-β-D-ribofuranose-5′-yl diphenyl phosphate (13a).

Creamy-colored powder. Yield 23%. ^{1}H NMR (MeOD, 600 MHz): δ 8.09 (s, 1H, H-5”), 7.60 (d, *J* = 7.9 Hz, 1H, H-6), 7.38 – 7.28 (m, 4H, H-10), 7.25 – 7.11 (m, 6H, H-9, H-11), 6.06 (d, *J* = 3.1 Hz, 1H, H-1’), 5.59 (d, *J* = 7.9 Hz, 1H, H-5), 4.94 (s, 2H, H-7), 4.58 – 4.51 (m, 2H, H-2’, H-3’), 4.44 (dd, 2H, H-5’), 4.36 – 4.31 (m, 1H, H-4’). ^{13}C NMR (DMSO-d_6, 151 MHz): δ 166.49 (s, C=O, C-4), 152.46 (s, C=O, C-2), 151.59 (d, *J* = 7.0 Hz, C-8), 146.66 (s, C-6), 143.79 (s, C-4’’), 131.05 (s, C-10), 126.89 (s, C-11), 124.47 (s, C-5’’), 121.13 (d, *J* = 5.0 Hz, C-9), 102.70 (s, C-5), 94.16 (s, C-1’), 84.10 (d, *J* = 7.7 Hz, C-4’), 76.20 (s, C-3’), 71.54 (s, C-2’), 69.73 (d, *J* = 6.5 Hz, C-5’), 43.76 (s, C-7). ^{31}P NMR (MeOD, 162 MHz): δ -12.1. MALDI-MS: calcd. for $C_{24}H_{24}N_5O_9P$ [*M*+H]$^+$ = 558.1; found [*M*+H]$^+$ = 558.3. Anal., %: C 51.73, H 4.37, N 12.54, P 5.55. $C_{24}H_{24}N_5O_9P$. Calcd., %: C 51.71; H 4.34; N 12.56; P 5.56.

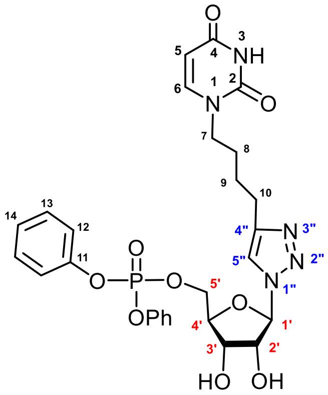

1′-[4″-(2,4-dioxo-pyrimidine-1-yl-butyl)-1″,2″,3″-triazol-1″-yl]-β-D-ribofuranose-5′-yl diphenyl phosphate (14a).

Transparent viscous oil. Yield 42 %. ^{1}H NMR (MeOD, 600 MHz) δ 7.78 (s, 1H, H-5"), 7.44 (d, *J* = 7.8 Hz, 1H, H-6), 7.36 – 7.26 (m, 4H, H-12), 7.22 – 7.15 (m, 2H, H-14), 7.16 – 7.10 (m, 4H, H-13), 6.00 (d, *J* = 3.0 Hz, 1H, H-1'), 5.55 (d, *J* = 7.9 Hz, 1H, H-5), 4.53 (ddd, *J* = 11.1, 6.0, 2.5 Hz, 1H, H-2'), 4.50 – 4.47 (m, 1H, H-3'), 4.44 – 4.36 (m, 2H, H-5'), 4.32 – 4.27 (m, 1H, H-4'), 3.65 (t, *J* = 7.0 Hz, 2H, H-7), 2.61 (t, *J* = 7.1 Hz, 2H, H-10), 1.68 – 1.50 (m, 4H, H-8,9). ^{13}C NMR (MeOD, 151 MHz): δ 166.64 (s, C-4), 152.70 (s, C-2), 151.61 (d, *J* = 7.1 Hz, C-11), 148.76 (s, C-4"), 147.17 (s, C-6), 131.08 (d, *J* = 4.0 Hz, C-13), 126.91 (s, C-14), 122.16 (s, C-5"), 121.14 (d, *J* = 4.0 Hz, C-12), 102.19 (s, C-5), 94.16 (s, C-1'), 83.98 (d, *J* = 7.5 Hz, C-4'), 76.30 (s, C-3'), 71.52 (s, C-2'), 69.76 (d, *J* = 6.0 Hz, C-5'), 49.19 (d, *J* = 4.5 Hz, C-7), 29.24 (s, C-10), 26.98 (s, C8), 25.66 (s, C-9). ^{31}P NMR ($CDCl_3$, 162 MHz) δ -11.6. ESI-MS: calcd. for $C_{27}H_{30}N_5O_9P$ $[M+H]^+$ = 600.2; found $[M+H]^+$ = 600.1. Anal., %: C 54.07, H 5.06, N 11.64, P 5.15. $C_{27}H_{30}N_5O_9P$. Calcd., %: C 54.09; H 5.04; N 11.68; P 5.17.

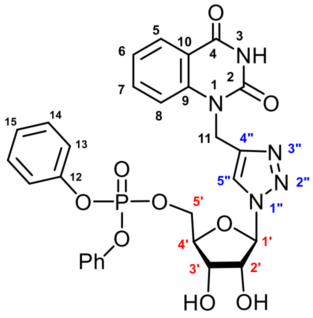

1′-[4″-(2,4-dioxo-quinazoline-1-yl-methyl)-1″,2″,3″-triazol-1″-yl]-β-D-ribofuranose-5′-yl diphenyl phosphate (13b).

White powder. Yield 27 %. ^{1}H NMR (MeOD, 400 MHz): δ 8.09 (s, 1H, H-5"), 8.06 (dd, *J* = 7.9, 1.5 Hz, 1H, H-5), 7.62 (ddd, *J* = 8.7, 7.2, 1.6 Hz, 1H, H-7), 7.53 (d, *J* = 8.4 Hz, 1H, H-8), 7.35 – 7.28 (m, 4H, H-14), 7.27 – 7.08 (m, 7H, H-6,13,15), 6.01 (d, *J* = 3.1 Hz, 1H, H-1'), 5.37 (s, 2H, H-11), 4.51 (dd, *J* = 5.1, 3.0 Hz, 1H, H-2'), 4.49 – 4.46 (m, 1H, H-3'), 4.39 (t, *J* = 5.6 Hz, 2H, H-5'), 4.32 – 4.25 (m, 1H, H-4'). ^{13}C NMR (DMSO-d_6, 101 MHz): δ 162.57 (s, C-4), 150.77 (s, C-2), 150.28 (d, *J* = 7.0 Hz, C-12), 143.09 (s, C-4"), 141.13 (s, C-9), 135.91 (s, C-7), 130.59 (s, C-14), 128.11 (s, C-5), 126.24 (s, C-15), 123.54 (s, C-6), 123.48 (s, C-5"), 120.32 (d, *J* = 4.4 Hz, C-13), 116.10 (s, C-10), 115.44 (s, C-8), 92.37 (s, C-1'), 82.89 (d, *J* = 7.6 Hz, C-4'), 74.76 (s, C-3'), 70.41 (s, C-2'), 69.12 (d, *J* = 5.3 Hz, C-5'), 38.11 (s, C-11). ^{31}P NMR (MeOD, 162 MHz) δ -12.1. ESI-MS: calcd. for $C_{28}H_{26}N_5O_9P$ $[M+H]^+$ = 608.2; found $[M+H]^+$ = 608.1. Anal., %: C 55.35, H 4.36, N 11.54, P 5.08. $C_{28}H_{26}N_5O_9P$. Calcd., %: C 55.36; H 4.31; N 11.53; P 5.10.

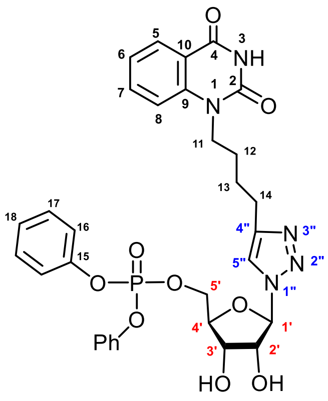

1′-[4″-(2,4-dioxo-quinazoline-1-yl-butyl)-1″,2″,3″-triazol-1″-yl]-β-D-ribofuranose-5′-yl diphenyl phosphate (14b).

Transparent viscous oil. Yield 43 %. ^{1}H NMR (MeOD, 500 MHz): δ 8.00 (dd, *J* = 8.0, 1.3 Hz, 1H, H-5), 7.84 (s, 1H, H-5''), 7.64 – 7.55 (m, 1H, H-7), 7.36 – 7.29 (m, 4H, H-17), 7.19 (t, *J* = 7.5 Hz, 2H, H-18), 7.17 – 7.09 (m, 6H, H-16,8,6), 6.03 (d, *J* = 3.1 Hz, 1H, H-1'), 4.56 (ddd, *J* = 11.3, 6.0, 2.7 Hz, 1H, H-2'), 4.51 (dd, *J* = 4.9, 3.2 Hz, 1H, H-3'), 4.48 – 4.38 (m, 2H, H-5'), 4.36 – 4.29 (m, 1H, H-4'), 3.98 (t, *J* = 6.9 Hz, 2H, H-11), 2.67 (t, *J* = 6.8 Hz, 2H, H-14), 1.70 – 1.59 (m, 4H, H-12,13). ^{13}C NMR (MeOD, 126 MHz): δ 164.24 (s, C-4), 152.32 (s, C-2), 151.61 (d, *J* = 7.3 Hz, C-15), 148.97 (s, C-4''), 140.73 (s, C-9), 136.18 (s, C-7), 131.05 (s, C-17), 128.82 (s, C-5), 126.89 (s, C-18), 124.00 (s, C-6), 122.20 (s, C-5''), 121.13 (d, *J* = 4.4 Hz, C-16), 116.09 (s, C-8), 115.46 (s, C-10), 94.26 (s, C-1'), 83.96 (d, *J* = 7.9 Hz, C-4'), 76.34 (s, C-3'), 71.54 (s, C-2'), 69.80 (d, *J* = 6.0 Hz, C-5'), 41.32 (s, C-11), 28.16 (s, C-14), 27.50 (s, C-12), 25.75 (s, C-13). ^{31}P NMR (MeOD, 162 MHz) δ -11.6. MALDI-MS: calcd. for $C_{31}H_{32}N_5O_9P$ $[M+H]^+$ = 650.2; found $[M+H]^+$ = 650.2. Anal., %: C 57.34, H 4.99, N 10.74, P 4.75. $C_{28}H_{26}N_5O_9P$. Calcd., %: C 57.32; H 4.97; N 10.78; P 4.77.

4. General procedure for the synthesis of 5′-(phenyl methoxy-L-alaninyl)phosphates of 1″,2″,3″-triazolyl nucleoside analogues

1. **Phenyl phosphorodichloridate 5c** was prepared by the reaction of phenol with phosphorus oxychloride according to known procedure [3s]. Yield 60%. Clear viscous oil. ^{31}P NMR ($CDCl_3$, 162 MHz): δ -1.5 ppm.

2. **Methyl (chloro(phenoxy)phosphoryl)-L-alaninate (7c)** was prepared according to the reported procedure [4s] as follows. A solution of triethylamine (4.16 ml, 3.03g, 0.03 mol) in dry dichloromethane (DCM) (70 mL) was added dropwise to a stirred suspension of L-alanine methyl ester hydrochloride and phenyl dichlorophosphate in 120 ml of DCM under argon atmosphere at -70°C during 2 h. Then, the reaction mixture was slowly warmed to the room temperature and stirred for an additional 6 h. A solvent and volatile components were removed *in vacuo*, and the residue dissolved in THF was filtered and evaporated. Yield 3.96 g (95%). ^{31}P NMR ($CDCl_3$, 162 MHz): δ 8.5, 8.6 ppm

(mixture of two diastereomers with an equal ratio). Obtained viscous transparent oil was dissolved in DCM and used as a 20% solution for subsequent reactions.

3. Starting nucleoside **9a,b** or **10a,b** (1 eq) was heated *in vacuo* (60°C, 0.05 Torr) for 30 min, then the flask was filled with argon, and this operation was repeated 2 more times. The dried nucleoside was dissolved in 15 ml of dry DCM, and a flask was equipped with a rubber septum and argon inlet tube and cooled with a water bath (18–20°C). Then 2.2–2.5 eq of the methyl (chloro(phenoxy)phosphoryl)-L-alaninate solution in DCM (see above) was added by the syringe under an argon atmosphere. The flask was tightly closed using a PTFE seal and left to stir for 12-24 hours at room temperature. Upon completion of the reaction (under TLC and ^{31}P NMR control), 1 ml of dry MeOH was added to the resulting mixture and left to stir for another 30 min. Then all volatile components were removed using a water jet pump and dried *in vacuo* (40°C, 0.05 Torr). The residue was purified by flash chromatography to isolate compounds **15a** and **16a,b** (eluent - a mixture of EtOAc : ethanol 10 : 1). Compound **15b** was not isolated and was treated without purification with aqueous trifluoroacetic acid to remove the isopropylidene protection (see step 4).

4. To remove the isopropylidene protection group, 4 ml of trifluoroacetic acid (50% v/v) was added and left to stir for 45 minutes at room temperature. Then, all the volatiles were removed *in vacuo*. The residue was purified by flash chromatography to isolate the target product (eluent - a mixture of EtOAc : ethanol 10 : 1).

4.1 Characterization of compounds

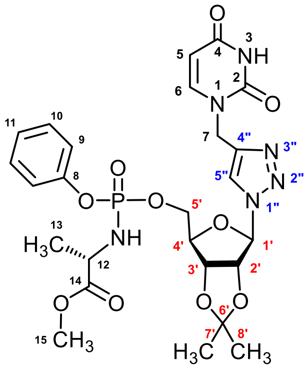

1′-[4″-(2,4-dioxo-pyrimidine-1-yl-methyl)-1″,2″,3″-triazol-1″-yl]-2′,3′-*O*-isopropylidene-β-D-ribofuranose-5′-(phenyl methoxy-L-alaninyl)phosphate (15a).

Transparent viscous oil. Yield 42 %. Mixture of two diastereomers with ratio 60 (A): 40 (B).

^{1}H NMR (MeOD, 400 MHz): δ 8.18 (s, 1H, H-5" (A)), 8.17 (s, 1H, H-5" (B)), 7.70 (d, *J* = 7.9 Hz, 1H, H-6 (B)), 7.69 (d, *J* = 7.9 Hz, 1H, H-6 (A)), 7.37 (d, *J* = 9.3 Hz, 2H, H-9 (B)), 7.35 (d, *J* = 8.3 Hz, 2H, H-9 (A)), 7.22 – 7.16 (m, 3H, H-10,11), 6.27 (d, *J* = 1.7 Hz, 1H, H-1' (A)), 6.25 (d, *J* = 2.0 Hz, 1H, H-1' (B)), 5.66 (d, *J* = 7.9 Hz, 1H, H-5 (B)), 5.66 (d, *J* = 7.9 Hz, 1H, H-5 (A)), 5.40 (dd, *J* = 6.0, 1.7 Hz, 1H, H-2' (A)), 5.26 (dd, *J* = 6.0, 1.9 Hz, 1H, H-2' (B)), 5.04 – 4.96 (m, 3H, H-3',7), 4.58 (t, *J* = 4.8 Hz, 1H, H-4' (B)), 4.53 (td, *J* = 5.5, 2.0 Hz, 1H, H-4' (A)), 4.22 – 4.01 (m, 2H, H-5'), 3.98 – 3.89 (m, 1H,

H-12), 3.69 (s, 3H, H-15 (B)), 3.68 (s, 3H, H-15 (A)), 1.58 (s, 3H, H-7'), 1.39 (s, 3H, H-8' (A)), 1.38 (s, 3H, H-8' (B)), 1.33 (dd, *J* = 7.2, 0.9 Hz, 3H, H-13 (A)), 1.31 (dd, *J* = 7.7, 1.0 Hz, 3H, H-13 (B)). ^{13}C NMR (MeOD, 101 MHz): δ 174.13 (d, *J* = 4.7 Hz, C-14 (B)), 173.96 (d, *J* = 5.1 Hz, C-14 (A)), 165.13 (s, C-4), 151.18 (s, C-2), 150.68 (d, *J* = 6.9 Hz, C-8 (B)), 150.65 (d, *J* = 6.8 Hz, C-8 (A)), 145.42 (s, C-6), 142.76 (s, C-4" (A)), 142.72 (s, C-4" (B)), 129.44 (s, C-10 (B)), 129.42 (s, C-10 (A)), 124.87 (s, C-11 (A)), 124.81 (s, C-11 (B)), 123.69 (s, C-5" (A)), 123.45 (s, C-5" (B)), 120.06 (d, *J* = 4.7 Hz, C-9), 113.77 (s, C-6' (A)), 113.75 (s, C-6' (B)), 101.35 (s, C-5), 94.20 (s, C-1' (B)), 93.83 (s, C-1' (A)), 86.39 – 86.23 (m, C-4' (A)), 86.25 – 86.11 (m, C-4' (B)), 84.53 (s, C-2' (B)), 84.45 (s, C-2' (A)), 81.58 (s, C-3'), 66.00 (d, *J* = 6.1 Hz, C-5' (B)), 65.94 (d, *J* = 5.7 Hz, C-5' (A)), 51.46 (s, C-15), 50.19 (d, *J* = 1.6 Hz, C-12 (B)), 50.04 (d, *J* = 1.2 Hz, C-12 (A)), 42.54 (s, C-7), 25.83 (s, C-7'), 24.01 (s, C-8'), 19.07 (d, *J* = 6.5 Hz, C-13 (A)), 18.94 (d, *J* = 7.1 Hz, C-13 (B)). ^{31}P NMR (MeOD, 243 MHz): δ 3.8, 4.13. MALDI-MS: calcd. for $C_{25}H_{31}N_6O_{10}P$ [*M*+Na]$^+$ = 607.2; found [*M*+Na]$^+$ = 607.4. Anal., %: C 49.54, H 5.19, N 13.84, P 5.08. $C_{25}H_{31}N_6O_{10}P$ Calcd., %: C 49.51; H 5.15; N 13.86; P 5.11.

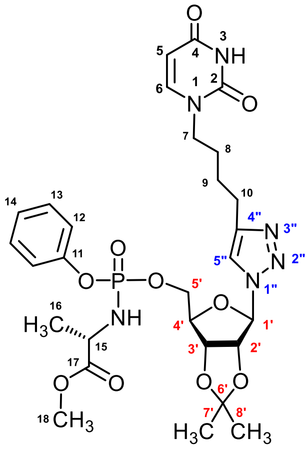

1′-[4″-(2,4-dioxo-pyrimidine-1-yl-butyl)-1″,2″,3″-triazol-1″-yl]-2′,3′-*O*-isopropylidene-β-D-ribofuranose-5′-(phenyl methoxy-L-alaninyl)phosphate (16a).

Light-yellow viscous oil. Yield 31%. A mixture of two diastereomers (A) and (B) with an equal ratio. ^{1}H NMR (MeOD, 500 MHz): δ 7.93 (s, 1H, H-5"), 7.55 (d, *J* = 7.8 Hz, 1H, H-6), 7.39 – 7.34 (m, 2H, H-12), 7.28 – 7.16 (m, 3H, H-13,14), 6.24 (d, *J* = 1.8 Hz, 1H, H-1' (A)), 6.23 (d, *J* = 2.0 Hz, 1H, H-1' (B)), 5.64 (d, *J* = 7.8 Hz, 1H, H-5), 5.39 (dd, *J* = 6.0, 1.8 Hz, 1H, H-2' (A)), 5.32 (dd, *J* = 6.0, 2.0 Hz, 1H, H-2' (B)), 5.01 (t, *J* = 1.9 Hz, 1H, H-3' (A)), 5.00 (t, *J* = 1.9 Hz, 1H, H-3' (B)), 4.56 (td, *J* = 5.3, 2.2 Hz, 1H, H-4' (A)), 4.52 (td, *J* = 5.6, 2.6 Hz, 1H, H-4' (B)), 4.20 – 4.00 (m, 2H, H-5'), 3.97 – 3.88 (m, 1H, H-15), 3.79 – 3.73 (m, 2H, H-7), 3.69 (s, 3H, H-18), 2.74 (td, *J* = 7.0, 4.3 Hz, 2H, H-10), 1.75 – 1.65 (m, 4H, H-8,9), 1.59 (s, 3H, H-7'), 1.41 (s, 3H, H-8' (A)), 1.40 (s, 3H, H-8' (B)), 1.33 (dd, *J* = 7.2, 0.7 Hz, 3H, H-16 (A)), 1.31 (dd, *J* = 7.2, 1.2 Hz, 3H, H-16 (B)). ^{13}C NMR (MeOD, 126 MHz): δ 175.43 (s, C-17 (A)), 175.28 (d, *J* = 5.1 Hz, C-17 (B)), 166.71 (s, C-4), 153.92 (s, C-2 (A)), 152.77 (s,

C-2 (B)), 152.03 (d, *J* = 6.9 Hz, C-11), 149.10 (s, C-4"), 147.23 (s, C-6), 130.80 (s, C-13 (A)), 130.16 (s, C-13 (B)), 126.20 (s, C-14), 124.61 (s, C-5"), 121.47 (d, *J* = 4.9 Hz, C-12 (A)), 121.43 (d, *J* = 4.8 Hz, C-12 (B)), 115.14 (s, C-6'), 102.23 (s, C-5), 95.36 (s, C-1' (A)), 95.10 (s, C-1' (B)), 87.48 (d, *J* = 8.9 Hz, C-4' (A)), 87.45 (d, *J* = 10.9 Hz, C-4' (B)), 85.77 (s, C-2'), 82.98 (s, C-3'), 67.34 (d, *J* = 6.7 Hz, C-5' (A)), 67.29 (d, *J* = 5.9 Hz, C-5' (B)), 52.78 (s, C-18), 51.56 (s, C-15 (A)), 51.44 (s, C-15 (B)), 49.00 (s, C-7 overlapped with the signal of MeOD), 29.30 (s, C-8), 27.21 (s, C-10), 27.09 (s, C-7'), 25.68 (s, C-9), 25.39 (s, C-8'), 20.40 (d, *J* = 6.6 Hz, C-16 (A)), 20.29 (d, *J* = 7.2 Hz, C-16 (B)). ^{31}P NMR (MeOD, 243 MHz): δ 2.5, 2.3. MALDI-MS: calcd. for $C_{28}H_{37}N_6O_{10}P$ [*M*+Na]$^+$ = 671.2; found [*M*+Na]$^+$ = 607.1. Anal., %: C 51.84, H 5.79, N 12.84, P 7.75. $C_{28}H_{26}N_5O_9P$. Calcd., %: C 51.85; H 5.75; N 12.96; P 4.78.

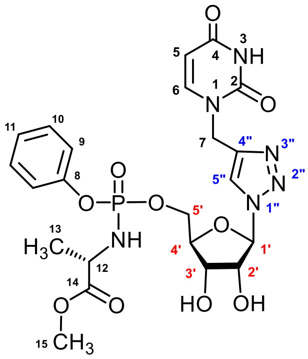

1′-[4″-(2,4-dioxo-pyrimidine-1-yl-methyl)-1″,2″,3″-triazol-1″-yl]-β-D-ribofuranose-5′-(phenyl methoxy-L-alaninyl)phosphate (17a).

White powder. Yield 64 %. Mixture of two diastereomers with ratio 60 (A): 40 (B). ^{1}H NMR (MeOD, 600 MHz): δ 8.21 (s, 1H, H-5" (B)), 8.18 (s, 1H, H-5" (A)), 7.70 (d, *J* = 8.2 Hz, 1H, H-6 (B)), 7.69 (d, *J* = 8.0 Hz, 1H, H-6 (A)), 7.37 – 7.32 (m, 2H, H-9), 7.23 – 7.16 (m, 3H, H-10,11), 6.06 (d, *J* = 3.7 Hz, 1H, H-1'), 5.65 (d, *J* = 7.9 Hz, 1H, H-5 (B)), 5.65 (d, *J* = 7.9 Hz, 1H, H-5 (A)), 5.01 (s, 2H, H-7), 4.54 – 4.50 (m, 1H, H-5'), 4.42 – 4.38 (m, 1H, H-5'), 4.37 – 4.23 (m, 3H, H-2',3',4'), 4.00 – 3.94 (m, 1H, H-12 (A)), 3.93 – 3.88 (m, 1H, H-12 (B)), 3.66 (s, 3H, H-15 (B)), 3.65 (s, 3H, H-15 (A)), 1.32 (d, *J* = 6.9 Hz, 3H, H-13 (A)), 1.29 (d, *J* = 7.2 Hz, 3H, H-13 (B)). ^{13}C NMR (MeOD, 126 MHz): δ 175.50 (d, *J* = 4.4 Hz, C-14 (B)), 175.35 (d, *J* = 5.3 Hz, C-14 (A)), 166.50 (s, C-4), 152.53 (s, C-2), 152.06 (d, *J* = 6.7 Hz, C-8), 146.79 (s, C-6), 143.87 (s, C-4"), 130.79 (s, C-10), 126.21 (s, C-11 (B)), 126.18 – 126.16 (m, C-11 (A)), 124.25 (s, C-5"), 121.48 (d, *J* = 5.0 Hz, C-9 (B)), 121.44 (d, *J* = 4.8 Hz, C-9 (A)), 102.68 (s, C-5), 94.22 (s, C-1'), 84.77 (d, *J* = 8.4 Hz, C-4' (B)), 84.59 (d, *J* = 8.1 Hz, C-4' (A)), 76.52 (s, C-2' (B)), 76.47 (s, C-2' (A)), 71.72 (s, C-3' (A)), 71.66 (s, C-3' (B)), 67.59 (d, *J* = 5.5 Hz, C-5' (A)), 67.27 – 67.11 (m, C-5' (B)), 52.81 (s, C-15), 51.54 (s, C-12 (A)), 51.44 (s, C-12 (B)), 43.87 (s, C_7), 20.47 (d, *J* = 6.2 Hz, C-13 (A)), 20.30 (d, *J* = 7.1 Hz, C-13 (B)). ^{31}P NMR ($CDCl_3$, 243 MHz): δ 3.7, 3.9. MALDI-MS: calcd. for $C_{22}H_{27}O_{10}N_6P$ [*M*+Na]$^+$ = 589.1; found [*M*+Na]$^+$ = 589.1. Anal., %: C 46.65; H 4.79, N 14.81, P 5.45.$C_{22}H_{27}O_{10}N_6P$. Calcd., %: C 46.65; H 4.80; N 14.84; P 5.47.

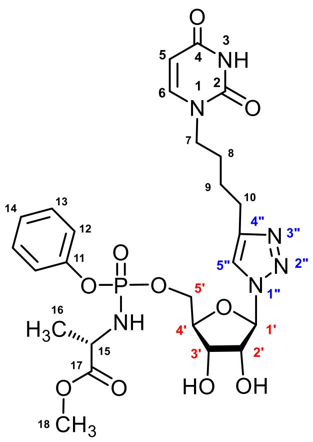

1′-[4″-(2,4-dioxo-pyrimidine-1-yl-butyl)-1″,2″,3″-triazol-1″-yl]-β-D-ribofuranose-5′-(phenyl methoxy-L-alaninyl)phosphate (18a).

White foam. Yield 33 %. Mixture of two diastereomers (A) and (B) with an equal ratio. ^{1}H NMR (MeOD, 400 MHz): δ 7.94 (s, 1H, H-5" (A)), 7.92 (s, 1H, H-5" (A)), 7.54 (d, *J* = 7.8 Hz, 1H, H-6 (A)), 7.54 (d, *J* = 7.8 Hz, 1H, H-6 (B)), 7.38 – 7.33 (m, 2H, H-12), 7.24 – 7.16 (m, 3H, H-13,14), 6.03 (d, *J* = 3.9 Hz, 1H, H1' (A)), 6.03 (d, *J* = 3.7 Hz, 1H, H1' (B)), 5.63 (d, *J* = 7.8 Hz, 1H, H-5 (A)), 5.63 (d, *J* = 7.8 Hz, 1H, H-5 (B)), 4.51 (dd, *J* = 5.0, 3.9 Hz, 1H, H-2' (A)), 4.49 (dd, *J* = 4.9, 3.8 Hz, 1H, H-2' (B)), 4.43 – 4.21 (m, 4H, H-3', H-4’, H-5’), 4.01 – 3.83 (m, 1H, H-15), 3.78 – 3.71 (m, 2H, H-7), 3.67 (s, 3H, H-18 (A)), 3.65 (s, 3H, H-18 (B)), 2.78 – 2.64 (m, 2H, H-10), 1.75 – 1.63 (m, 4H, H-8,9), 1.32 (d, *J* = 7.2 Hz, 3H, H-16 (A)), 1.29 (dd, *J* = 7.2, 0.9 Hz, 3H, H-16 (B)). ^{13}C NMR (MeOD, 101 MHz): δ 175.48 (d, *J* = 4.5 Hz, C-17 (A)), 175.29 (d, *J* = 5.3 Hz, C-17 (B)), 166.71 (s, C-4), 152.76 (s, C-2), 152.07 (d, *J* = 6.2 Hz, C-11), 148.90 (s, C-4"), 147.24 (s, C-6), 130.81 (s, C-13), 126.23 (s, C-14 (A)), 126.21 – 126.18 (m, C-14 (B)), 122.12 (s, C-5" (A)), 122.08 (s, C-5" (B)), 121.48 (d, *J* = 4.5 Hz, C-12 (A)), 121.43 (d, *J* = 4.5 Hz, C-12 (B)), 102.22 (s, C-5), 94.16 (s, C-1'), 84.61 (d, *J* = 8.2 Hz, C-4' (A)), 84.47 (d, *J* = 8.2 Hz, C-4' (B)), 76.55 (s, C-2' (A)), 76.51 (s, C-2' (B)), 71.77 (d, *J* = 2.0 Hz, C-3' (A)), 71.70 (s, C-3' (B)), 67.59 (d, *J* = 5.4 Hz, C-5' (A)), 67.28 (d, *J* = 5.4 Hz, C-5' (B)), 52.78 (s, C-18 (A)), 52.76 (s, C-18 (B)), 51.55 (s, C-15 (A)), 51.45 (s, C-15 (B)), 48.79 (s, C-7 overlapped with the signal of MeOD), 29.32 (s, C-8 (A)), 29.29 (s, C-8 (B)), 27.17 (s, C-10 (A)), 27.14 (s, C-10 (B)), 25.74 (s, C-9 (A)), 25.71 (s, C-9 (B)), 20.44 (d, *J* = 6.5 Hz, C-16 (A)), 20.32 (d, *J* = 7.2 Hz, C-16 (B)). ^{31}P NMR ($CDCl_3$, 243 MHz): δ 2.3, 2.2. MALDI-MS: calcd. for $C_{25}H_{33}O_{10}N_6P$ $[M+H]^+$ = 609.2; found $[M+H]^+$ = 609.2. Anal., %: C 49.31; H 5.49, N 13.79, P 5.08. $C_{25}H_{33}O_{10}N_6P$. Calcd., %: C 49.34; H 5.47; N 13.81; P 5.09.

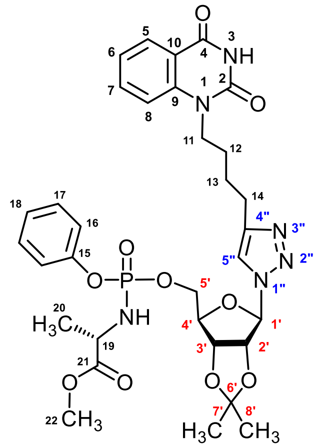

1′-[4″-(2,4-dioxo-quinazoline-1-yl-butyl)-1″,2″,3″-triazol-1″-yl]-2′,3′-*O*-isopropylidene-β-D-ribofuranose-5′-(phenyl methoxy-L-alaninyl)phosphate (16b).

Light-yellow viscous oil. Yield 67 %. Mixture of two diastereomers with ratio 60 (A): 40 (B). ^{1}H NMR ($CDCl_3$, 500 MHz): δ 8.23 (dd, *J* = 7.8, 1.6 Hz, 1H, H-5 (A)), 8.22 (dd, *J* = 7.8, 1.6 Hz, 1H, H-5 (B)), 7.73 – 7.68 (m, 1H, H-7), 7.62 (s, 1H, H-5"), 7.34 – 7.29 (m, 2H, H-6,8), 7.28 – 7.19 (m, 4H, H-16,17), 7.18 – 7.13 (m, 1H, H-18), 6.14 (d, *J* = 1.5 Hz, 1H, H-1' (A)), 6.12 (d, *J* = 2.0 Hz, 1H, H-1' (B)), 5.44 (dd, *J* = 6.0, 1.6 Hz, 1H, H2' (B)), 5.25 (dd, *J* = 6.0, 2.0 Hz, 1H, H2' (A)), 4.92 (dd, *J* = 5.9, 2.2 Hz, 1H, H-3' (B)), 4.90 (dd, *J* = 5.8, 2.1 Hz, 1H, H-3' (A)), 4.58 – 4.51 (m, 1H, H4'), 4.30 – 3.98 (m, 5H, H-5', H-11, H-19), 3.73 (s, 3H, H-22 (B)), 3.72 (s, 3H, H-22 (A)), 2.93 – 2.73 (m, 2H, H-14), 1.89 – 1.70 (m, 4H, H-12,13), 1.60 (s, 3H, H-7'), 1.40 (d, *J* = 6.3 Hz, 3H, H-20 (A)), 1.40 (s, 3H, H-8' (A)), 1.38 (s, 3H, H-8' (B)), 1.34 (d, *J* = 7.0 Hz, 3H, H-20 (B)). ^{13}C NMR ($CDCl_3$, 126 MHz): δ 174.47 (s, C-21 (A)), 174.24 (s, C-21 (B)), 161.85 (s, C-4), 150.76 (d, *J* = 6.2 Hz, C-15), 150.58 (s, C-2 (A)), 150.41 (s, C-2 (B)), 148.14 (s, C-4" (A)), 148.05 (s, C-4" (B)), 141.03 (s, C-9), 135.66 (s, C-7), 129.82 (s, C-17), 129.08 (s, C-5), 125.18 (s, C-18 (A)), 125.10 (s, C-18 B)), 123.20 (s, C-6 (A)), 123.14 (s, C-6 (B)), 120.79 (s, C-5" (B)), 120.57 (s, C-5" (A)), 120.39 (d, *J* = 4.3 Hz, C-16 (A)), 120.30 (d, *J* = 4.4 Hz, C-16 (B)), 116.37 (s, C-10), 114.17 (s, C-8), 114.20 (s, C-6'), 94.24 (s, C-1' (A)), 93.79 (s, C-1' (B)), 86.34 (d, *J* = 8.2 Hz, C-4' (A)), 86.01 (d, *J* = 7.9 Hz, C-4' (B)), 84.69 (s, C-2' (A)), 84.54 (s, C-2' (B)), 81.77 (s, C-3' (A)), 81.62 (s, C-3' (B)), 66.01 (s, C-5'), 52.68 (s, C-22), 50.46 (s, C-19 (A)), 50.33 (s, C-19 (B)), 42.39 (s, C-11), 27.09 (s, C-7' (A)), 27.03 (s, C-7' (B)), 26.53 (s, C-12 (B)), 26.41 (s, C-12 (A)), 26.41 (s, C-14 (A)), 26.31 (s, C-14 (B)), 25.33 (s, C-8'), 24.95 (s, C-13 (A)), 24.87 (s, C-13 (B)), 21.04 (d, *J* = 5.0 Hz, C-20). ^{31}P NMR ($CDCl_3$, 243 MHz): δ 2.7, 2.4. MALDI-MS: calcd. for $C_{32}H_{39}O_{10}N_6P$ $[M+H]^+$ = 698.3; found $[M+H]^+$ = 699.2. Anal., %: C 54.99; H 5.65, N 12.05, P 4.40. $C_{32}H_{39}O_{10}N_6P$. Calcd., %: C 55.01; H 5.63; N 12.03; P 4.43.

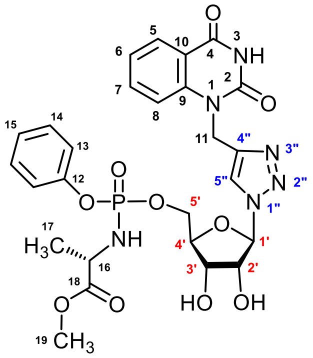

1′-[4″-(2,4-dioxo-quinazoline-1-yl-methyl)-1″,2″,3″-triazol-1″-yl]-β-D-ribofuranose-5′-(phenyl methoxy-L-alaninyl)phosphate (17b).

White powder. Yield 35 %. Mixture of two diastereomers with ratio 70 (A): 30 (B). ^{1}H NMR (MeOD, 400 MHz): δ 8.18 (s, 1H, H-5" (A)), 8.16 (s, 1H, H-5" (B)), 8.09 (d, *J* = 7.9 Hz, 1H, H-5 (B)), 8.08 (d, *J* = 7.9 Hz, 1H, H-5 (A)), 7.73 – 7.65 (m, 1H, H-6), 7.62 – 7.56 (m, 1H, H-8), 7.35 – 7.23 (m, 3H, H-7, 13), 7.20 – 7.11 (m, 3H, H-14,15), 6.02 (d, *J* = 4.0 Hz, 1H, H-1' (B)), 6.01 (d, *J* = 4.0 Hz, 1H, H-1' (A)), 5.44 (s, 2H, H-11 (A)), 5.41 (s, 2H, H-11 (B)), 4.49 (dd, *J* = 5.0, 4.0 Hz, 1H, H-2' (A)), 4.48 (dd, *J* = 5.0, 3.7 Hz, 1H, H-2'(B)), 4.39 – 4.34 (m, 2H, H-3', H-4'), 4.33 – 4.18 (m, 2H, H-5'), 3.98 – 3.84 (m, 1H, H-16), 3.64 (s, 3H, H-19 (A)), 3.60 (s, 3H, H-19 (B)), 1.30 (dd, *J* = 7.1, 0.8 Hz, 3H, H-17 (B)), 1.26 (dd, *J* = 7.2, 1.1 Hz, 3H, H-17 (A)). ^{13}C NMR (MeOD, 101 MHz): δ 175.39 (s, C-18 (B)), 175.32 (s, C-18 (A)), 164.07 (s, C-4), 152.27 (s, C-2), 152.03 (d, *J* = 6.9 Hz, C-12), 144.45 (s, C-4"), 142.28 (s, C-9), 136.64 (s, C-7), 130.73 (s, C-14), 129.08 (s, C-5), 126.12 (s, C-15), 124.26 (s, C-6), 123.97 (s, C-5"), 121.42 (d, *J* = 4.7 Hz, C-13), 117.35 (s, C-10), 116.10 (s, C-8), 94.19 (s, C-1'), 84.54 (d, *J* = 8.1 Hz, C-4'), 76.41 (s, C-3'), 71.70 (s, C-2'), 67.61 (d, *J* = 5.4 Hz, C-5'), 52.77 (s, C-19), 51.41 (s, C-16), 39.13 (s, C-11), 20.46 (d, *J* = 6.3 Hz, C-17). ^{31}P NMR (MeOD, 243 MHz): δ 3.8 (A), 3.6 (B). MALDI-MS: calcd. for $C_{26}H_{29}O_{10}N_6P$ $[M+H]^+$ = 617.2; found $[M+H]^+$ = 617.1. Anal., %: C 50.62; H 4.76, N 13.60, P 5.01. $C_{32}H_{39}O_{10}N_6P$. Calcd., %: C 50.65; H 4.74, N 13.63, P 5.02.

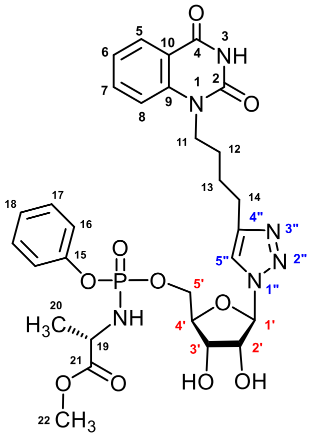

1′-[4″-(2,4-dioxo-quinazoline-1-yl-butyl)-1″,2″,3″-triazol-1″-yl]-β-D-ribofuranose-5′-(phenyl methoxy-L-alaninyl)phosphate (18b).

Cream-colored powder. Yield 52 %. Mixture of two diastereomers with an equal ratio. ^{1}H NMR (MeOD, 400 MHz): δ 8.11 (dd, *J* = 7.9, 1.5 Hz, 1H, H-5), 7.95 (s, 1H, H-5'' (A)), 7.93 (s, 1H, H-5'' (B)), 7.81 – 7.68 (m, 1H, H-7), 7.41 – 7.25 (m, 4H, H-6,8,16), 7.25 – 7.12 (m, 3H, H-17,18), 6.03 (d, *J* = 4.0 Hz, 1H, H-1' (A)), 6.02 (d, *J* = 3.8 Hz, 1H, H-1' (B)), 4.49 (ddd, *J* = 9.9, 5.0, 3.8 Hz, 1H, H-2'), 4.42 – 4.20 (m, 4H, H-3',4',5'), 4.12 (t, *J* = 7.1 Hz, 1H, H-11), 4.01 – 3.83 (m, 1H, H-19), 3.65 (s, 3H, H-22 (A)), 3.63 (s, 1H, H-22 (B)), 2.76 (dt, *J* = 10.6, 7.0 Hz, 1H, H-14), 1.94 – 1.61 (m, 1H, H-12,13), 1.31 (dd, *J* = 7.2, 1.0 Hz, 3H, H-20 (A)), 1.27 (dd, *J* = 7.2, 1.2 Hz, 3H, H-20 (B)). ^{13}C NMR (MeOD, 101 MHz): δ 175.47 (d, *J* = 4.2 Hz, C-21 (A)), 175.28 (d, *J* = 5.1 Hz, C-21 (B)), 164.14 (s, C-4), 152.15 (s, C-2), 152.06 (d, *J* = 6.6 Hz, C-15), 148.92 (s, C-4'' (A)), 148.88 (s, C-4'' (B)), 142.41 (s, C-9), 136.80 (s, C-7), 130.78 (s, C-17), 129.16 (s, C-5), 126.17 (s, C-6), 124.01 (s, C-18), 122.06 (s, C-5''), 121.46 (d, *J* = 4.3 Hz, C-16 (A)), 121.42 (d, *J* = 4.1 Hz, C-16 (B)), 117.27 (s, C-10), 115.75 (s, C-8), 94.21 (s, C-1'), 84.60 (d, *J* = 8.5 Hz, C-4' (A)), 84.45 (d, *J* = 8.2 Hz, C-4' (B)), 76.59 (s, C-3' (A)), 76.54 (s, C-3' (B)), 71.75 (s, C-2' (A)), 71.69 (s, C-2' (B)), 67.57 (d, *J* = 5.1 Hz, C-5' (A)), 67.25 (d, *J* = 5.4 Hz, C-5' (B)), 52.75 (s, C-22), 51.55 (s, C-19 (A)), 51.44 (s, C-19 (B)), 43.25 (s, C-11), 27.63 (s, C-12), 27.33 (s, C-14), 25.77 (s, C-13), 20.44 (d, *J* = 6.3 Hz, C-20 (A)), 20.31 (d, *J* = 7.2 Hz, C-20 (B)). ^{31}P NMR (MeOD, 243 MHz): δ 3.8 (A), 3.6 (B). ESI-MS: calcd. for $C_{29}H_{35}O_{10}N_6P$ $[M+H]^+$ = 659.2; found $[M+H]^+$ = 659.2. Anal., %: C 52.86; H 5.39; N 12.73; P 4.68. $C_{29}H_{35}O_{10}N_6P$. Calcd., %: C 52.89; H 5.36; N 12.76; P 4.70.

5. General procedure for the synthesis of 5′-*H*-phosponates of 1′′,2′′,3′′-triazolyl nucleoside analogues

2-Chloro-4*H*-benzo[*d*][1,3,2]dioxaphosphinin-4-one 9c was prepared as described [5s]. Yield 60 %. B.p. 135-137°C (15 torr). White crystalline solid. ^{31}P NMR ($CDCl_3$, 162 MHz): δ 149.7 ppm.

Starting nucleoside **9a,b** or **10a,b** (1eq) was heated *in vacuo* (60°C, 0.05 Torr) for 30 min, then the flask was filled with argon, and this operation was repeated 2 more times. The dried nucleoside was dissolved in dry DCM (10 ml per 1 mmol of nucleoside) and 10 eq of pyridine was added. A flask was equipped with a rubber septum, and argon inlet tube and cooled with a water bath (18–20°C). Then 2.2–2.5 eq of the 2-chloro-4*H*-benzo[*d*][1,3,2]dioxaphosphinin-4-one 20 % solution in DCM was added by the syringe under argon atmosphere. The flask was stirred for 30 min at room temperature and then 0.5 ml of distilled water and 0.5 ml of Et_3N were added in one portion and stirred for an additional 30 min. Then all volatile components were removed on a rotary evaporator and dried *in vacuo* (40°C, 0.05 Torr). The residue was purified by flash chromatography to isolate the target product (gradient elution from CH_2Cl_2:MeOH+0.5% Et_3N 25:1 to pure MeOH).

To remove the isopropylidene protection group, 4 ml of trifluoroacetic acid (50% v/v) was added and left to stir for 45 min at room temperature. Then, all the volatiles were removed *in vacuo*. The residue

was purified by flash chromatography to isolate the target compounds **23-24a,b** (eluent - a mixture of $CHCl_3$/MeOH + 0.5% Et_3N from 30:1 to pure MeOH).

5.1 Characterization of compounds

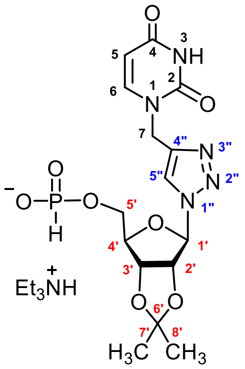

1′-[4″-(2,4-dioxo-pyrimidine-1-yl-methyl)-1″,2″,3″-triazol-1″-yl]-2′,3′-*O*-isopropylidene-β-D-ribofuranose-5′-yl *H*-phosphonate triethylammonium salt (21a).

White powder. Yield 81 %. ^{1}H NMR ($CDCl_3$, 400 MHz): δ 8.17 (s, 1H, H-5’’), 7.50 (d, J = 7.9 Hz, 1H, H-6), 6.73 (d, J = 622.2 Hz, 1H, PH), 6.17 (д, J = 2.2 Hz, 1H, H-1’), 5.65 (d, J = 7.9 Hz, 1H, H-5), 5.29 (dd, J = 5.9, 2.2 Hz, 1H, H-2’), 5.01 – 4.97 (m, 3H, H-3’, H-7), 4.55 – 4.49 (m, 1H, H-4’), 3.93 – 3.81 (m, 2H, H-5’), 1.57 (s, 3H, H-7’), 1.35 (s, 3H, H-8’). ^{13}C NMR ($CDCl_3$, 101 MHz): δ 164.03 (s, C=O, C-4), 151.13 (s, C=O, C-2), 144.39 (s, C-6), 142.42 (s, C-4’’), 123.11 (s, C-5’’), 113.89 (s, C-6’), 102.68 (s, C-5), 95.02 (s, C-1’), 86.84 (d, J = 7.7 Hz, C-4’), 85.05 (s, C-2’), 82.02 (s, C-3’), 63.26 (d, J = 4.4 Hz, C-5’), 45.62 (s, CH_2), 42.88 (s, C-7), 27.09 (s, C-7’), 25.30 (s, C-8’), 8.71 (s, CH_3). ^{31}P NMR ($CDCl_3$, 162 MHz): δ 4.7. ESI-MS: calcd. for $C_{15}H_{19}N_5O_8P^-$ [M+H]$^+$ = 429.1; found [M+H]$^+$ = 429.2. Anal., %: C 47.50; H 6.69; N 15.87; P 5.79. $C_{29}H_{35}O_{10}N_6P$. Calcd., %: C 47.54; H 6.65; N 15.84; P 5.84.

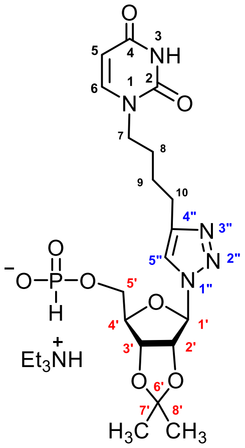

1′-[4″-(2,4-dioxo-pyrimidine-1-yl-butyl)-1″,2″,3″-triazol-1″-yl]-2′,3′-*O*-isopropylidene-β-D-ribofuranose-5′-yl *H*-phosphonate triethylammonium salt (22a).

Light-yellow powder. Yield 86 %. ^{1}H NMR ($CDCl_3$, 600 MHz): δ 7.78 (s, 1H, H-5''), 7.19 (d, *J* = 7.8 Hz, 1H, H-6), 6.79 (d, *J* = 622.4 Hz, 1H, PH), 6.12 (s, 1H, H-1'), 5.59 (d, *J* = 7.8 Hz, 1H, H-5), 5.23 (dd, *J* = 6.0, 2.2 Hz, 1H, H-3'), 4.96 (dd, *J* = 6.1, 1.5 Hz, 1H, H-2'), 4.48 – 4.44 (m, 1H, H-4'), 3.93 – 3.79 (m, 2H, H-5'), 3.71 (dd, *J* = 11.9, 6.1 Hz, 2H, H-10), 3.00 (q, *J* = 7.3 Hz, 6H, CH_2), 2.76 – 2.70 (m, 2H, H-7), 1.70 – 1.65 (m, 4H, H-8,9), 1.54 (s, 3H, H7'), 1.32 (s, 3H, H8'), 1.25 (t, *J* = 7.2 Hz, 9H, CH_3). ^{13}C NMR ($CDCl_3$, 151 MHz): δ 164.30 (s, C-4), 151.28 (s, C-2), 147.88 (s, C-4''), 144.56 (s, C-6), 120.34 (s, C-5''), 113.85 (s, C-6'), 102.22 (s, C-5), 94.76 (s, C-1'), 86.27 (d, *J* = 7.9 Hz, C-4'), 85.03 (s, C-2'), 82.08 (s, C-3'), 63.16 (d, *J* = 4.4 Hz, C-5'), 48.10 (s, C-7), 45.74 (s, CH_2), 27.96 (s, C-8), 27.19 (s, C-7'), 25.87 (s, C-9), 25.38 (s, C-10), 24.56 (s, C-8'), 9.11 (s, CH_3). ^{31}P NMR ($CDCl_3$, 162 MHz): δ 4.7. MALDI-MS: calcd. for $C_{18}H_{26}N_5O_8P$ $[M{+}H]^+$ = 472.1; found $[M{+}H]^+$ = 472.2. Anal., %: C 50.37; H 7.26; N 14.65; P 5.38. $C_{24}H_{41}O_8N_6P$. Calcd., %: C 50.34; H 7.22; N 14.68; P 5.41.

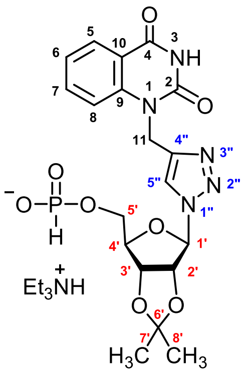

1′-[4′′-(2,4-dioxo-quinazoline-1-yl-methyl)-1′′,2′′,3′′-triazol-1′′-yl]-2′,3′-*O*-isopropylidene-β-D-ribofuranose-5′-yl *H*-phosphonate triethylammonium salt (21b).

White powder. Yield 87 %. ^{1}H NMR ($CDCl_3$, 400 MHz): δ 8.11 (d, *J* = 7.8 Hz, 1H, H-5), 8.06 (s, 1H, H-5''), 7.68 – 7.63 (m, 1H, H-7,8), 7.20 (t, *J* = 8.0 Hz, 1H, H-6), 6.67 (d, *J* = 621.5 Hz, 1H, PH), 6.10 (d, *J* = 2.1 Hz, 1H, H-1’), 5.36 (m, 3H, H-3',11), 4.98 (dd, *J* = 6.0, 1.7 Hz, 1H, H-2'), 4.49 (t, *J* = 4.1 Hz, 1H, H-4'), 3.89 – 3.74 (m, 2H, H-5'), 2.84 (q, *J* = 7.4 Hz, 6H, CH_2), 1.53 (s, 3H, H-7'), 1.31 (s, 3H, H-8'), 1.18 (t, *J* = 7.3 Hz, 9H, CH_3). ^{13}C NMR ($CDCl_3$, 126 MHz): δ 162.28 (s, C4), 150.81 (s, C-2), 143.31 (s, C-4''), 140.91 (s, C-9), 135.59 (s, C-7), 128.45 (s, C-5), 123.19 (s, C-6), 122.86 (s, C-5''), 116.33 (s, C-10), 115.03 (s, C-8), 113.82 (s, C-6'), 94.86 (s, C-1'), 86.75 (d, *J* = 7.6 Hz, C-4'), 84.91 (s, C-3'), 82.05 (s, C-2'), 63.16 (d, *J* = 3.9 Hz, C-5'), 45.65 (s, CH_2), 38.53 (s, C-11), 27.07 (s, C-7'), 25.25 (s, C-8'), 8.62 (s, CH_3). ^{31}P NMR ($CDCl_3$, 162 MHz): δ 4.2. ESI-MS: calcd. for $C_{19}H_{21}N_5O_8P$ $[M{-}H]^-$ = 478.1; found $[M{-}H]^-$ = 478.0. Anal., %: C 51.80; H 6.45; N 14.52; P 5.29. $C_{25}H_{37}O_8N_6P$. Calcd., %: C 51.82; H 6.42; N 14.48; P 5.33.

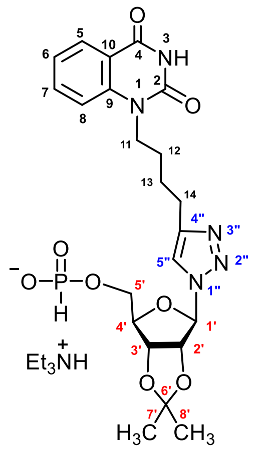

1′-[4″-(2,4-dioxo-quinazoline-1-yl-butyl)-1″,2″,3″-triazol-1″-yl]-2′,3′-*O*-isopropylidene-β-D-ribofuranose-5′-yl *H*-phosphonate triethylammonium salt (22b).

White foam. Yield 87 %. ^{1}H NMR ($CDCl_3$, 400 MHz): δ 8.17 (dd, *J* = 8.0, 1.7 Hz, 1H, H-5), 7.95 (s, 1H, H-5''), 7.71 – 7.65 (m, 1H, H-7), 7.25 – 7.20 (m, 2H, H-6,8), 6.95 (d, *J* = 617.3 Hz, 1H, PH), 6.20 (d, *J* = 2.8 Hz, 1H, H-1'), 5.16 (dd, *J* = 6.0, 2.8 Hz, 1H, H-2'), 5.03 (dd, *J* = 6.0, 1.9 Hz, 1H, H-3'), 4.53 (ddd, *J* = 6.0, 4.2, 2.0 Hz, 1H, H-4'), 4.21 – 4.14 (m, 2H, H-11), 4.06 – 3.91 (m, 2H, H-5'), 3.03 (q, *J* = 7.2 Hz, 6H, CH_2), 2.87 (t, *J* = 7.4 Hz, 2H, H-14), 1.84 – 1.74 (m, 4H, H-12,13), 1.60 (s, 3H, H-7'), 1.36 (s, 3H, H-8'), 1.32 (t, *J* = 7.3 Hz, 9H, CH_3). ^{13}C NMR ($CDCl_3$, 126 MHz): δ 162.41 (s, C4), 150.85 (s, C-2), 148.11 (s, C-4''), 141.09 (s, C-9), 135.36 (s, C-7), 128.75 (s, C-5), 122.74 (s, C-6), 120.30 (s, C-5''), 116.48 (s, C-10), 114.07 (s, C-8), 113.88 (s, C-6'), 94.82 (s, C-1'), 86.08 (d, *J* = 8.0 Hz, C-4'), 85.12 (s, 2'), 82.02 (s, C-3'), 63.20 (d, *J* = 4.1 Hz, C-5'), 45.75 (s, CH_2), 41.79 (s, C-11), 27.22 (s, C-12), 26.27 (s, C-13), 26.12 (s, C-7'), 25.38 (s, C-14), 24.62 (s, C-8'), 8.68 (s, CH_3). ^{31}P NMR ($CDCl_3$, 162 MHz): δ 4.2. MALDI-MS: calcd. for $C_{22}H_{28}N_5O_8P$ [M+H]$^+$ = 522.2; found [M-H]$^-$ = 522.2. Anal., %: C 54.05; H 6.98; N 13.54; P 4.96. $C_{28}H_{43}O_8N_6P$. Calcd., %: C 54.01; H 6.96; N 13.50; P 4.97.

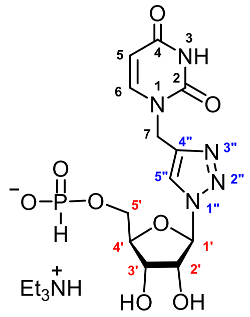

1′-[4″-(2,4-dioxo-pyrimidine-1-yl-methyl)-1″,2″,3″-triazol-1″-yl]-β-D-ribofuranose-5′-yl *H*-phosphonate triethylammonium salt (23a).

White powder. Yield 50 %. ^{1}H NMR (MeOD, 400 MHz): δ 8.33 (s, 1H, H-5’’), 7.72 (d, *J* = 7.9 Hz, 1H, H-6), 6.77 (d, J = 621 Hz, 1H, PH), 6.05 (d, *J* = 4.6 Hz, 1H, H-1’), 5.67 (d, *J* = 7.9 Hz, 1H, H-5), 5.06 (s, 2H, H-7), 4.53 (t, *J* = 4.8 Hz, 1H, H-3’), 4.33 (t, *J* = 4.7 Hz, 1H, H-2’), 4.25 – 4.20 (m, 1H, H-4’), 4.11 – 3.97 (m, 2H, H-5’), 3.16 (q, *J* = 7.3 Hz, 6H, CH_2), 1.29 (t, *J* = 7.3 Hz, 9H, CH_3). ^{13}C NMR

(MeOD, 151 MHz): δ 166.58 (s, C=O, C-4), 152.54 (s, C=O, C-2), 146.87 (s, C-6), 144.05 (s, C-4”), 123.68 (s, C-5”), 102.66 (d, *J* = 10.3 Hz, C-5), 94.47 (s, C-1’), 85.87 (s, C-4’), 76.91 (s, C-2’), 72.14 (s, C-3’), 64.31 (s, C-5’), 47.73 (s, CH_2), 43.60 (s, C-7), 9.25 (s, CH_3),^{31}P NMR (MeOD, 162 MHz): δ 4.6. MALDI-MS: calcd. for $C_{12}H_{15}O_8N_5P$ [*M*-H]$^-$ = 388.1; found [*M*-H]$^-$ = 388.0. Anal., %: C 44.11; H 6.39; N 17.11; P 6.28. $C_{18}H_{31}O_8N_6P$. Calcd., %: C 44.08; H 6.37; N 17.14; P 6.32.

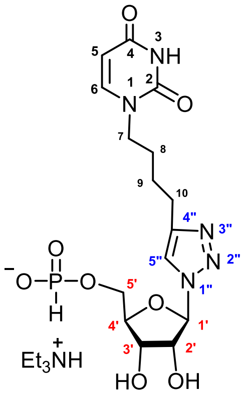

1′-[4″-(2,4-dioxo-pyrimidine-1-yl-butyl)-1″,2″,3″-triazol-1″-yl]-β-D-ribofuranose-5′-yl *H*-phosphonate triethylammonium salt (24a).

White powder. Yield 80 %. ^{1}H NMR (MeOD, 600 MHz): δ 8.10 (s, 1H, H-5''), 7.60 (d, *J* = 7.8 Hz, 1H, H-6), 6.24 (d, *J* = 620.2 Hz, 1H, PH), 6.02 (d, *J* = 4.8 Hz, 1H, H-1'), 5.64 (d, *J* = 7.8 Hz, 1H, H-5), 4.51 (t, *J* = 4.9 Hz, 1H, H-2'), 4.33 (t, *J* = 4.6 Hz, 1H, H-3'), 4.23 – 4.21 (m, 1H, H-4'), 4.12 – 3.94 (m, 2H, H-5'), 3.80 – 3.77 (m, 2H, H-7), 3.08 (q, *J* = 7.3 Hz, 6H, CH_2), 2.85 – 2.71 (m, 2H, H-10), 1.73 (t, *J* = 3.5 Hz, 4H, H-8,9), 1.26 (t, *J* = 7.3 Hz, 9H, CH_3). ^{13}C NMR (MeOD, 151 MHz): δ 166.75 (s, C-4), 152.76 (s, C-2), 149.03 (s, C-4''), 147.39 (d, *J* = 4.7 Hz, C-6), 121.65 (s, C-5''), 102.15 (d, *J* = 6.8 Hz, C-5), 94.32 (d, *J* = 4.3 Hz, C-1'), 85.79 (s, C-4'), 76.92 (s, C-3'), 72.26 (s, C-2'), 64.41 (d, *J* = 4.4 Hz, C-5'), 49.00 (s, C-7, overlapped with the signal of MeOD), 47.65 (s, CH_2), 29.35 (s, C-8), 27.24 (s, C-9), 25.76 (s, C-10), 9.54 (s, CH_3). ^{31}P NMR (MeOD, 162 MHz): δ 4.6. ESI-MS: calcd. for $C_{15}H_{21}O_8N_5P$ [*M*-H]$^-$ = 430.1; found [*M*-H]$^-$ = 430.0. Anal., %: C 47.33; H 7.01; N 15.75; P 5.79. $C_{18}H_{31}O_8N_6P$. Calcd., %: C 47.36; H 7.00; N 15.78; P 5.82.

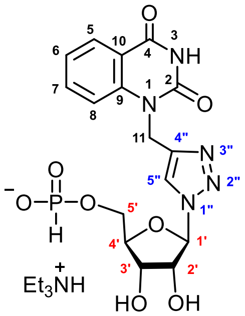

1′-[4″-(2,4-dioxo-quinazoline-1-yl-methyl)-1″,2″,3″-triazol-1″-yl]-β-D-ribofuranose-5′-yl *H*-phosphonate triethylammonium salt (23b).

White powder. Yield 65 %. ^{1}H NMR (MeOD, 400 MHz): δ 8.34 (s, 1H, H-5''), 8.12 (dd, *J* = 7.9, 1.6 Hz, 1H, H-5), 7.74 (ddd, *J* = 8.8, 7.2, 1.7 Hz, 1H, H-7), 7.56 (d, *J* = 8.5 Hz, 1H, H-8), 7.33 – 7.22 (m, 1H, H-6), 6.76 (d, *J* = 621.7 Hz, 1H, PH), 6.03 (d, *J* = 4.7 Hz, 1H, H-1'), 5.49 (m, 2H, H-11), 4.53 (t, *J* = 4.8 Hz, 1H, H-2'), 4.34 (t, *J* = 4.7 Hz, 1H, H-3'), 4.28 – 4.20 (m, 1H, H-4'), 4.14 – 3.95 (m, 2H, H-5'), 3.15 (q, *J* = 7.3 Hz, 6H, CH_2), 1.29 (t, *J* = 7.3 Hz, 9H, CH_3). ^{13}C NMR (MeOD, 101 MHz): δ 164.19 (s, C-4), 152.28 (s, C-2), 144.45 (s, C-4''), 142.34 (s, C-9), 136.73 (s, C-7), 129.03 (s, C-5), 124.20 (s, C-6), 123.21 (s, C-5''), 117.37 (s, C-10), 116.20 (s, C-8), 94.37 (s, C-1'), 85.87 (d, *J* = 7.7 Hz, C-4'), 76.84 (s, C-3'), 72.18 (s, C-2'), 64.30 (d, *J* = 4.5 Hz, C-5'), 47.73 (s, CH_2), 38.99 (s, C_{11}), 9.33 (s, CH_3). ^{31}P NMR (MeOD, 162 MHz): δ 4.6. ESI-MS: calcd. for $C_{16}H_{17}O_8N_5P$ [*M*-H]$^-$ = 438.1; found [*M*-H]$^-$ = 438.0. Anal., %: C 48.90; H 6.17; N 15.56; P 5.71. $C_{18}H_{31}O_8N_6P$. Calcd., %: C 48.89; H 6.15; N 15.55; P 5.73.

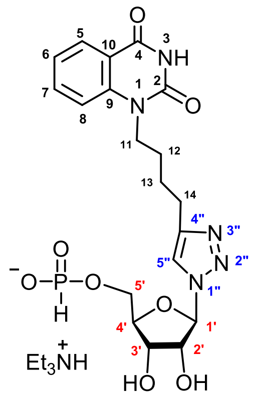

1′-[4″-(2,4-dioxo-quinazoline-1-yl-butyl)-1″,2″,3″-triazol-1″-yl]-β-D-ribofuranose-5′-yl *H*-phosphonate triethylammonium salt (24b).

White powder. Yield 67 %. ^{1}H NMR (MeOD, 400 MHz): δ 8.12 (s, 1H, H-5''), 8.11 (dd, *J* = 7.9, 1.8 Hz, 1H, H-5), 7.78 (ddd, *J* = 8.7, 7.3, 1.7 Hz, 1H, H-7), 7.43 (d, *J* = 8.5 Hz, 1H, H-8), 7.29 (t, *J* = 7.5 Hz, 1H, H-6), 6.78 (d, *J* = 620.3 Hz, 1H, PH), 6.04 (d, *J* = 4.8 Hz, 1H, H-1'), 4.53 (t, *J* = 4.9 Hz, 1H, H-2'), 4.36 (t, *J* = 4.6 Hz, 1H, H-3'), 4.25 (q, *J* = 3.6 Hz, 1H, H-4'), 4.20 – 4.15 (m, 2H, H-11), 4.13 – 4.00 (m, 2H, H-5'), 3.19 (q, *J* = 7.3 Hz, 6H, CH_2), 2.83 (t, *J* = 7.1 Hz, 2H, H-14), 1.90 – 1.73 (m, 4H, H-12,13), 1.31 (t, *J* = 7.3 Hz, 9H, CH_3). ^{13}C NMR (MeOD, 101 MHz): δ 164.12 (s, C-4), 152.09 (s, C-2), 149.08 (s, C-4''), 142.42 (s, C-9), 136.83 (s, C-7), 129.08 (s, C-5), 123.95 (s, C-6), 121.70 (s, C-5''), 117.20 (s, C-10), 115.81 (s, C-8), 94.28 (s, C-1'), 85.74 (d, *J* = 7.7 Hz, C-4'), 76.87 (s, C-3'), 72.24 (s, C-2'), 64.44 (d, *J* = 4.5 Hz, C-5'), 47.73 (s, CH_2), 43.30 (s, C-11), 27.70 (s, C-12), 27.47 (s, C-13), 25.82 (s, C-14), 9.23 (s, CH_3). ^{31}P NMR (MeOD, 162 MHz): δ 4.6. ESI-MS: calcd. for $C_{19}H_{23}O_8N_5P$ [*M*-H]$^-$ = 480.1; found [*M*-H]$^-$ = 480.1. Anal., %: C 51.53; H 6.77; N 14.41; P 5.31. $C_{25}H_{39}O_8N_6P$. Calcd., %: C 51.54; H 6.75; N 14.43; P 5.32.

6. General procedure for the synthesis of 5′-phosphates of 1″,2″,3″-triazolyl nucleoside analogues

Starting nucleotide **21a,b** or **22a,b** (1eq) was heated *in vacuo* (60°C, 0.05 Torr) for 30 min, then the flask was filled with argon, and this operation was repeated 2 more times. The dried nucleoside was dissolved in dry DCM (10 ml per 1 mmol of nucleoside) and 10 eq of triethylamine was added. A flask was equipped with a rubber septum, and argon inlet tube and cooled with a water bath (18–20°C). Then 5 eq of the TMSCl was added by the syringe under an argon atmosphere. The flask was stirred for 30 min at room temperature and then 1.05 eq of crystalline iodine was added in one portion and the mixture was stirred for an additional 30 min. Then all volatile components were removed on a rotary evaporator and dried *in vacuo* (40°C, 0.05 Torr). The residue was purified by flash chromatography to isolate compounds **31a,b** and **32a,b** (gradient elution CH_2Cl_2:MeOH+0.5%Et_3N 25:1 to pure MeOH). To remove the isopropylidene protection group, compounds **31a,b**, **32a,b** were dissolved in aqueous trifluoroacetic acid (50% v/v; 4 ml per 1 mmol of nucleotide) and left to stir for 45 min at room temperature. The solution was concentrated on rotary evaporator and dried *in vacuo* (40°C, 0.05 Torr). The residue was purified by flash chromatography to give compounds **25a,b**, **26a,b** (eluent - a mixture of $CHCl_3$/MeOH from 30:1 to pure MeOH).

6.1 Characterization of compounds

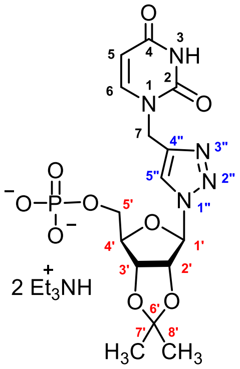

1′-[4″-(2,4-dioxo-pyrimidine-1-yl-methyl)-1″,2″,3″-triazol-1″-yl]-2′,3′-*O*-isopropylidene-β-D-ribofuranose-5′-yl phosphate bis(triethylammonium) salt (31a).

White powder. Yield 40 %. ^{1}H NMR ($CDCl_3$, 500 MHz): δ 8.35 (s, 1H, H-5’’), 7.54 (d, J = 7.9 Hz, 1H, H-6), 6.17 (d, J = 2.5 Hz, 1H, H-1’), 5.60 (d, J = 7.9 Hz, 1H, H-5), 5.18 – 5.11 (m, 1H, H-2’), 5.01 – 4.93 (m, 3H, H-3’, H-7), 4.52 – 4.43 (m, 1H, H-4’), 3.94 – 3.83 (m, 2H, H-5’), 2.89 (q, J = 7.3 Hz, 12H, CH_2), 1.53 (s, 3H, H-7’), 1.30 (s, 3H, H-8’), 1.16 (t, J = 7.3 Hz, 18H, CH_3). ^{13}C NMR ($CDCl_3$, 126 MHz): δ 164.63 (s, C-4), 151.53 (s, C-2), 144.96 (s, C-6), 142.70 (s, C-4”), 123.04 (s, C-5”), 113.66 (s, C-6’), 102.51 (s, C-5), 95.59 (s, C-1’), 86.61 (d, J = 8.7 Hz, C-4’), 85.38 (s, C-2’), 82.10 (s, C-3’), 64.84 (s, C-5’), 45.67 (s, CH_2), 42.92 (s, C-7), 27.19 (s, C-7’), 25.32 (s, C-8’), 9.11 (s, CH_3). ^{31}P NMR ($CDCl_3$, 162 MHz): δ 2.0. MALDI-MS: calcd. for $C_{15}H_{19}O_9N_5P$ [M-H]$^-$ = 444.1; found [M-H]$^-$ =

444.0. Anal., %: C 50.08; H 7.77; N 15.12; P 4.76. $C_{27}H_{50}O_9N_7P$. Calcd., %: C 50.07; H 7.78; N 15.14; P 4.78.

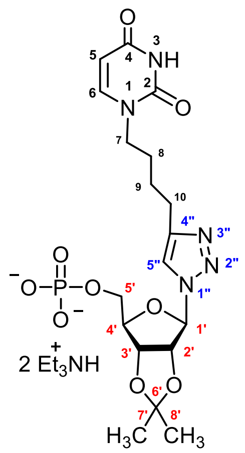

1′-[4″-(2,4-dioxo-pyrimidine-1-yl-butyl)-1″,2″,3″-triazol-1″-yl]-2′,3′-*O*-isopropylidene-β-D-ribofuranose-5′-yl phosphate bis(triethylammonium) salt (32a).

White powder. Yield 53 %. ^{1}H NMR (MeOD, 400 MHz): δ 8.11 (s, 1H, H-5”), 7.61 (d, *J* = 7.9 Hz, H-6), 6.18 (d, *J* = 2.6 Hz, 1H, H-1’), 5.64 (d, *J* = 7.8 Hz, 1H, H-5), 5.29 (dd, *J* = 5.9, 2.6 Hz, 1H, H-2’), 5.07 (dd, *J* = 6.0, 1.7 Hz, 1H, H-3’), 4.50 (t, *J* = 4.9 Hz, 1H, H-4’), 3.93 – 3.75 (m, 4H, H-5’, H-7), 3.11 (q, *J* = 7.3 Hz, 12H, CH_2), 2.79 – 2.72 (m, 2H, H-10), 1.79 – 1.67 (m, 4H, H-8, H-9), 1.57 (s, 3H, H-7’), 1.38 (s, 3H, H-8’), 1.28 (t, *J* = 7.3 Hz, 18H, CH_3). ^{13}C NMR (MeOD, 101 MHz): δ 166.72 (s, C=O, C-4), 152.82 (s, C=O, C-2), 149.22 (s, C-4”), 147.40 (s, C-6), 122.40 (s, C-5’’), 114.81 (s, C-6’), 102.20 (s, C-5), 96.26 (s, C-1’), 87.79 (d, *J* = 9.0 Hz, C-4’), 86.13 (s, C-2’), 83.57 (s, C-3’), 65.81 (d, *J* = 5.1 Hz, C-5’), 49.22 (s, C-7), 47.29 (s, CH_2), 29.36 (s, C-8), 27.39 (s, C-7’), 27.17 (s, C-9), 25.75 (s, C-10), 25.45 (s, C-8’), 9.23 (s, CH_3). ^{31}P NMR (MeOD, 162 MHz): δ 1.1. MALDI-MS: calcd. for $C_{18}H_{25}N_5O_9P$ [*M*-H]$^-$ = 486.1; found [*M*-H]$^-$ = 486.1. Anal., %: C 52.28; H 8.17; N 14.25; P 4.47. $C_{30}H_{56}N_7O_9P$. Calcd., %: C 52.24; H 8.18; N 14.21; P 4.49.

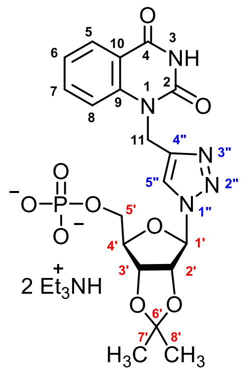

1′-[4″-(2,4-dioxo-quinazoline-1-yl-methyl)-1″,2″,3″-triazol-1″-yl]-2′,3′-*O*-isopropylidene-β-D-ribofuranose-5′-yl phosphate triethylammonium salt (31b).

White powder. Yield 47 %. ^{1}H NMR (MeOD, 400 MHz): δ 8.36 (s, 1H, H-5''), 8.11 (d, *J* = 7.8 Hz, 1H, H-5), 7.74 (ddd, *J* = 8.7, 7.2, 1.7 Hz, 1H, H-7), 7.57 (d, *J* = 8.5 Hz, 1H, H-8), 7.28 (t, *J* = 7.6 Hz, 1H, H-6), 6.18 (d, *J* = 2.5 Hz, 1H, H-1'), 5.46 (s, 2H, H-11), 5.30 (dd, *J* = 6.0, 2.5 Hz, 1H, H-2'), 5.08 (dd, *J* = 6.0, 1.7 Hz, 1H, H-3'), 4.51 (t, *J* = 5.2 Hz, 1H, H-4'), 3.91 – 3.83 (m, 2H, H-5'), 2.99 (q, *J* = 7.3 Hz, 12H, CH_2), 1.55 (s, 3H, H-7'), 1.37 (s, 3H, H-8'), 1.23 (t, *J* = 7.3 Hz, 18H, CH_3). ^{13}C NMR (MeOD, 101 MHz): δ 164.09 (s, C-4), 152.26 (s, C-2), 144.52 (s, C-9), 142.30 (s, C-4''), 136.73 (s, C-7), 128.98 (s, C-5), 124.22 (s, C-6), 124.20 (s, C5''), 117.29 (s, C-10), 116.25 (s, C-8), 114.78 (s, C-6'), 95.98 (s, C-1'), 88.30 (d, *J* = 9.0 Hz, C-4'), 86.01 (s, C-3'), 83.64 (s, C-2'), 65.40 (d, *J* = 4.9 Hz, C-5'), 46.86 (s, CH_2), 39.01 (s, C-11), 27.31 (s, C-7'), 25.42 (s, C-8'), 9.35 (s, CH_3). ^{31}P NMR (MeOD, 162 MHz): δ 1.6. MALDI-MS: calcd. for $C_{19}H_{21}N_5O_9P$ [*M*-H]$^-$ = 494.1; found [*M*-H]$^-$ = 494.0. Anal., %: C 53.38; H 7.54; N 14.08; P 4.47. $C_{30}H_{56}N_7O_9P$. Calcd., %: C 53.36; H 7.51; N 14.05; P 4.44.

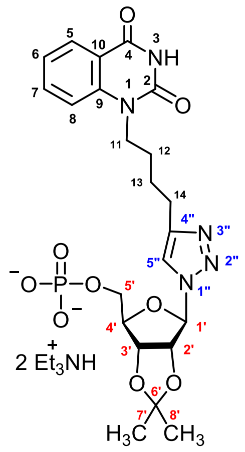

1′-[4″-(2,4-dioxo-quinazoline-1-yl-butyl)-1″,2″,3″-triazol-1″-yl]-2′,3′-*O*-isopropylidene-β-D-ribofuranose-5′-yl phosphate bis(triethylammonium) salt (32b).

White powder. Yield 55 %. ^{1}H NMR (MeOD, 400 MHz): δ 8.08 (s, 1H, H-5’’), 8.01 – 7.96 (m, 1H, H-5), 7.64 – 7.57 (m, 1H, H-7), 7.23 – 7.14 (m, 2H, H-6, H-8), 6.17 (d, *J* = 2.4 Hz, 1H, H-1’), 5.29 (dd, *J* = 6.0, 2.5 Hz, 1H, H-2’), 5.06 (dd, *J* = 6.0, 1.8 Hz, 1H, H-3’), 4.53 – 4.44 (m, 1H, H-4’), 4.09 – 3.98 (m, 2H, H-11), 3.94 – 3.80 (m, 2H, H-5’), 3.14 (q, *J* = 7.3 Hz, 9H, CH_2), 2.78 (m, 2H, H-14), 1.78 – 1.69 (m, 4H, H-12, H-13), 1.56 (s, 3H, H-7’), 1.37 (s, 3H, H-8’), 1.28 (t, *J* = 7.3 Hz, 18H, CH_3). ^{13}C NMR (MeOD, 101 MHz): δ 163.37 (s, C=O, C-4), 151.62 (s, C=O, C-2), 148.56 (s, C-4”), 139.94 (s, C-9), 135.35 (s, C-7), 127.89 (s, C-5), 123.14 (s, C-6), 121.62 (s, C-5”), 115.43 (s, C-10), 114.59 (s, C-8), 113.99 (s, C-6’), 95.16 (s, C-1’), 86.93 (d, *J* = 8.7 Hz, C-4’), 85.20 (s, C-2’), 82.69 (s, C-3’), 65.00 (d, *J* = 5.2 Hz, C-5’), 46.55 (s, CH_2), 40.46 (s, C-11), 27.45 (s, C-12), 26.91 (s, C-13), 26.53 (s, C-7’), 25.01 (s, C-14), 24.60 (s, C-8’), 8.32 (s, CH_3). ^{31}P NMR (MeOD, 162 MHz): δ 1.12. ESI-MS: calcd. for $C_{22}H_{27}N_5O_9P$ [*M*-H]$^-$ = 536.2; found [*M*-H]$^-$ = 536.1. Anal., %: C 55.18; H 7.94; N 13.28; P 4.17. $C_{34}H_{58}N_7O_9P$. Calcd., %: C 55.20; H 7.90; N 13.25; P 4.19.

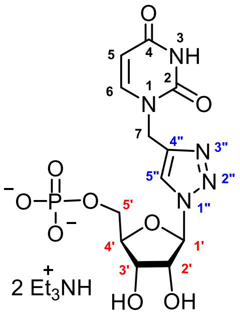

1′-[4″-(2,4-dioxo-pyrimidine-1-yl-methyl)-1″,2″,3″-triazol-1″-yl]-β-D-ribofuranose-5′-yl phosphate bis(triethylammonium) salt (25a).

White powder. Yield 63 %. ^{1}H NMR (MeOD, 400 MHz): δ 8.46 (s, 1H, H-5”), 7.73 (d, *J* = 7.9 Hz, 1H, H-6), 6.04 (d, *J* = 4.8 Hz, 1H, H-1’), 5.67 (d, *J* = 7.9 Hz, 1H, H-5), 5.06 (s, 2H, H-7), 4.57 (t, *J* = 4.8 Hz, 1H, H-3’), 4.40 – 4.37 (m, 1H, H-2’), 4.26 – 4.22 (m, 1H, H-4’), 4.09 – 3.98 (m, 2H, H-5’), 2.99 (q, *J* = 7.3 Hz, 12H, CH_2), 1.22 (t, *J* = 7.3 Hz, 18H, CH_3). ^{13}C NMR (MeOD, 126 MHz): δ 166.53 (s, C-4), 152.47 (s, C-2), 146.84 (s, C-6), 143.93 (s, C-4"), 124.39 (s, C-5"), 102.68 (s, C-5), 94.11 (s, C-1'), 84.36 (d, *J* = 7.7 Hz, C-4'), 76.29 (s, C-2'), 71.64 (s, C-3'), 68.04 (d, *J* = 5.6 Hz, C-5'), 65.62 (s, C-7), 43.89 (s, CH_2), 16.35 (s, CH_3). ^{31}P NMR ($CDCl_3$, 162 MHz): δ - 0.5. ESI-MS: calcd. for $C_{12}H_{15}N_5O_9P$ [*M*-H]$^-$ = 404.1; found [*M*-H]$^-$ = 403.9. Anal., %: C 47.41; H 7.64; N 16.12; P 5.07. $C_{24}H_{46}N_6O_9P$. Calcd., %: C 47.44; H 7.63; N 16.14; P 5.10.

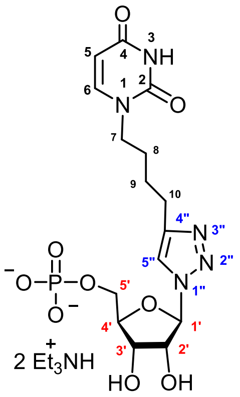

1′-[4″-(2,4-dioxo-pyrimidine-1-yl-butyl)-1″,2″,3″-triazol-1″-yl]-β-D-ribofuranose-5′-yl phosphate bis(triethylammonium) salt (26a).

Sticky solid. Yield 78 %. ^{1}H NMR (MeOD, 400 MHz): δ 8.18 (s, 1H, H-5”), 7.61 (d, *J* = 7.8 Hz, 1H, H-6), 6.02 (d, *J* = 5.1 Hz, 1H, H-1’), 5.65 (d, *J* = 7.8 Hz, 1H, H-5), 4.54 (t, *J* = 5.0 Hz, 1H, H-2’), 4.41 – 4.33 (m, 1H, H-3’), 4.27 – 4.20 (m, 1H, H-4’), 4.10 – 3.99 (m, 2H, H-5’), 3.82 – 3.75 (m, 2H, H-7), 3.07 (q, *J* = 7.3 Hz, 12H, CH_2), 2.83 – 2.71 (m, 2H, H-10), 1.78 – 1.65 (m, 4H, H-8, H-9), 1.26 (t, J = 7.3 Hz, 18H, CH_3). ^{13}C NMR (MeOD, 101 MHz): δ 166.77 (s, C=O, C-4), 152.82 (s, C=O, C-2), 148.98 (s, C-4”), 147.42 (s, C-6), 121.80 (s, C-5’’), 102.20 (s, C-5), 94.36 (s, C-1’), 86.30 (d, *J* = 8.8 Hz, C-4’), 77.11 (s, C-2’), 72.50 (s, C-3’), 65.70 (d, *J* = 5.0 Hz, C-5’), 48.73 (s, C-7, overlapped with the signal of MeOD), 47.18 (s, CH_2), 29.36 (s, C-8), 27.25 (s, C-9), 25.76 (s, C-10), 9.35 (s, CH_3). ^{31}P NMR (MeOD, 162 MHz): δ 1.36. ESI-MS: calcd. for $C_{15}H_{21}N_5O_9P$ [*M*-H]$^-$ = 446.1; found [*M*-H]$^-$ =

446.0. Anal., %: C 49.40; H 8.10; N 15.12; P 4.74. $C_{27}H_{52}N_7O_9P$. Calcd., %: C 49.41; H 8.07; N 15.09; P 4.77.

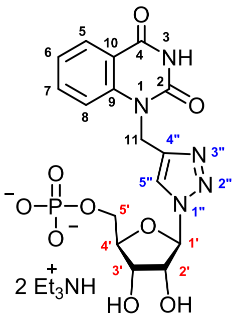

1′-[4″-(2,4-dioxo-quinazoline-1-yl-methyl)-1″,2″,3″-triazol-1″-yl]-β-D-ribofuranose-5′-yl phosphate bis(triethylammonium) salt (25b).

Sticky solid. Yield 56 %. ^{1}H NMR (MeOD, 500 MHz): δ 8.41 (s, 1H, H-5”), 8.10 (dd, *J* = 7.9, 1.6 Hz, 1H, H-5), 7.77 – 7.65 (m, 1H, H-7), 7.55 (d, *J* = 8.5 Hz, 1H, H-8), 7.27 (t, 1H, H-6), 6.01 (d, *J* = 4.9 Hz, 1H, H-1’), 5.47 (d, *J* = 2.6 Hz, 2H, H-11), 4.53 (t, *J* = 4.9 Hz, 1H, H-2’), 4.37 – 4.34 (m, 1H, H-3’), 4.25 – 4.21 (m, 1H, H-4’), 4.10 – 3.99 (m, 2H, H-5’), 3.12 (q, *J* = 7.3 Hz, 12H, CH_2), 1.27 (t, *J* = 7.3 Hz, 18H, CH_3). ^{13}C NMR (MeOD, 126 MHz): δ 164.18 (s, C=O, C-4), 152.36 (s, C=O, C-2), 144.36 (s, C-9), 142.35 (s, C-4’’), 136.78 (s, C-7), 129.00 (s, C-5), 124.21 (s, C-6), 123.32 (s, C-10), 117.36 (s, C-5’’), 116.28 (s, C-8), 94.47 (s, C-1’), 86.29 (d, *J* = 8.7 Hz, C-4’), 77.08 (s, C-2’), 72.35 (s, C-3’), 65.78 (d, *J* = 5.0 Hz, C-5’), 47.48 (s, CH_2), 39.01 (s, C-11), 9.23 (s, CH_3). ^{31}P NMR (MeOD, 162 MHz): δ 2.9. ESI-MS: calcd. for $C_{16}H_{17}N_5O_9P$ $[M\text{-}H]^-$ = 454.1; found $[M\text{-}H]^-$ = 453.9. Anal., %: C 51.10; H 7.39; N 14.92; P 4.70. $C_{28}H_{48}N_7O_9P$. Calcd., %: C 51.13; H 7.36; N 14.91; P 4.71.

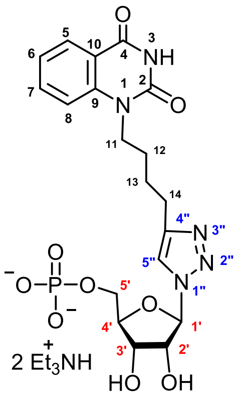

1′-[4″-(2,4-dioxo-quinazoline-1-yl-butyl)-1″,2″,3″-triazol-1″-yl]-β-D-ribofuranose-5′-yl phosphate bis(triethylammonium) salt (26b).

Sticky solid.. Yield 70%. ^{1}H NMR (MeOD, 400 MHz): δ 8.15 (s, 1H, H-5"), 7.99 (dd, *J* = 8.0, 1.5 Hz, 1H, H-5), 7.61 (ddd, *J* = 8.5, 7.3, 1.5 Hz, 1H, H-7), 7.24 – 7.17 (m, 2H, H-6,8), 6.02 (d, *J* = 4.9 Hz, 1H, H-1'), 4.56 (t, *J* = 5.0 Hz, 1H, H-2'), 4.43 – 4.37 (m, 1H, H-3'), 4.28 – 4.22 (m, *J* = 4.1 Hz, 1H, H-4'), 4.12 – 3.99 (m, 4H, H-5', H-11), 3.01 (q, *J* = 7.3 Hz, 12H, CH_2), 2.78 (t, *J* = 7.1 Hz, 2H, H-14),

1.77 – 1.70 (m, 4H, H-12,13), 1.24 (t, *J* = 7.3 Hz, 18H, CH_3). ^{13}C NMR (MeOD, 101 MHz): δ 164.22 (s, C-4), 152.38 (s, C-2), 149.07 (s, C-9), 140.83 (s, C-4''), 136.17 (s, C-7), 128.67 (s, C-5), 123.93 (s, C-6), 121.94 (s, C-10), 116.37 (s, C-5''), 115.36 (s, C-8), 94.22 (s, C-1'), 86.23 (d, *J* = 8.6 Hz, C-4'), 76.93 (s, C-2'), 72.58 (s, C3'), 65.73 (d, *J* = 4.7 Hz, C-5'), 46.93 (s, CH_2), 41.26 (s, C-11), 28.25 (s, C-12), 27.86 (s, C-13), 25.83 (s, C-14), 9.43 (s, CH_3). ^{31}P NMR (MeOD, 162 MHz): δ 2.4. ESI-MS: calcd. for $C_{19}H_{23}N_5O_9P$ [*M*-H]$^-$ = 496.1; found [*M*-H]$^-$ = 496.0. Anal., %: C 53.23; H 7.79; N 14.02; P 4.45. $C_{31}H_{54}N_7O_9P$. Calcd., %: C 53.21; H 7.78; N 14.01; P 4.43.

7. NMR spectra

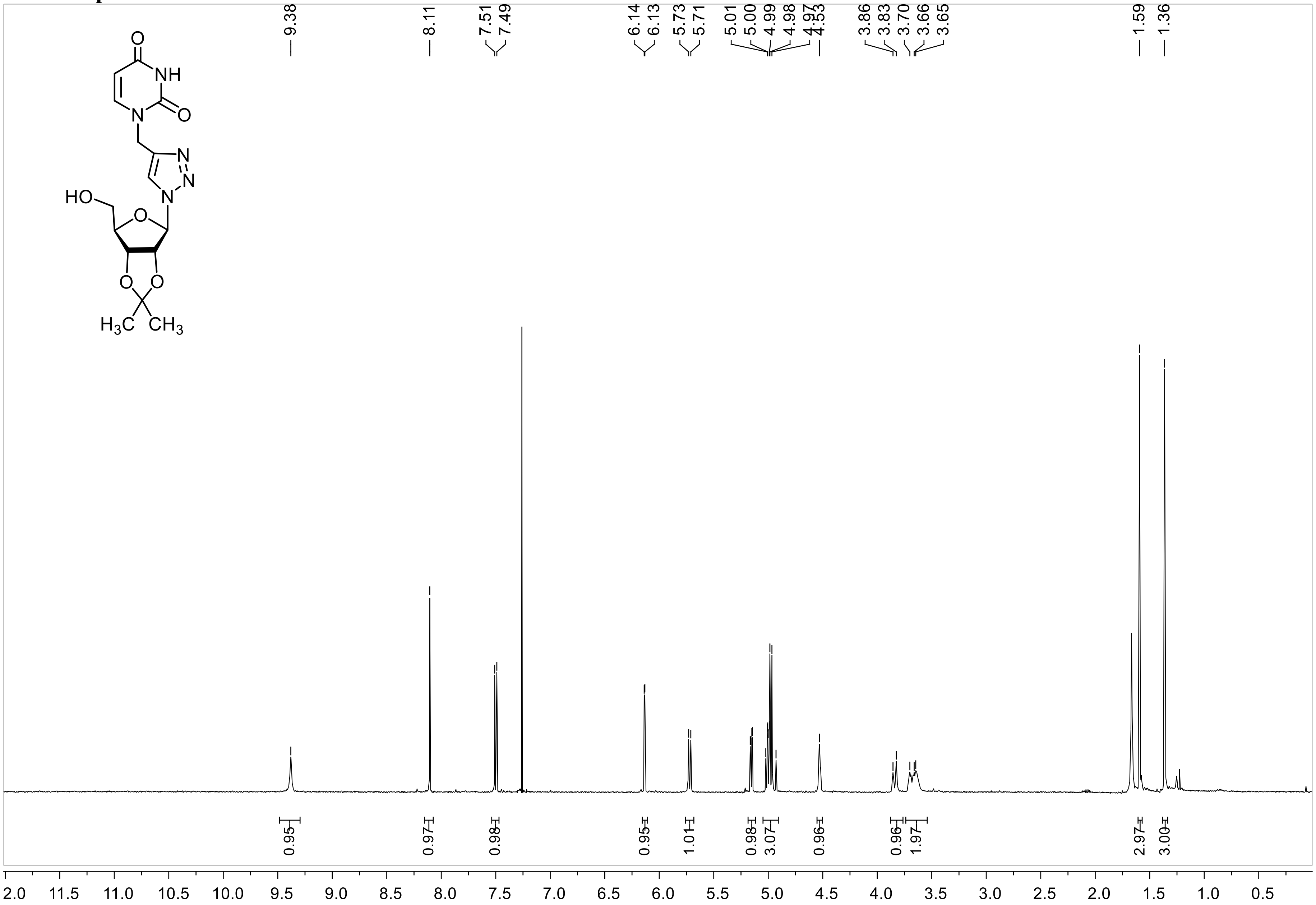

Figure S1. ^{1}H NMR spectrum of **9a** in $CDCl_3$

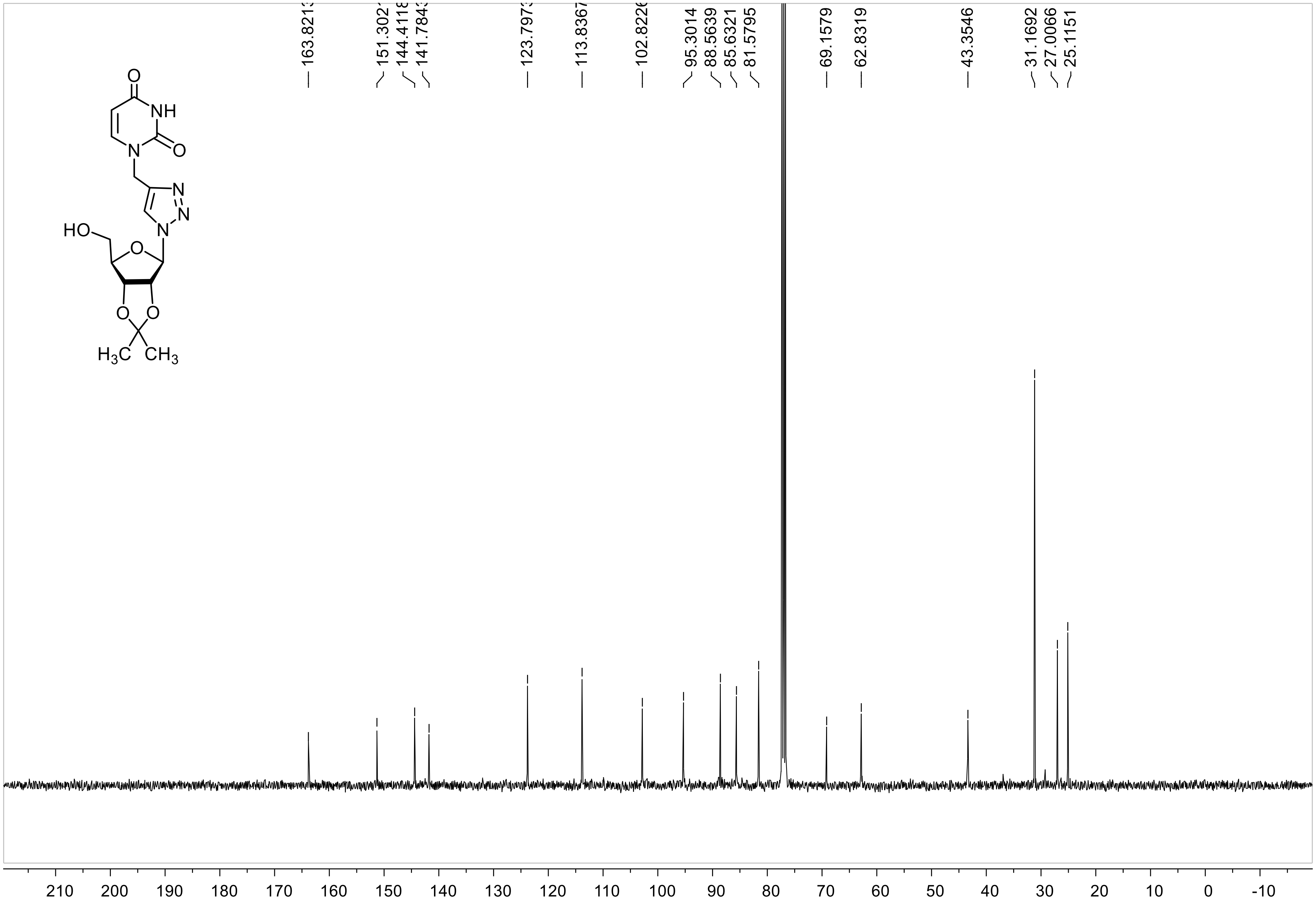

Figure S2. ^{13}C NMR spectrum of **9a** in $CDCl_3$

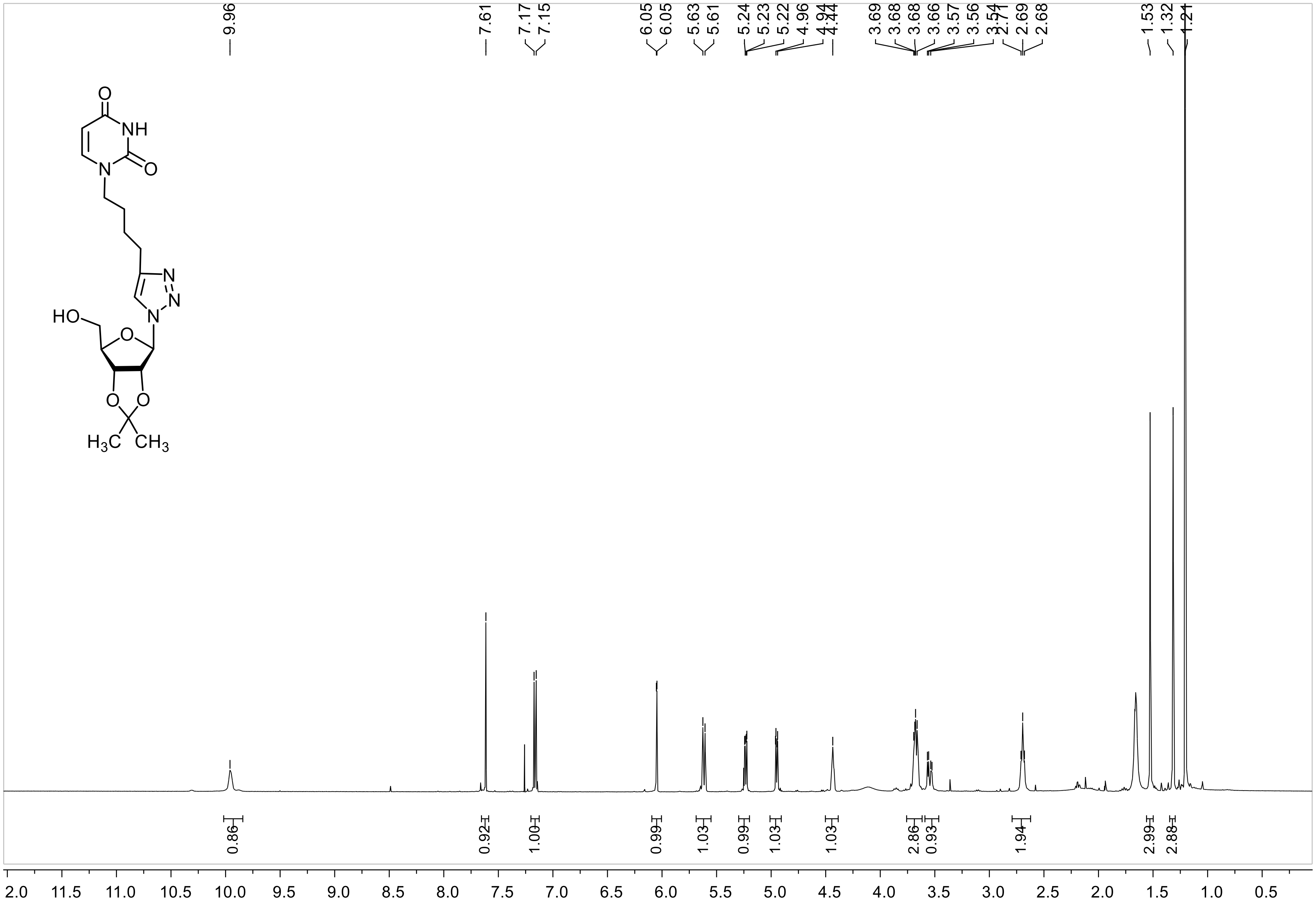

Figure S3. ^{1}H NMR spectrum of **10a** in $CDCl_3$

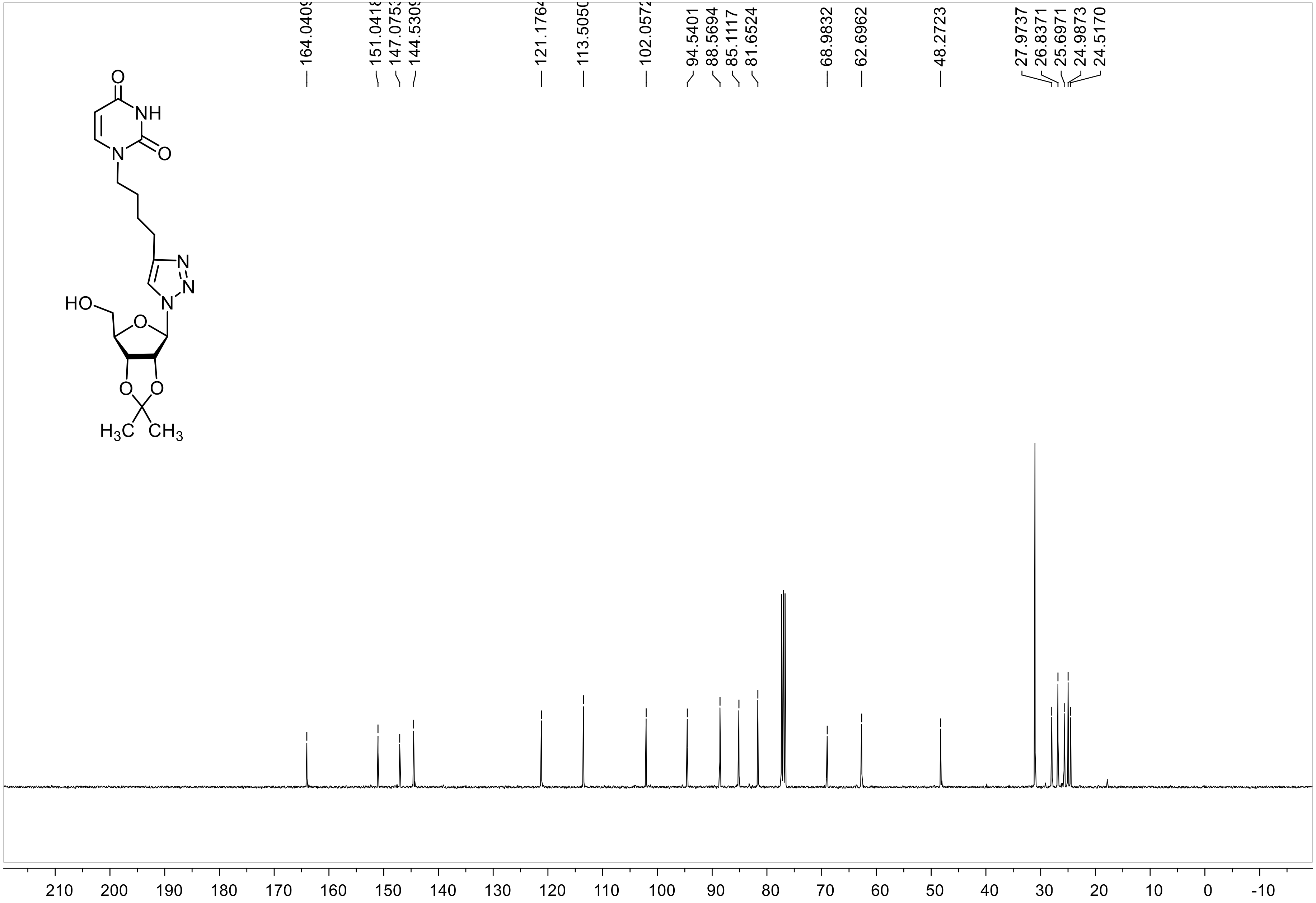

Figure S4. ^{13}C NMR spectrum of **10a** in $CDCl_3$

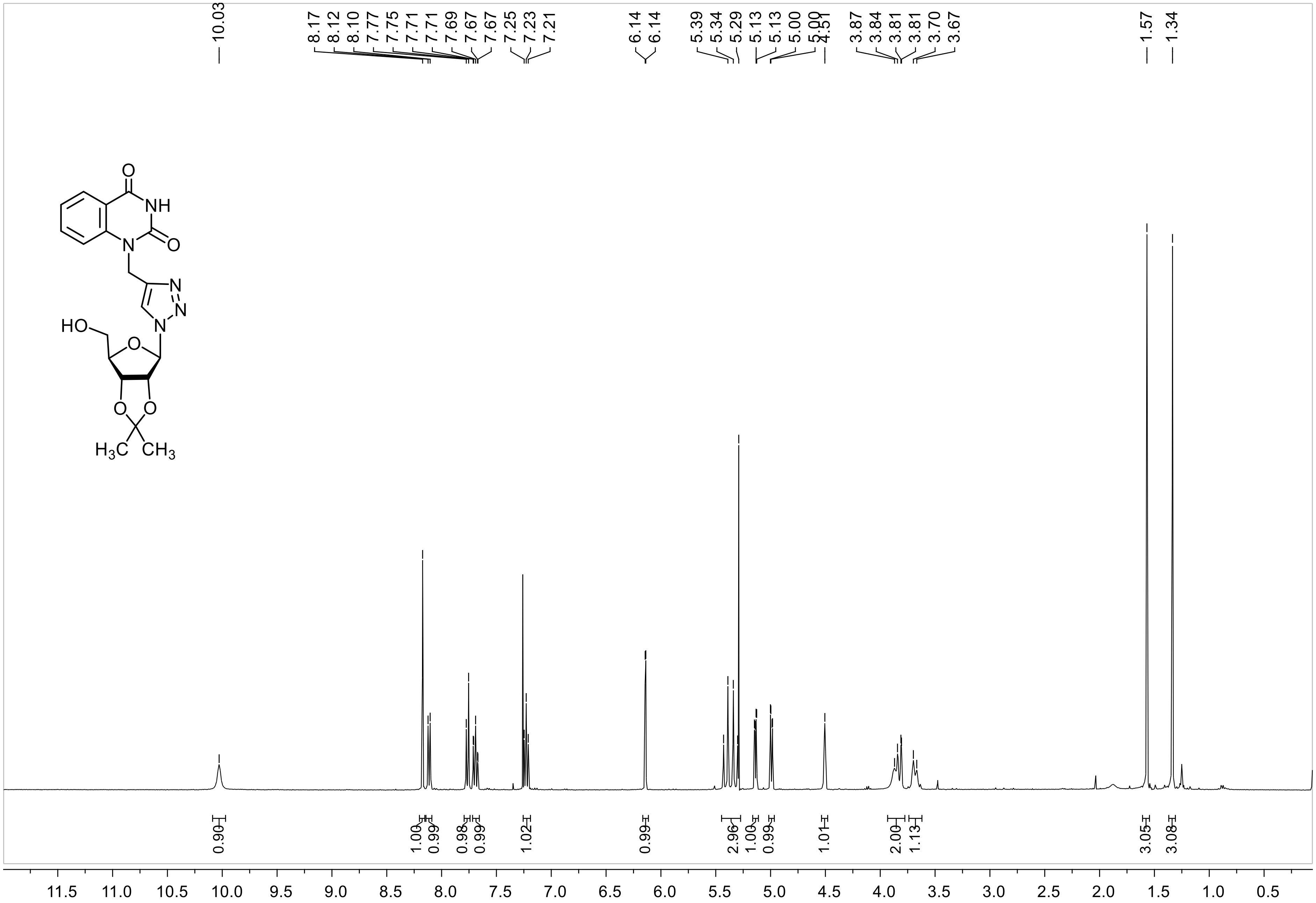

Figure S5. ^{1}H NMR spectrum of **9b** in $CDCl_3$

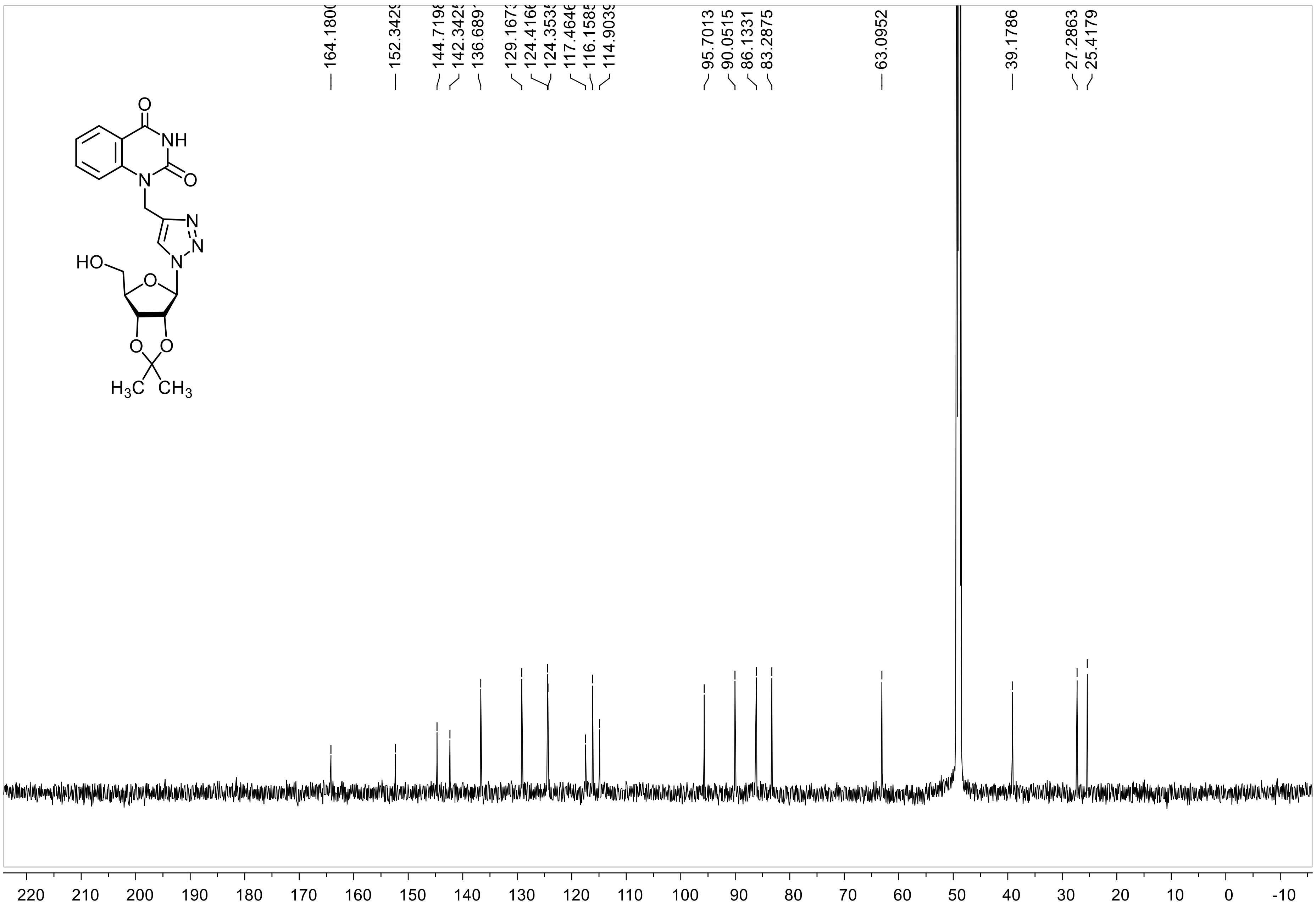

Figure S6. ^{13}C NMR spectrum of **9b** in $CDCl_3$

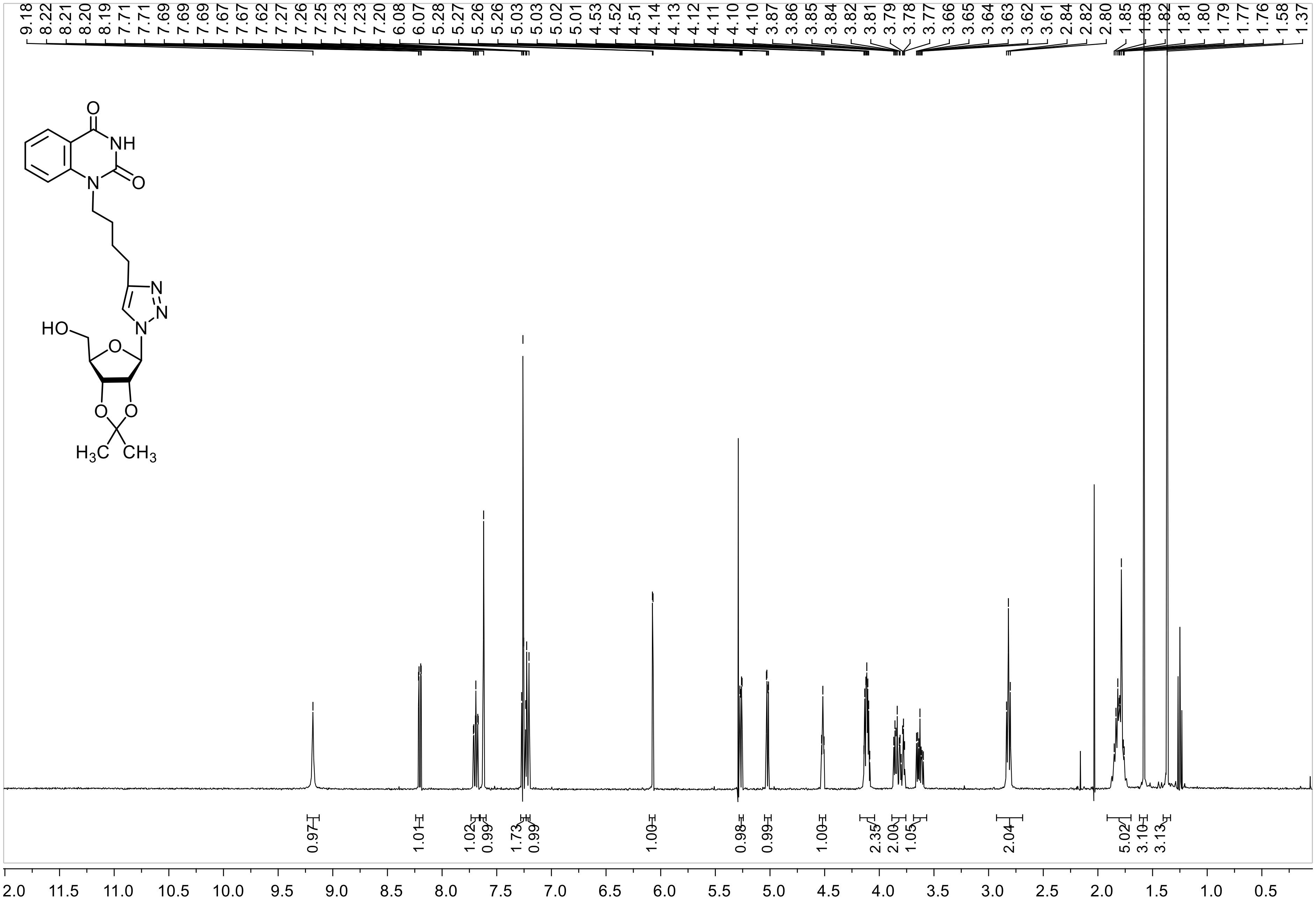

Figure S7. ^1H NMR spectrum of **10b** in $CDCl_3$

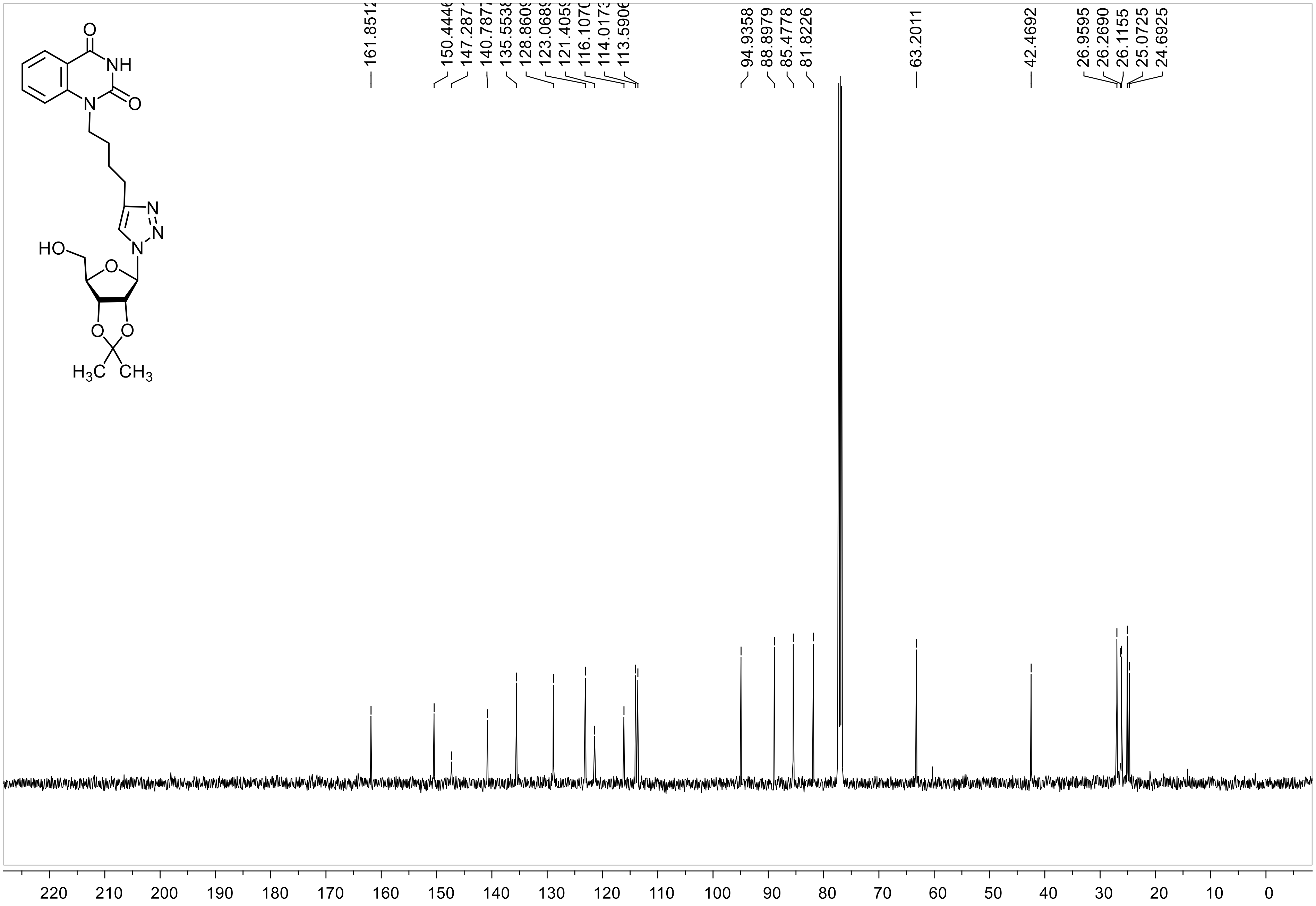

Figure S8. ^{13}C NMR spectrum of **10b** in $CDCl_3$

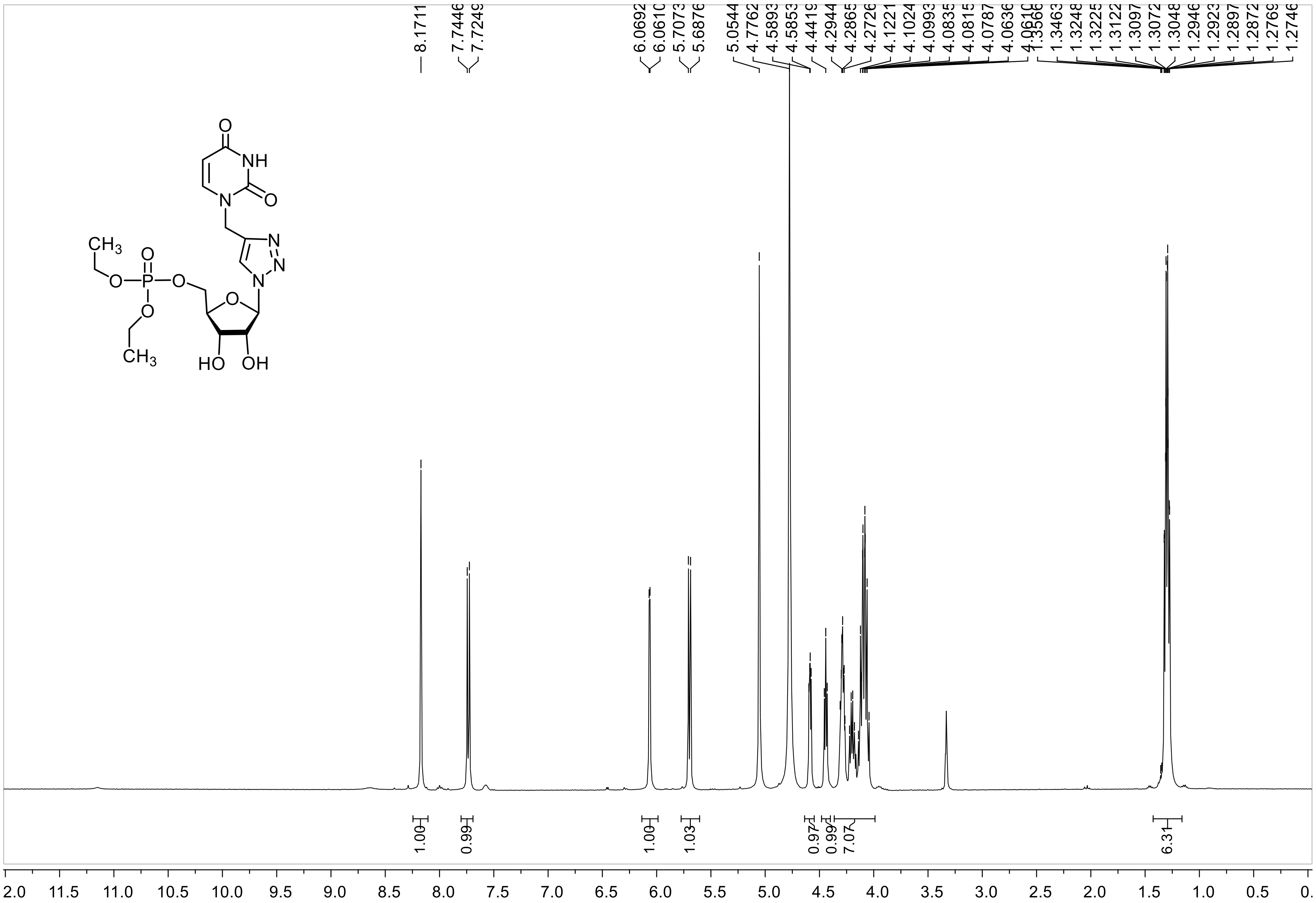

Figure S9. ^{1}H NMR spectrum of **11a** in CD_3OD

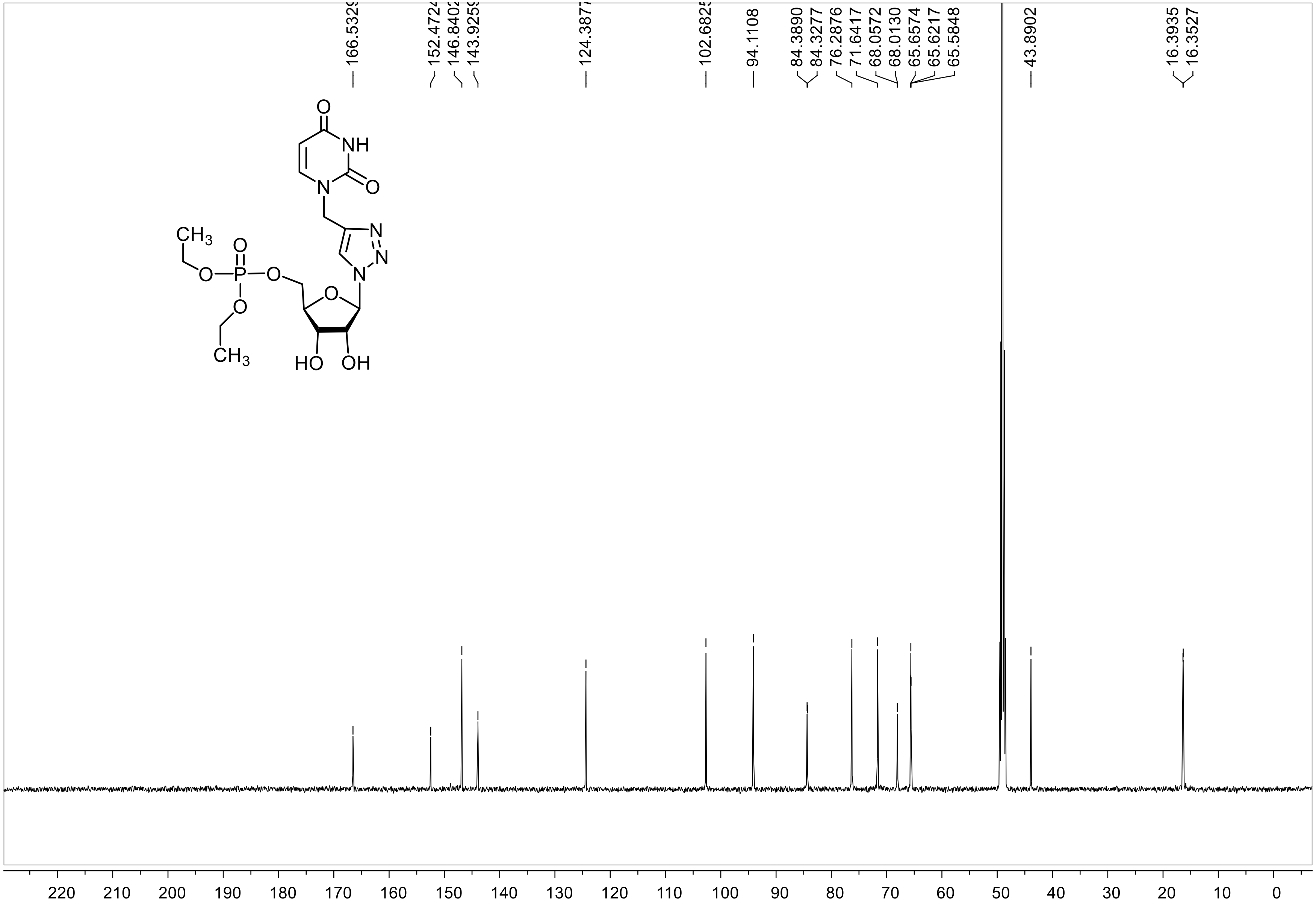

Figure S10. ^{13}C NMR spectrum of **11a** in CD_3OD

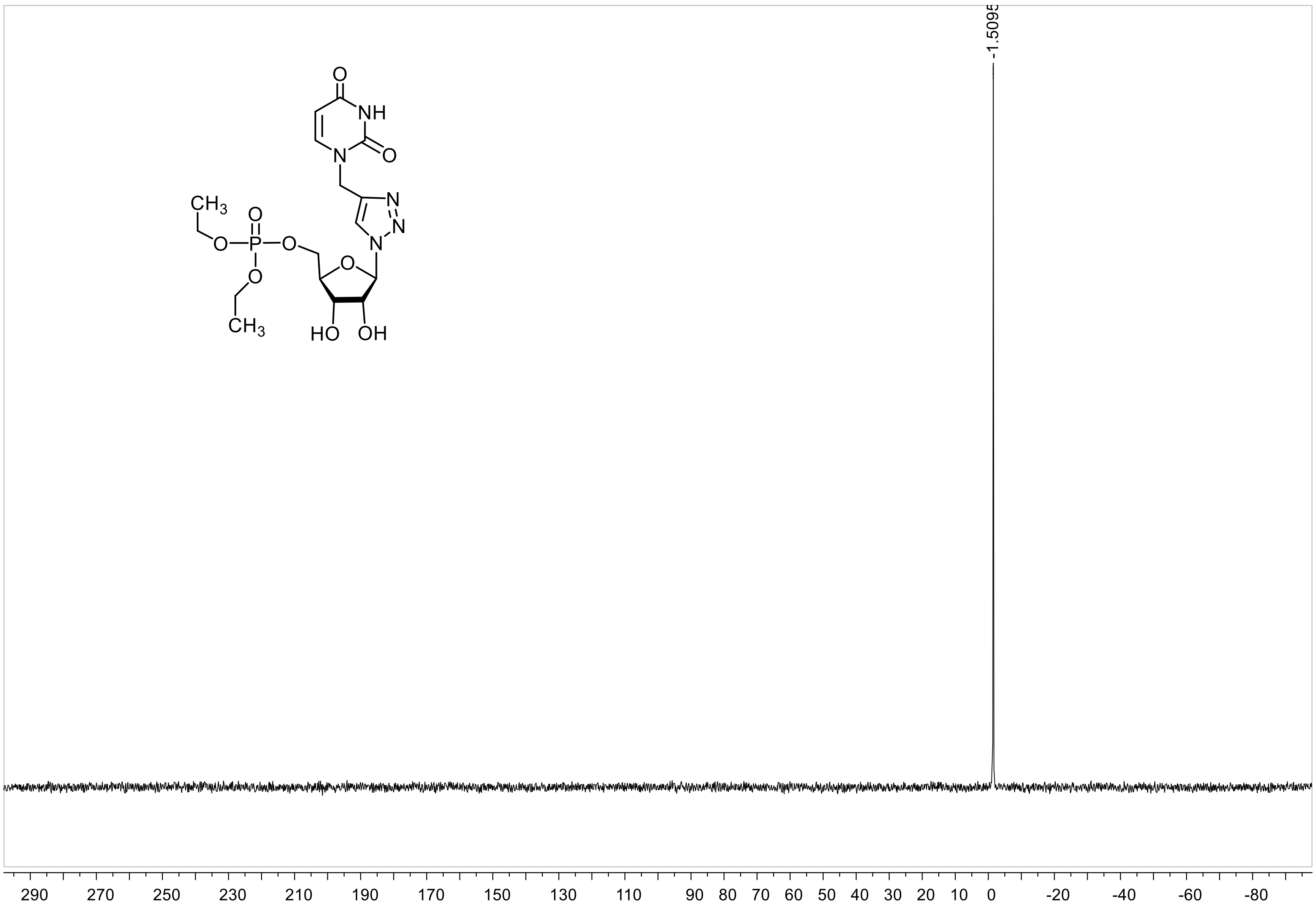

Figure S11. ^{31}P NMR spectrum of **11a** in CD_3OD

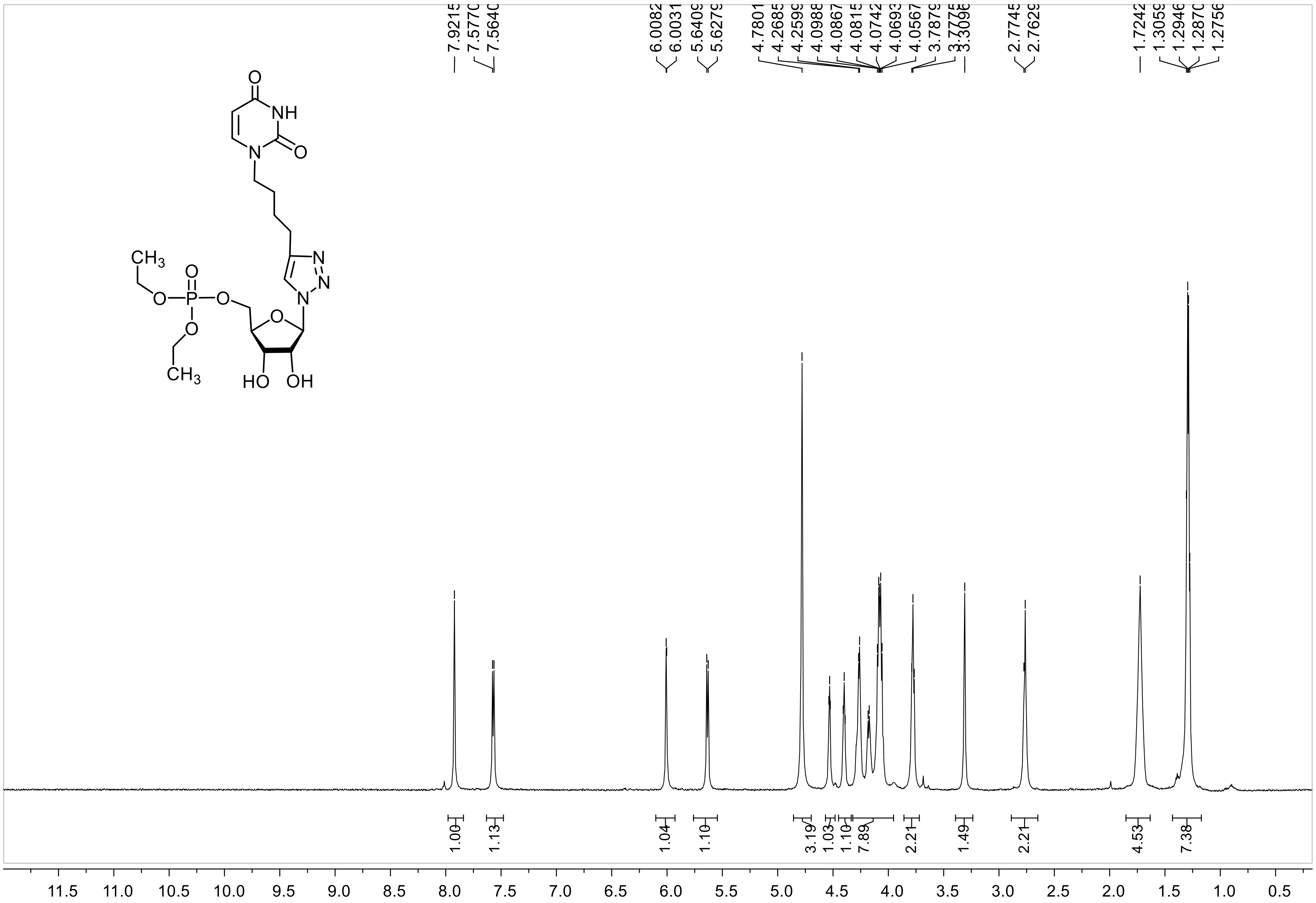

Figure S12. ^{1}H NMR spectrum of **12a** in CD_3OD

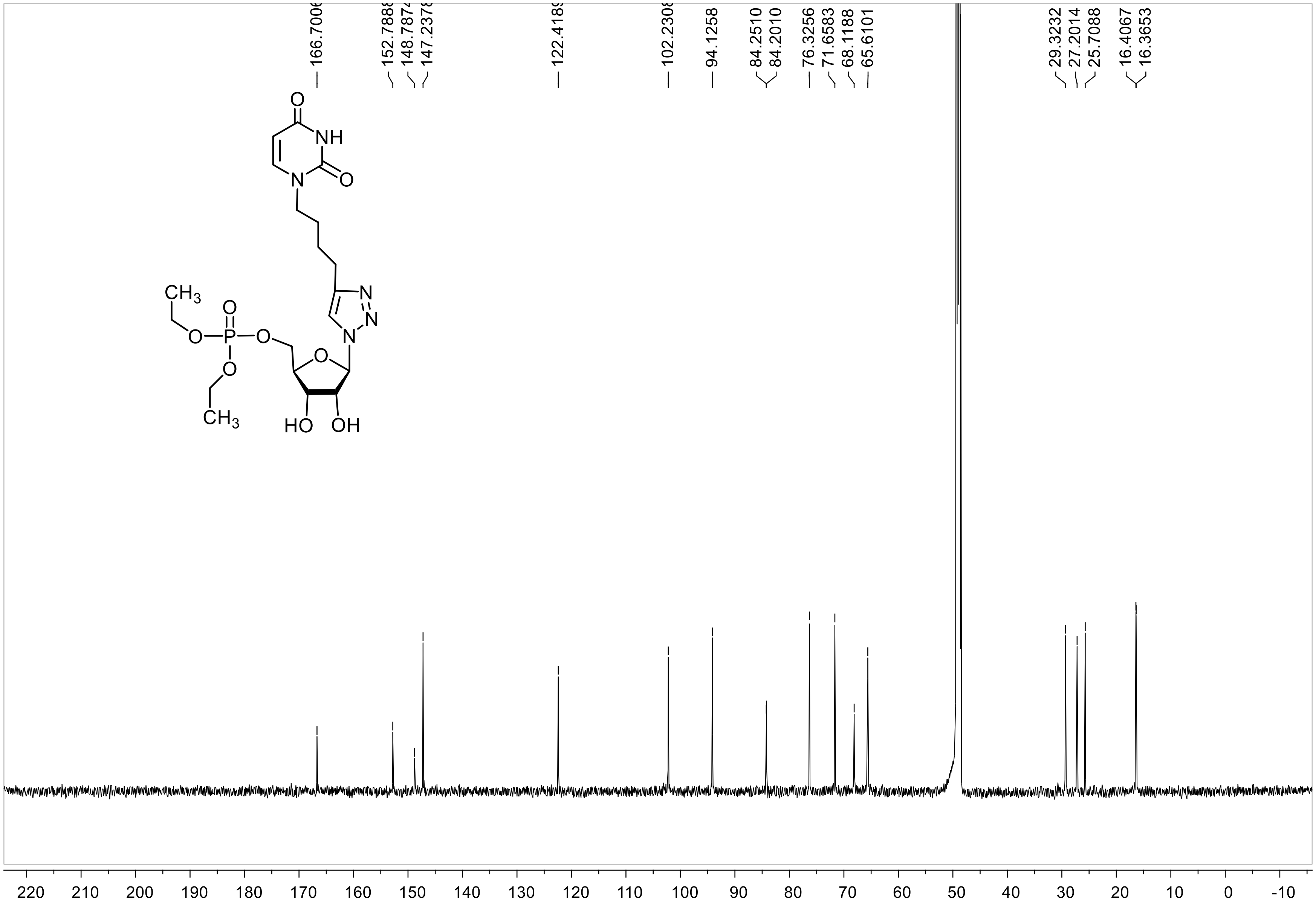

Figure S13. ^{13}C NMR spectrum of **12a** in CD_3OD

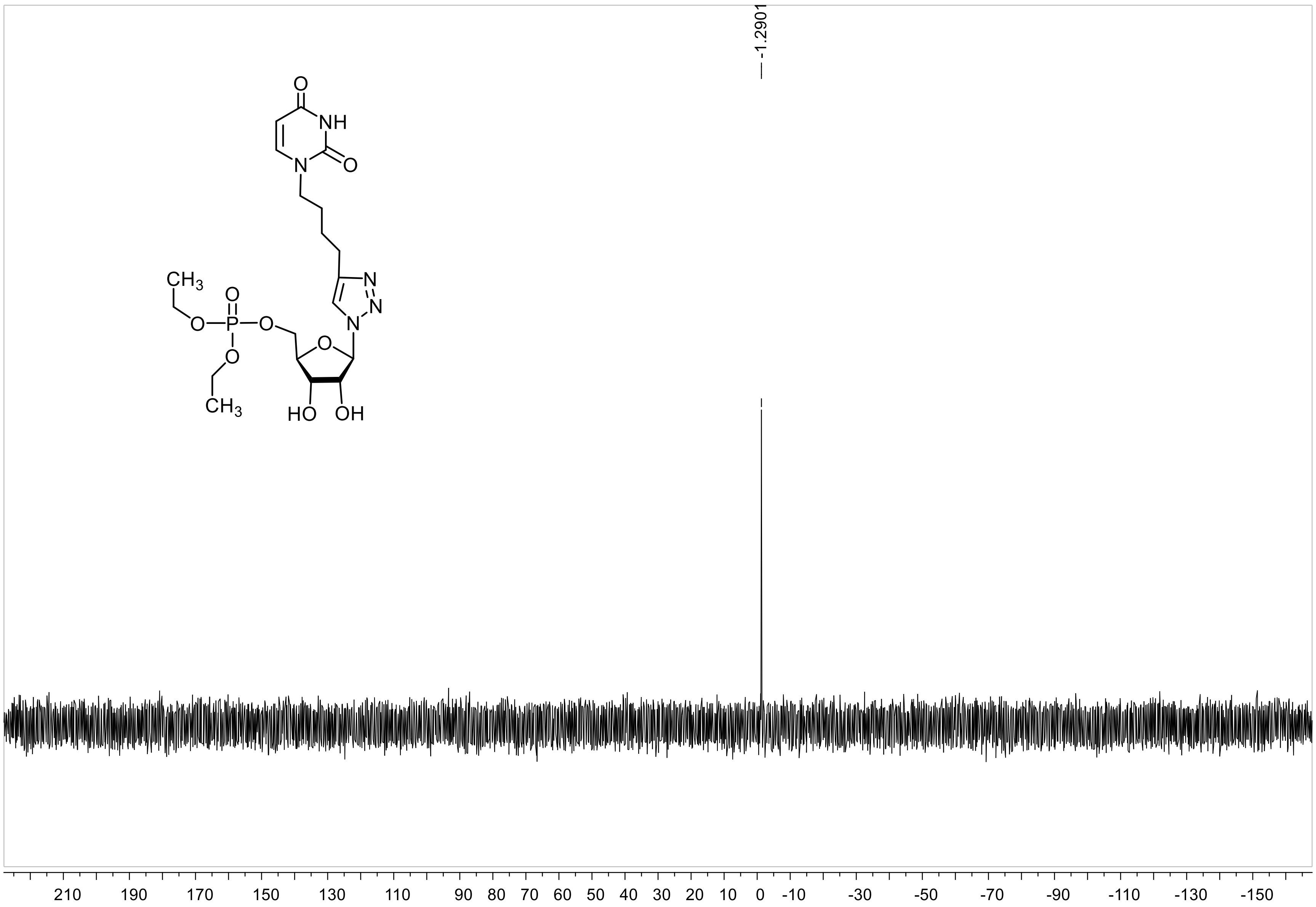

Figure S14. ^{31}P NMR spectrum of **12a** in CD_3OD

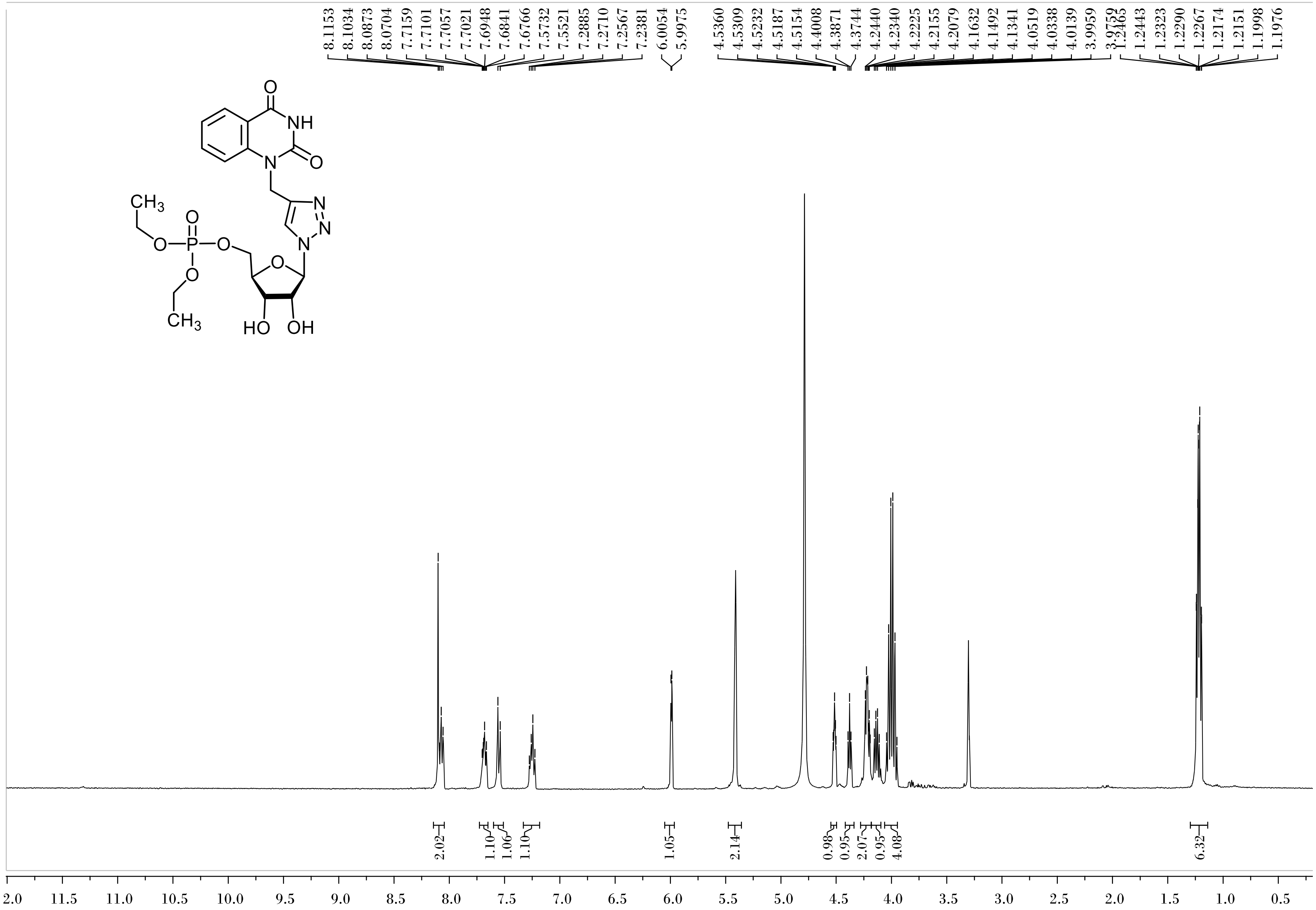

Figure S15. ¹H NMR spectrum of **11b** in CD_3OD

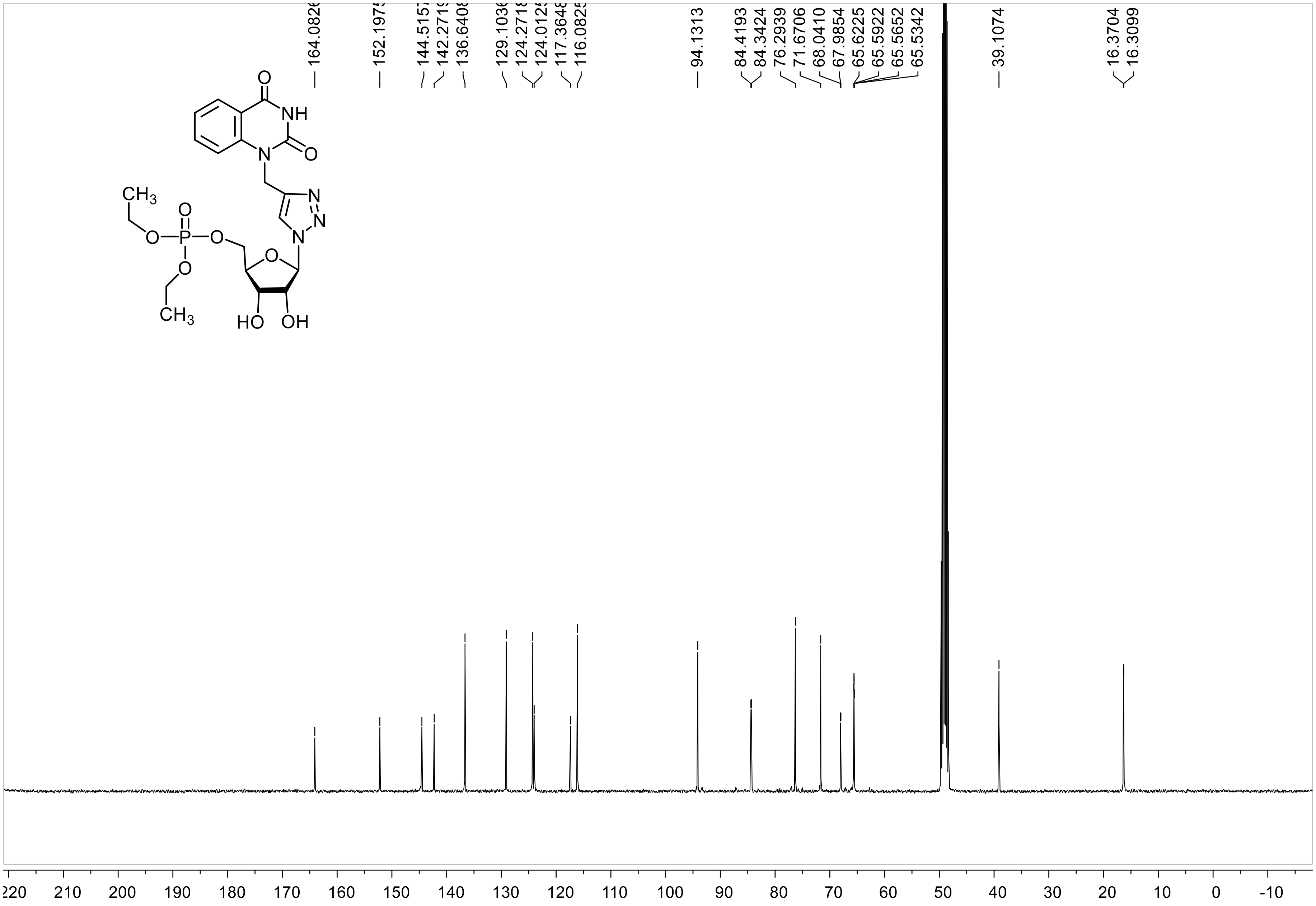

Figure S16. ^{13}C NMR spectrum of **11b** in CD_3OD

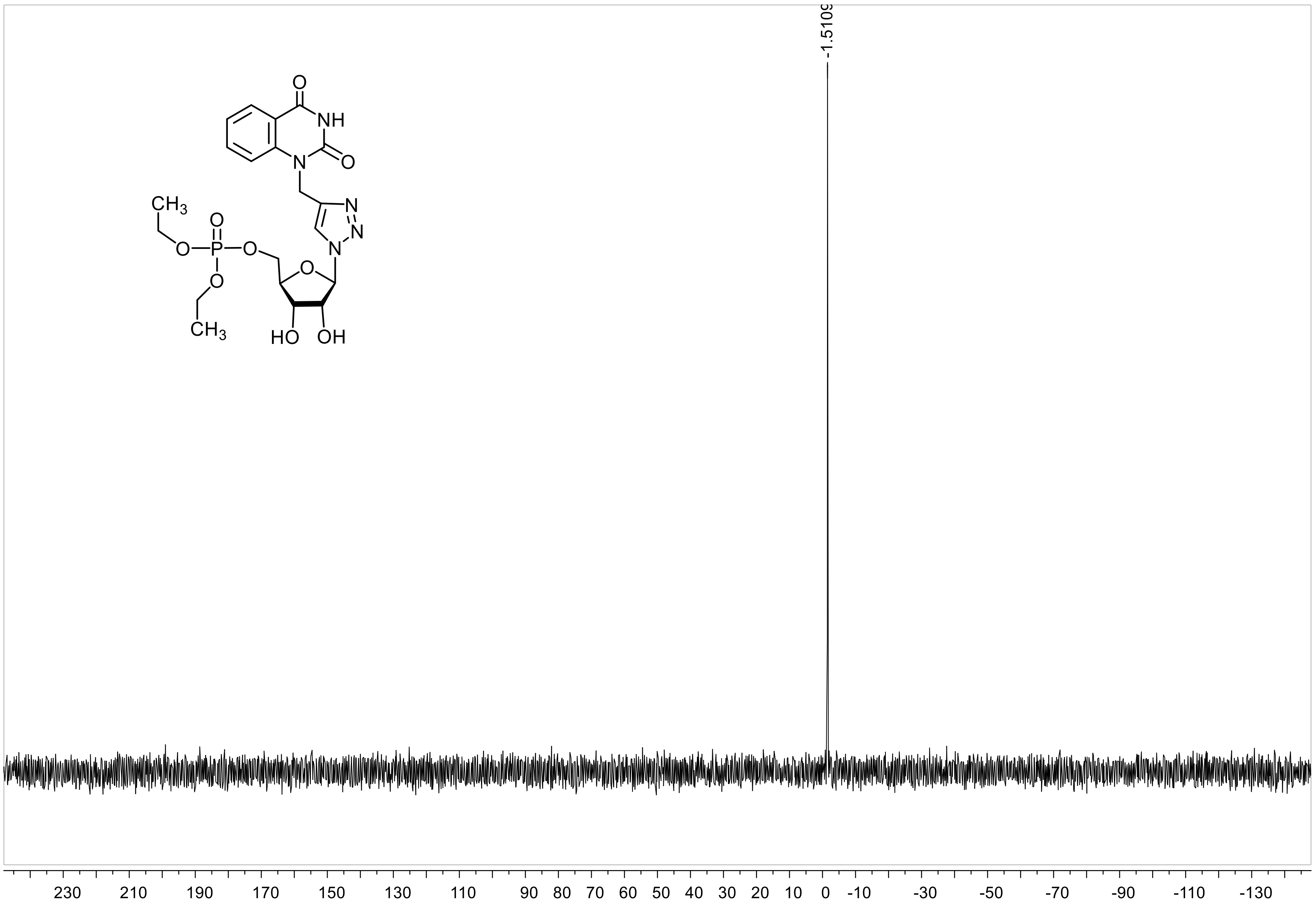

Figure S17. ^{31}P NMR spectrum of **11b** in CD_3OD

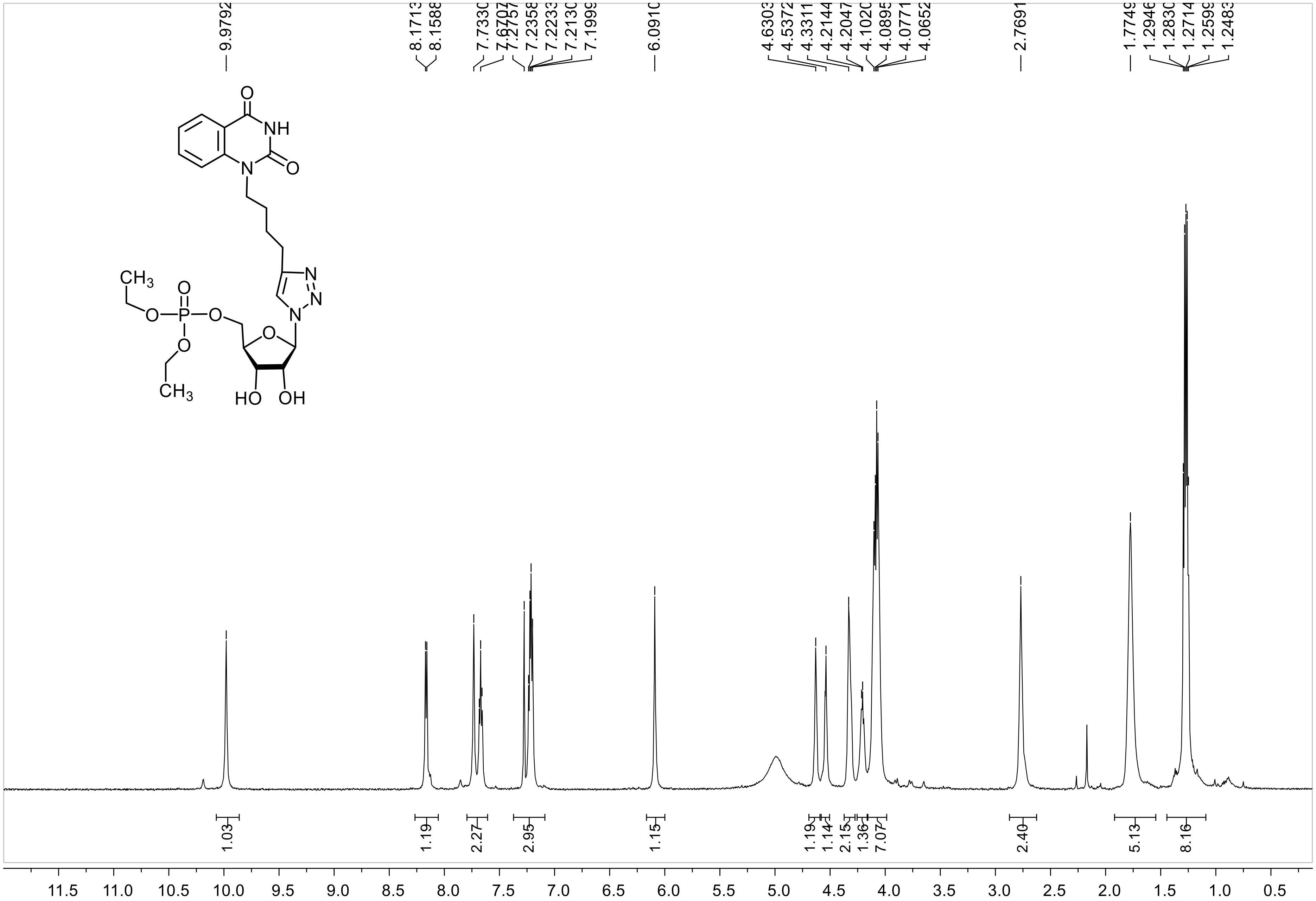

Figure S18. ^{1}H NMR spectrum of **12b** in CD_3OD

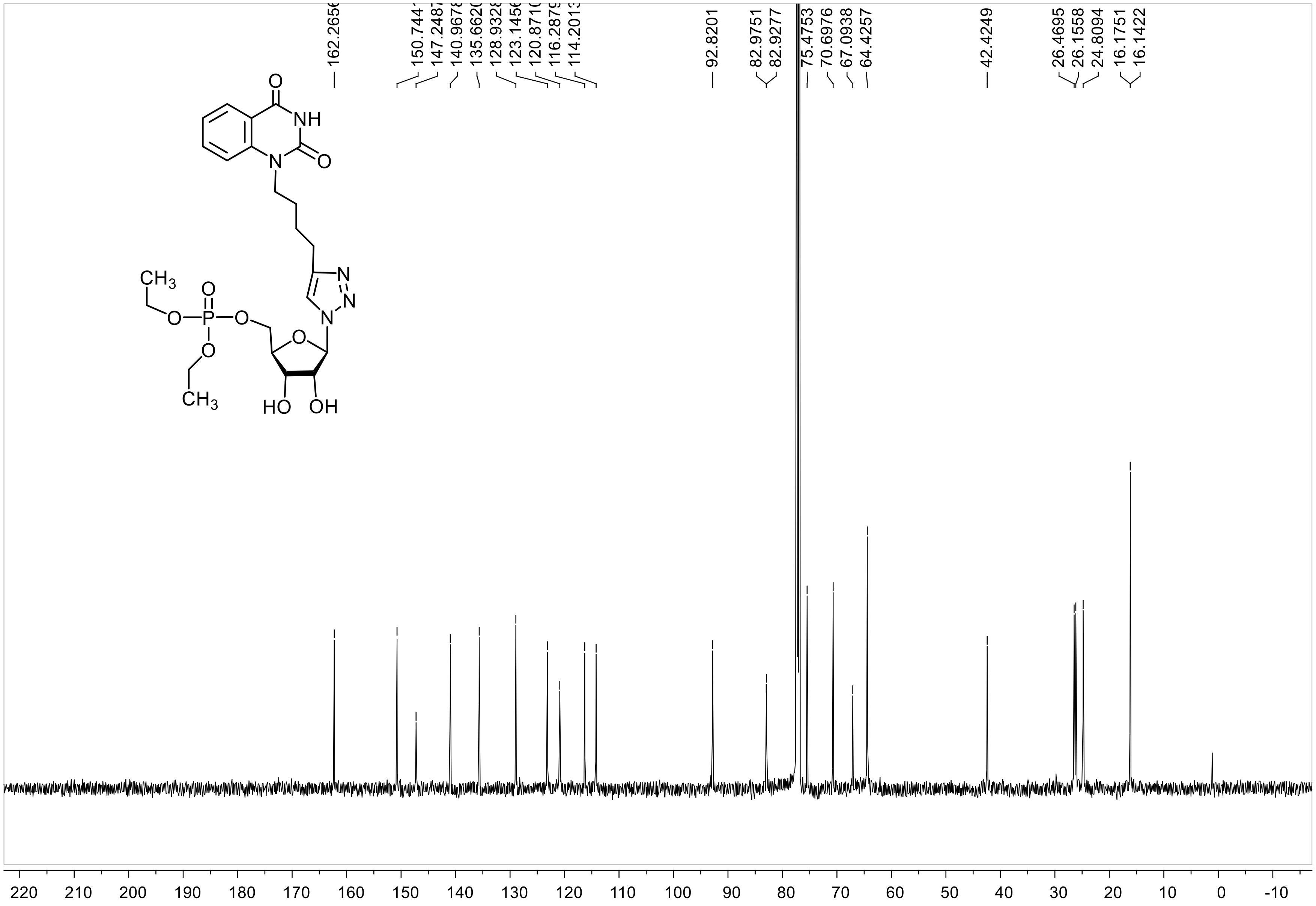

Figure S19. ^{13}C NMR spectrum of **12b** in CD_3OD

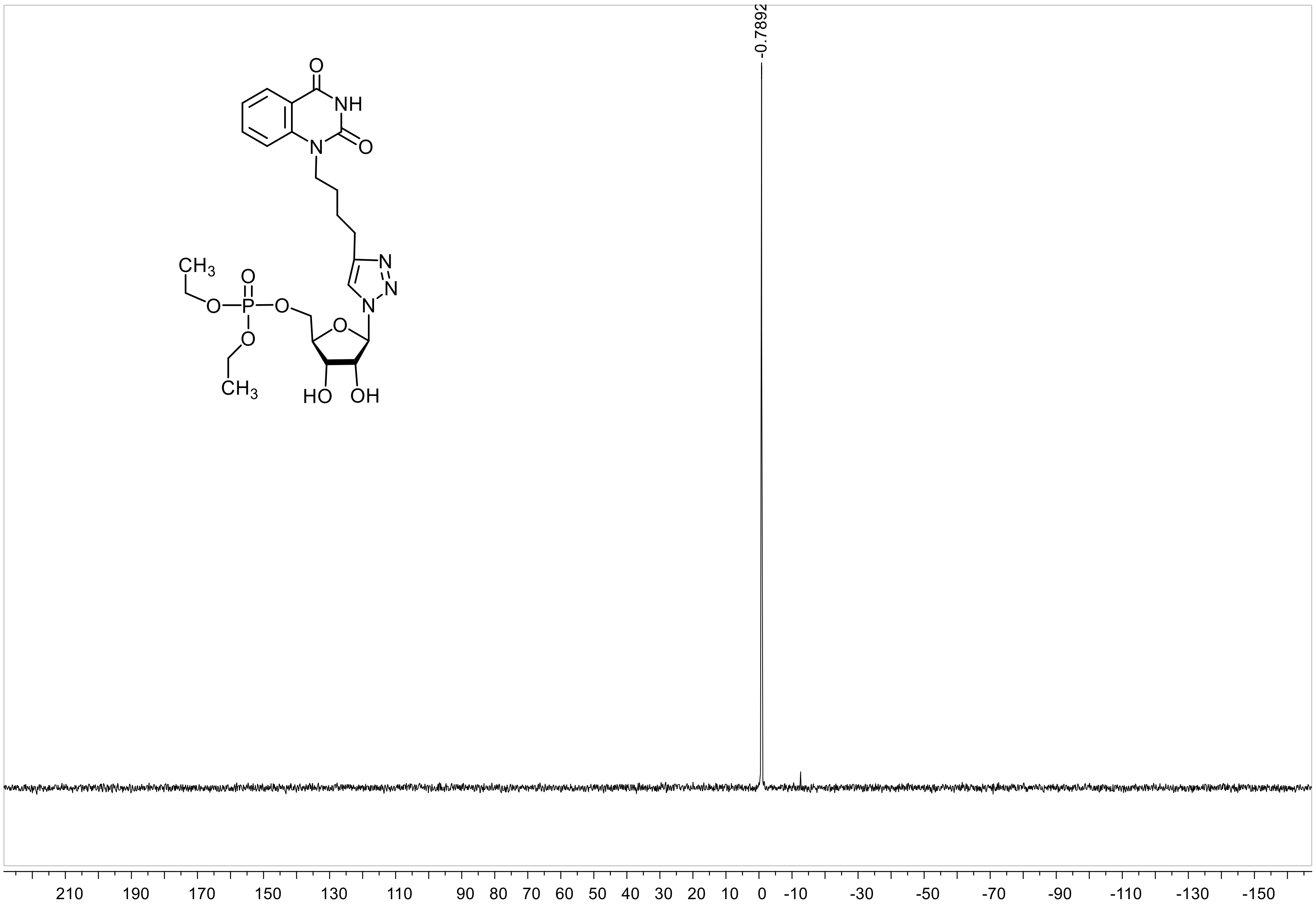

Figure S20. ^{31}P NMR spectrum of **12b** in CD_3OD

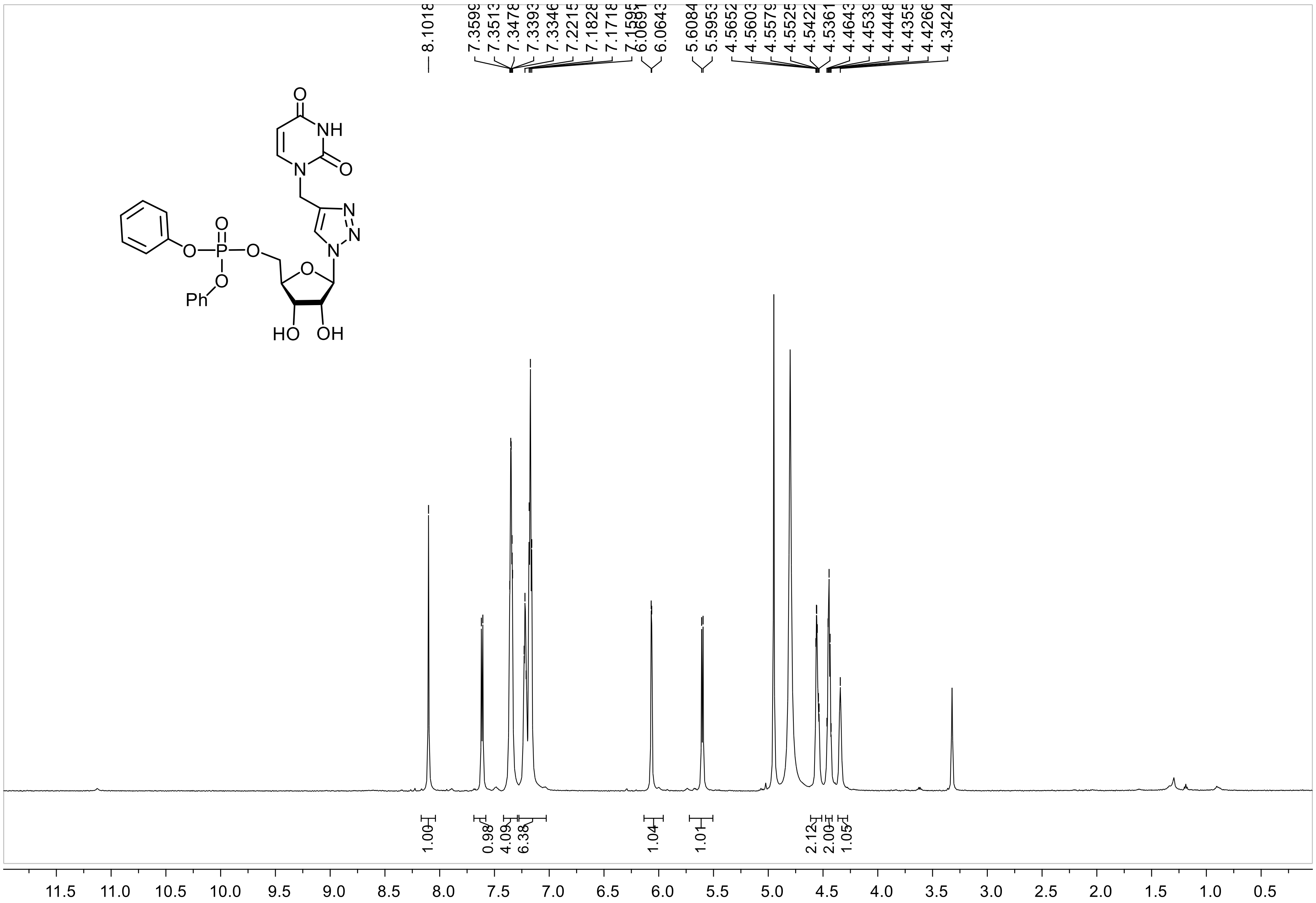

Figure S21. ^{1}H NMR spectrum of **13a** in CD_3OD

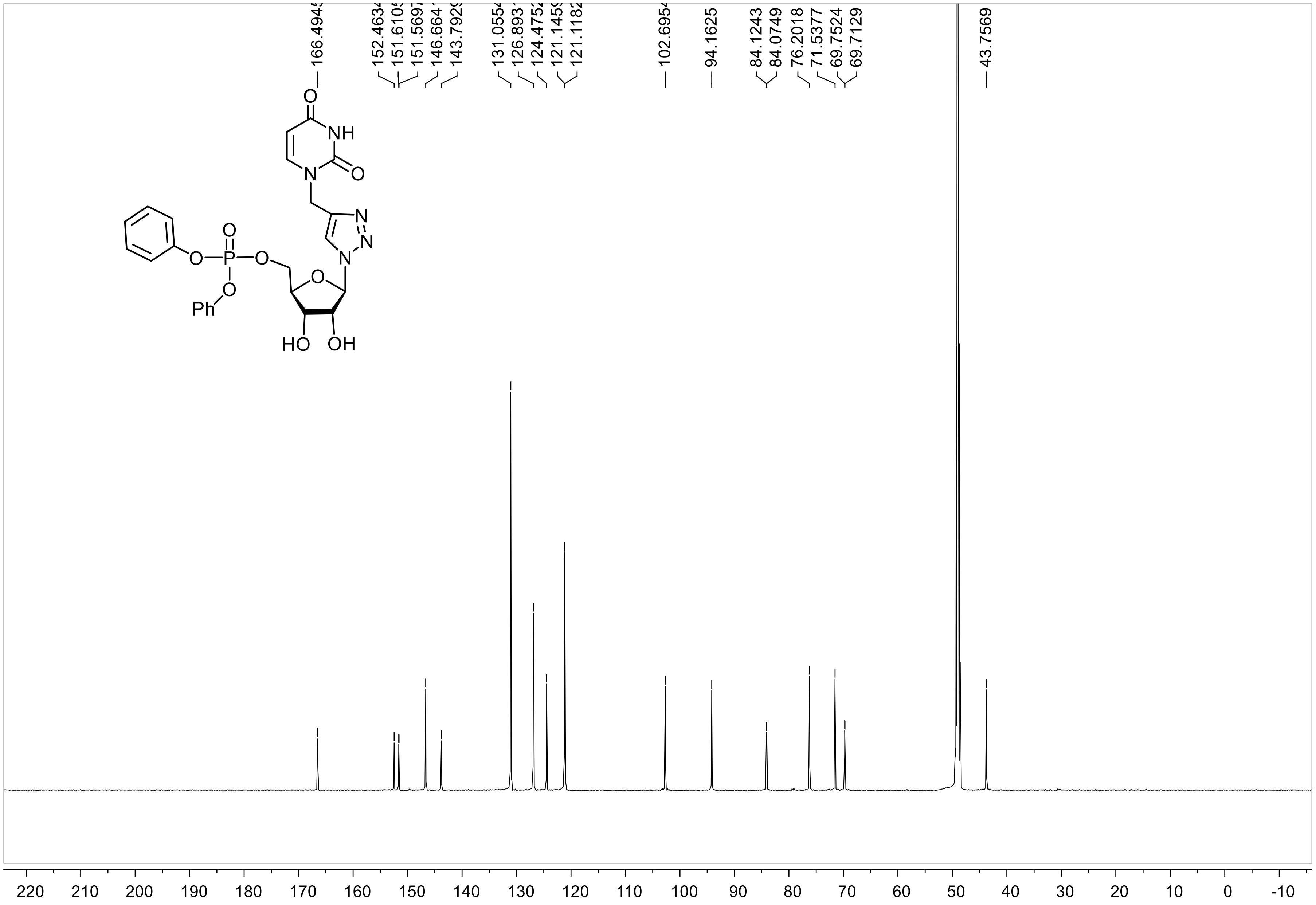

Figure S22. ^{13}C NMR spectrum of **13a** in CD_3OD

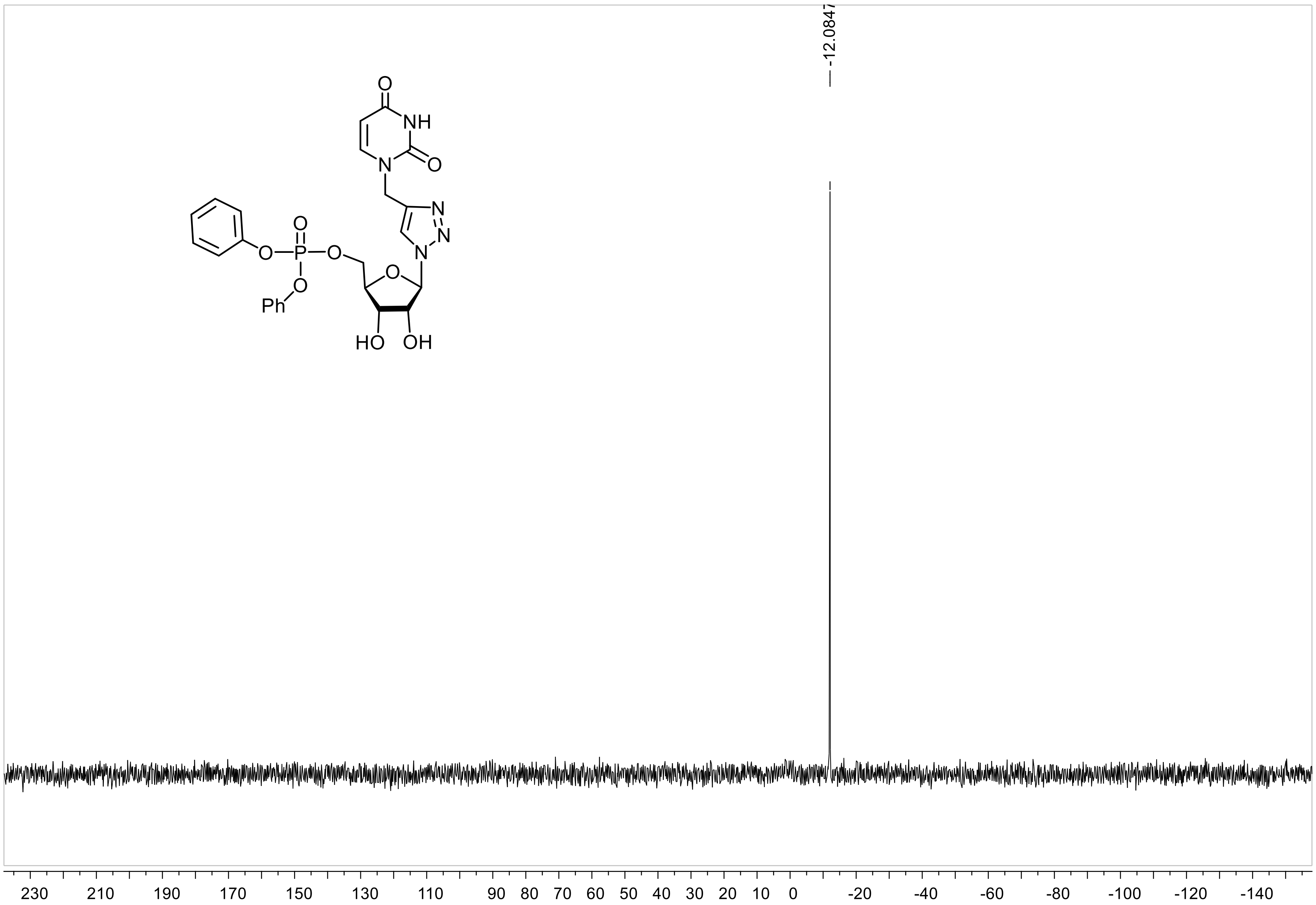

Figure S23. ^{31}P NMR spectrum of **13a** in CD_3OD

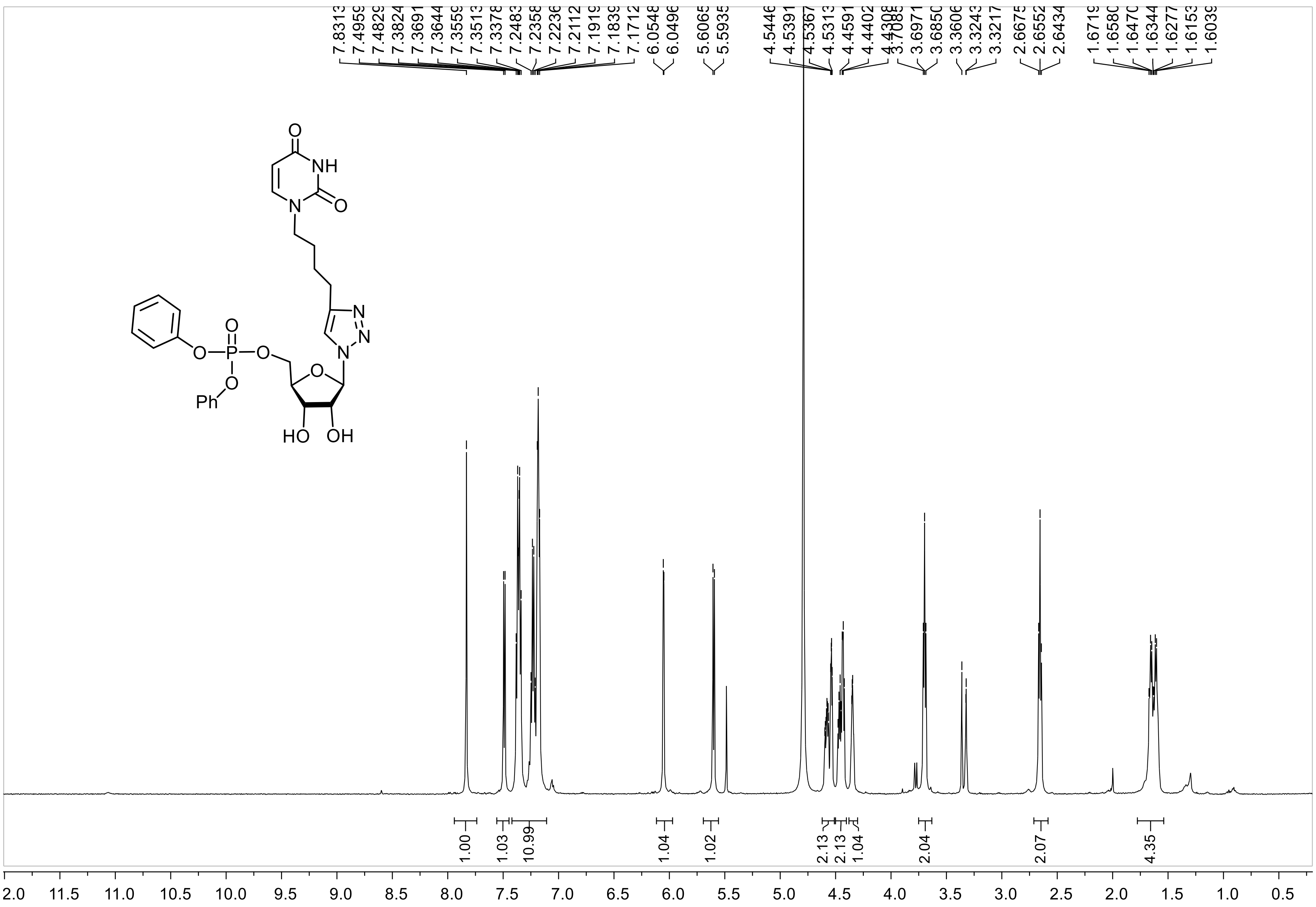

Figure S24. ^{1}H NMR spectrum of **14a** in CD_3OD

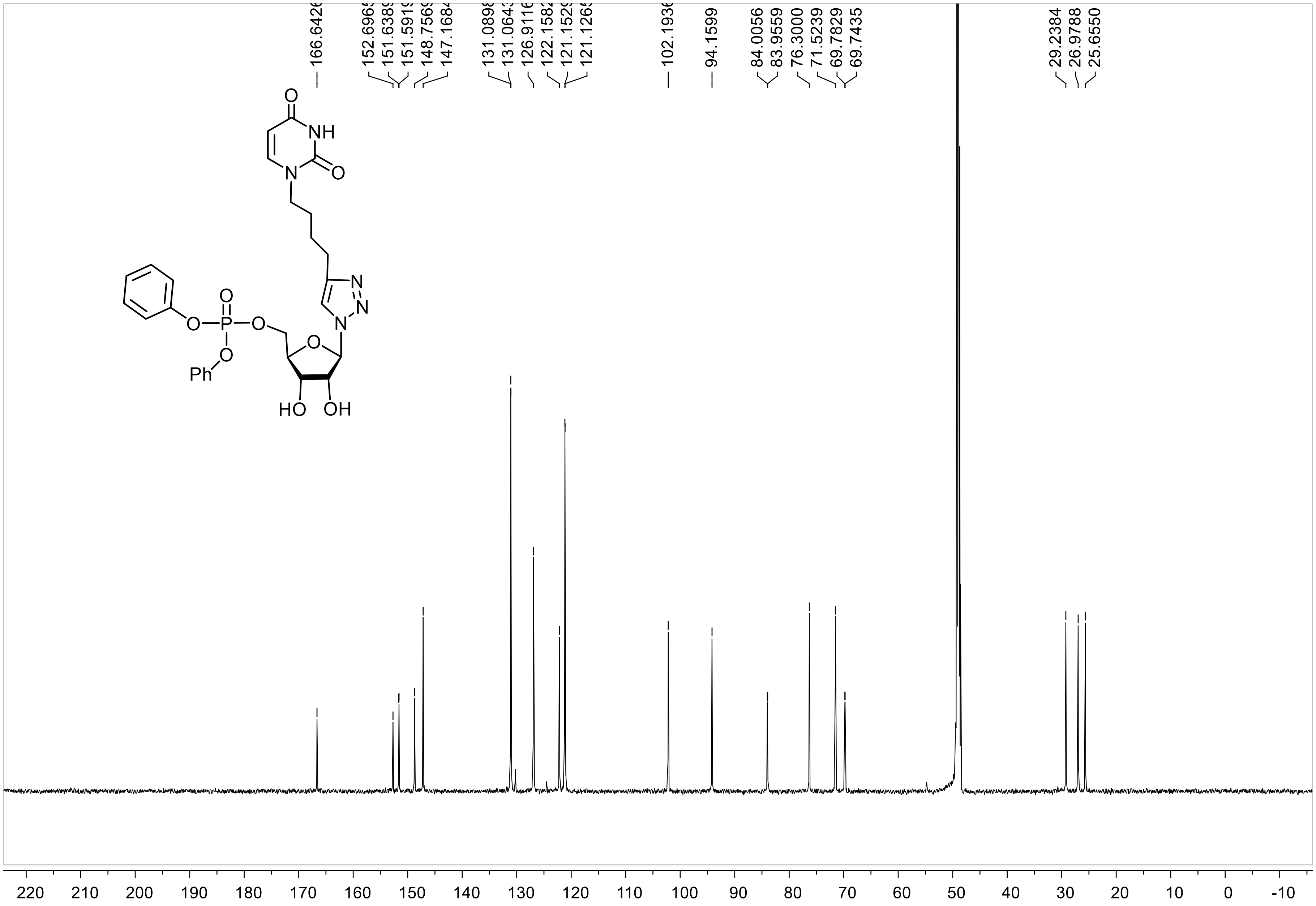

Figure S25. ^{13}C NMR spectrum of **14a** in CD_3OD

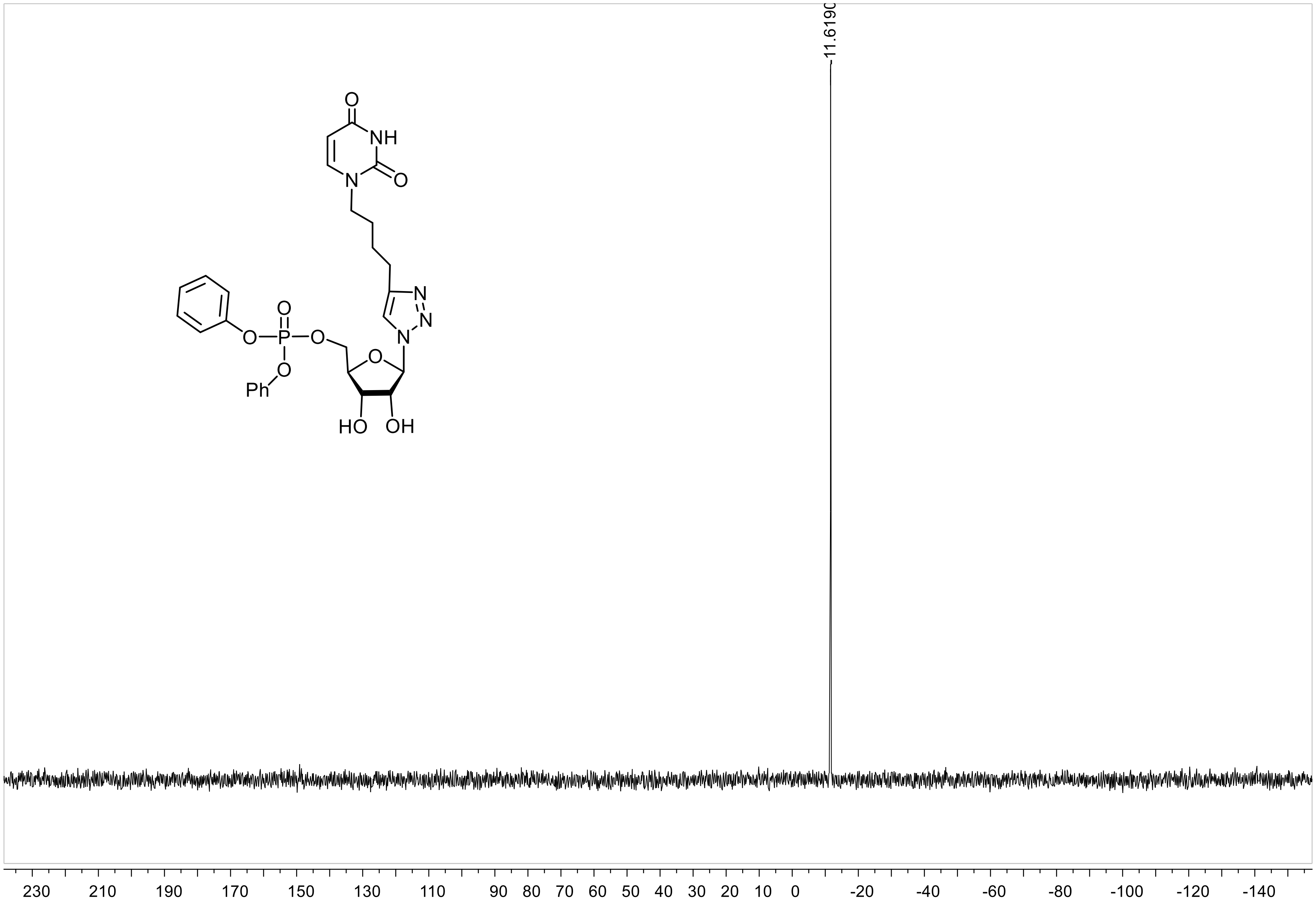

Figure S26. ^{31}P NMR spectrum of **14a** in CD_3OD

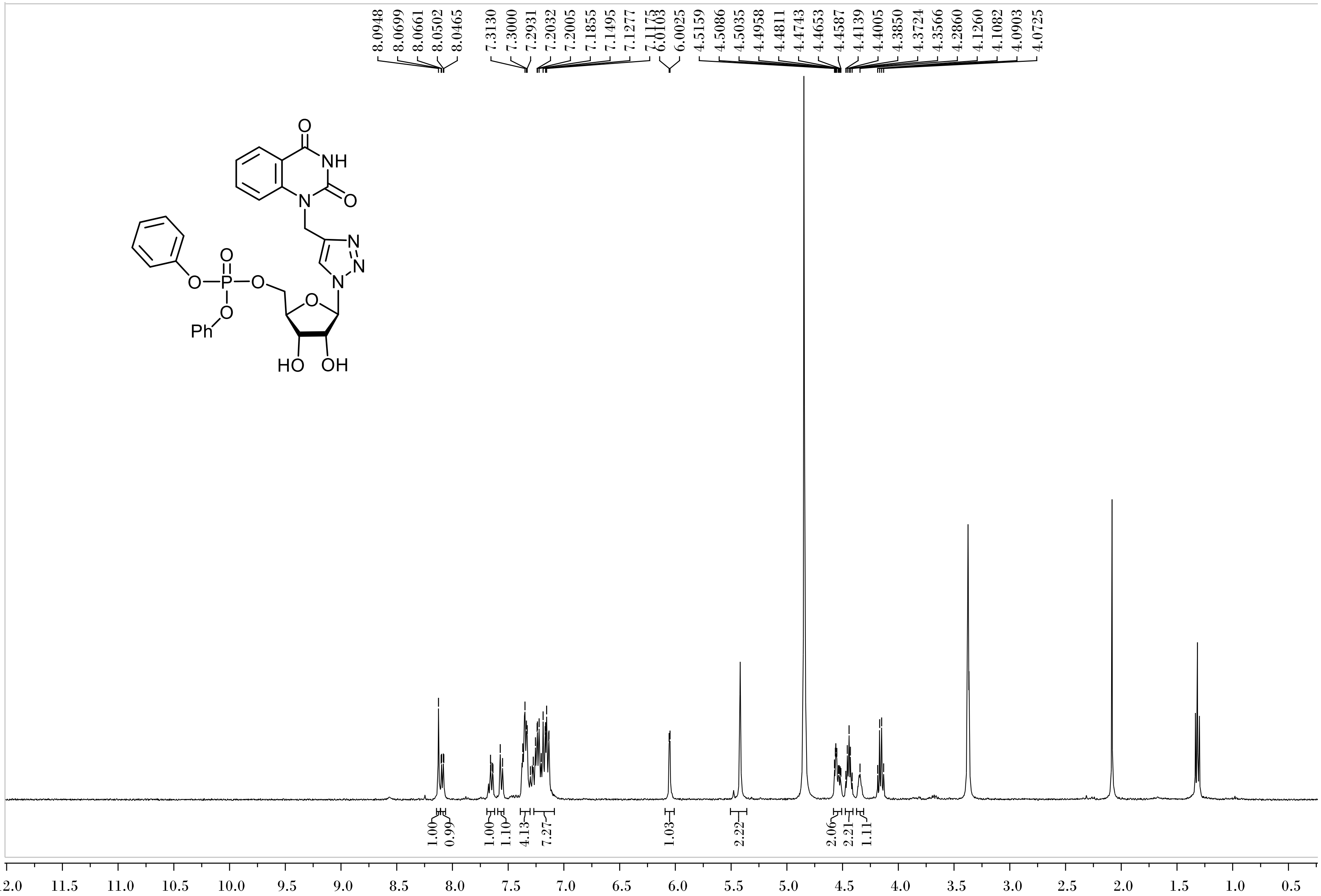

Figure S27. ^{1}H NMR spectrum of **13b** in CD_3OD

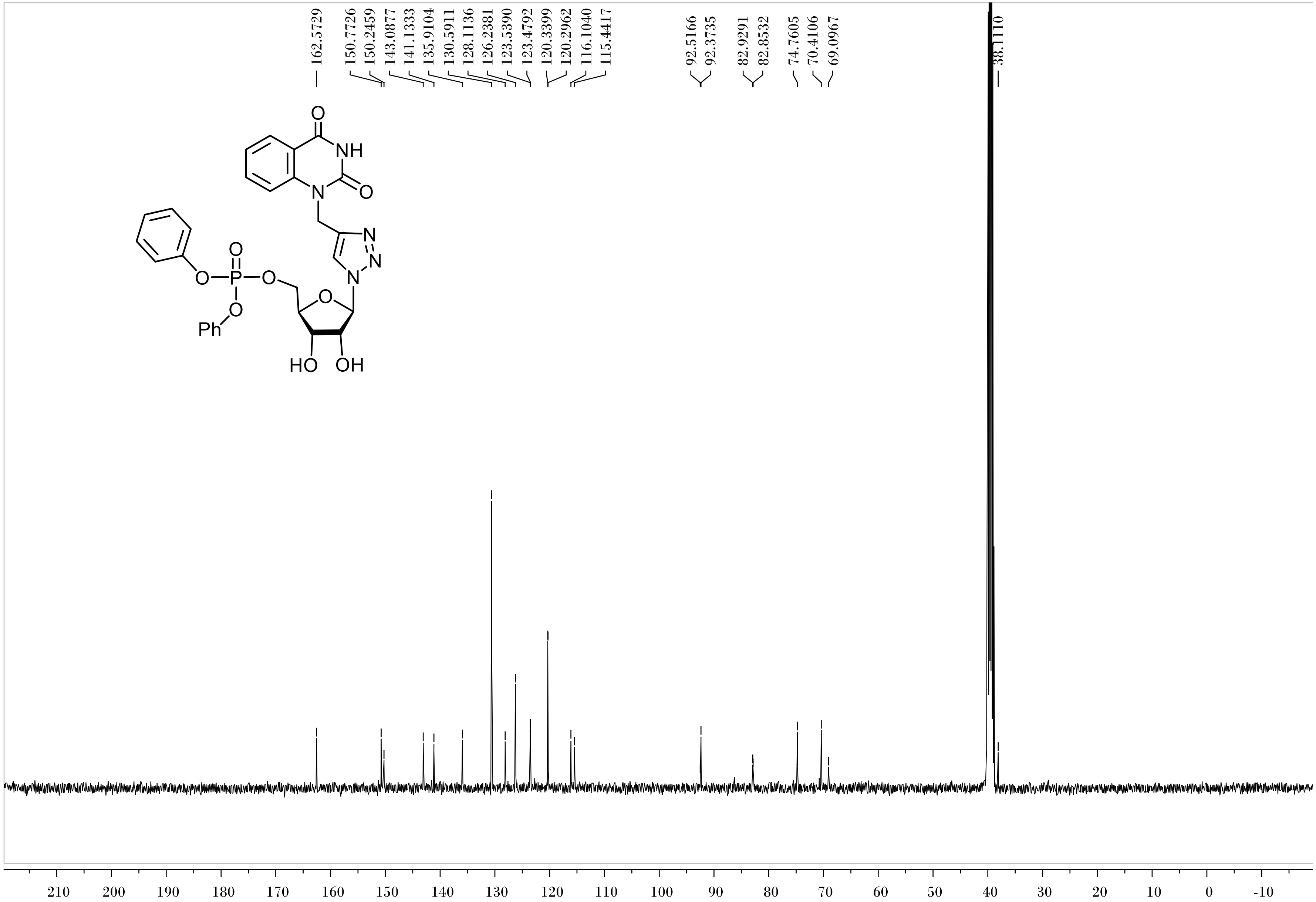

Figure S28. ^{13}C NMR spectrum of **13b** in CD_3OD

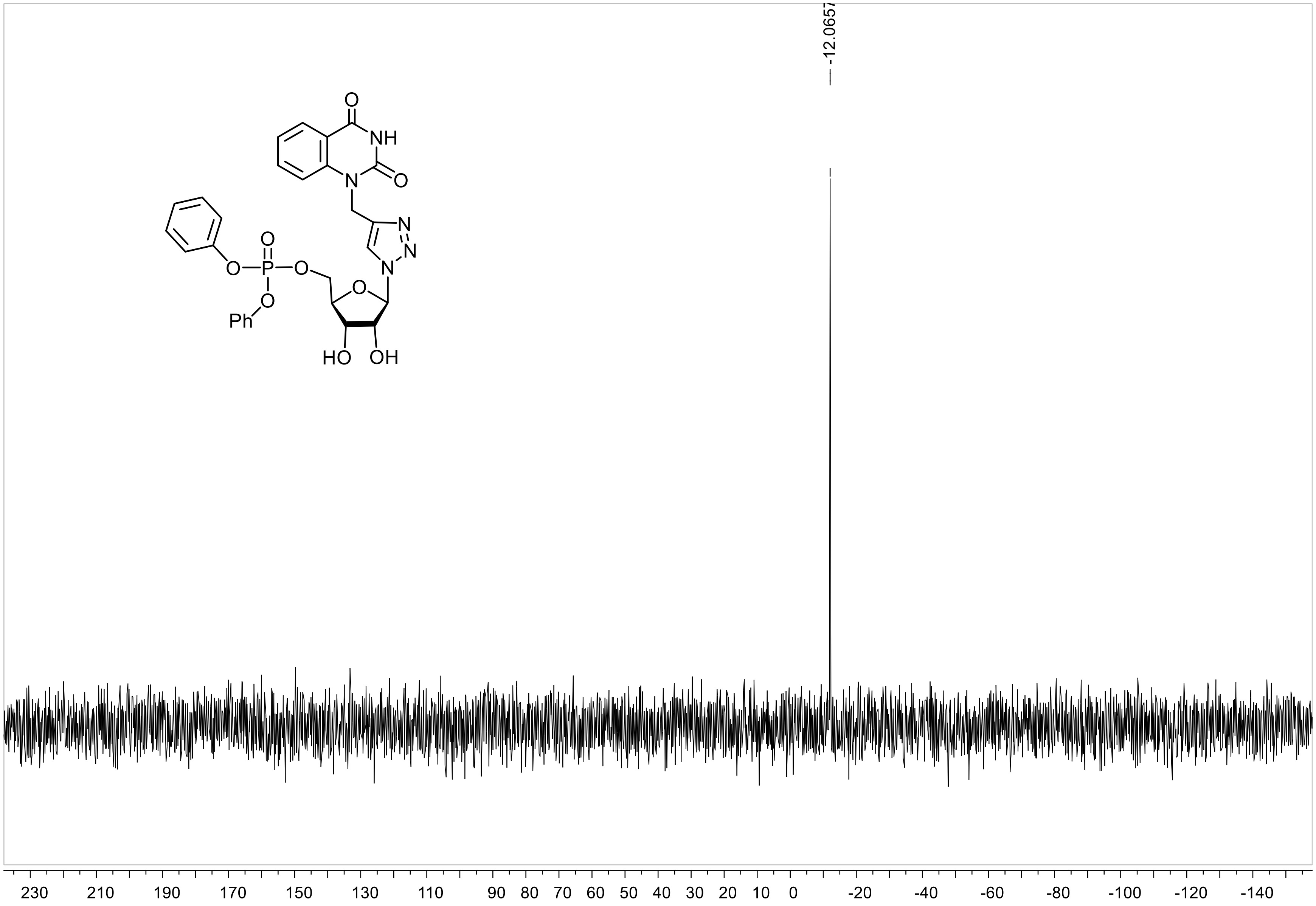

Figure S29. ^{31}P NMR spectrum of **13b** in CD_3OD

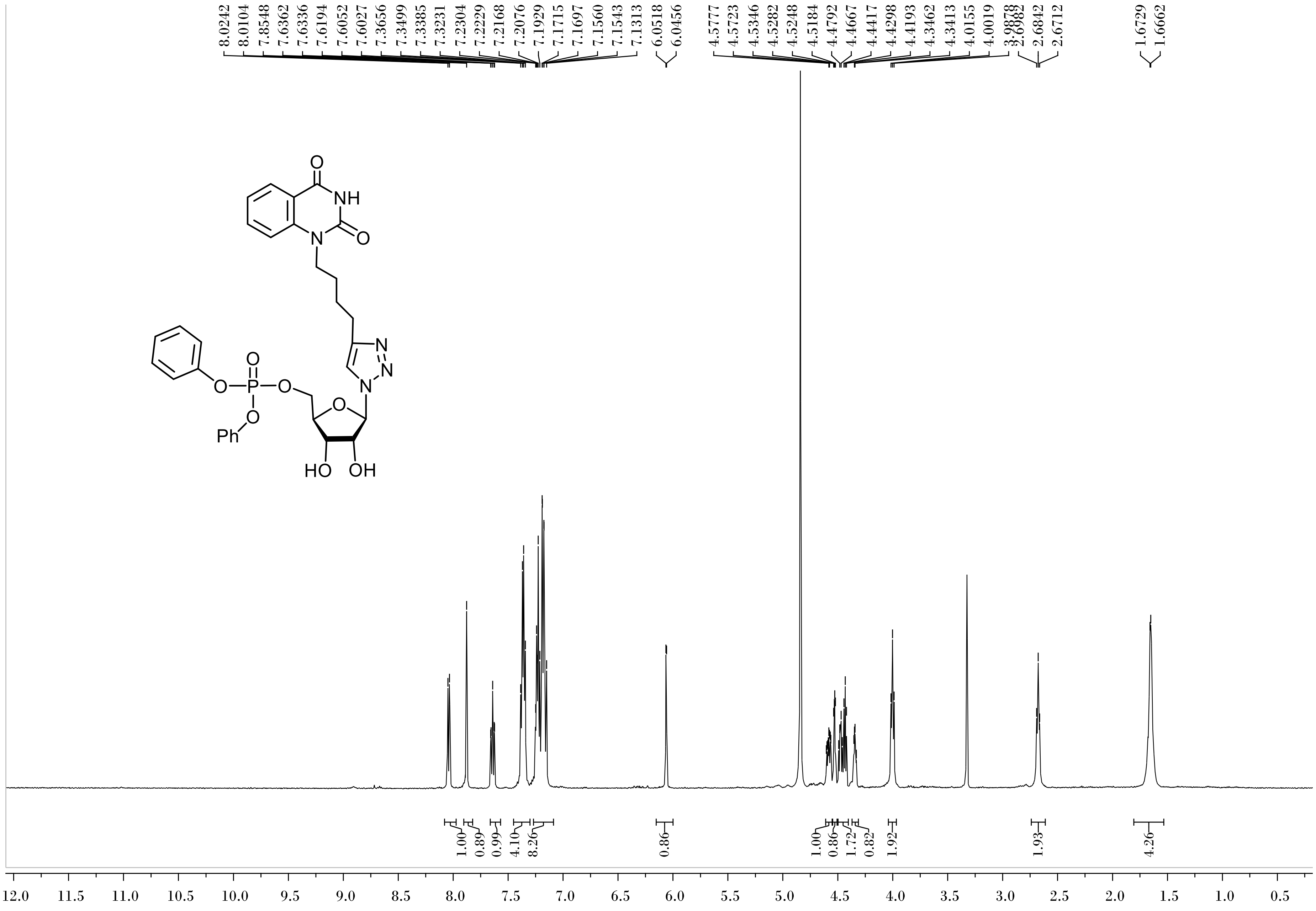

Figure S30. ^{1}H NMR spectrum of **14b** in CD_3OD

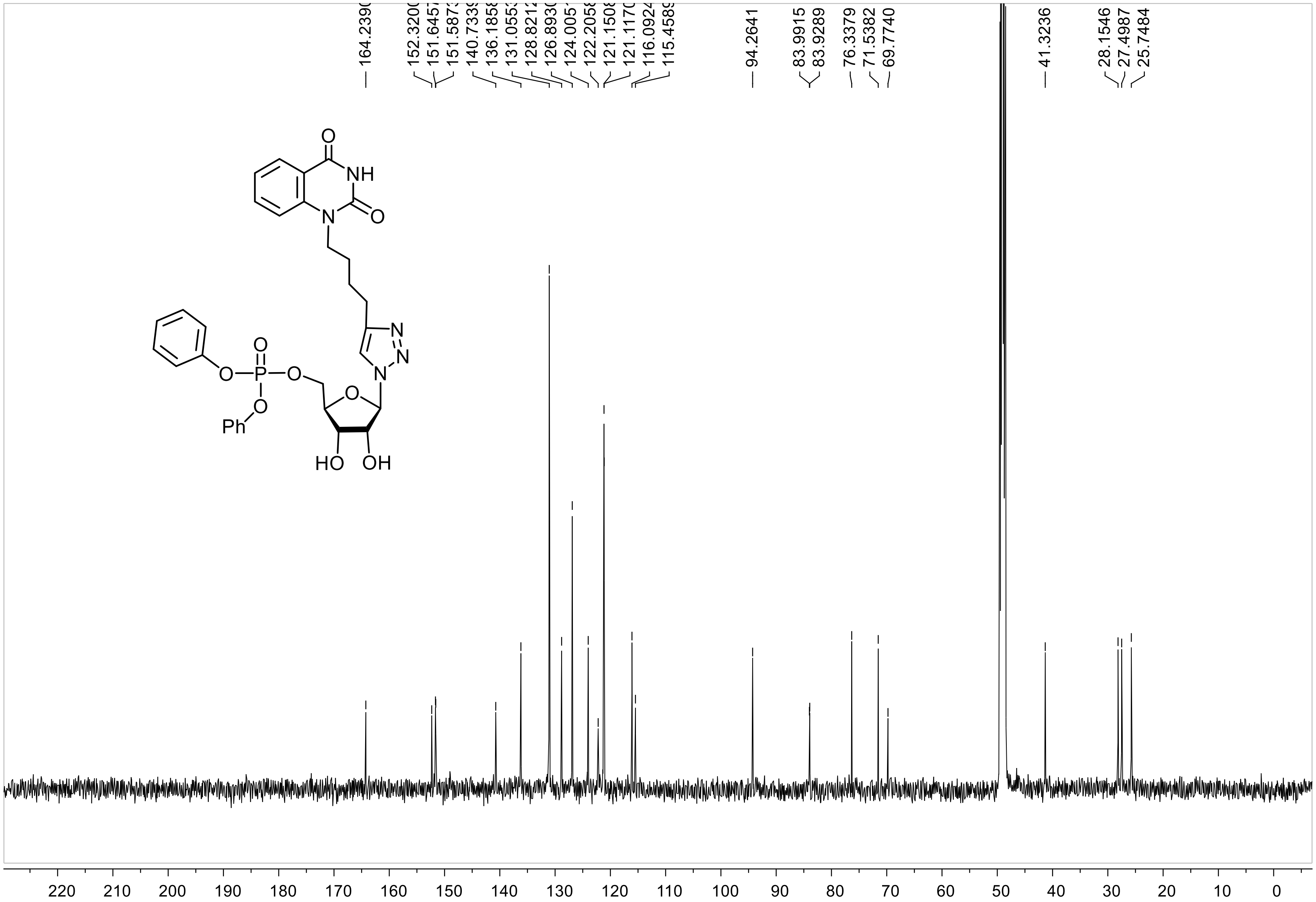

Figure S31. ^{13}C NMR spectrum of **14b** in CD_3OD

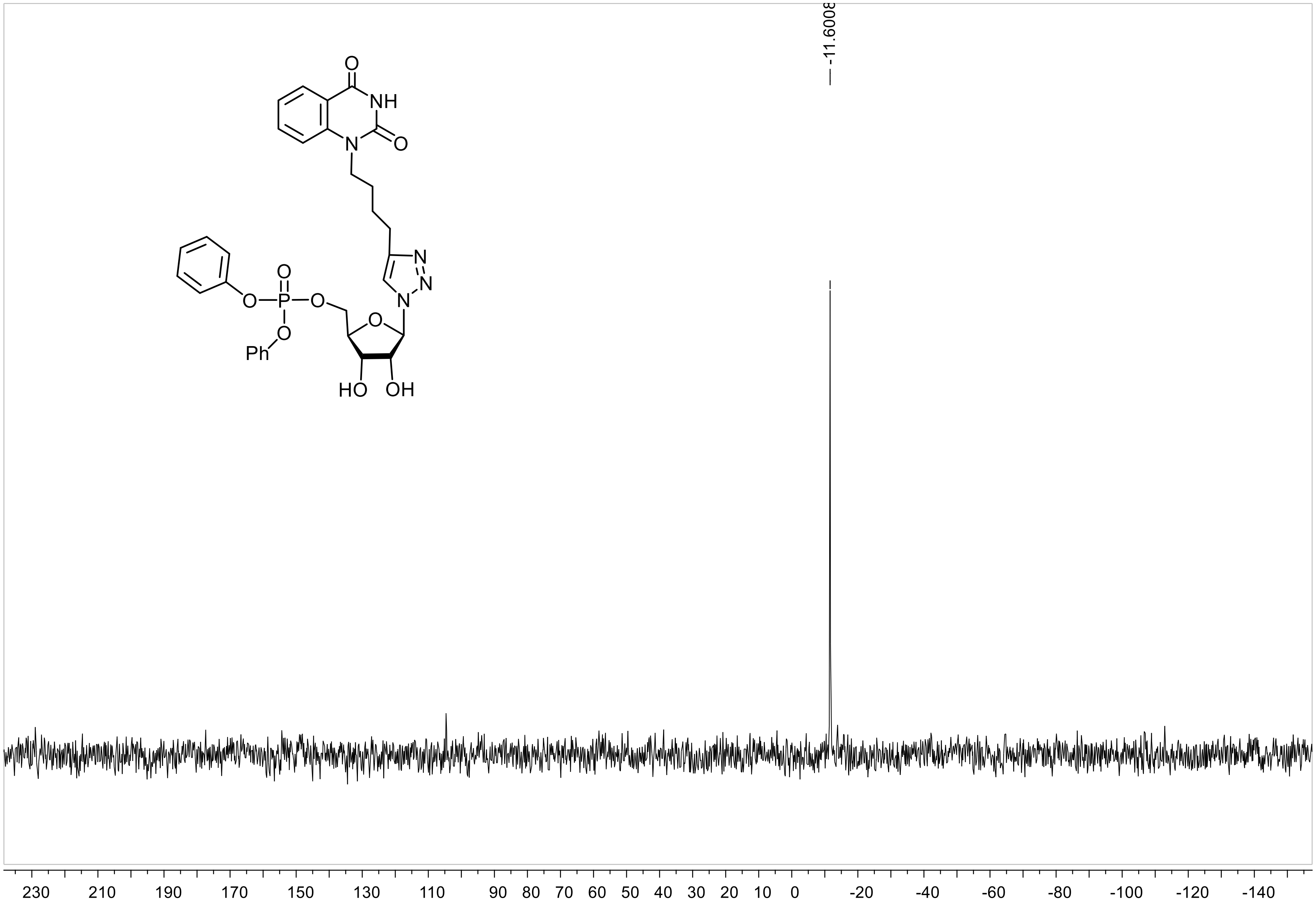

Figure S32. ^{31}P NMR spectrum of **14b** in CD_3OD

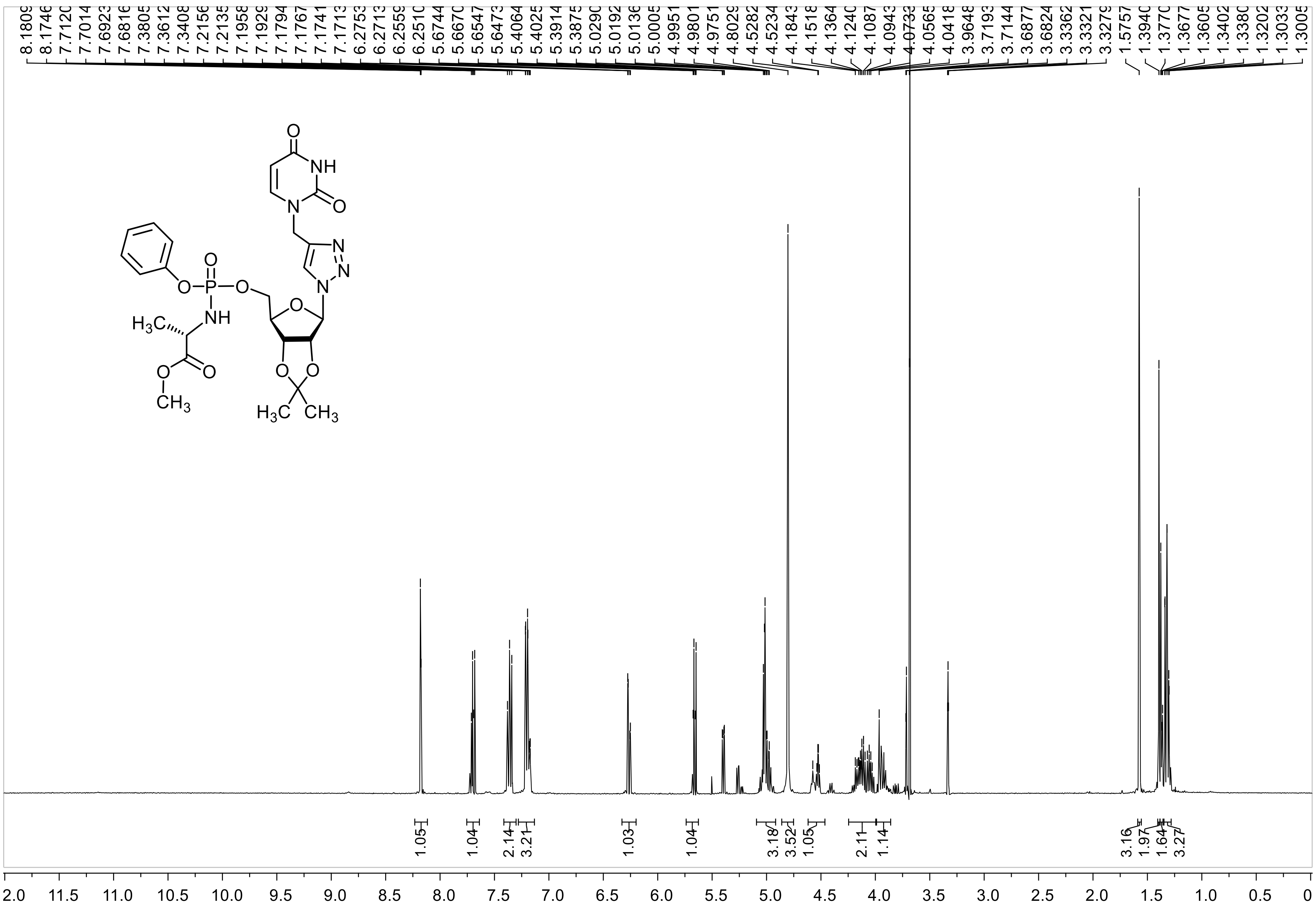

Figure S33. ^{1}H NMR spectrum of **15a** in CD_3OD

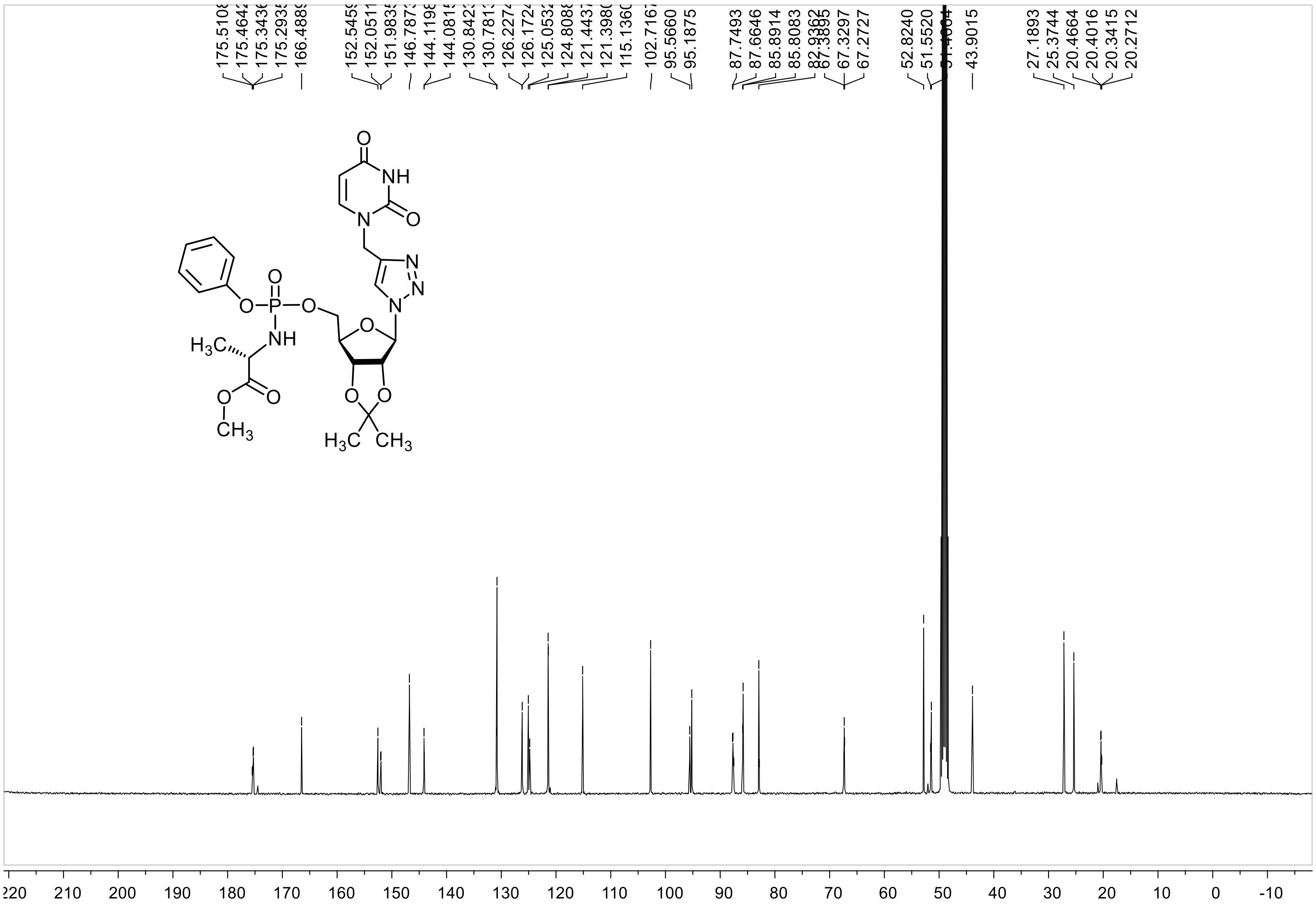

Figure S34. ^{13}C NMR NMR spectrum of **15a** in CD_3OD

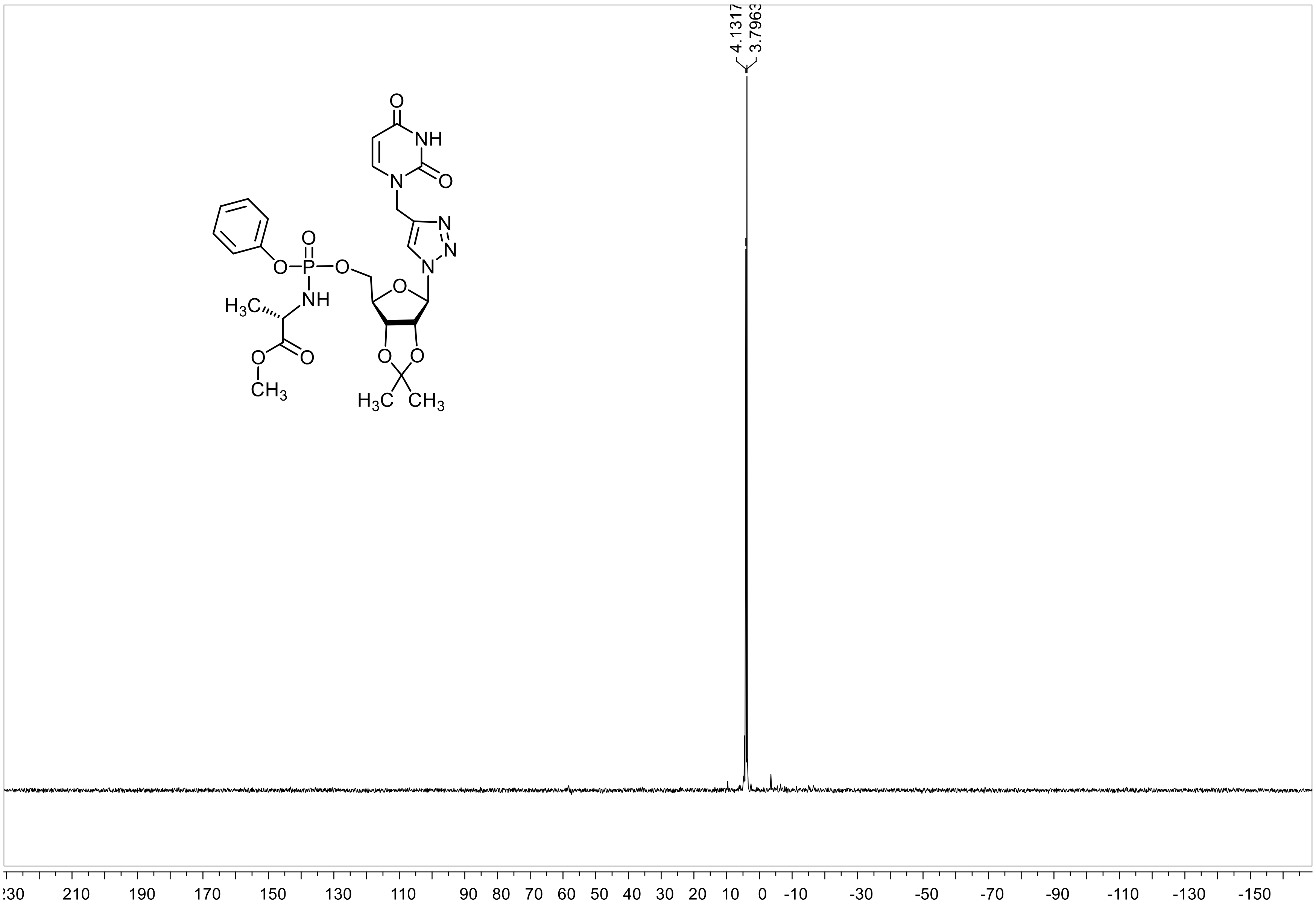

Figure S35. ^{31}P NMR NMR spectrum of **15a** in CD_3OD

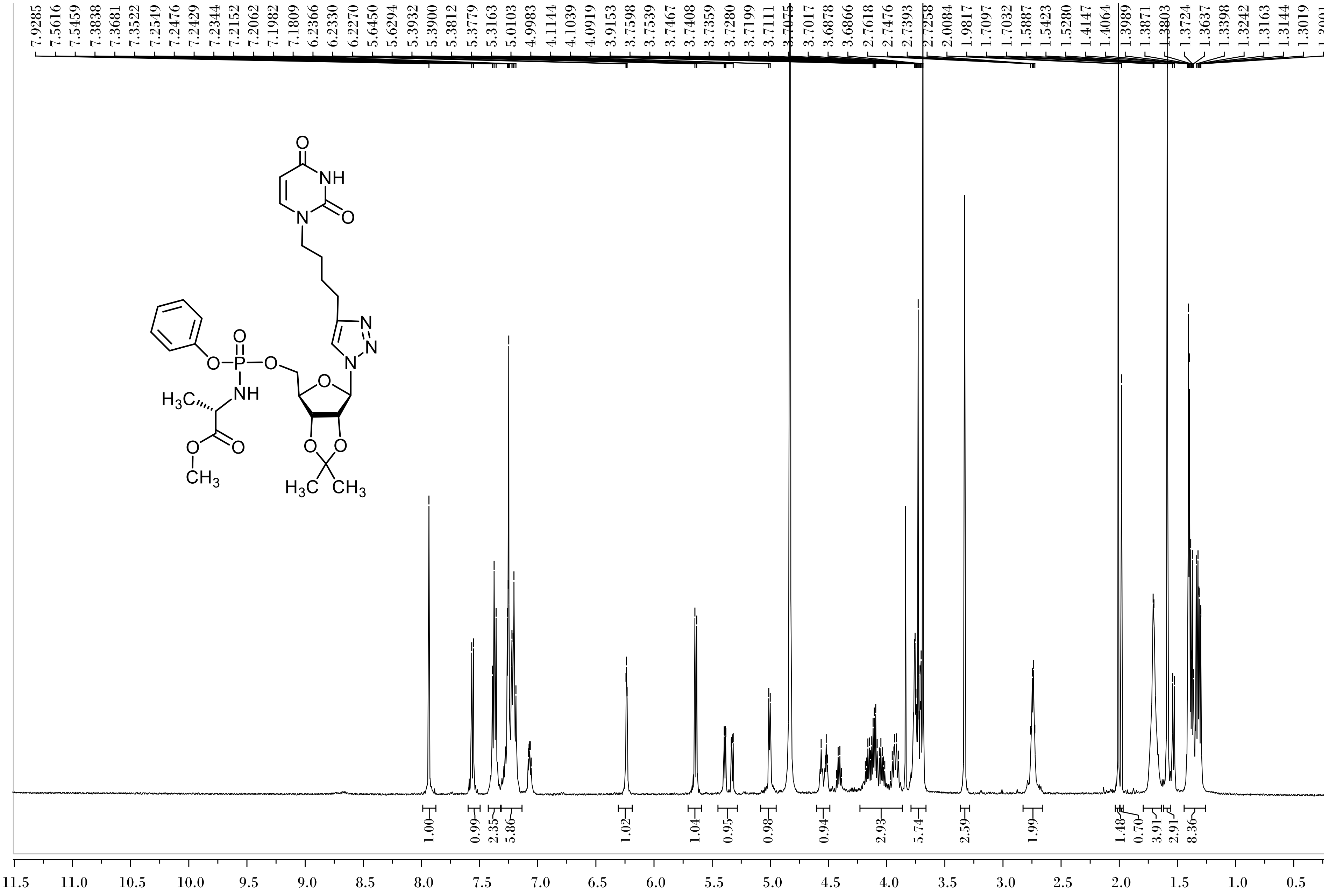

Figure S36. ^{1}H NMR NMR spectrum of **16a** in CD_3OD

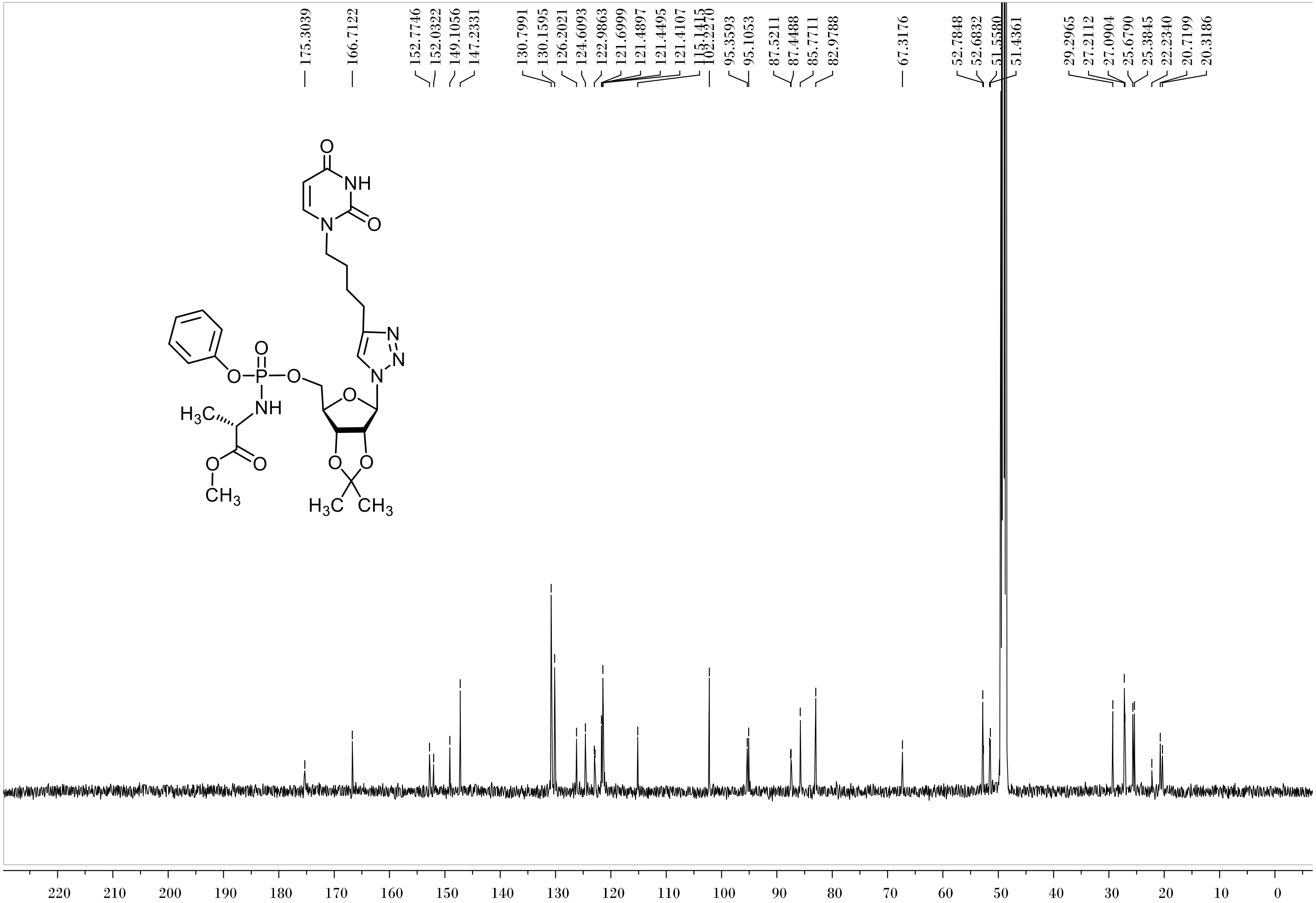

Figure S37. ^{13}C NMR spectrum of **16a** in CD_3OD

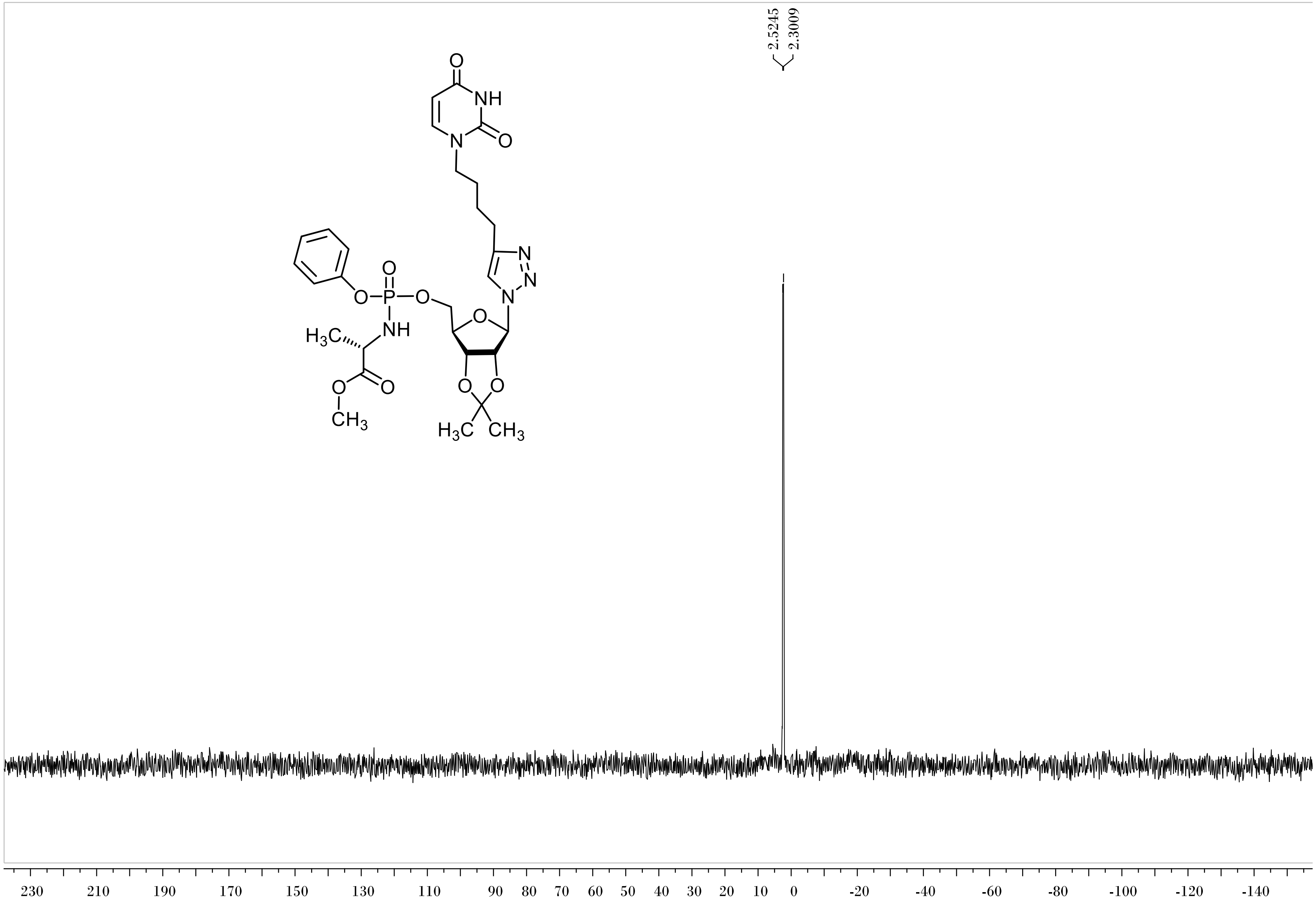

Figure S38. ^{31}P NMR spectrum of **16a** in CD_3OD

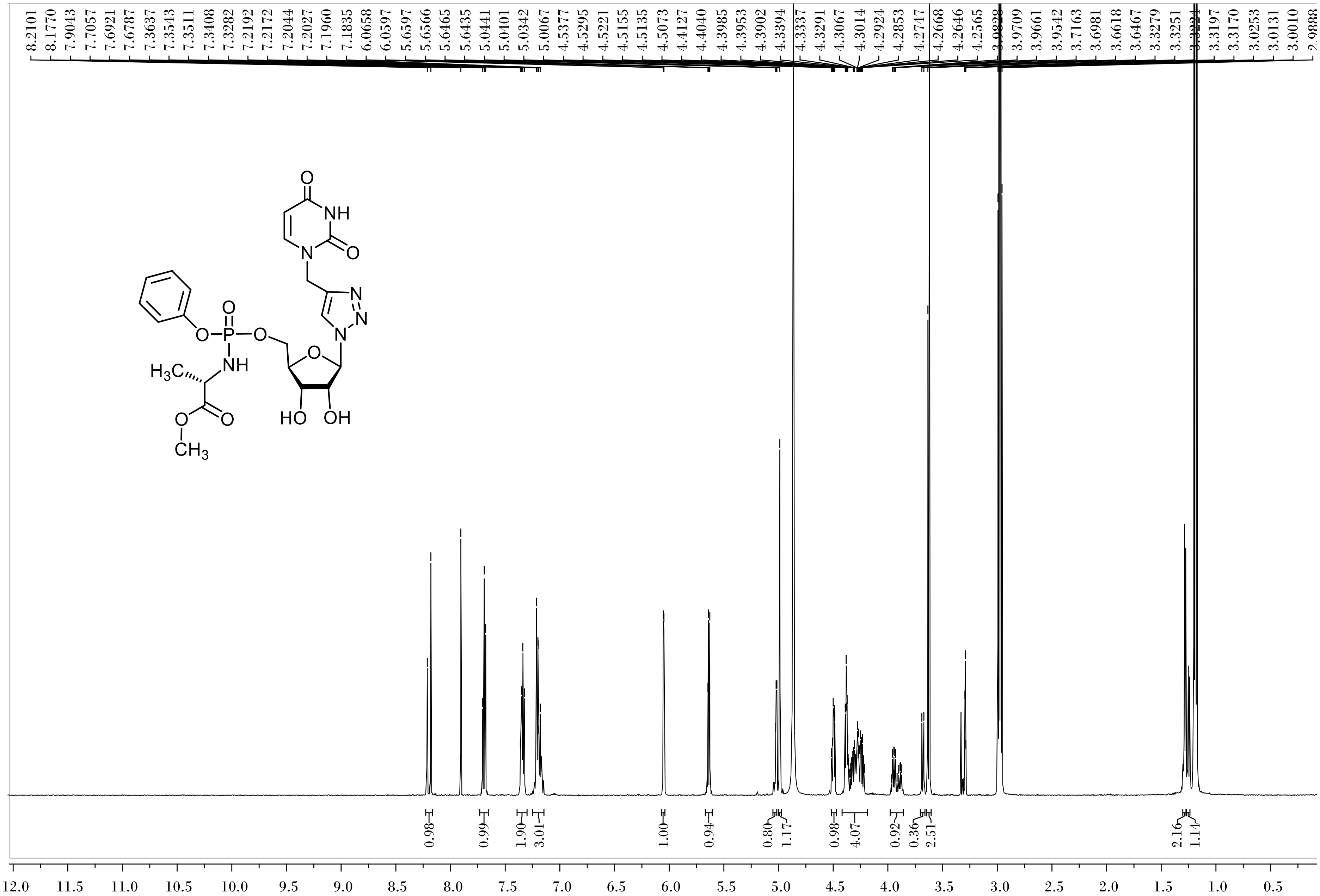

Figure S39. ^{1}H NMR spectrum of **17a** in CD_3OD

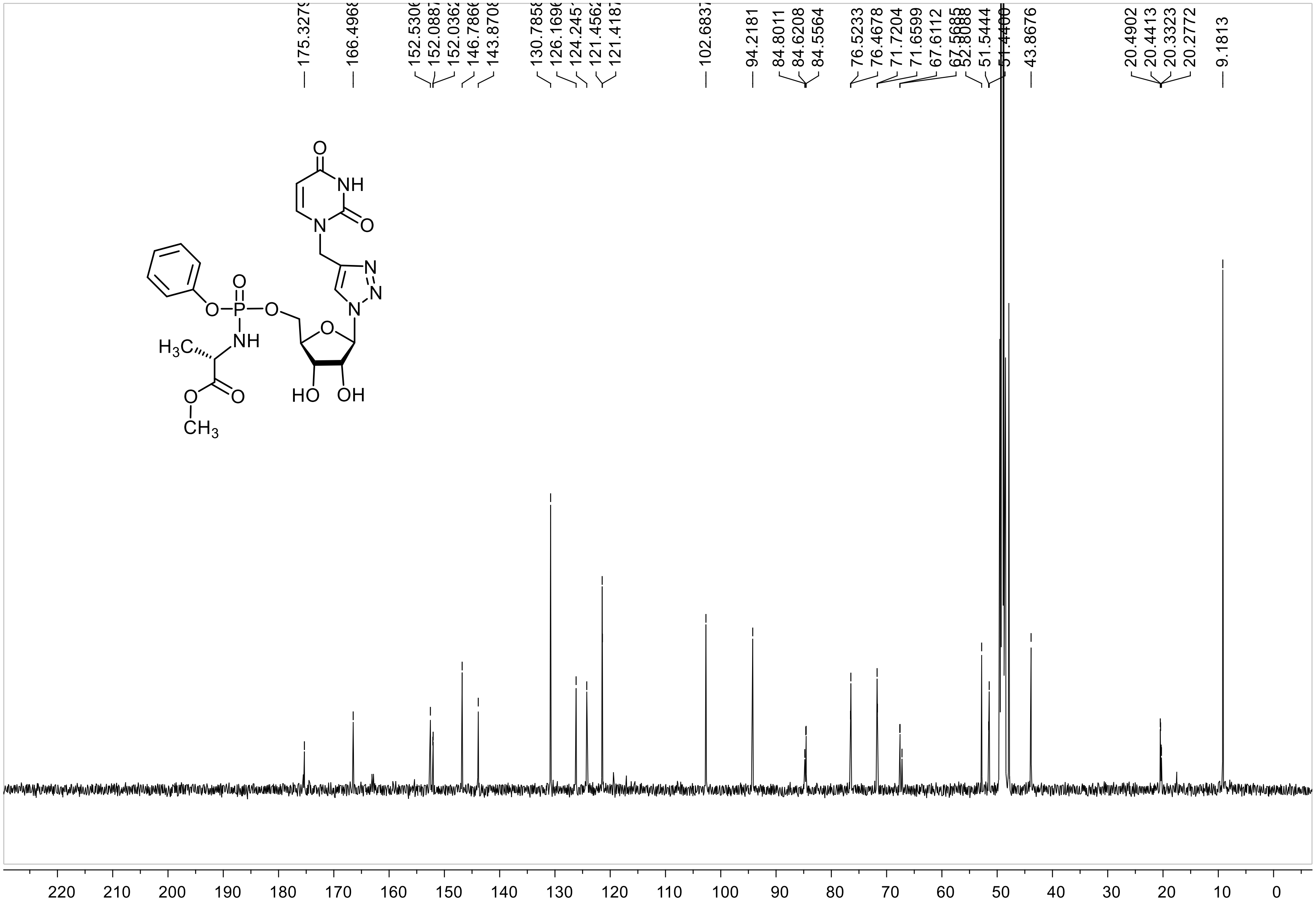

Figure S40. ^{13}C NMR spectrum of **17a** in DMSO-d_6

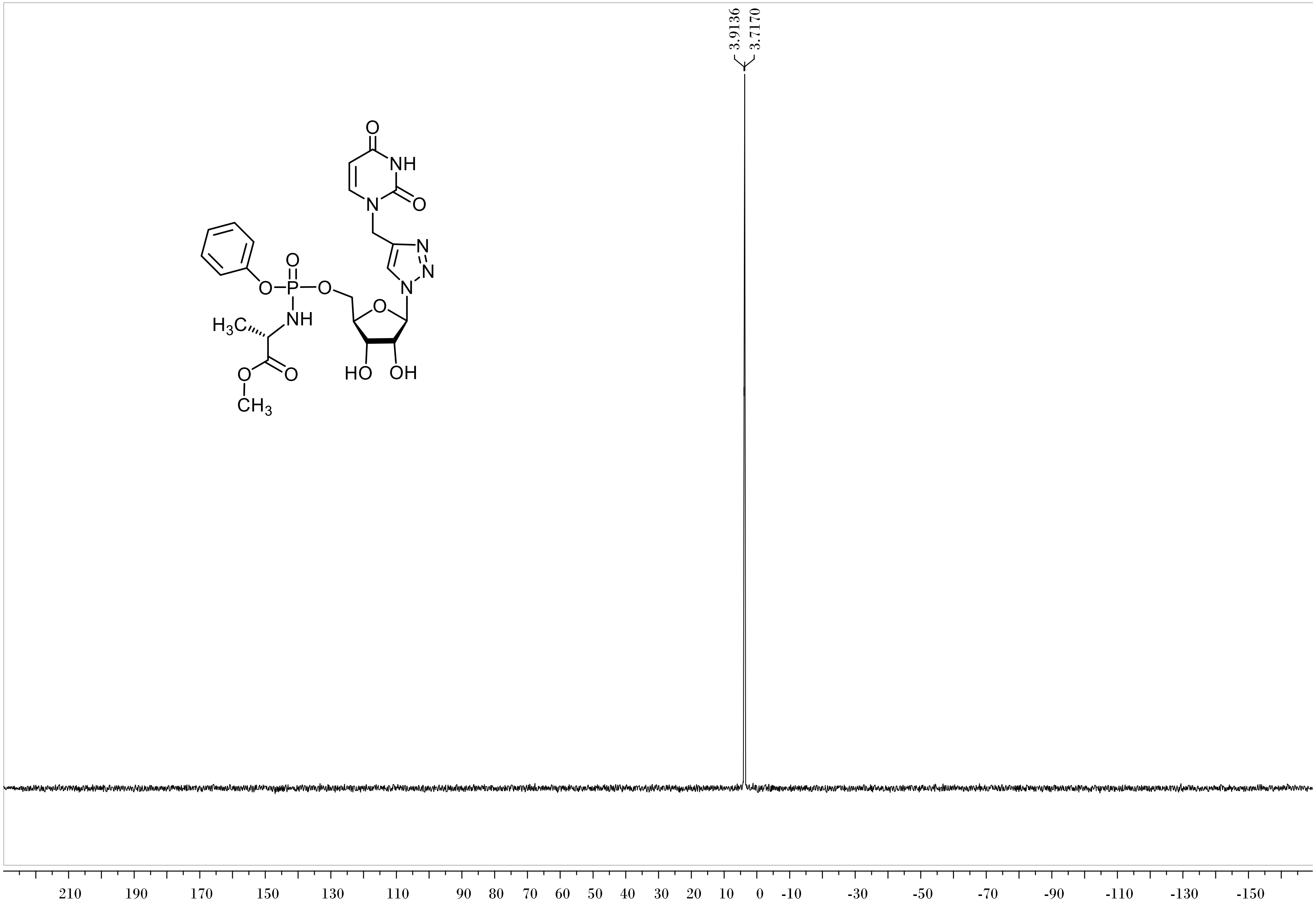

Figure S41. ^{31}P NMR spectrum of **17a** in CD_3OD

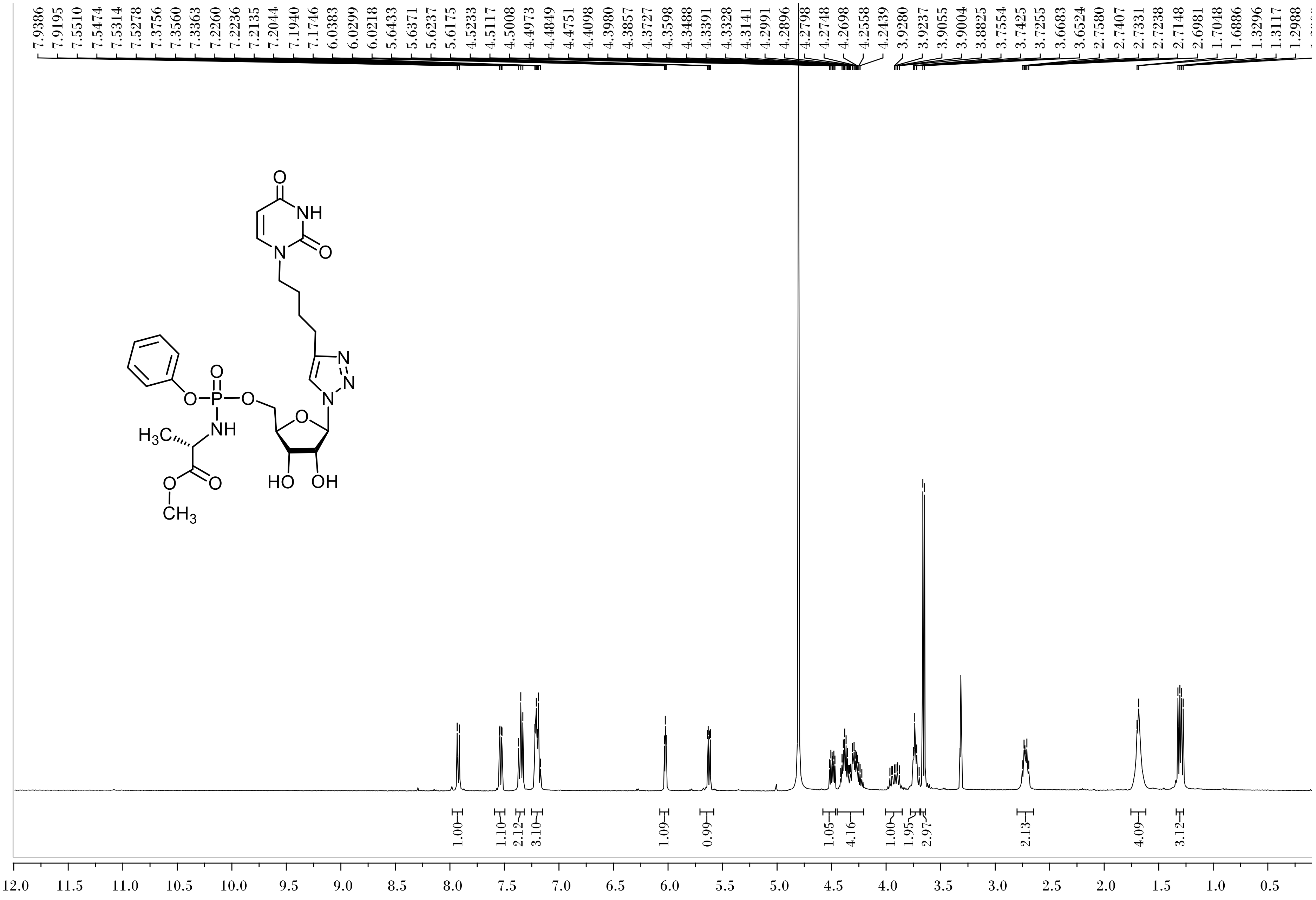

Figure S42. ^{1}H NMR spectrum of **18a** in CD_3OD

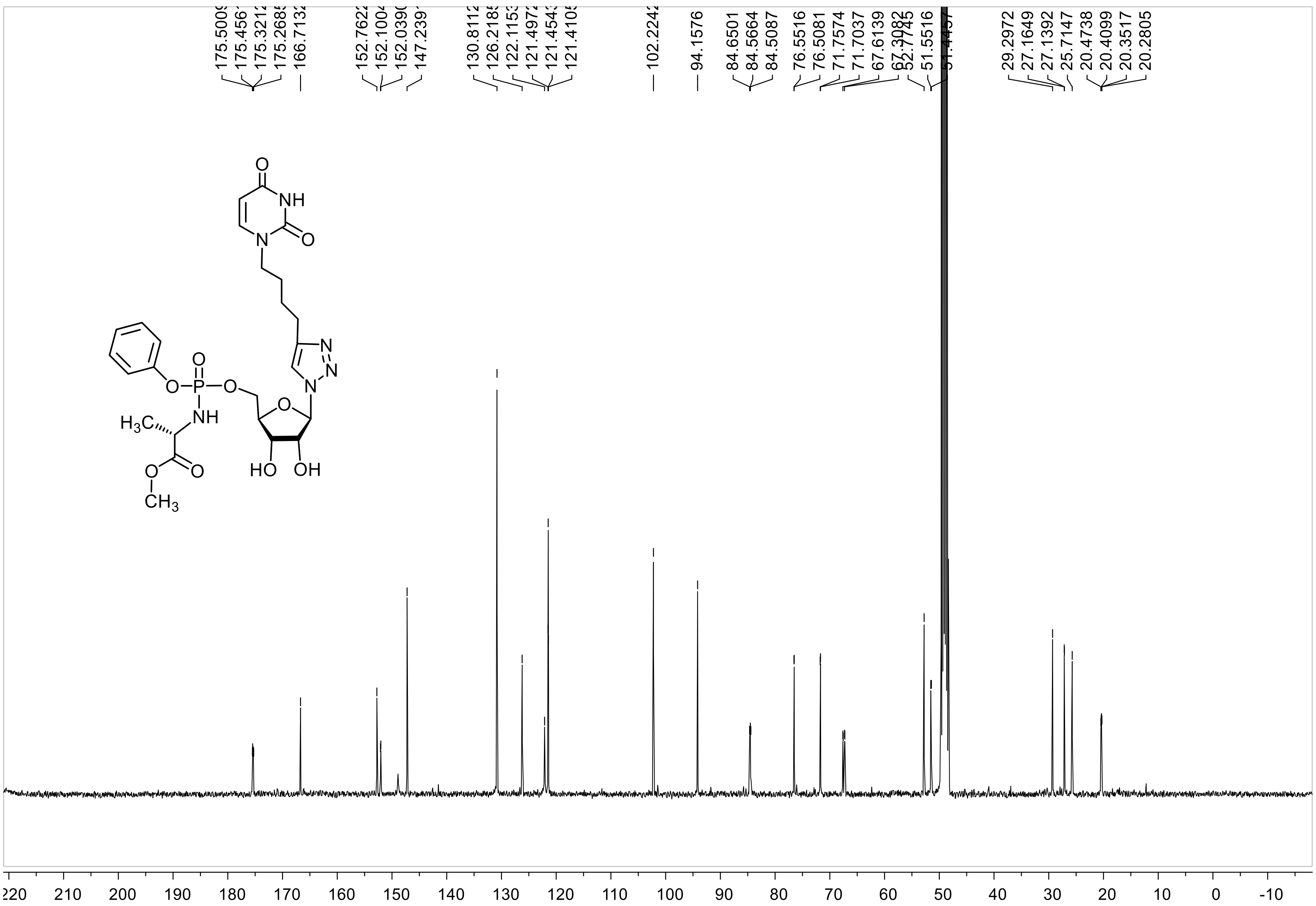

Figure S43. ^{13}C NMR spectrum of **18a** in CD_3OD

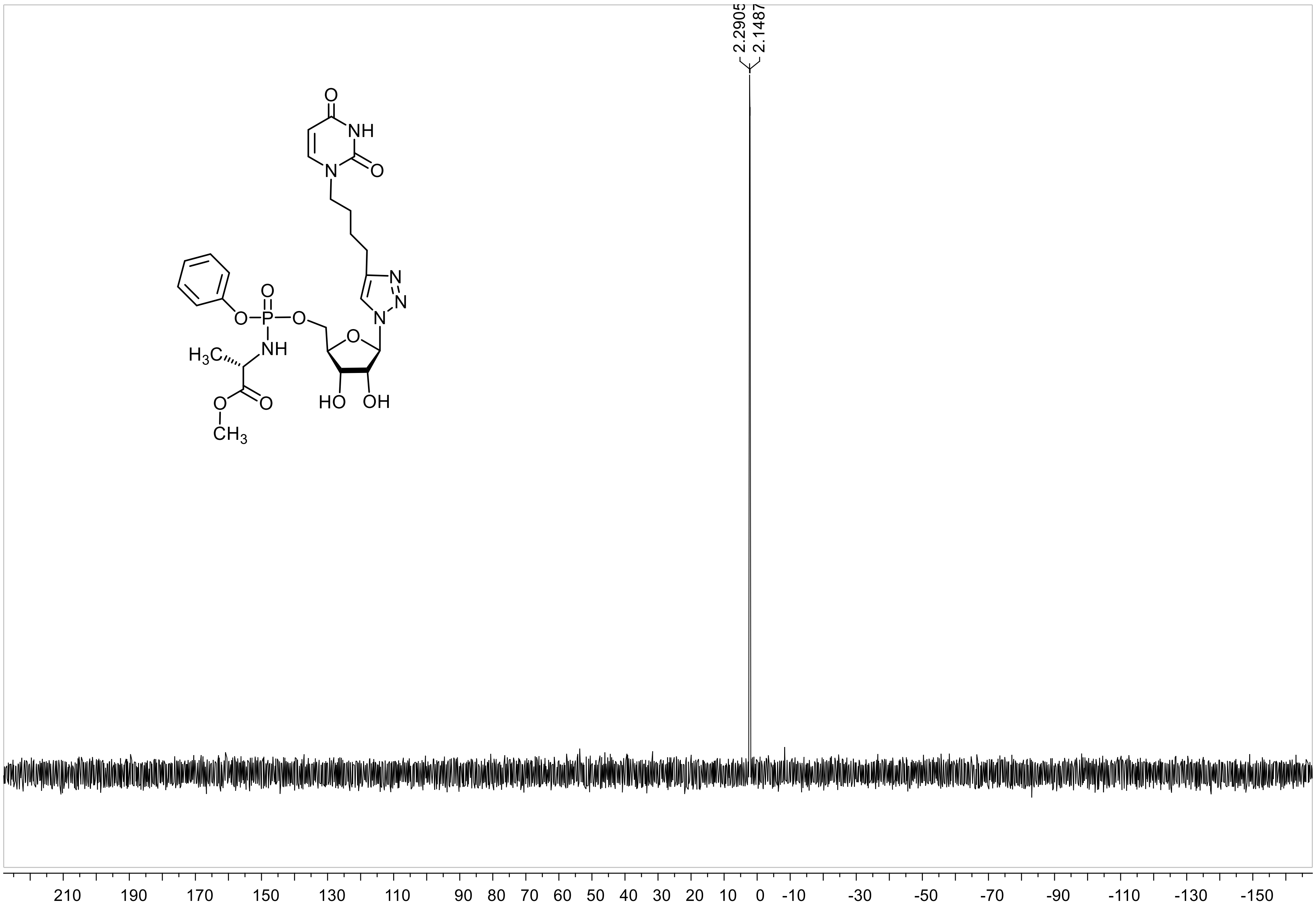

Figure S44. ^{31}P NMR spectrum of **18a** in CD_3OD

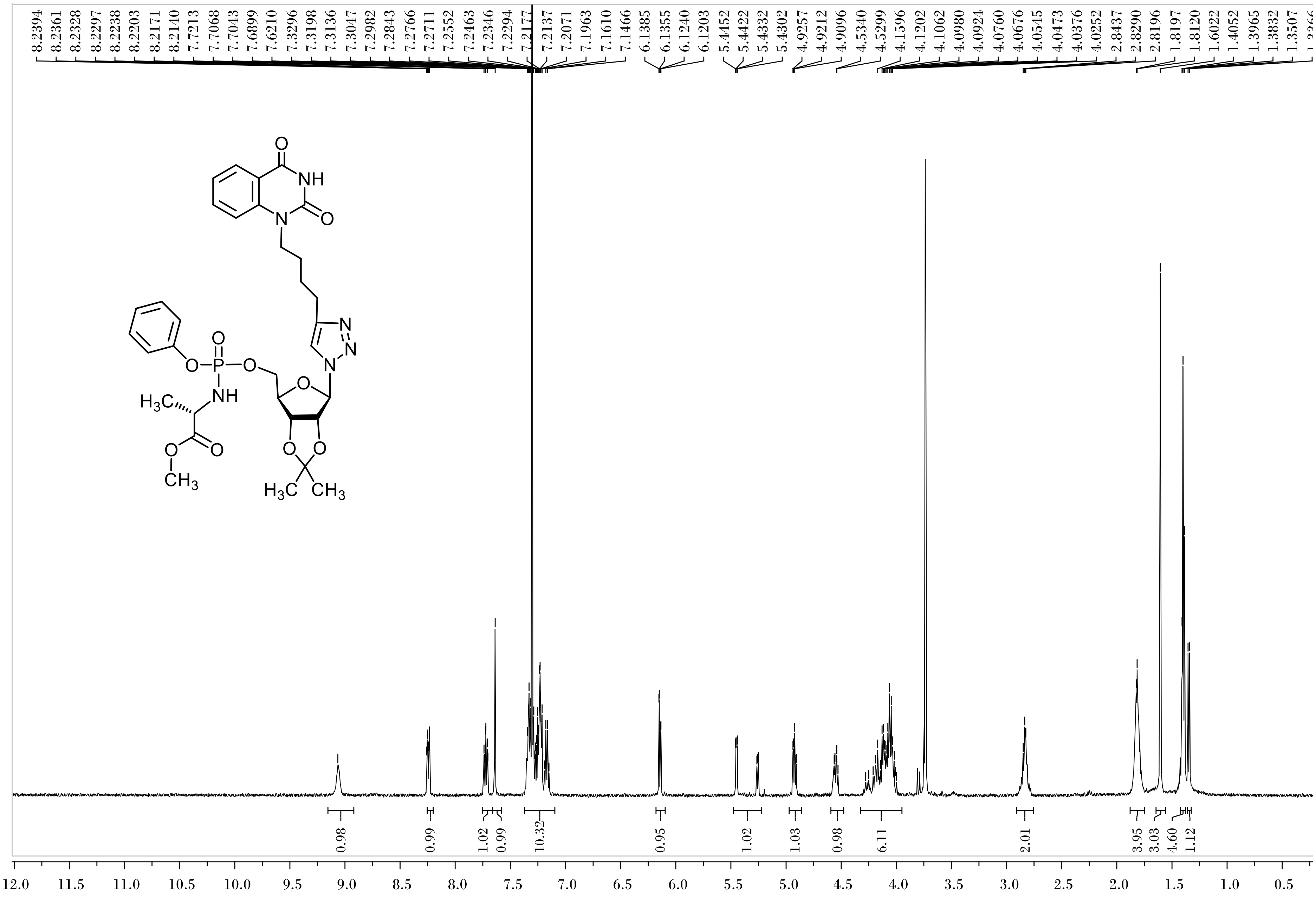

Figure S45. ^{1}H NMR spectrum of **16b** in $CDCl_3$

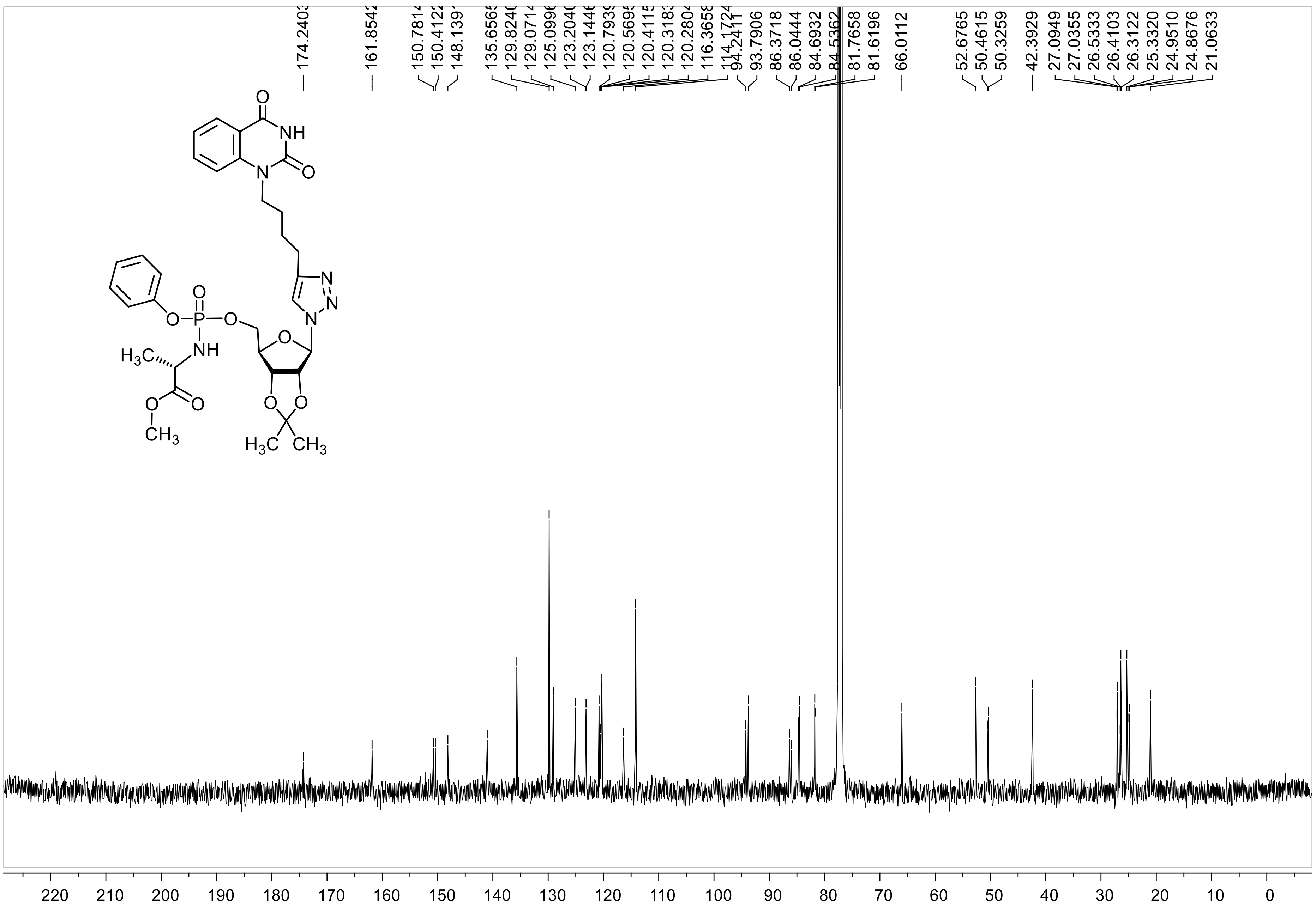

Figure S46. ^{13}C NMR spectrum of **16b** in $CDCl_3$

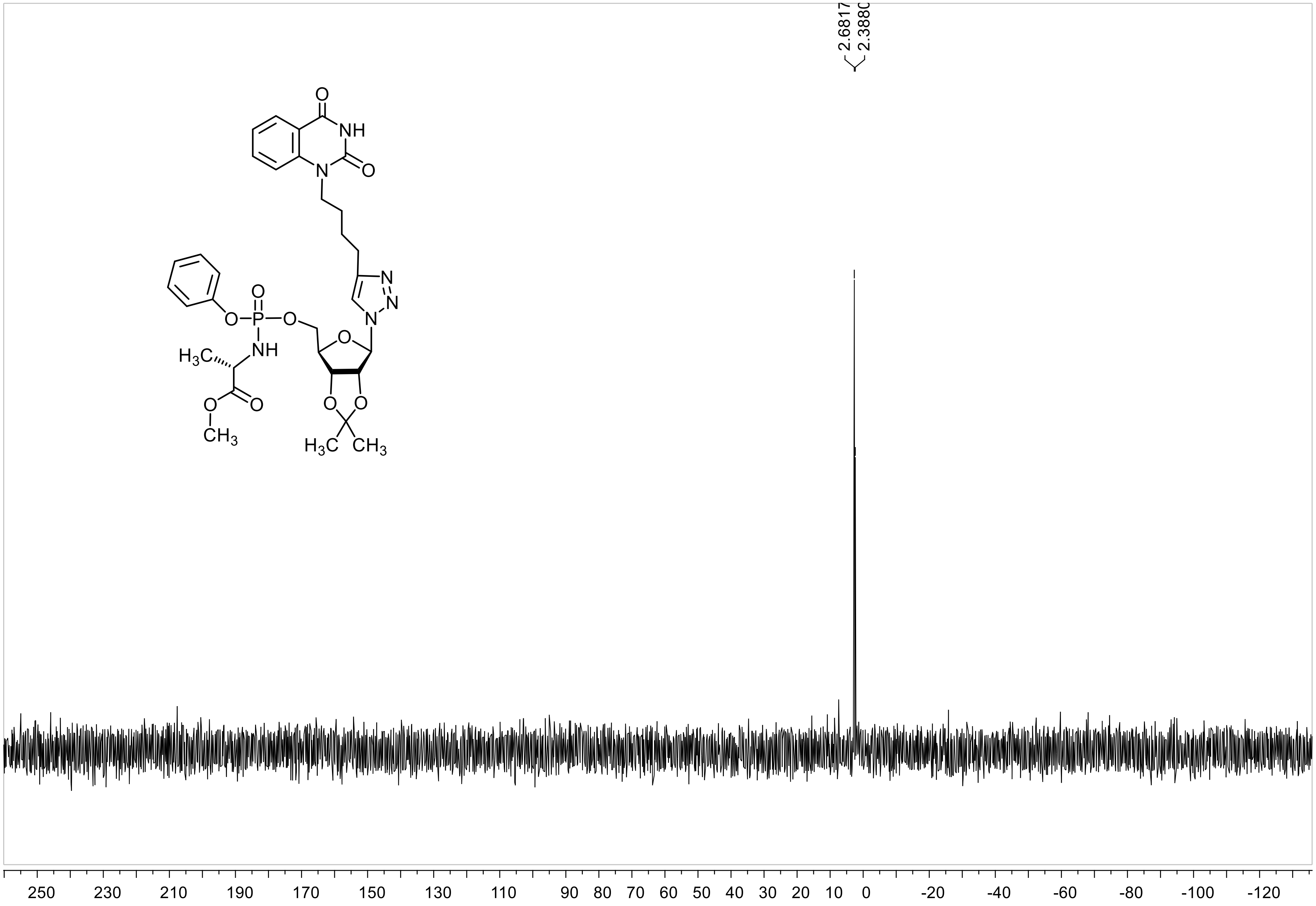

Figure S47. ^{31}P NMR spectrum of **16b** in $CDCl_3$

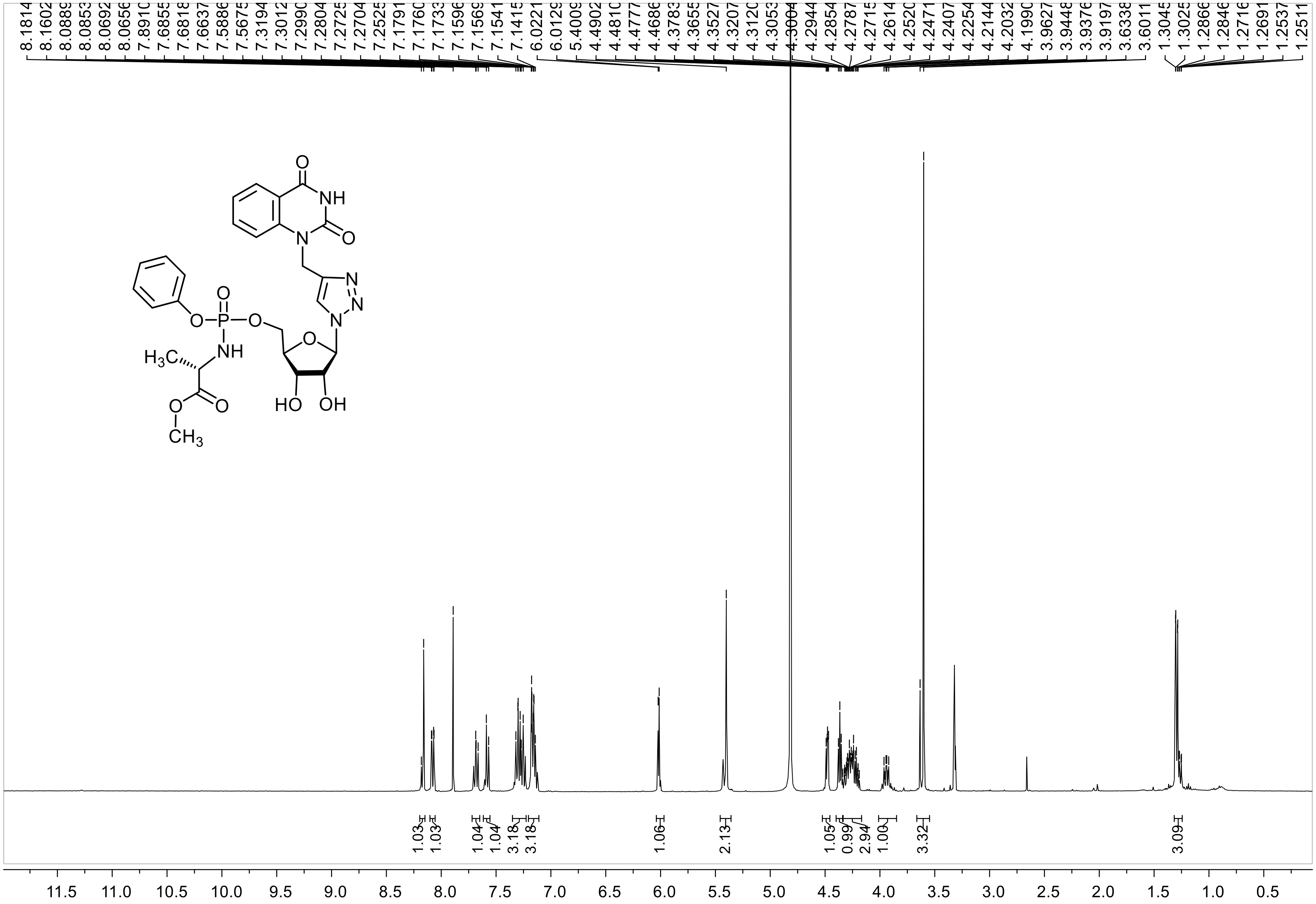

Figure S48. ^{1}H NMR spectrum of **17b** in CD_3OD

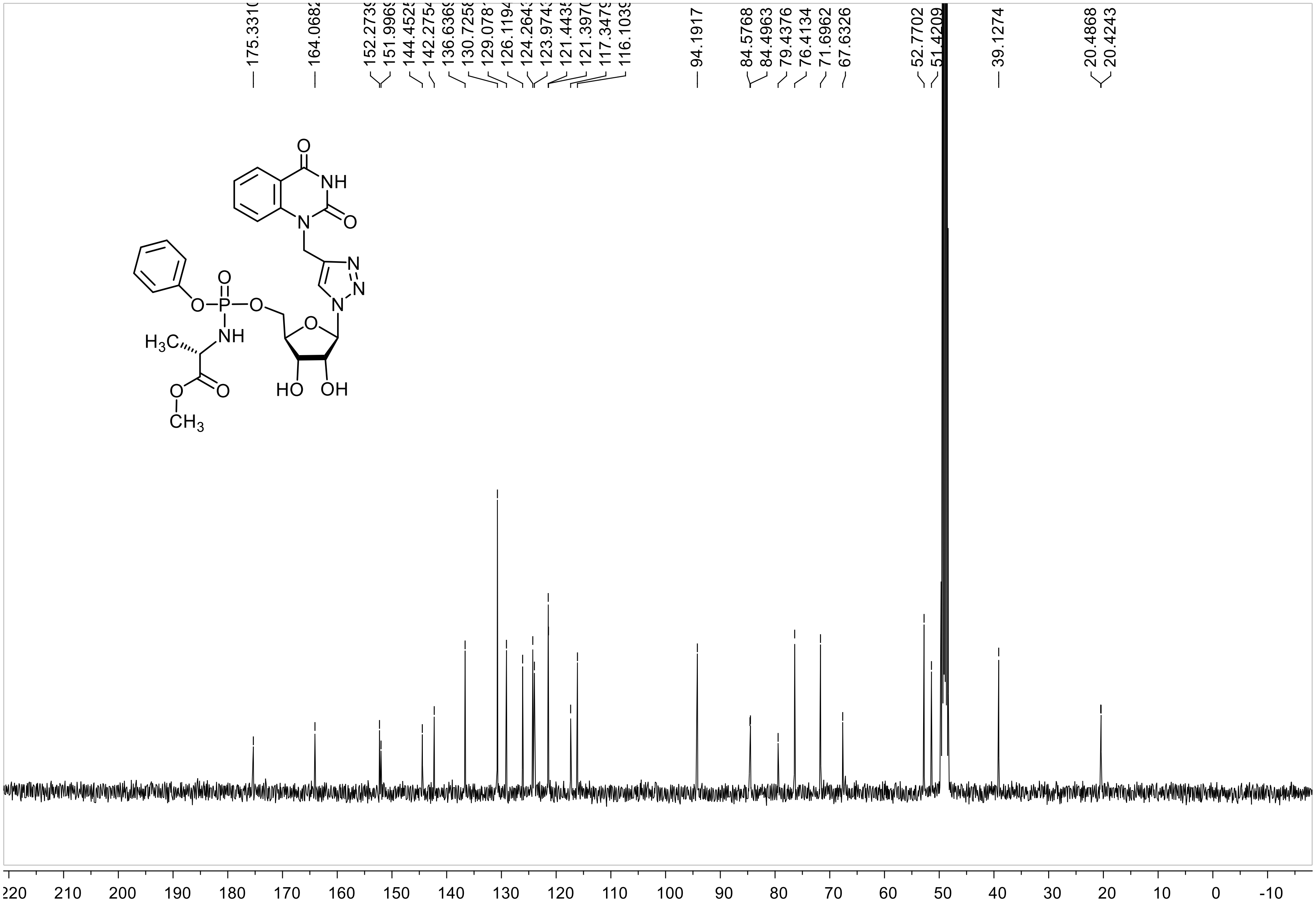

Figure S49. ^{13}C NMR spectrum of **17b** in CD_3OD

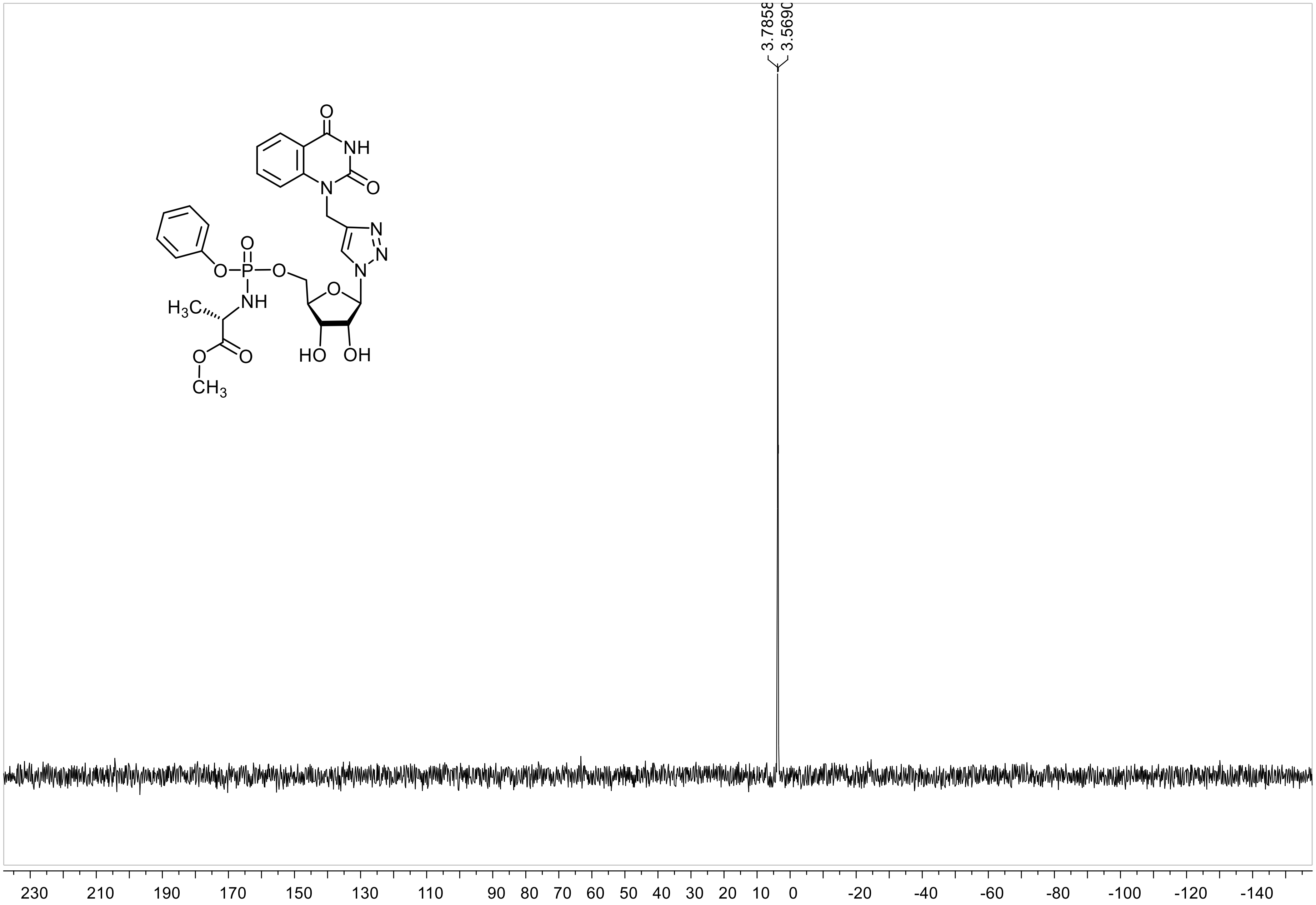

Figure S50. ^{31}P NMR spectrum of **17b** in CD_3OD

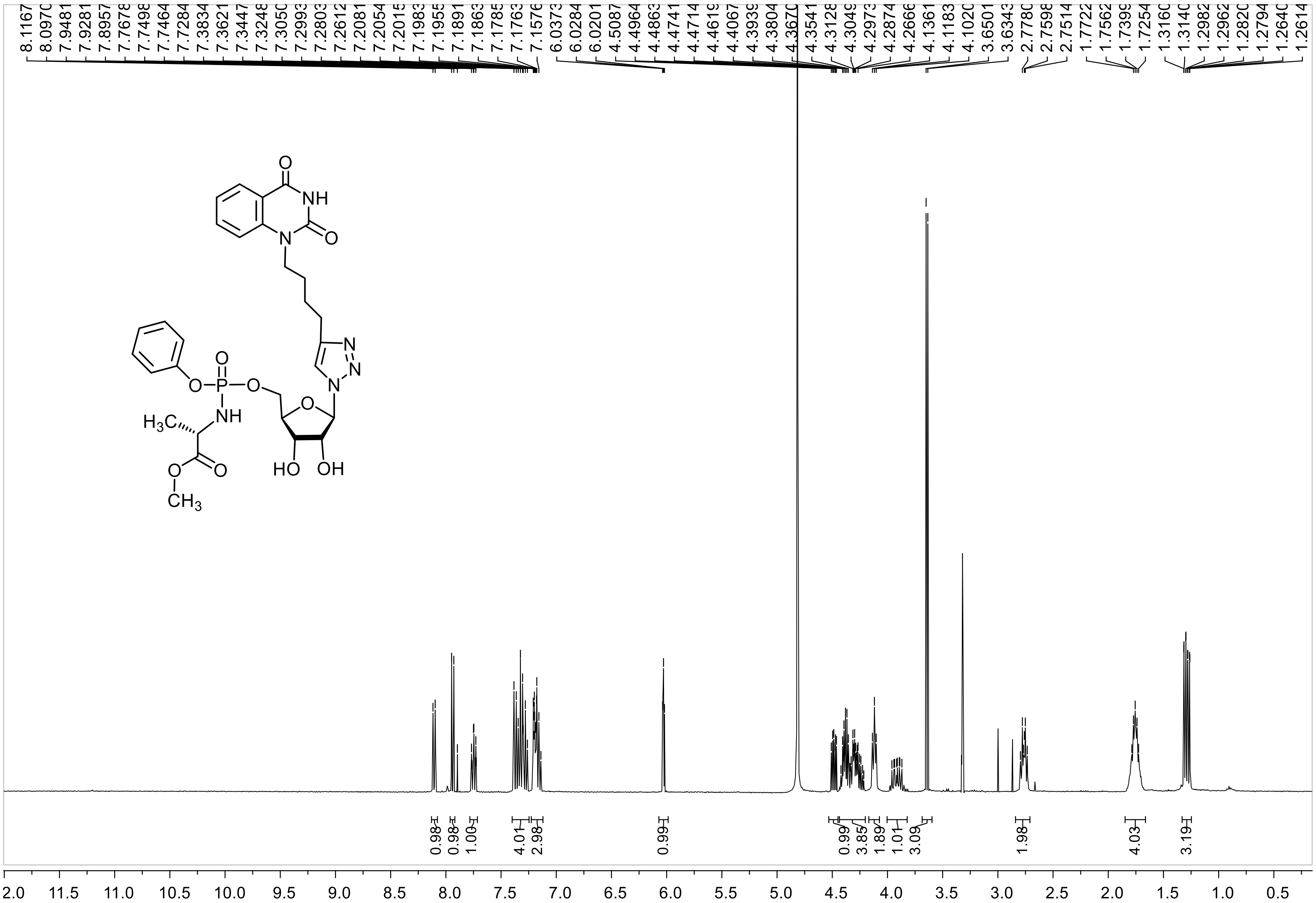

Figure S51. ^{1}H NMR spectrum of **18b** in CD_3OD

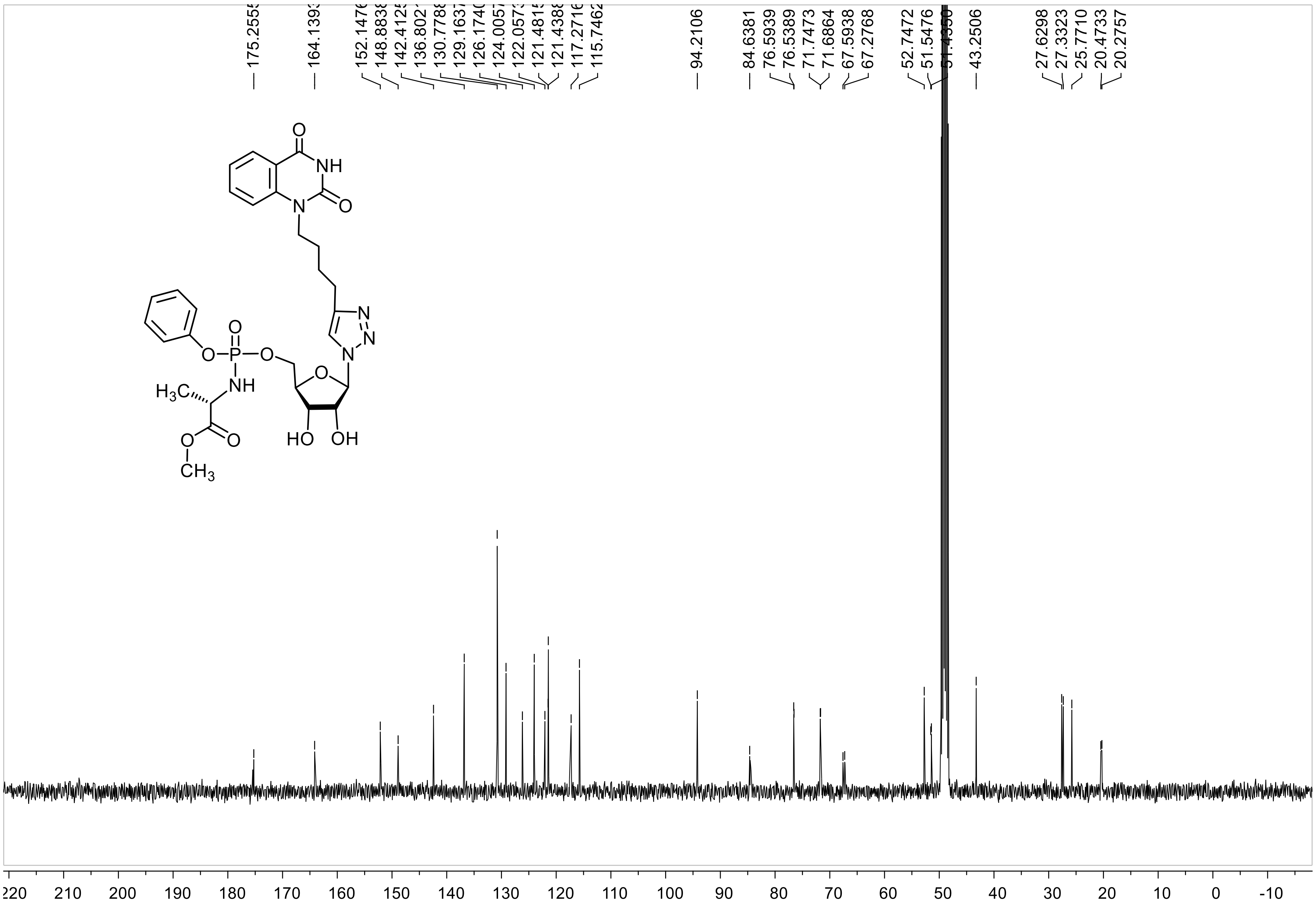

Figure S52. ^{13}C NMR spectrum of **18b** in CD_3OD

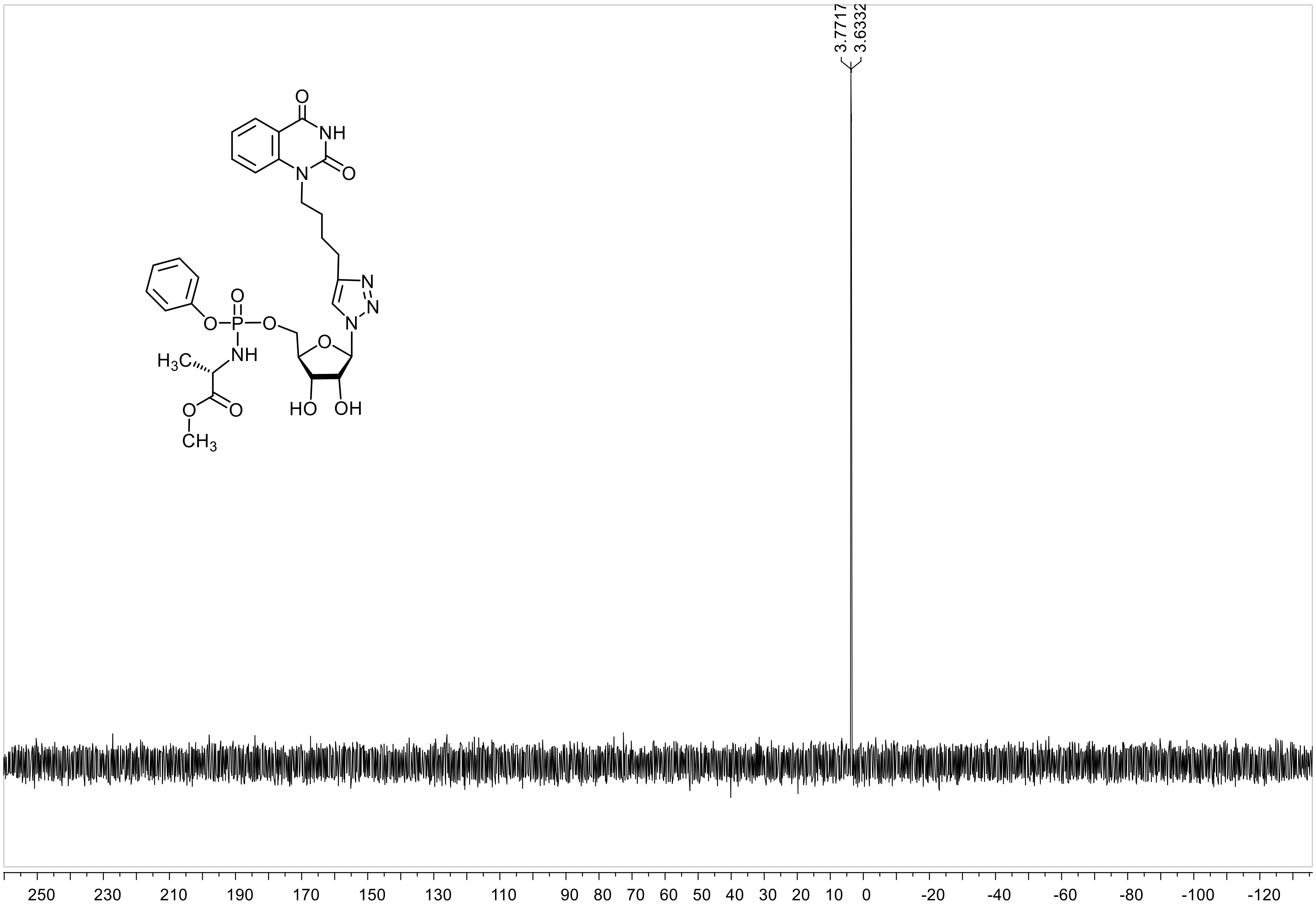

Figure S53. ^{31}P NMR spectrum of **18b** in $CDCl_3$

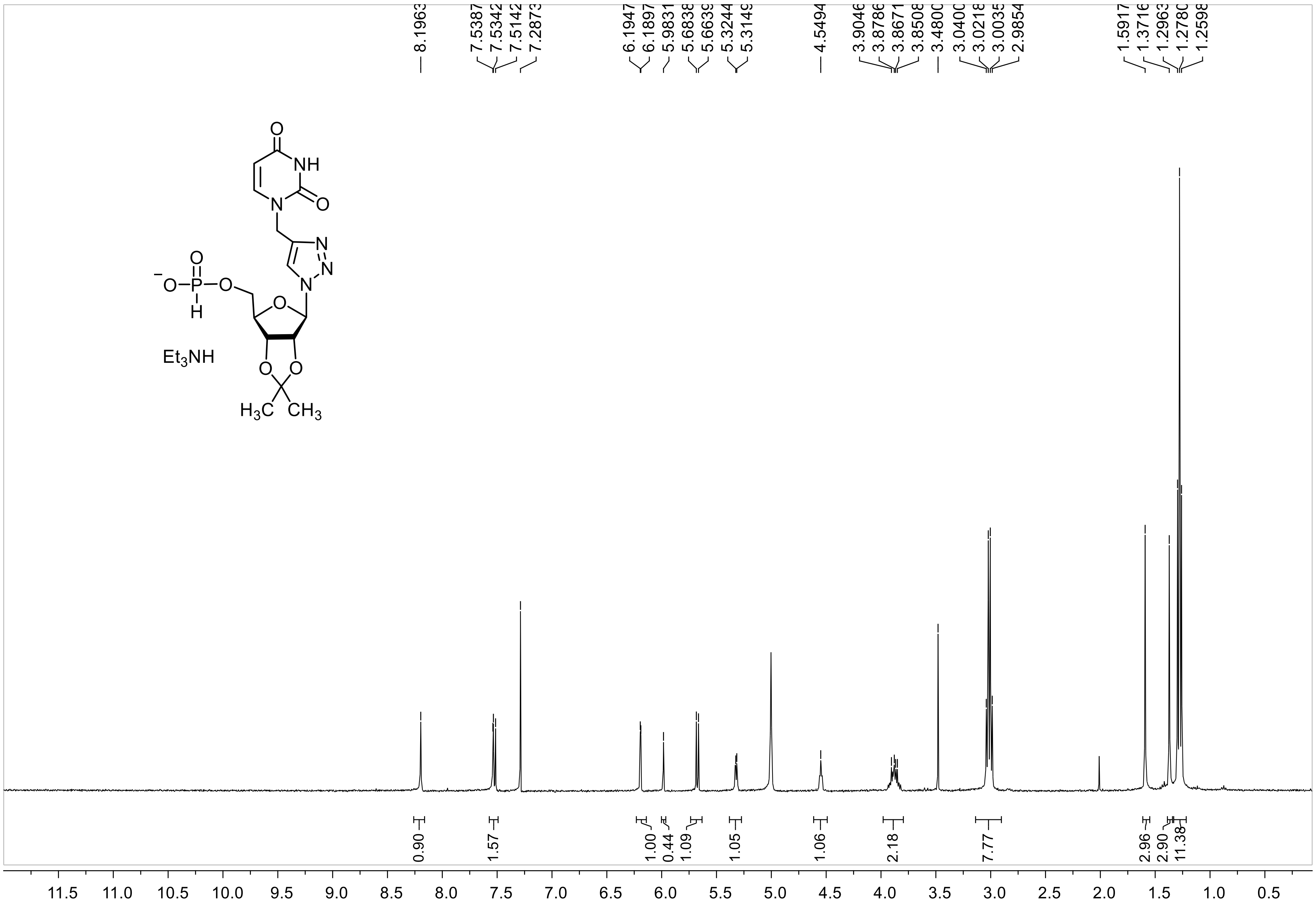

Figure S54. ^{1}H NMR spectrum of **21a** in $CDCl_3$

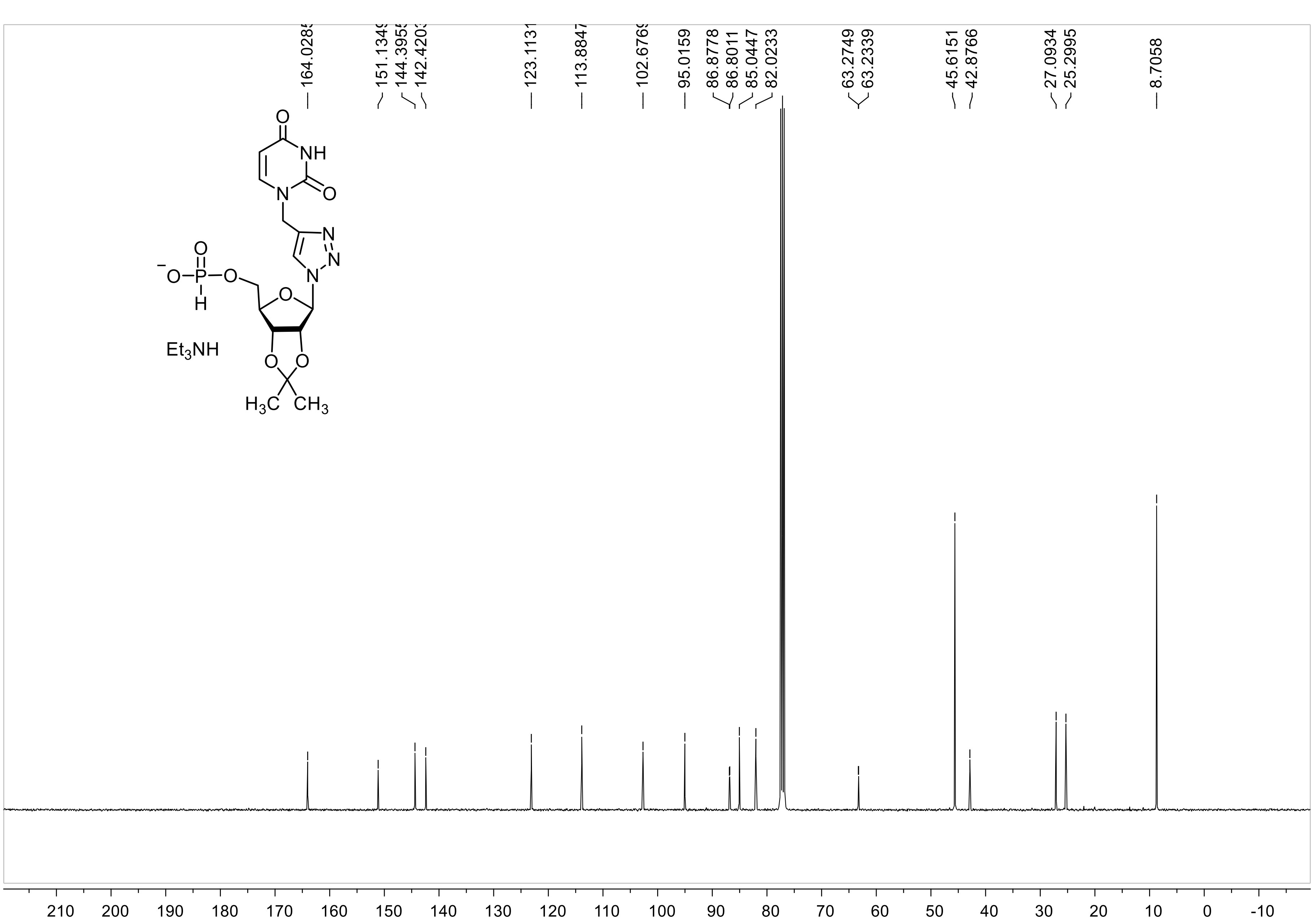

Figure S55. ^{13}C NMR spectrum of **21a** in $CDCl_3$

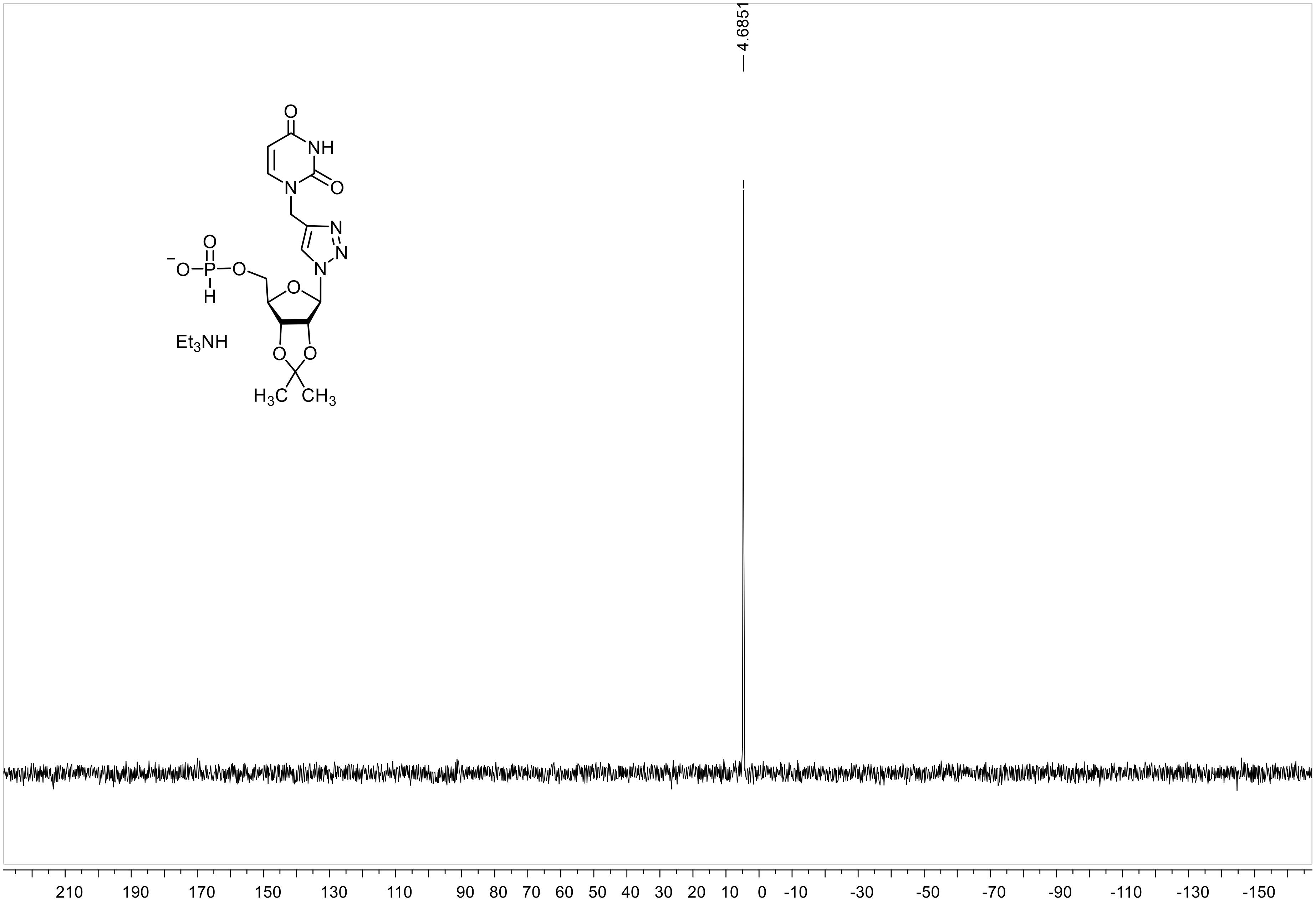

Figure S56. ^{31}P NMR spectrum of **21a** in $CDCl_3$

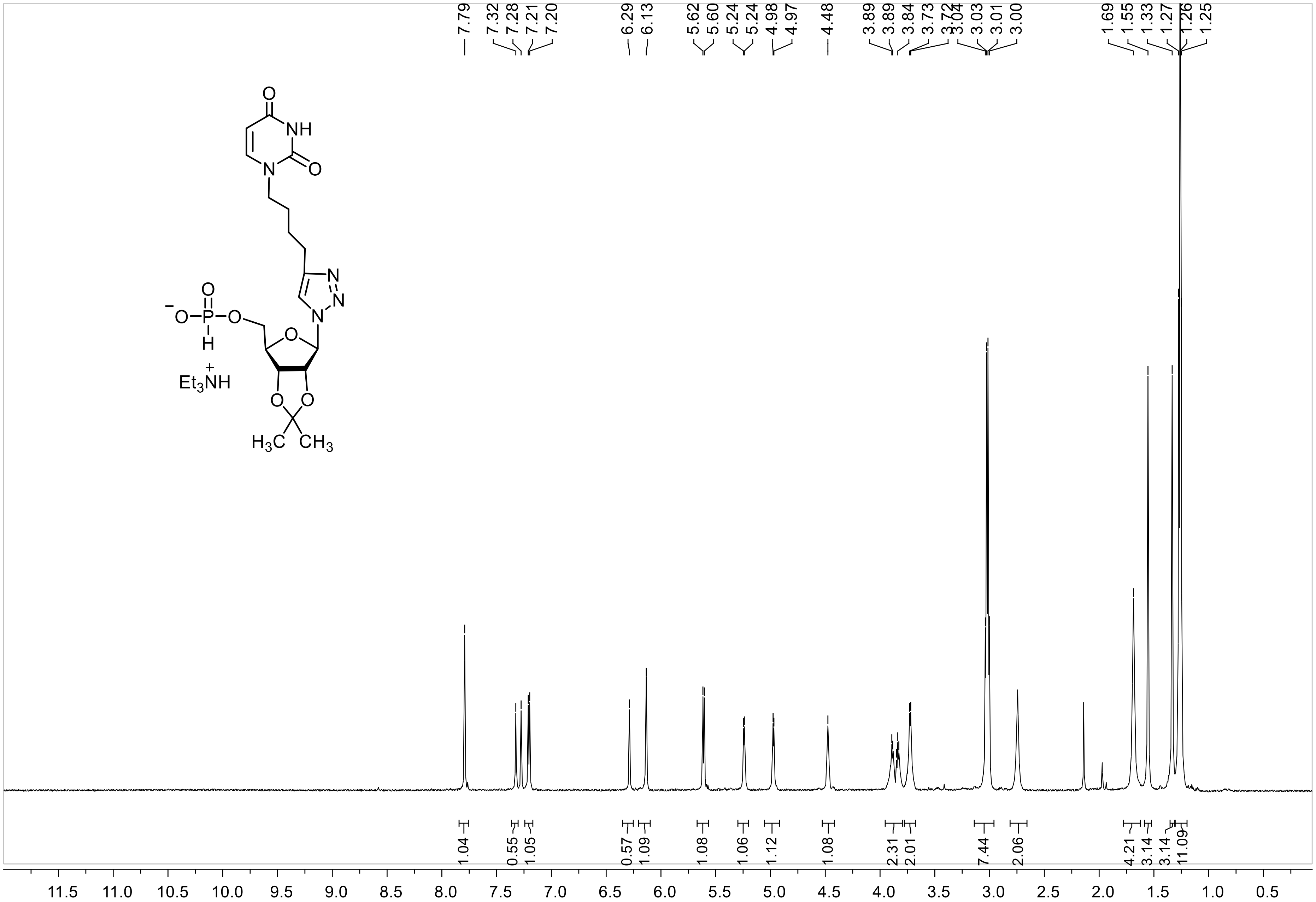

Figure S57. ^{1}H NMR spectrum of **22a** in $CDCl_3$

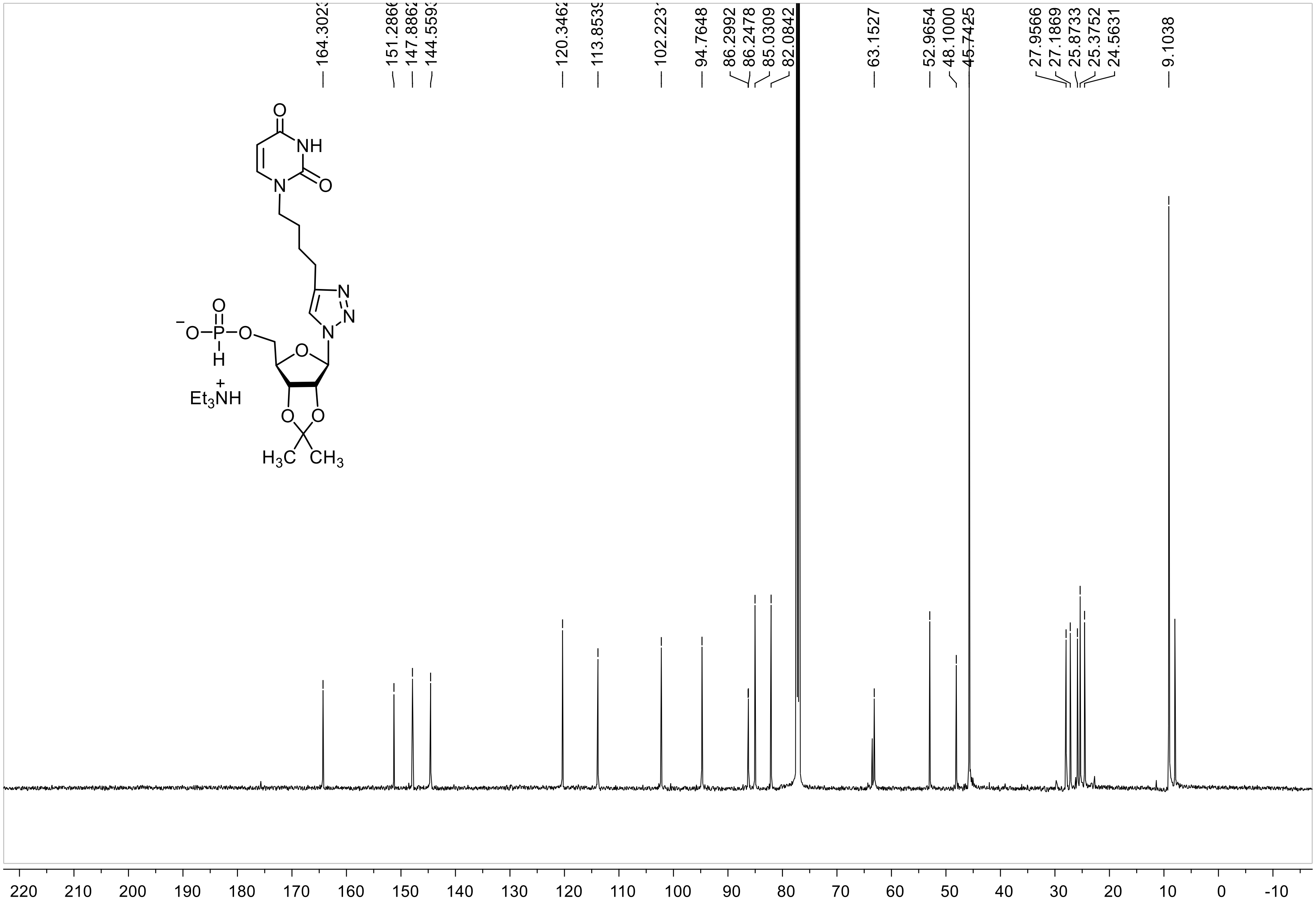

Figure S58. ^{13}C NMR spectrum of **22a** in $CDCl_3$

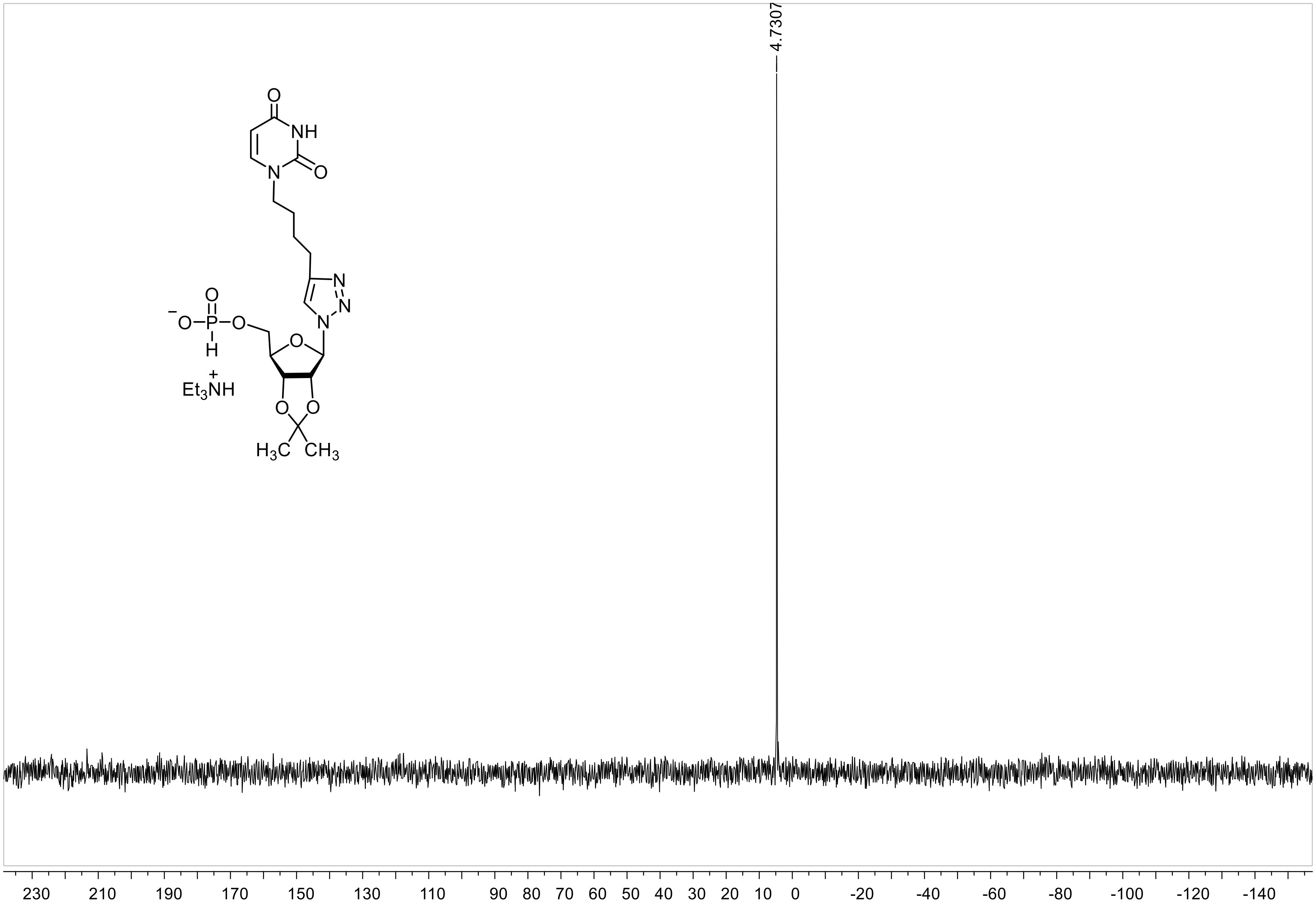

Figure S59. ^{31}P NMR spectrum of **22a** in $CDCl_3$

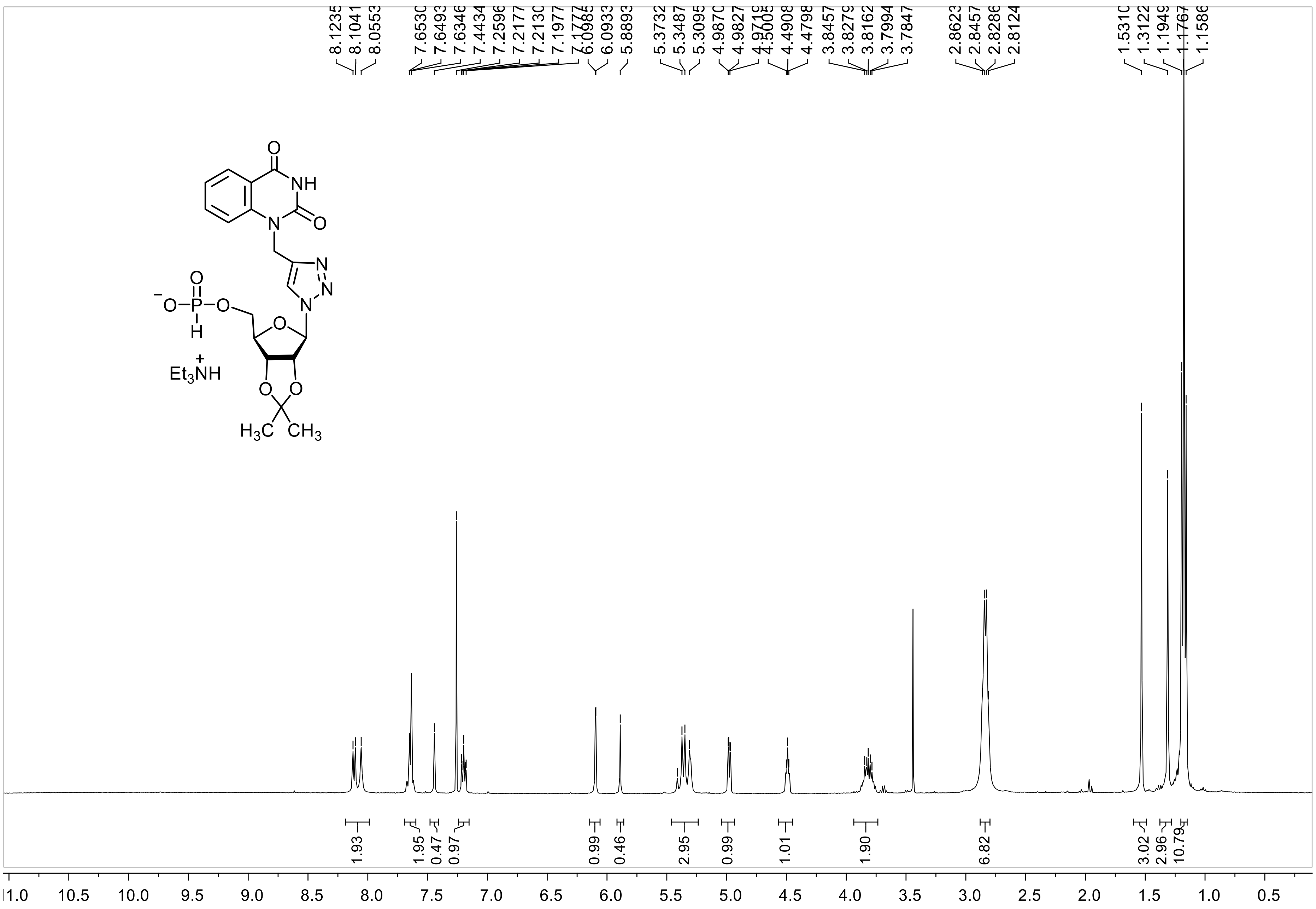

Figure S60. ^{1}H NMR spectrum of **21b** in $CDCl_3$

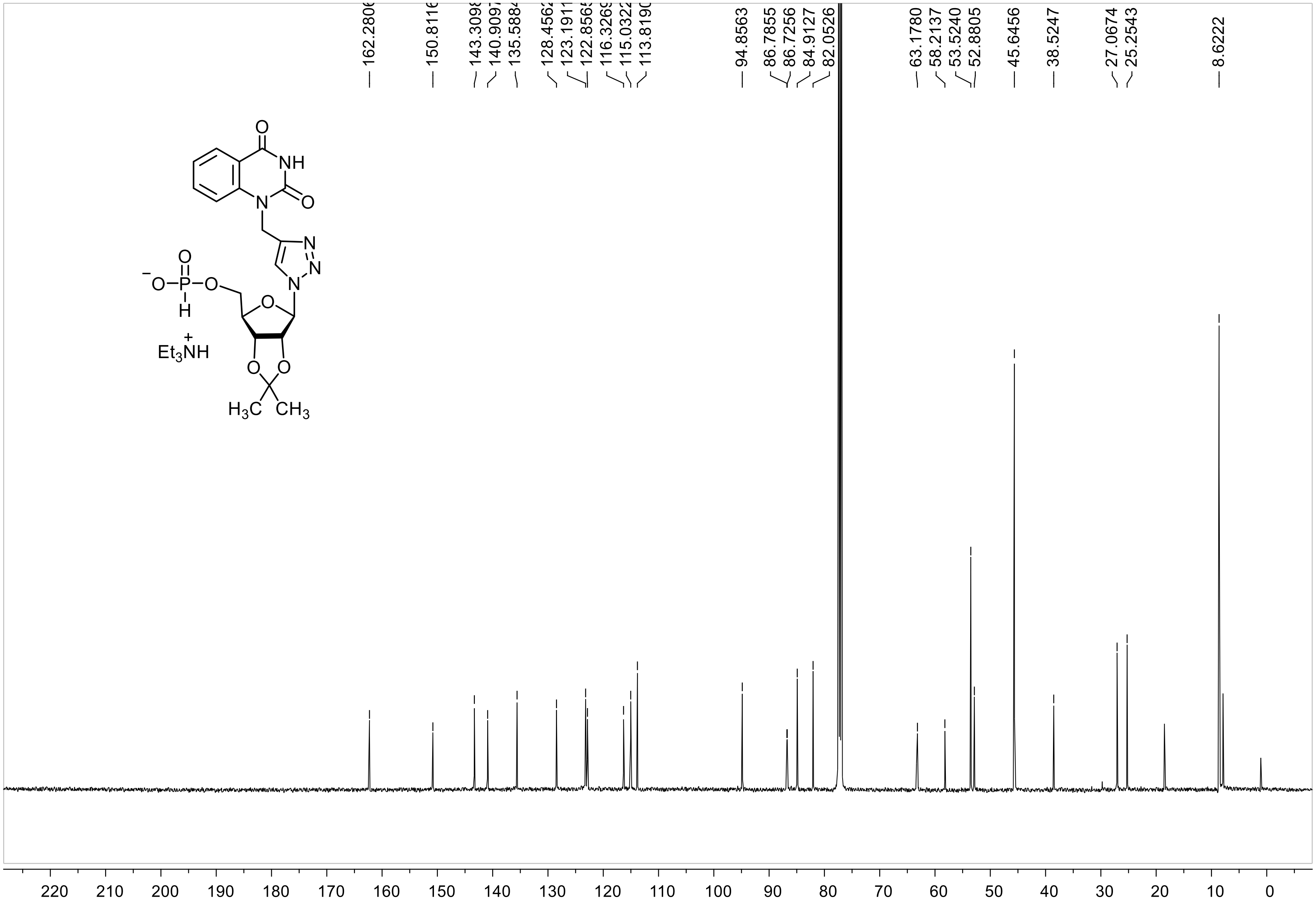

Figure S61. ^{13}C NMR spectrum of **21b** in $CDCl_3$

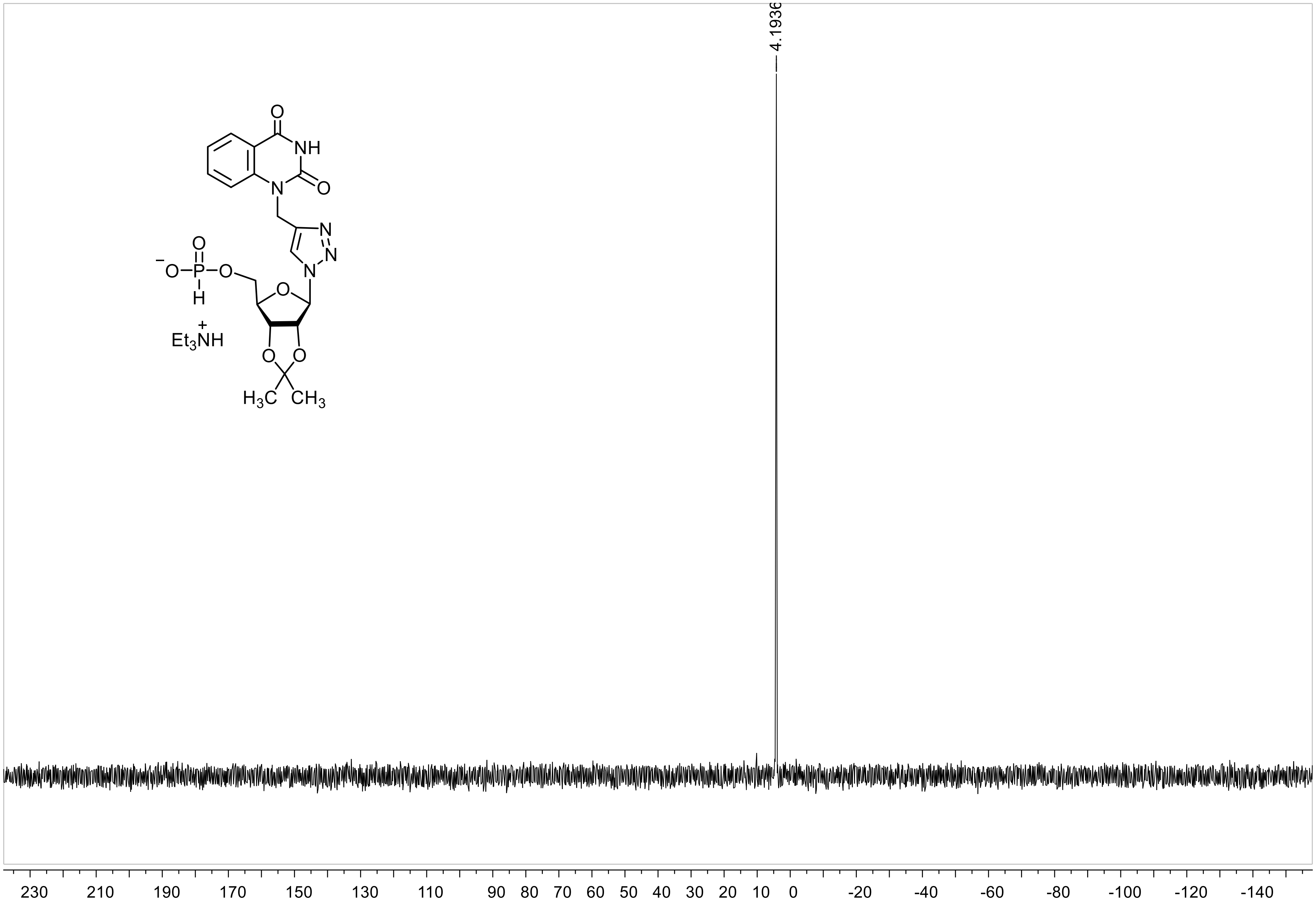

Figure S62. ^{31}P NMR spectrum of **21b** in $CDCl_3$

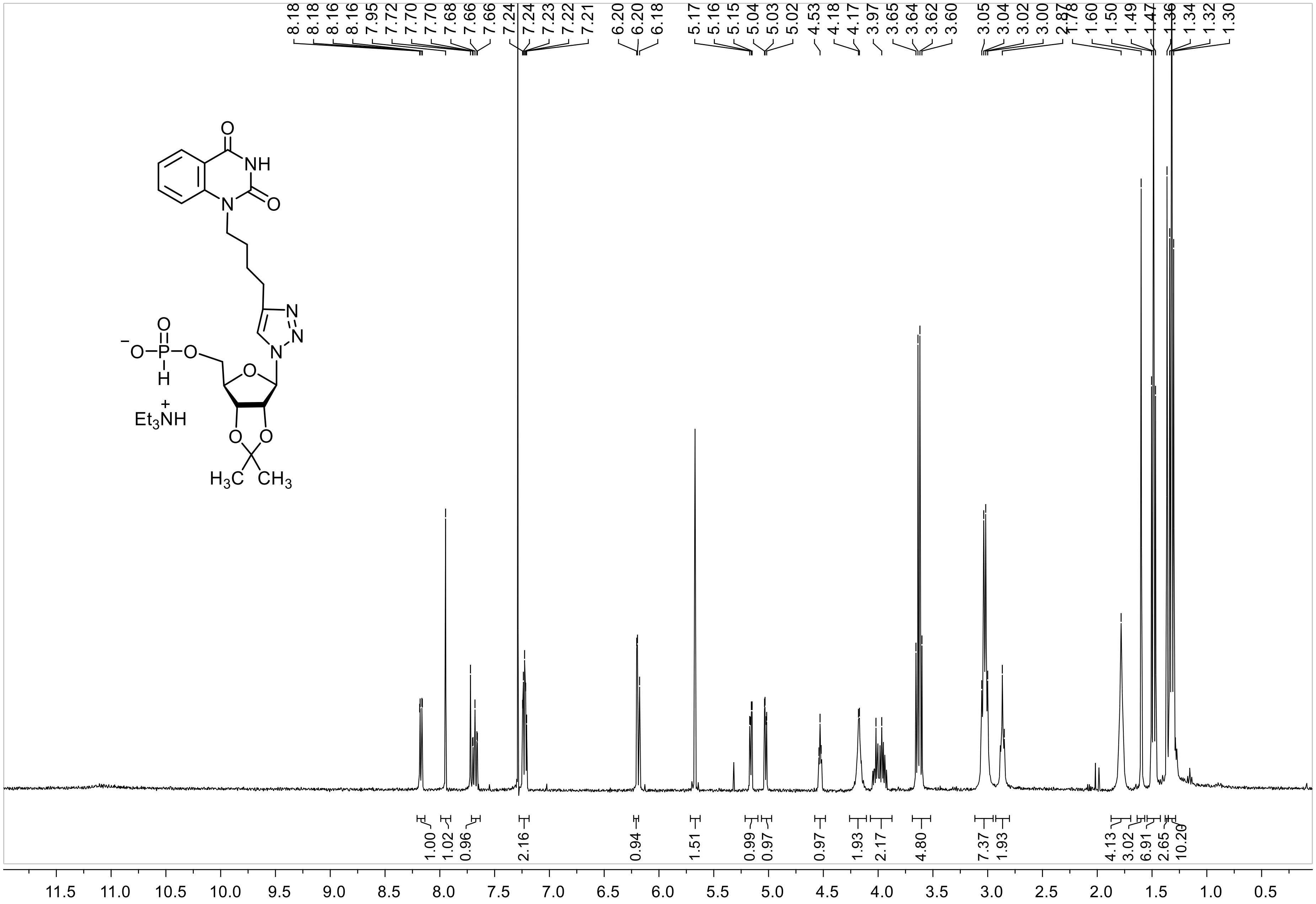

Figure S63. ^{1}H NMR spectrum of **22b** in $CDCl_3$

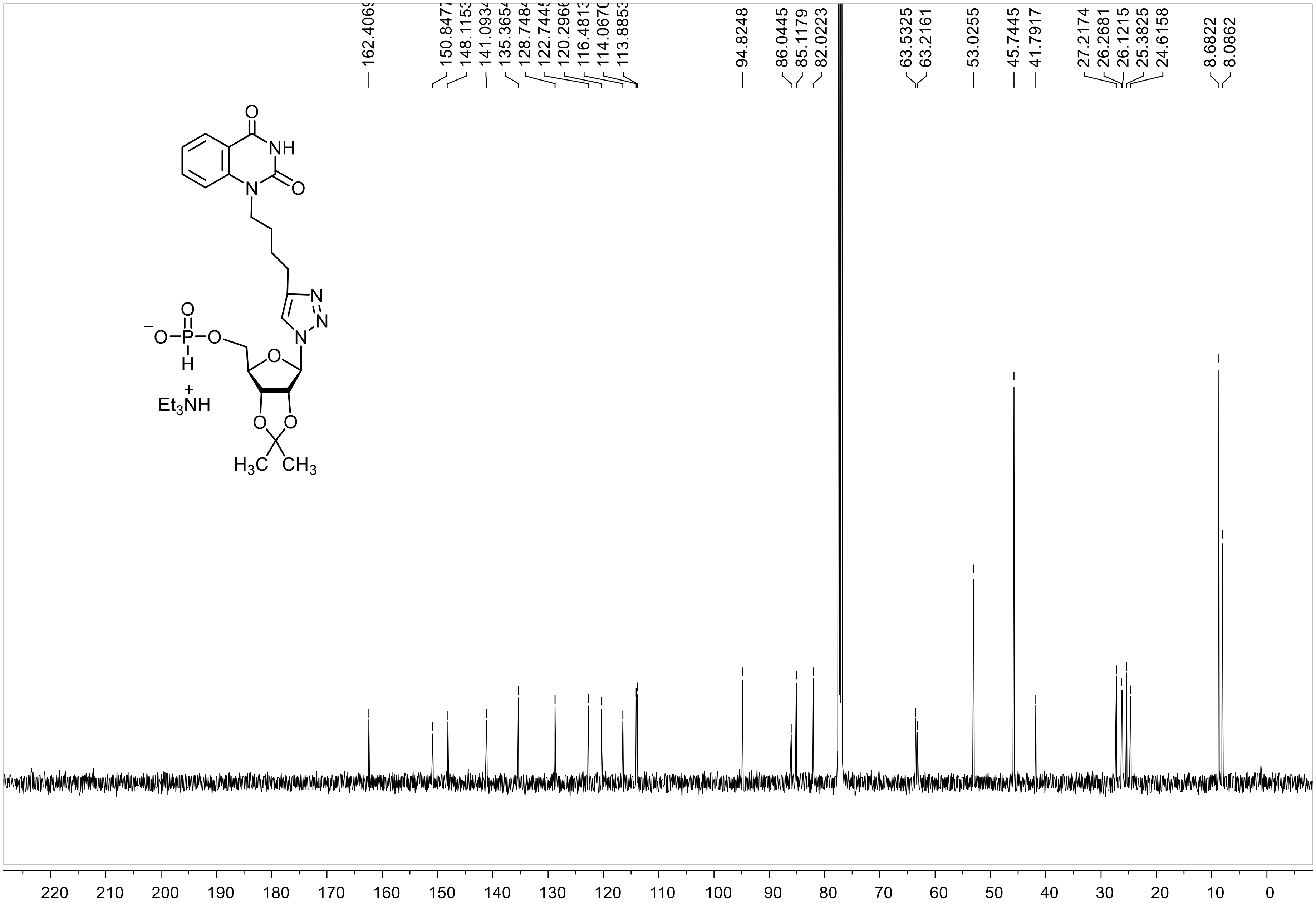

Figure S64. ^{13}C NMR spectrum of **22b** in $CDCl_3$

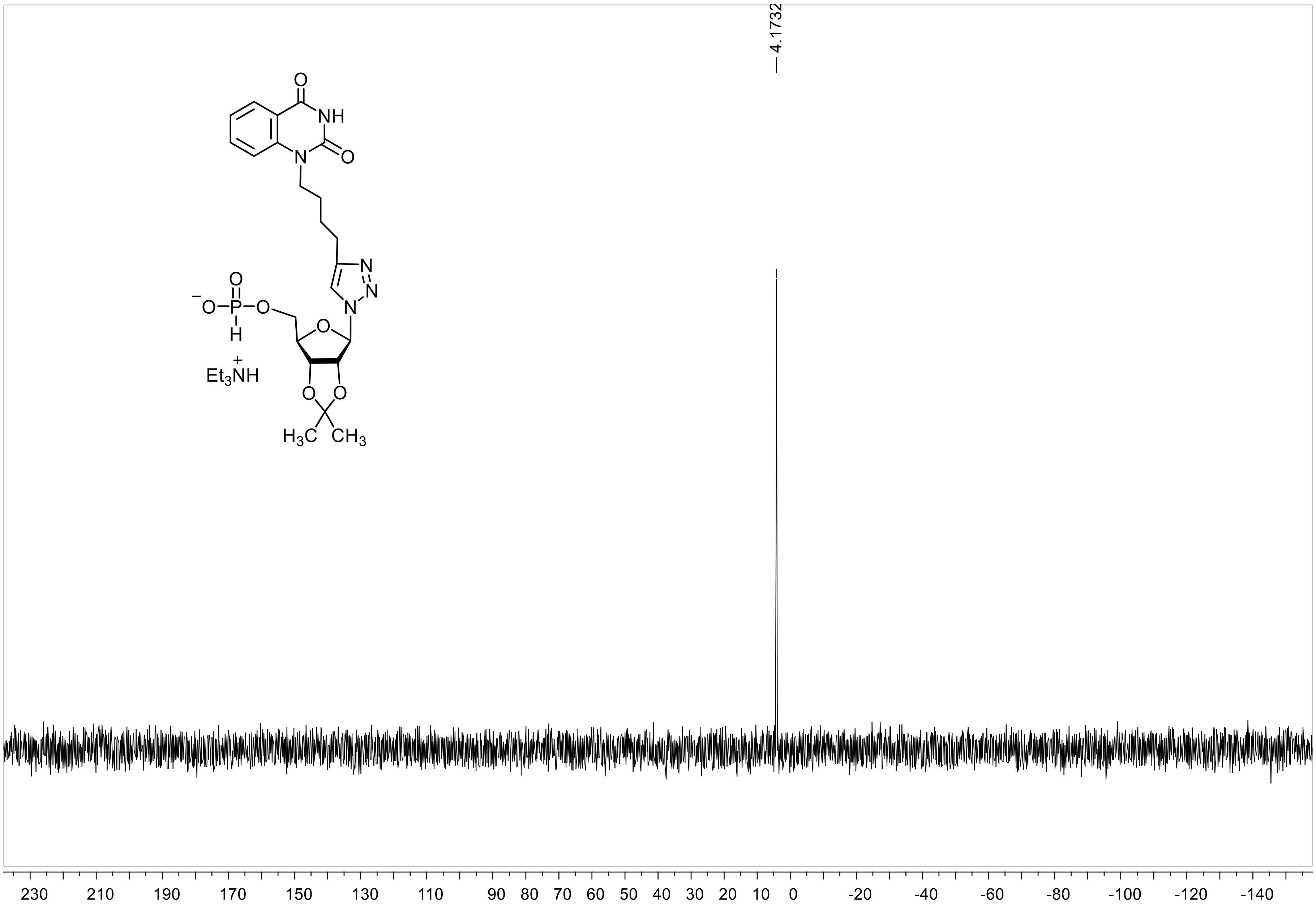

Figure S65. ^{31}P NMR spectrum of **22b** in $CDCl_3$

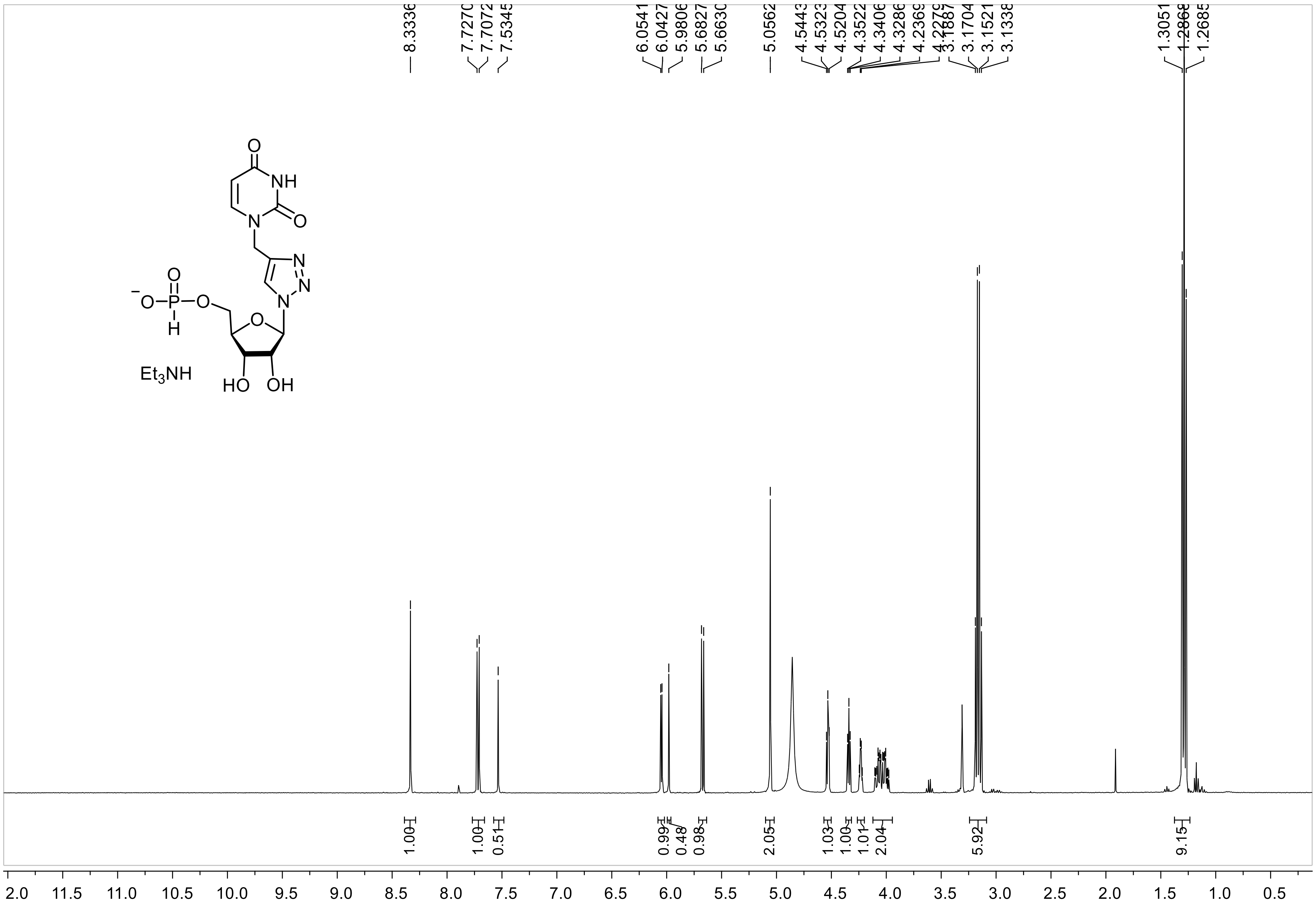

Figure S66. ^{1}H NMR spectrum of **23a** in CD_3OD

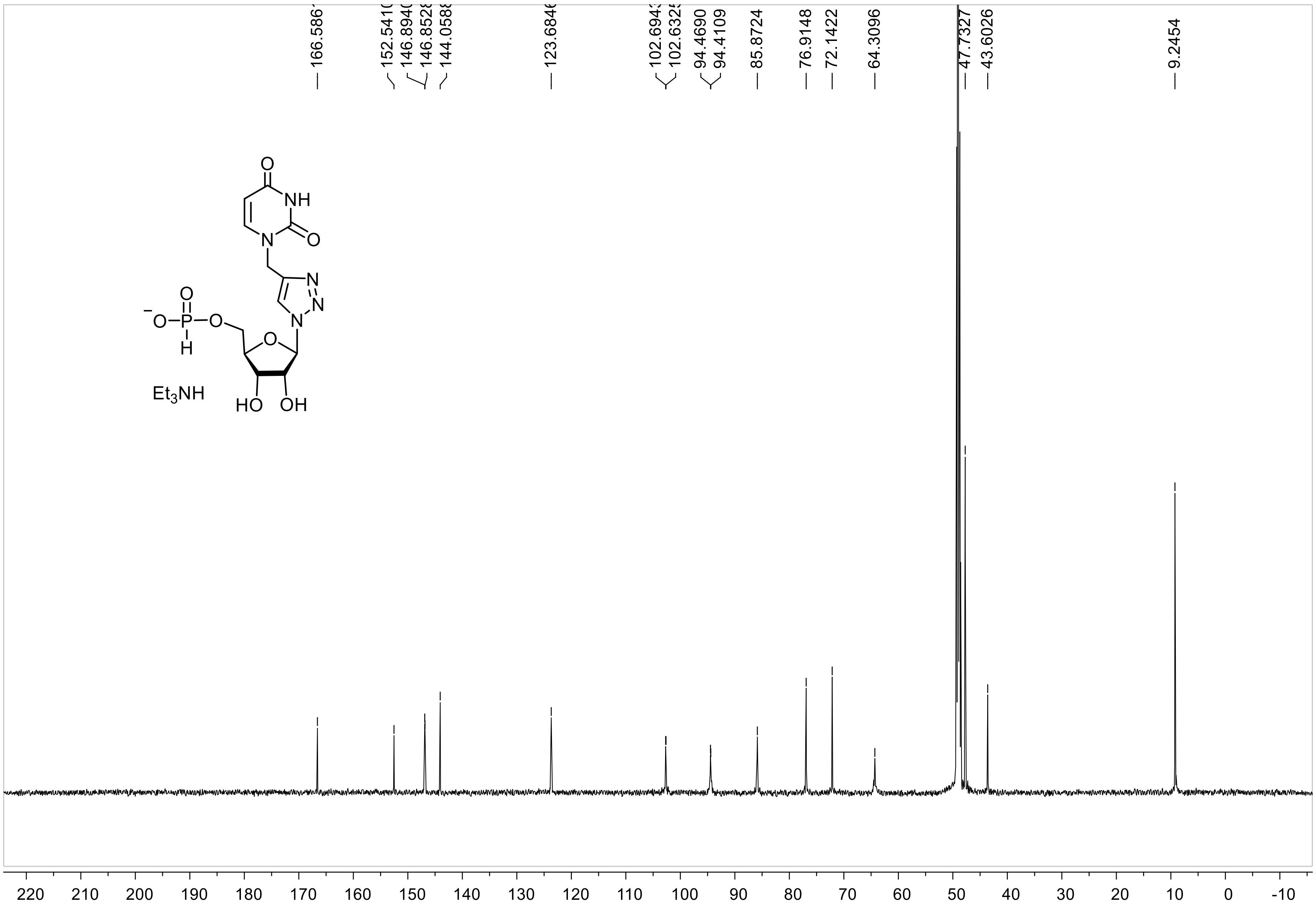

Figure S67. ^{13}C NMR spectrum of **23a** in CD_3OD

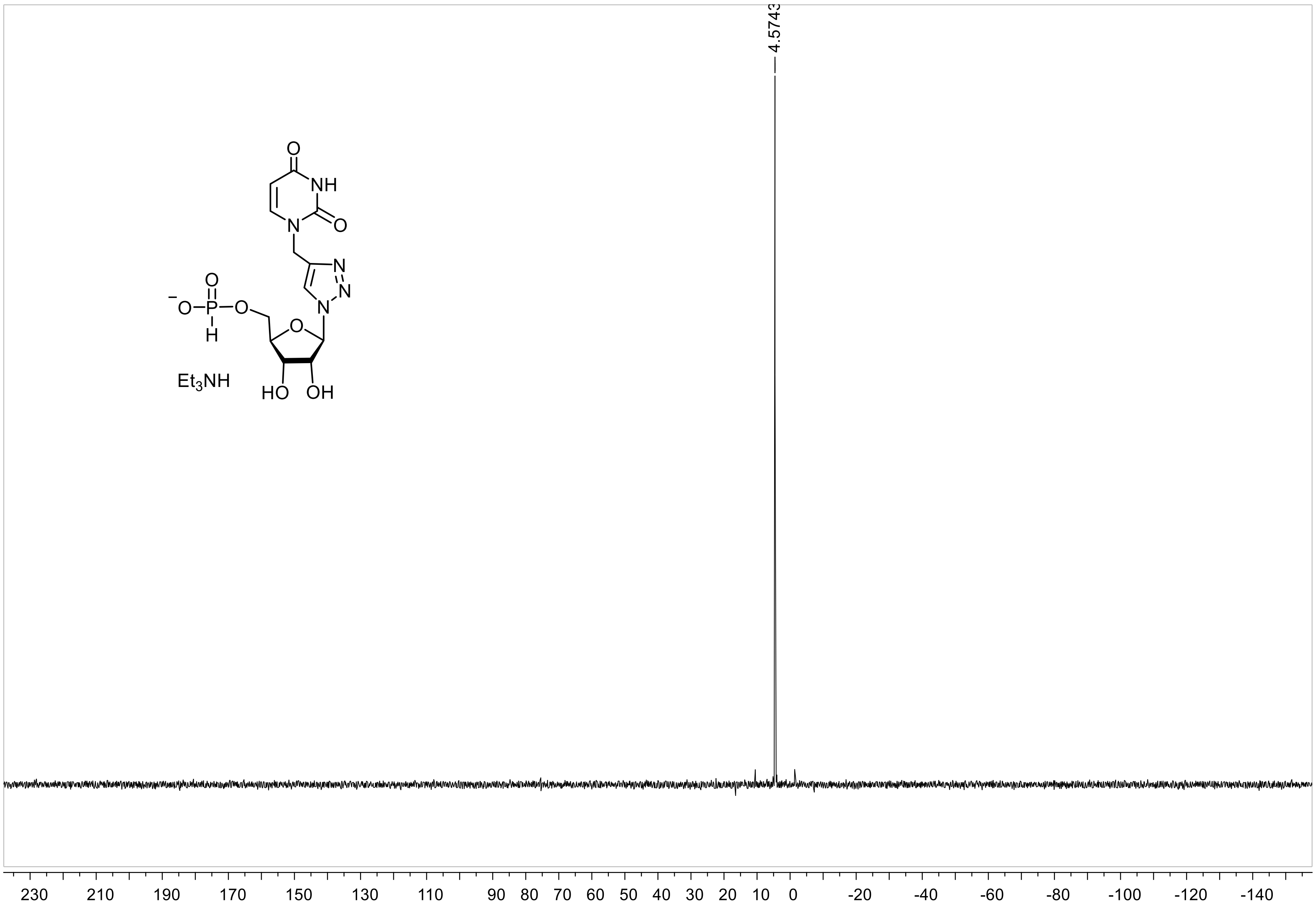

Figure S68. ^{31}P NMR spectrum of **23a** in CD_3OD

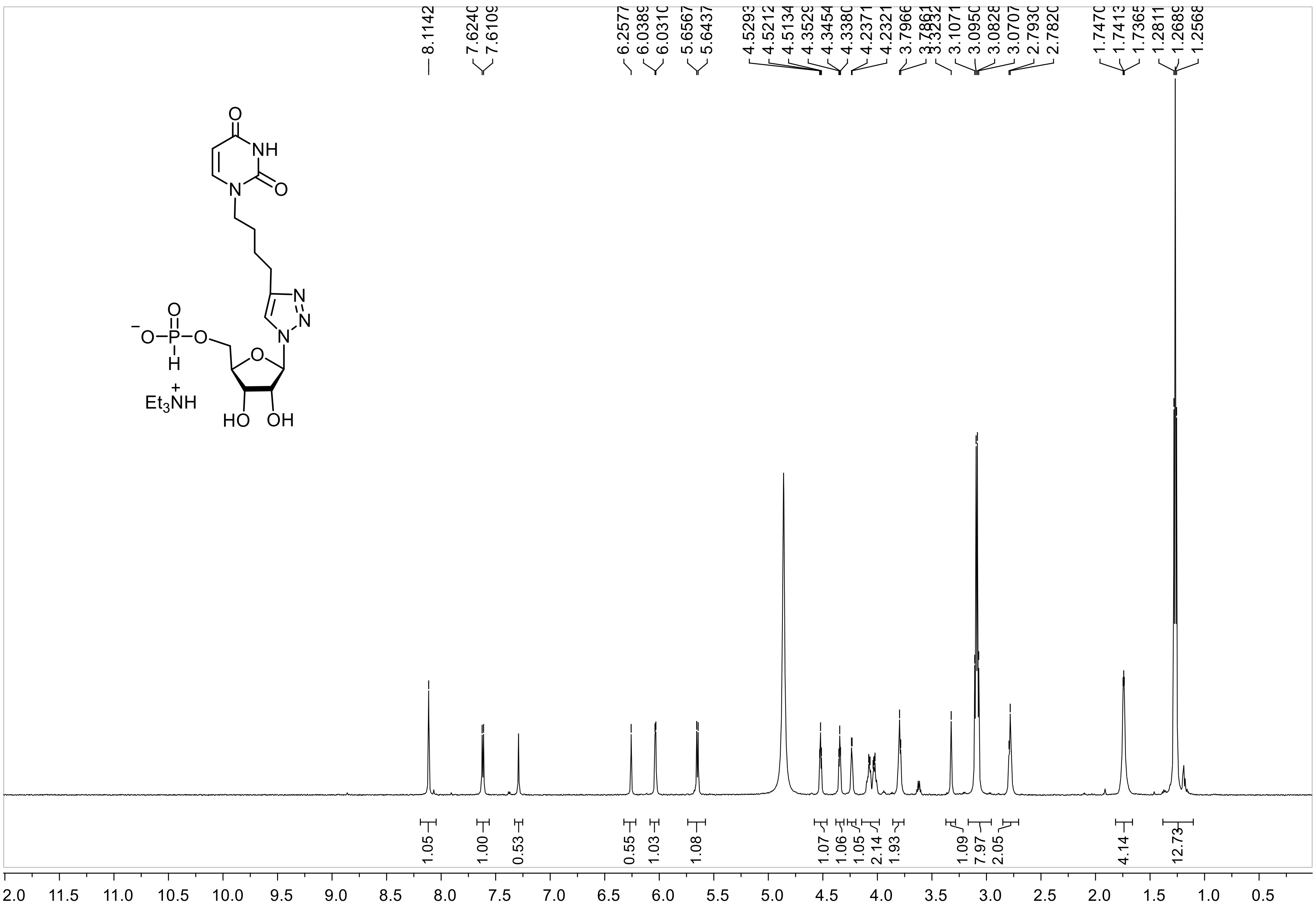

Figure S69. ^{1}H NMR spectrum of **24a** in CD_3OD

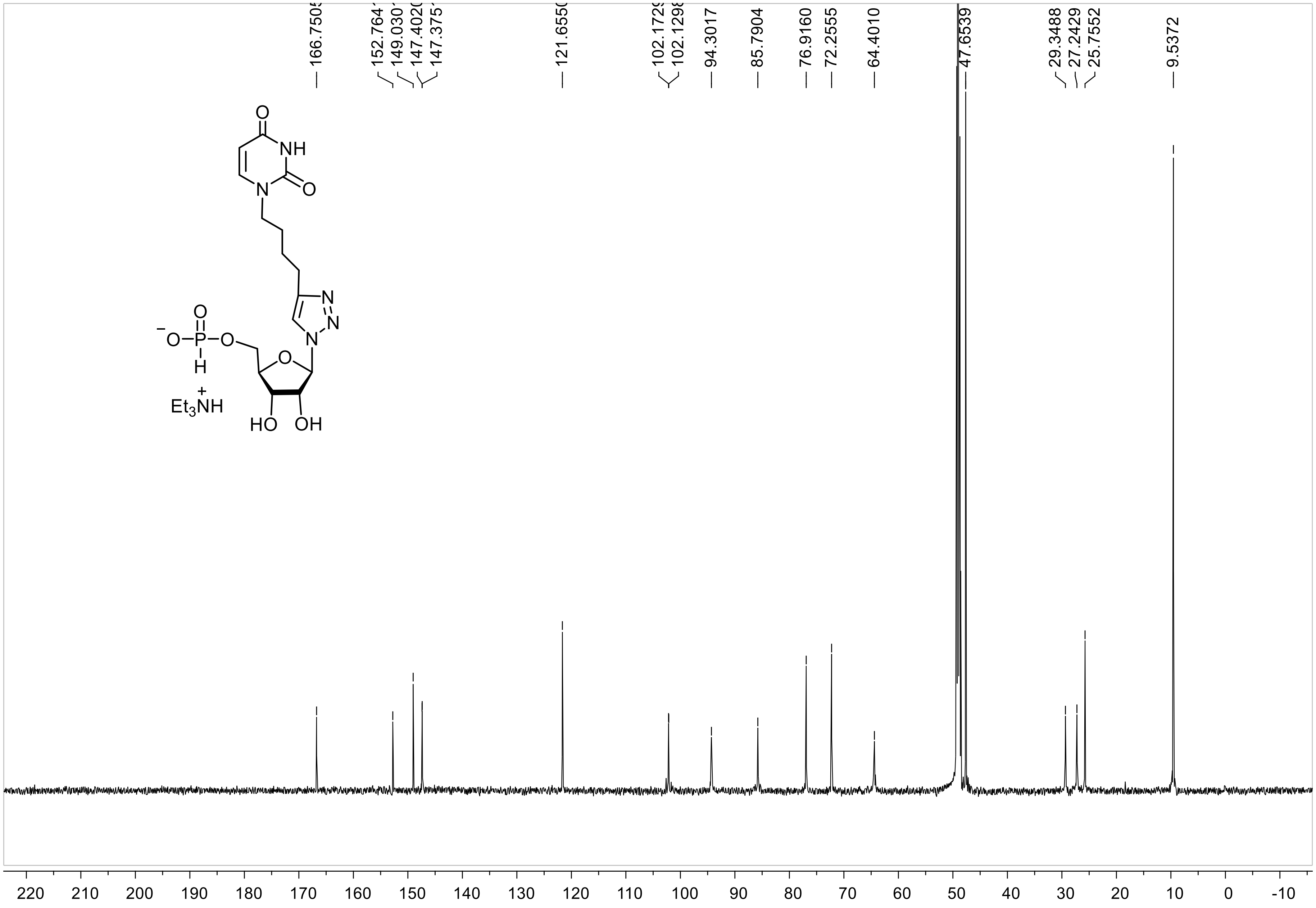

Figure S70. ^{13}C NMR spectrum of **24a** in CD_3OD

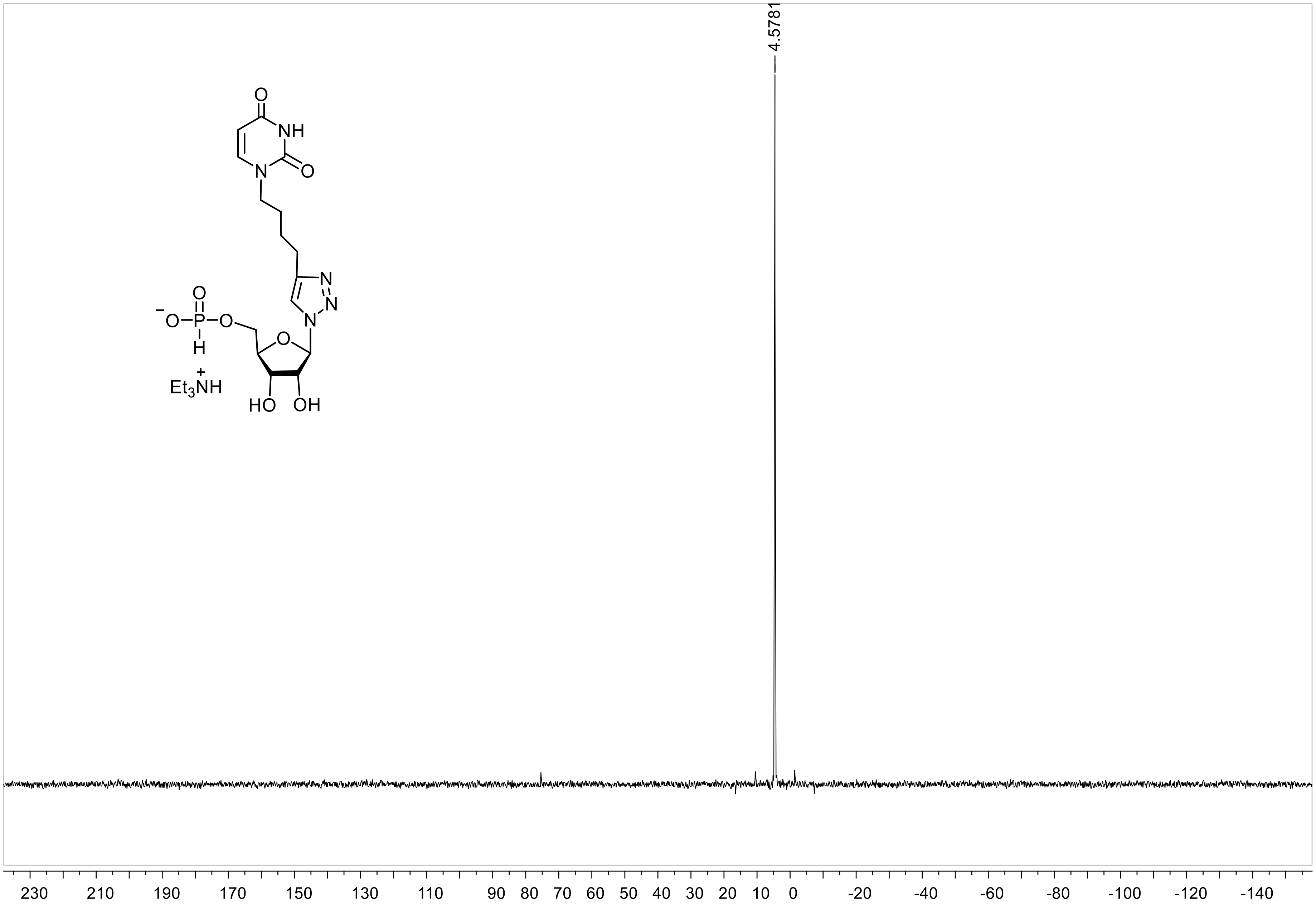

Figure S71. ^{31}P NMR spectrum of **24a** in CD_3OD

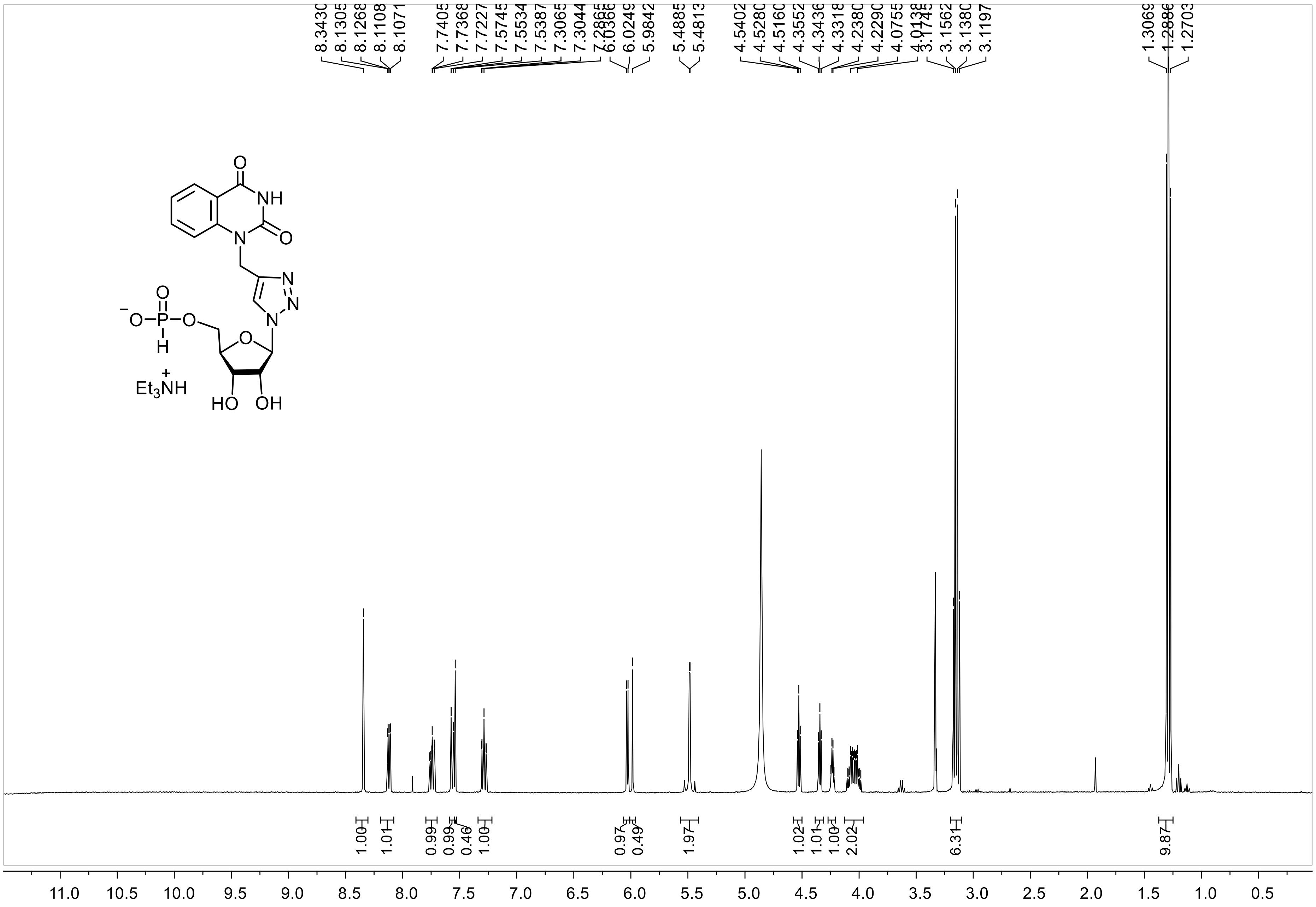

Figure S72. ^{1}H NMR spectrum of **23b** in CD_3OD

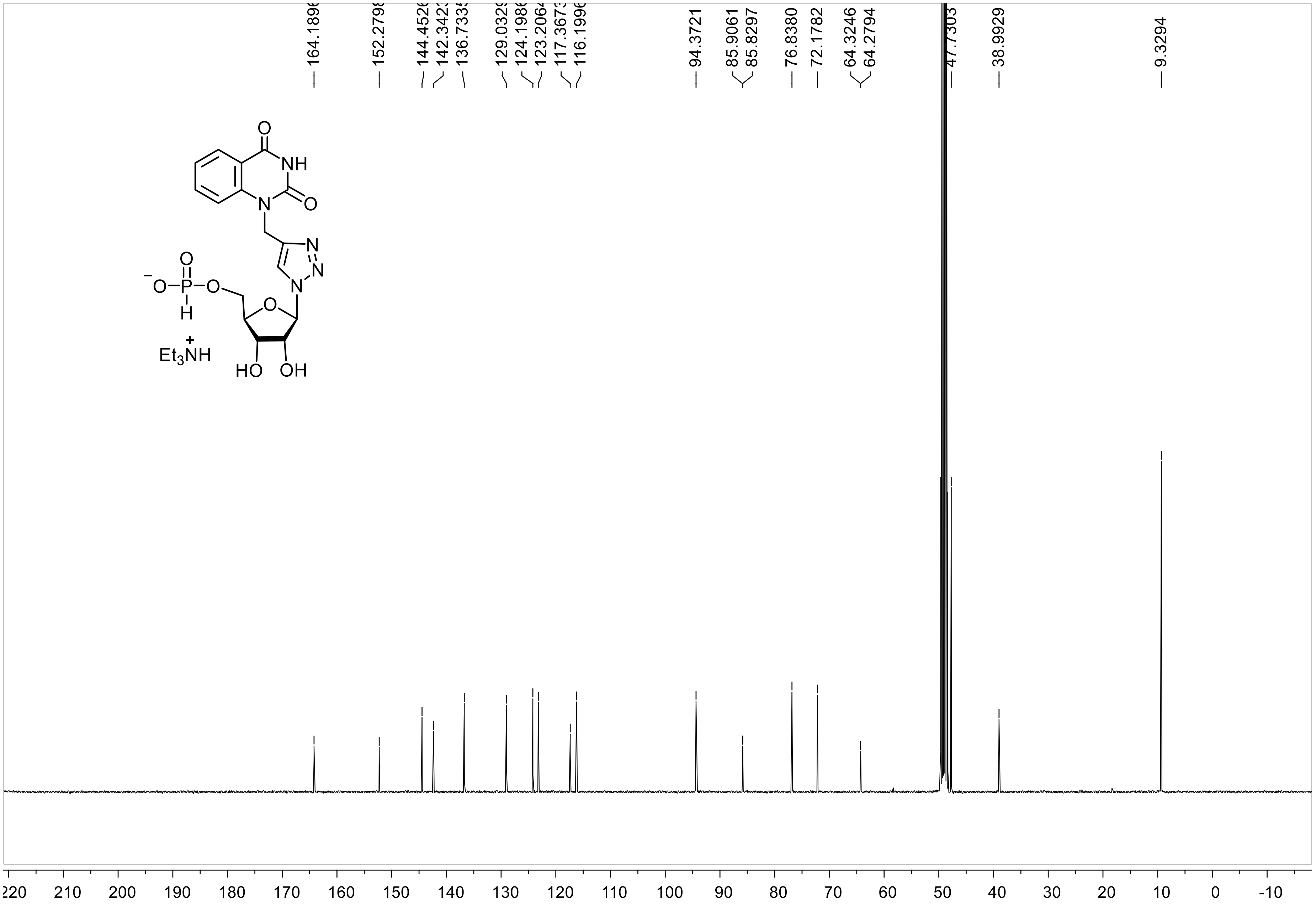

Figure S73. ^{13}C NMR spectrum of **23b** in CD_3OD

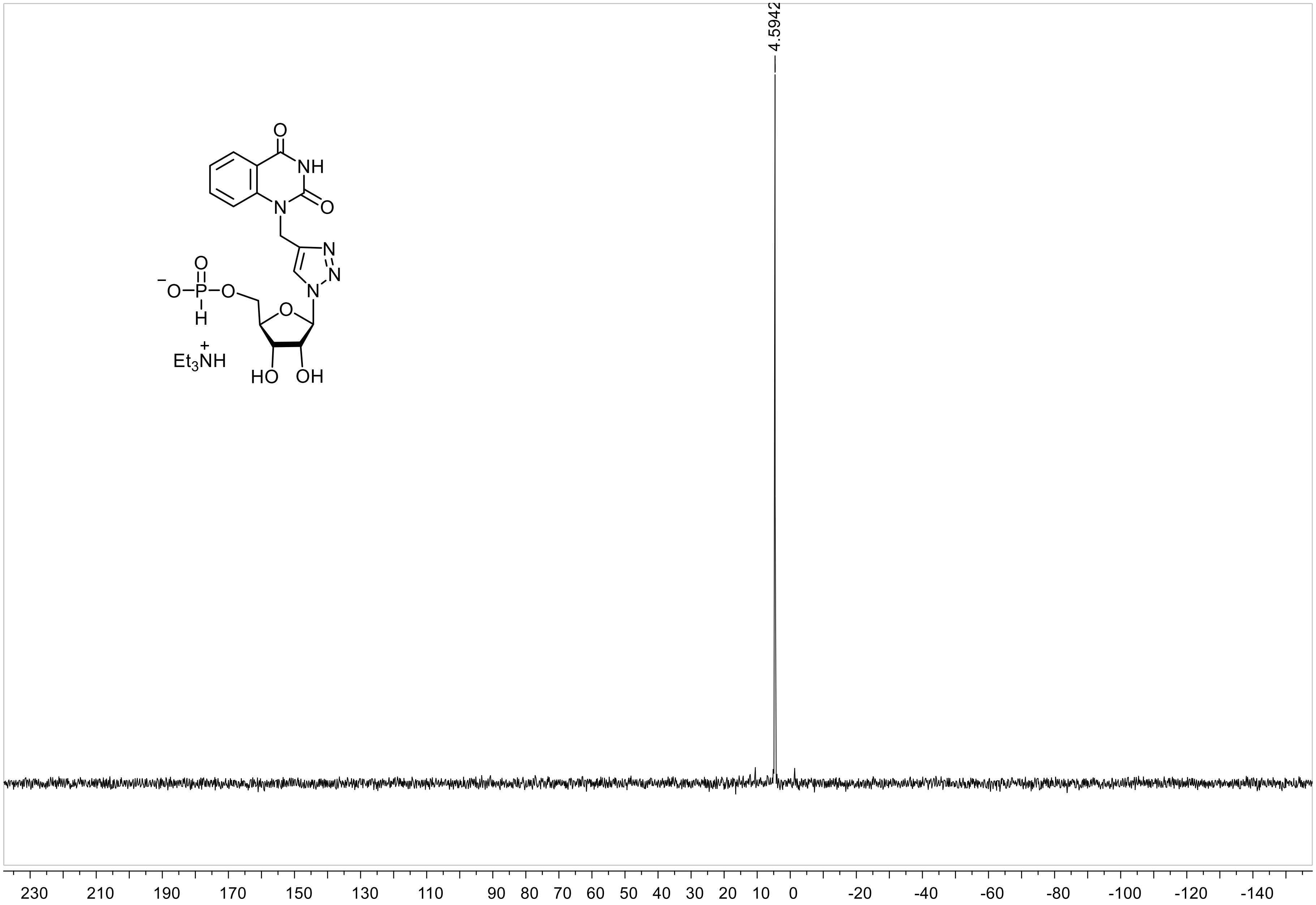

Figure S74. ^{31}P NMR spectrum of **23b** in CD_3OD

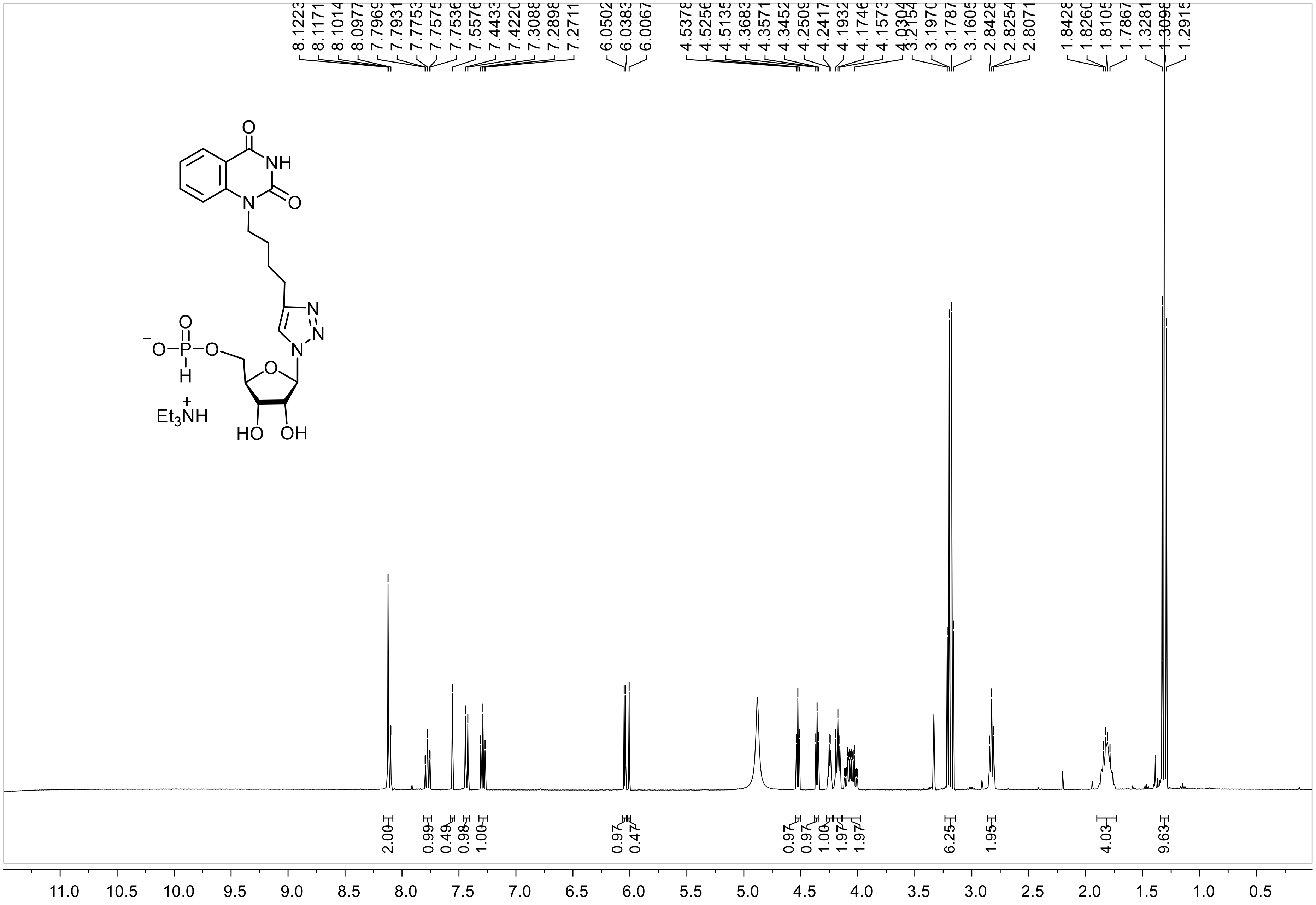

Figure S75. ^{1}H NMR spectrum of **24b** in CD_3OD

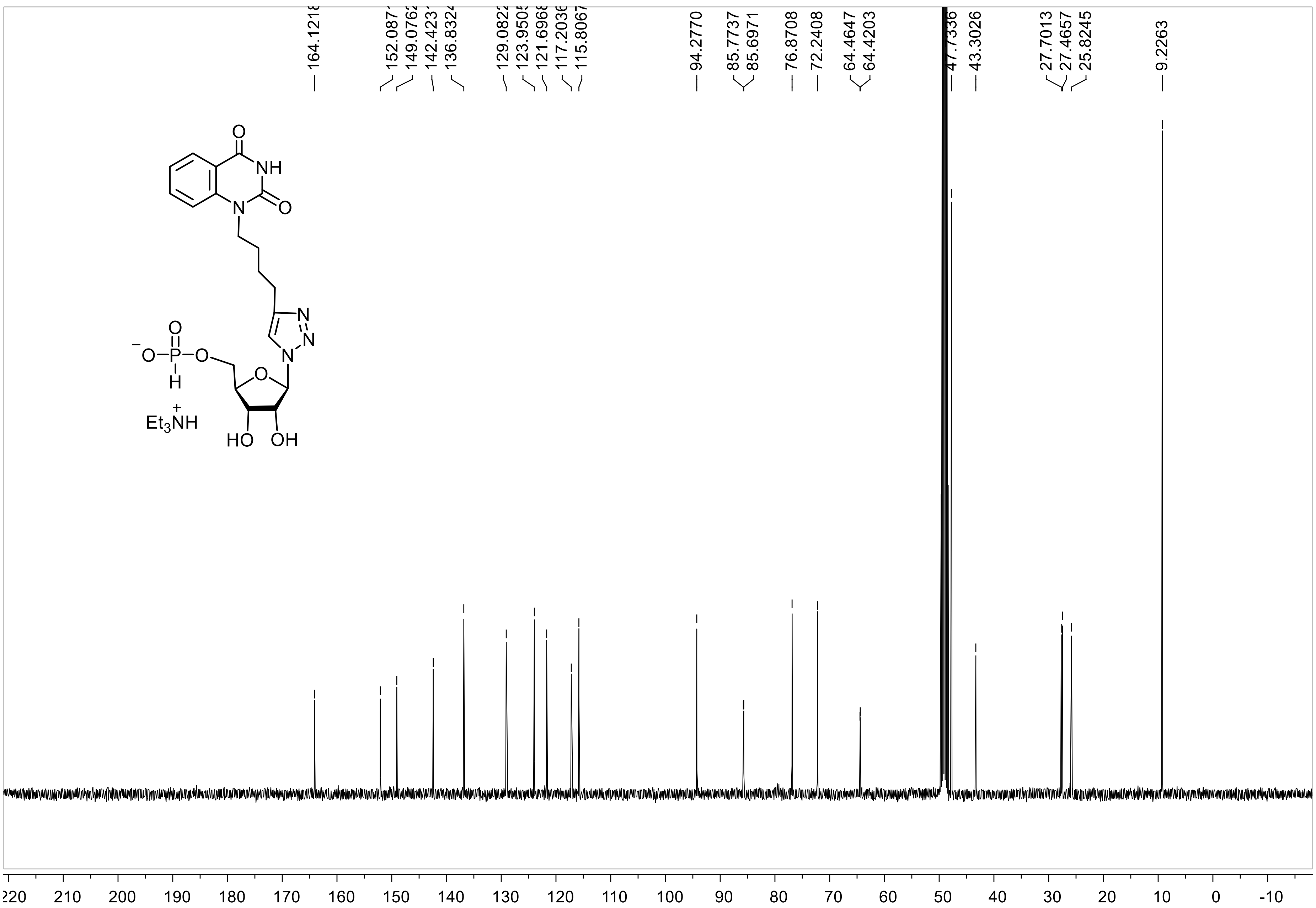

Figure S76. ^{13}C NMR spectrum of **24b** in CD_3OD

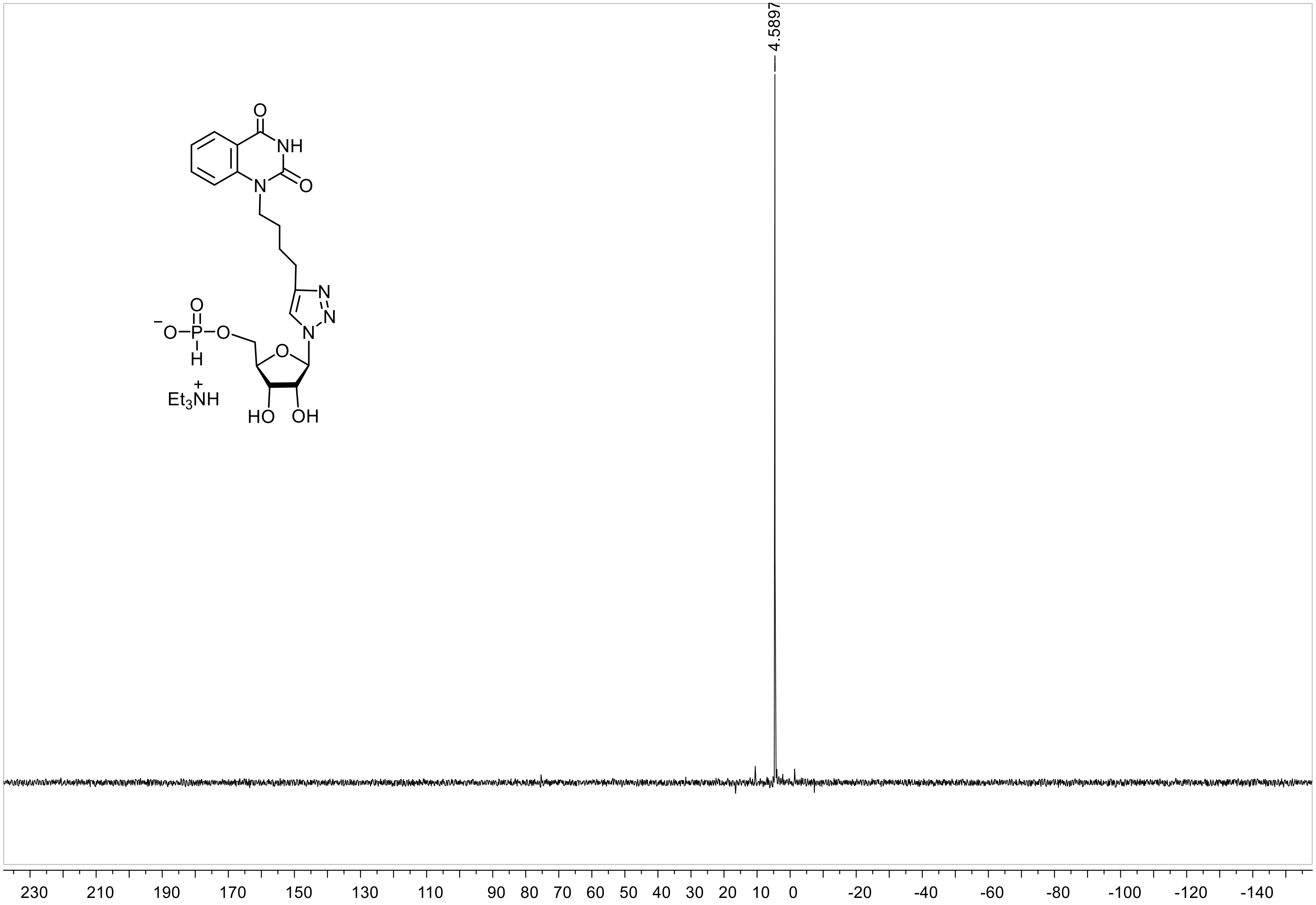

Figure S77. ^{31}P NMR spectrum of **24b** in CD_3OD

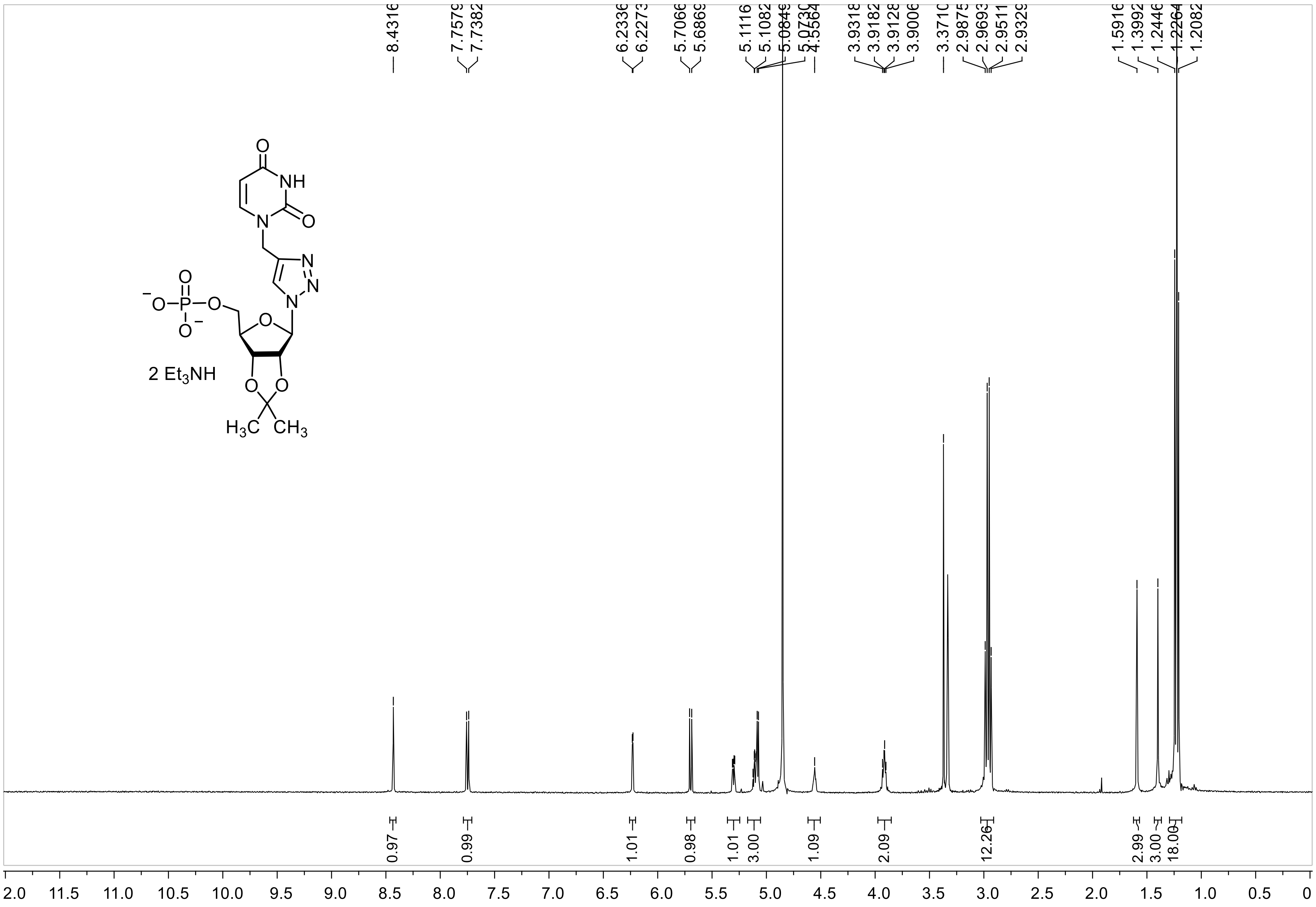

Figure S78. ^{1}H NMR spectrum of **31a** in $CDCl_3$

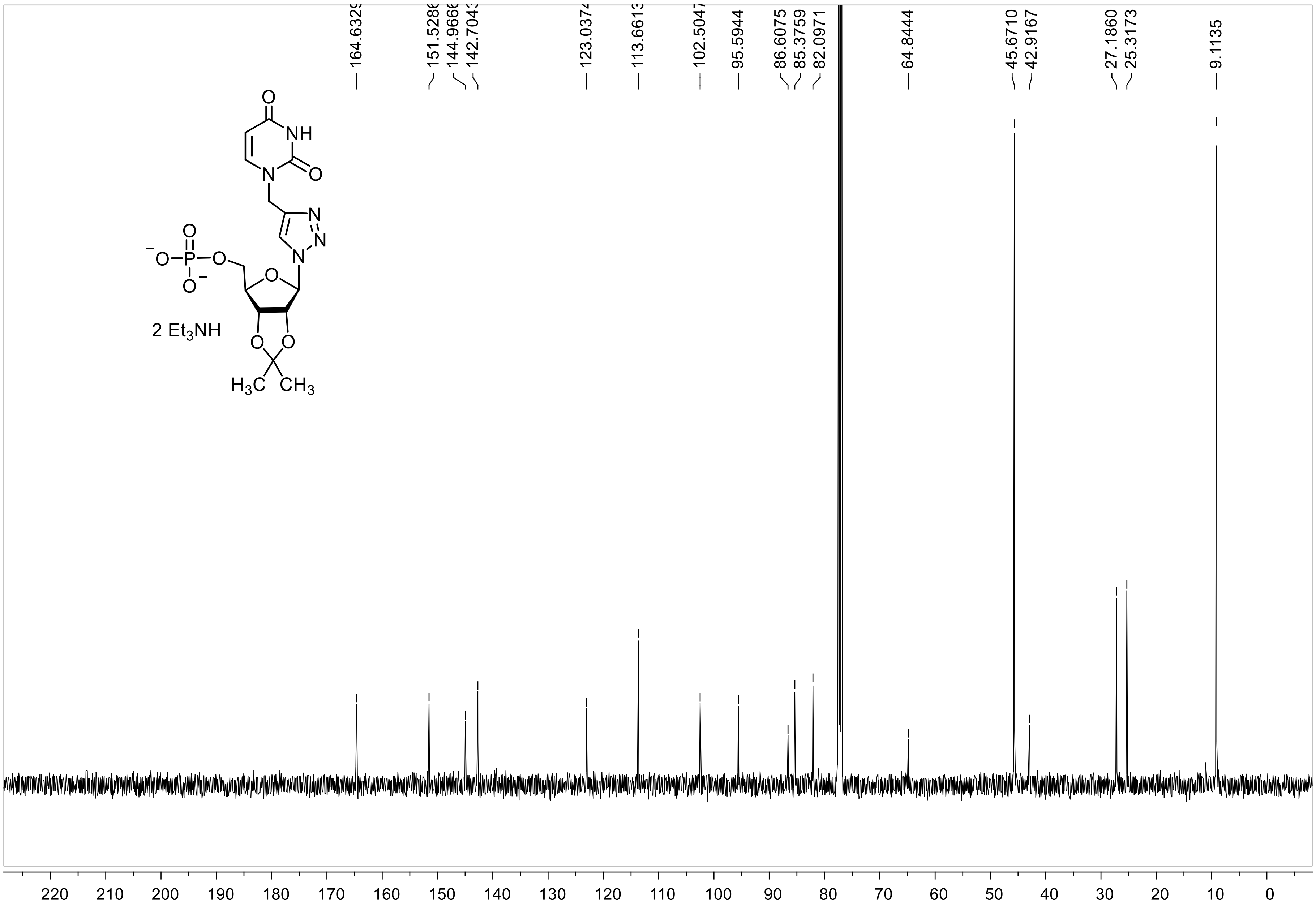

Figure S79. ^{13}C NMR spectrum of **31a** in $CDCl_3$

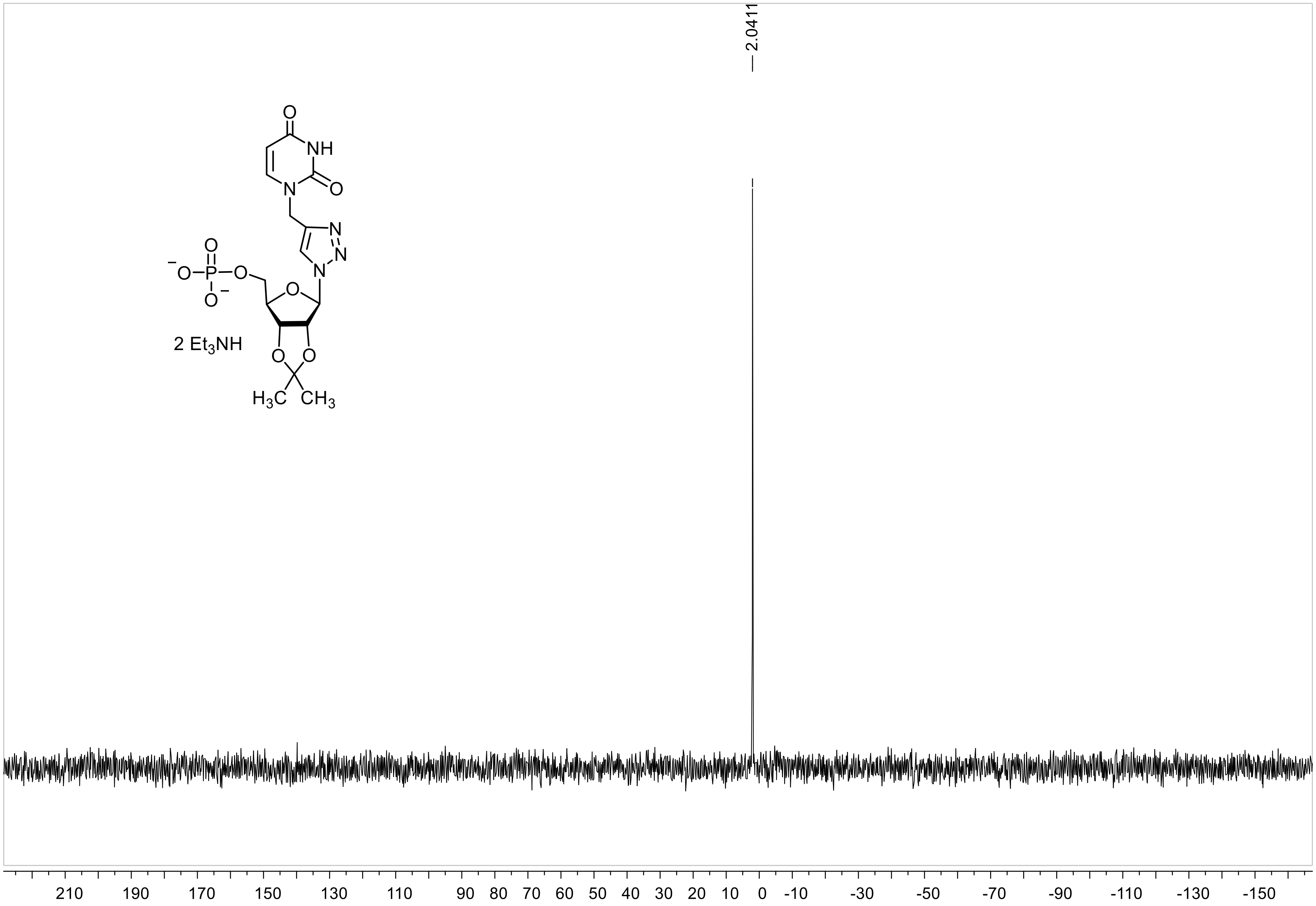

Figure S80. ^{31}P NMR spectrum of **31a** in $CDCl_3$

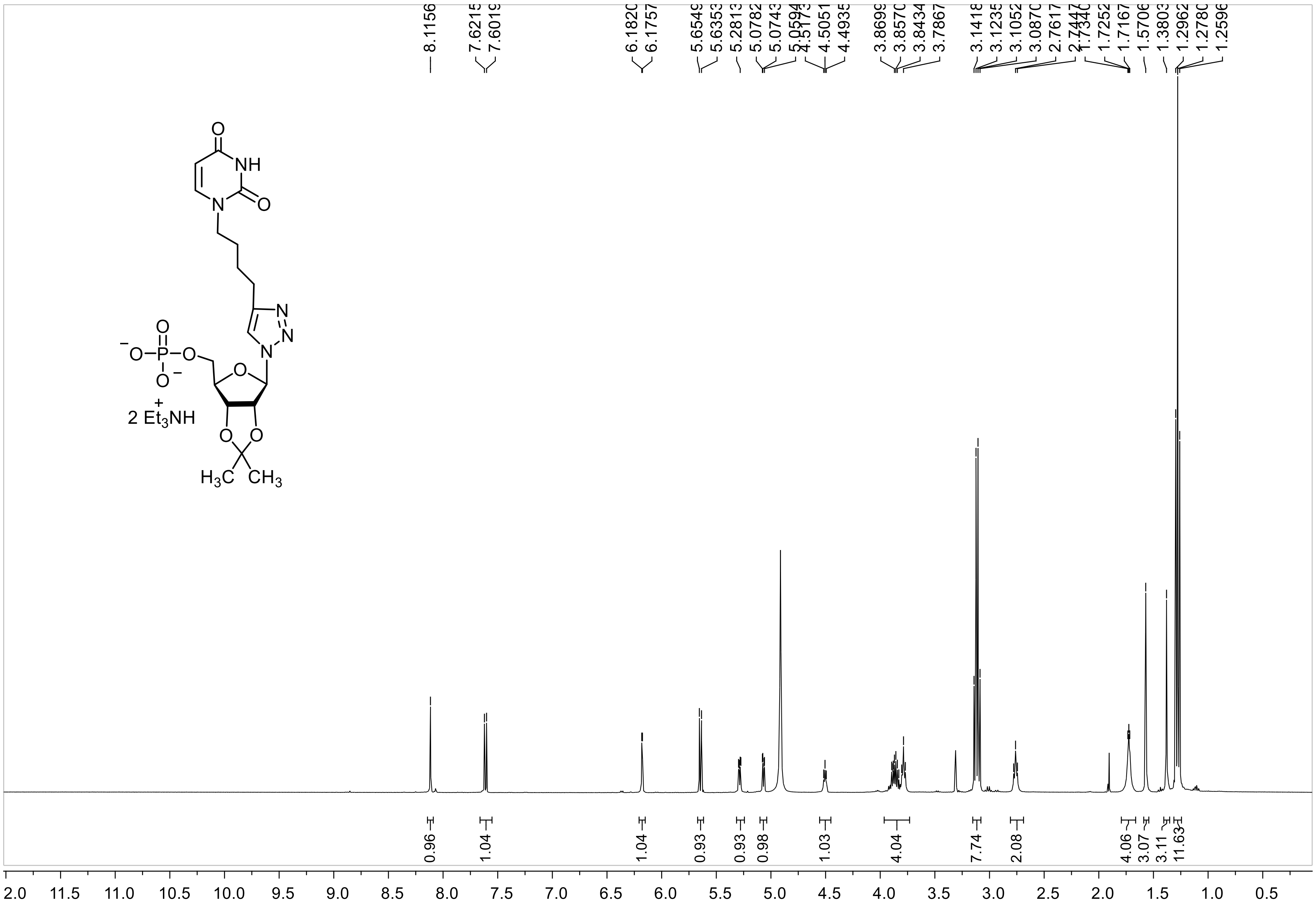

Figure S81. ^{1}H NMR spectrum of **32a** in CD_3OD

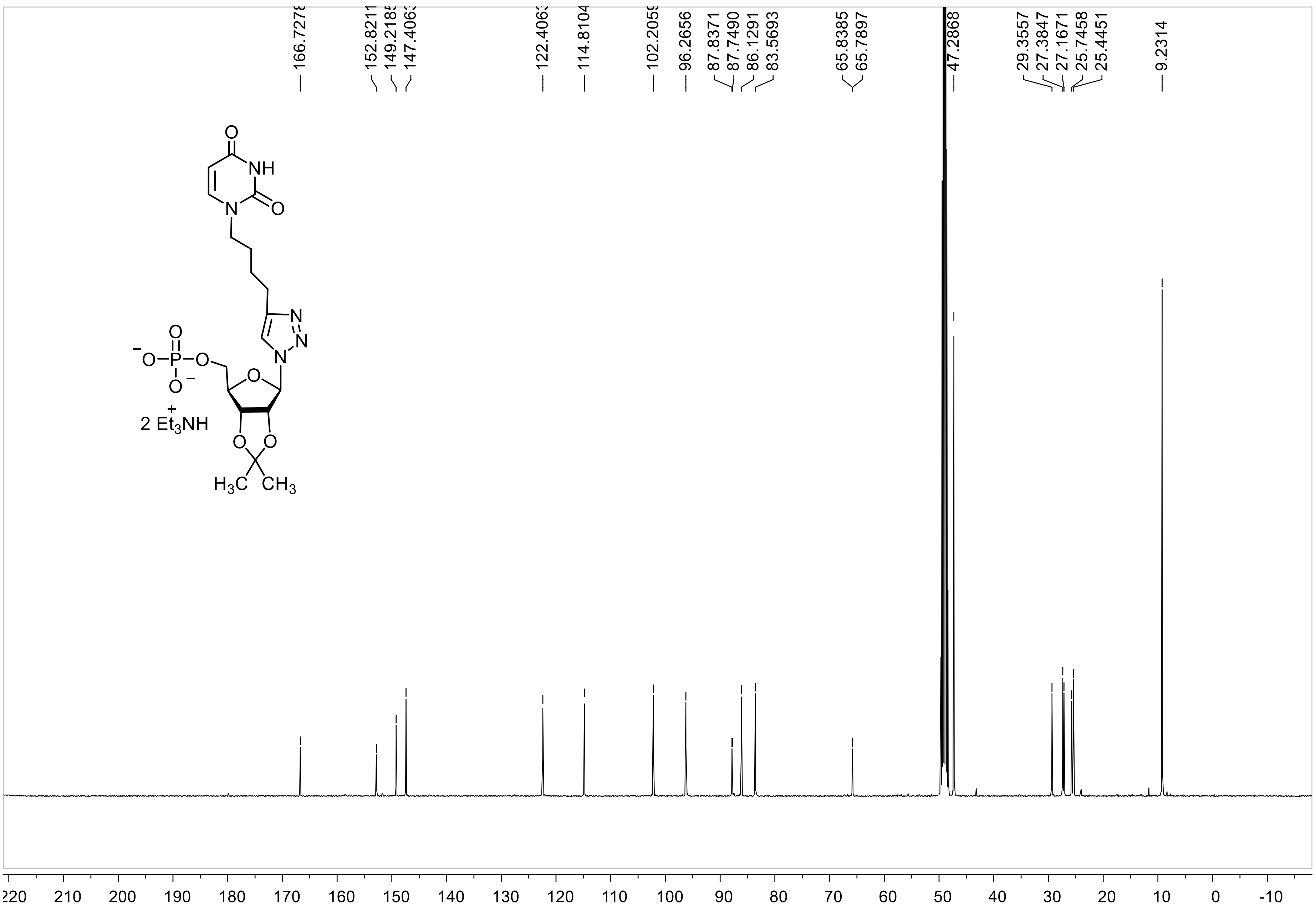

Figure S82. ^{13}C NMR spectrum of **32a** in CD_3OD

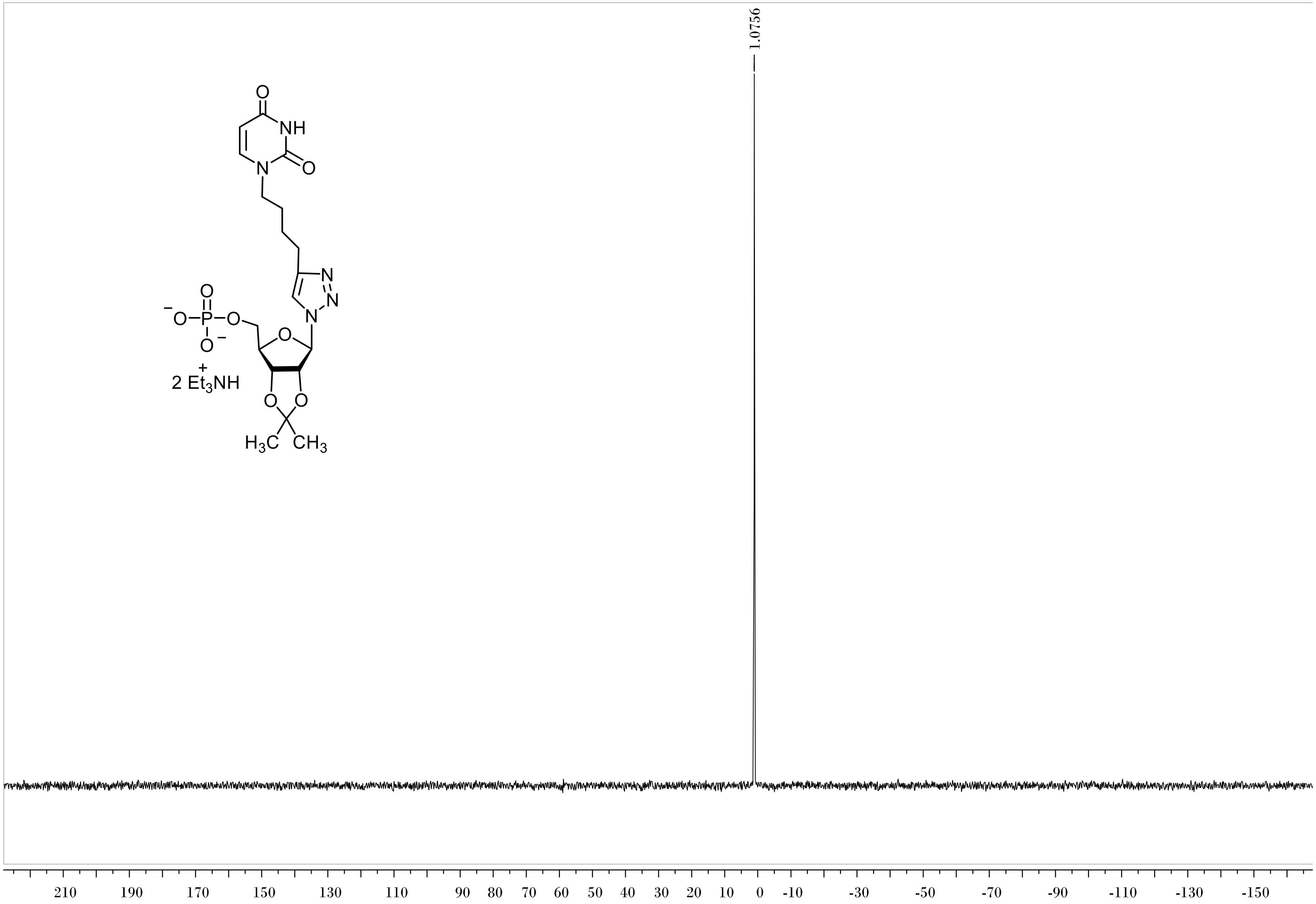

Figure S83. ^{31}P NMR spectrum of **32a** in CD_3OD

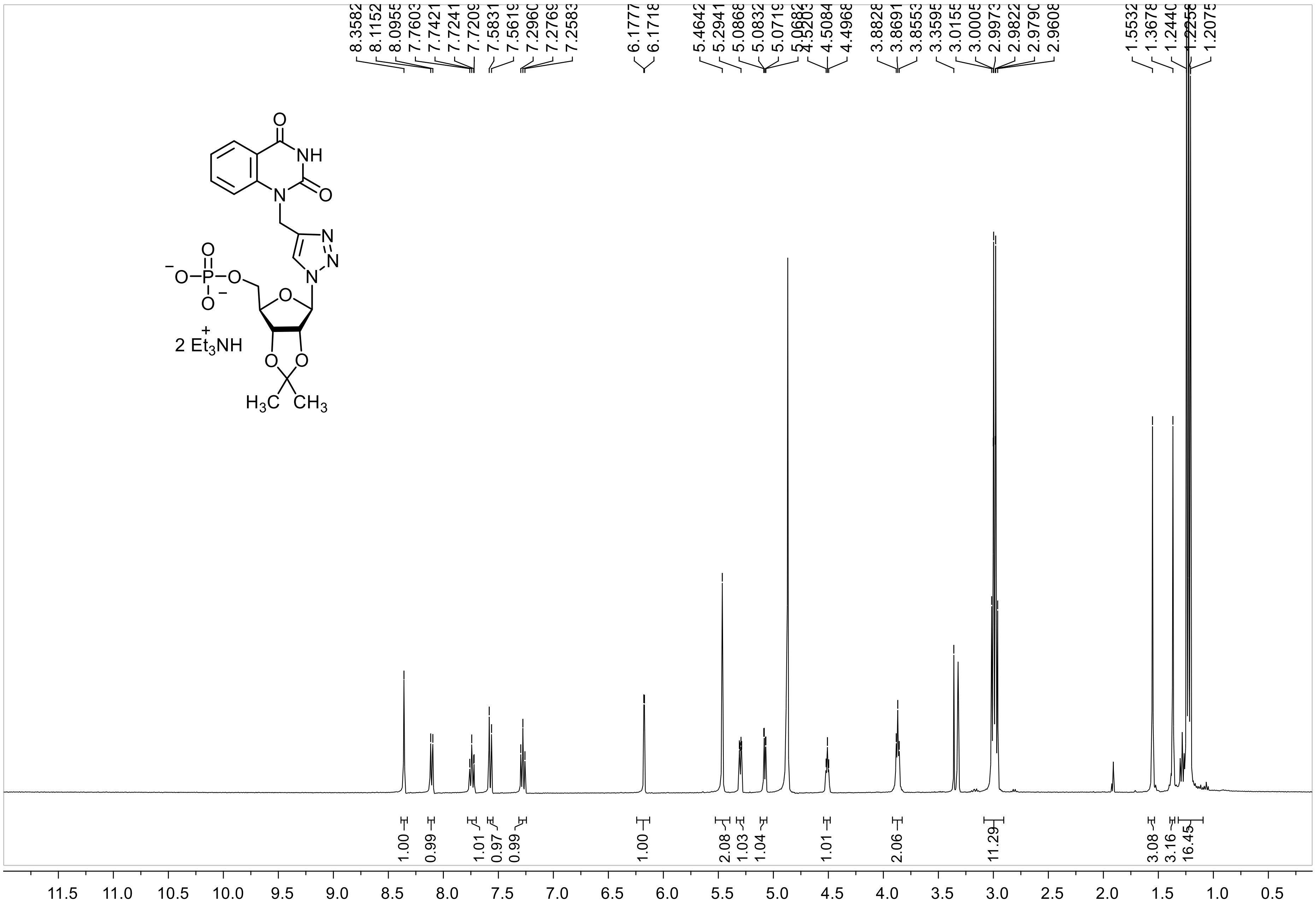

Figure S84. ^{1}H NMR spectrum of **31b** in CD_3OD

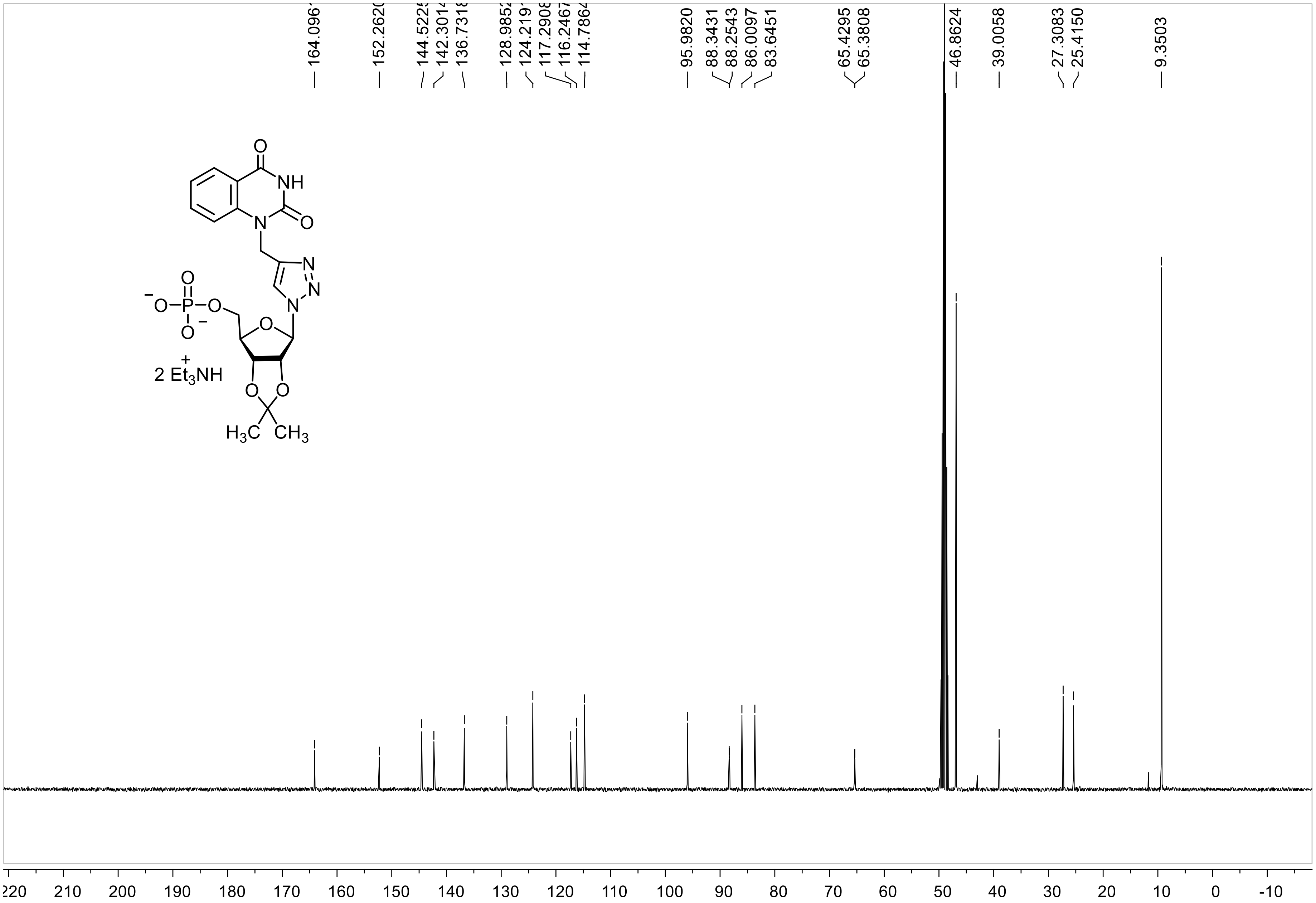

Figure S85. ^{13}C NMR spectrum of **31b** in CD_3OD

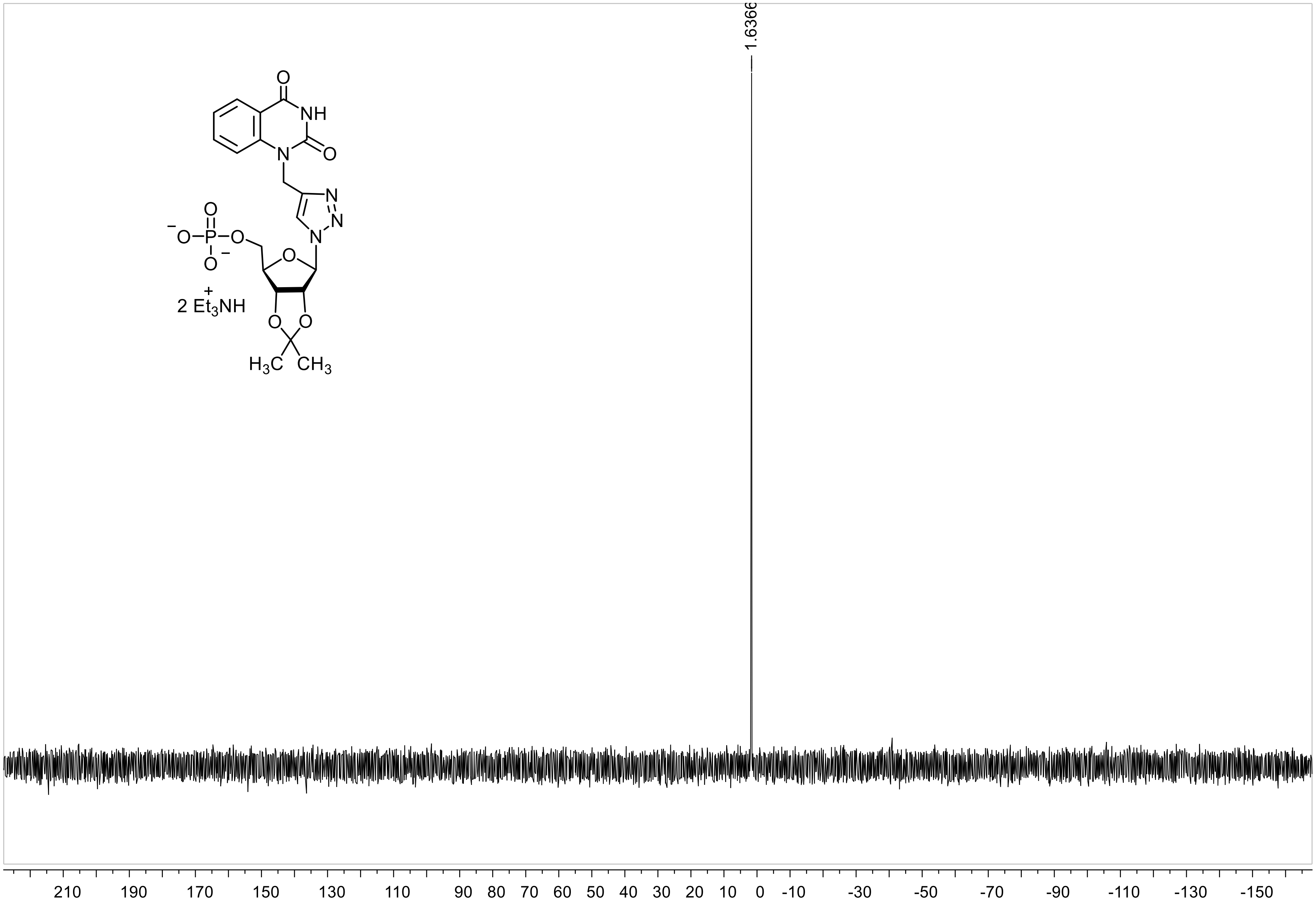

Figure S86. ^{31}P NMR spectrum of **31b** in CD_3OD

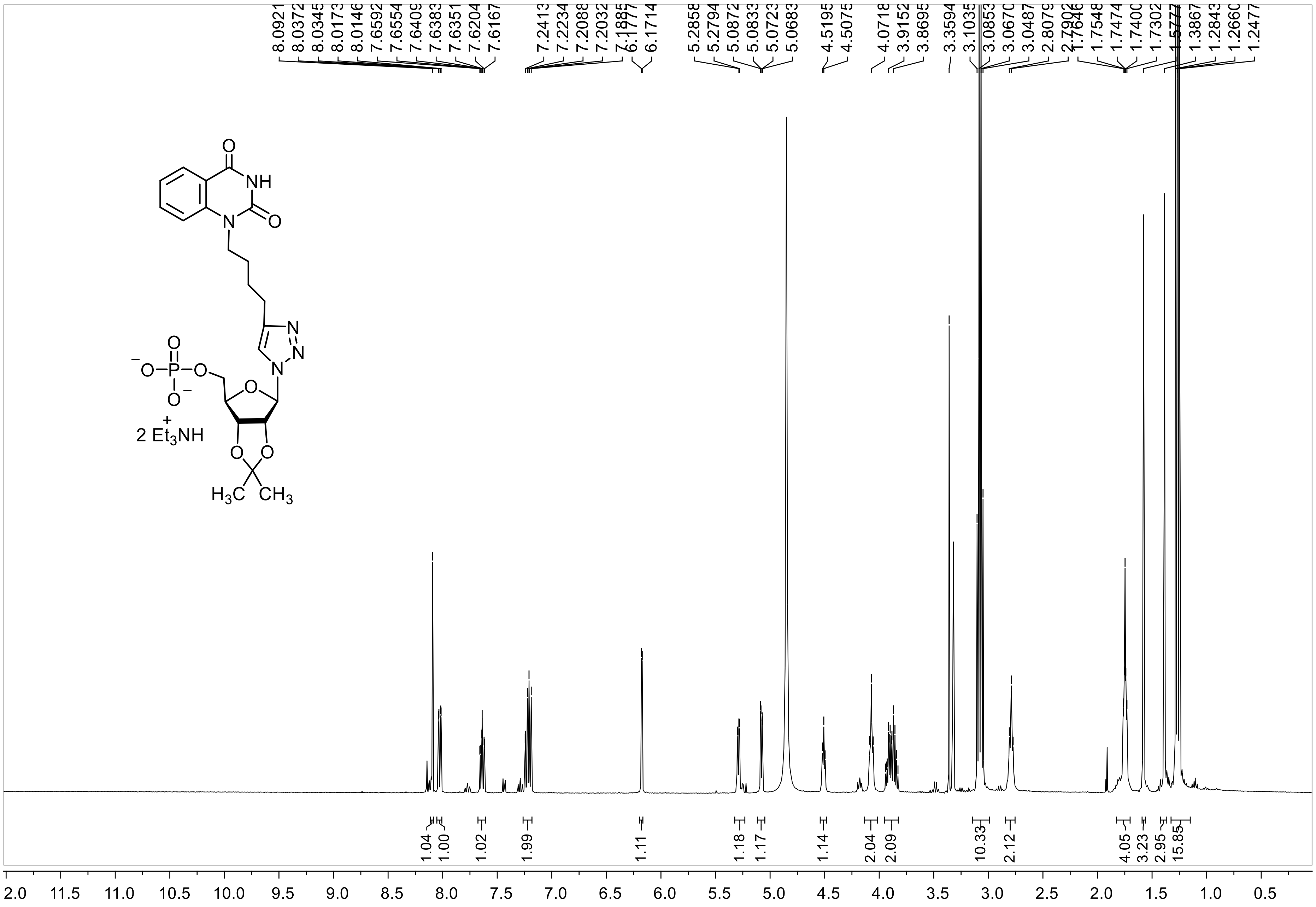

Figure S87. ^{1}H NMR spectrum of **32b** in CD_3OD

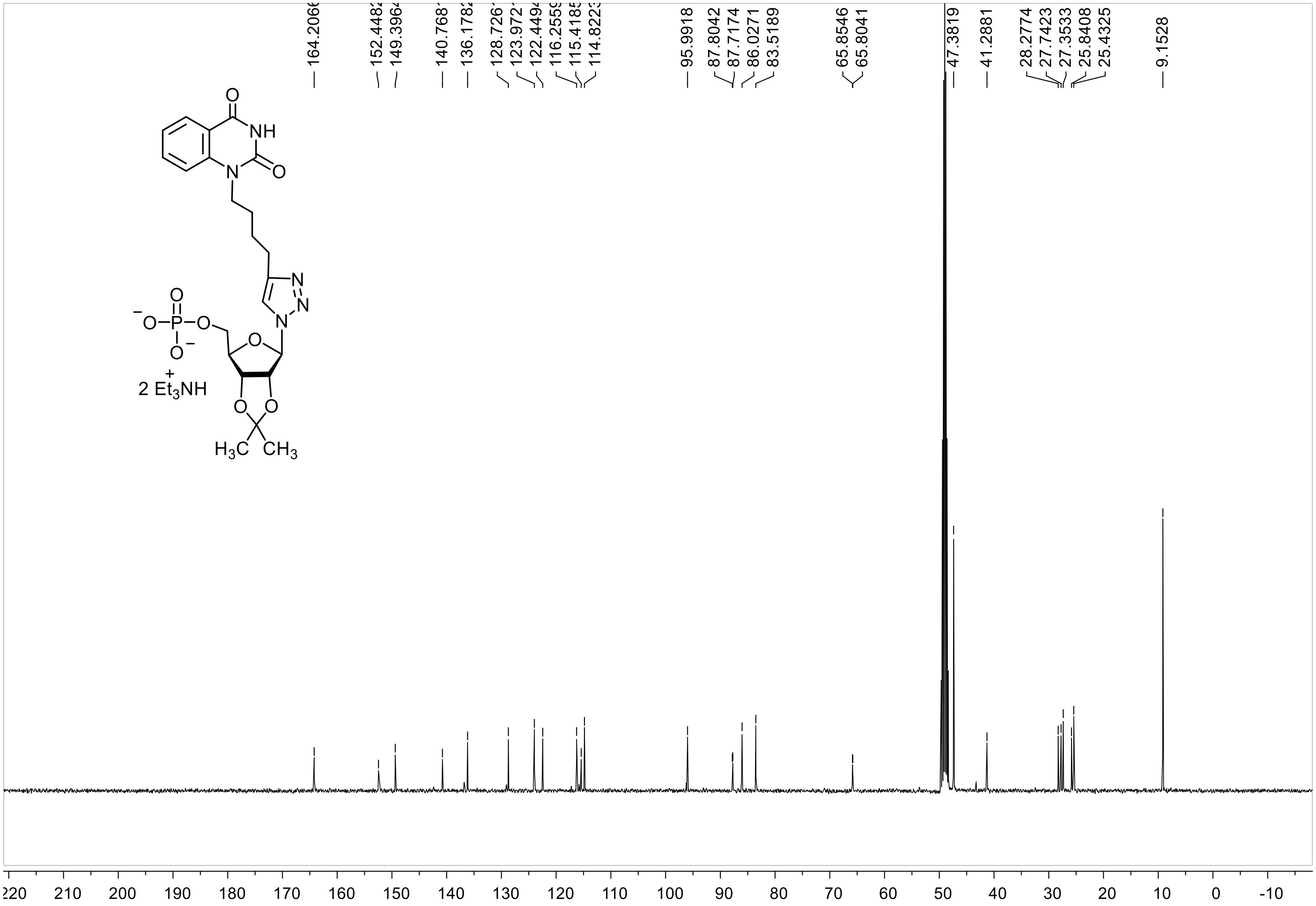

Figure S88. ^{13}C NMR spectrum of **32b** in CD_3OD

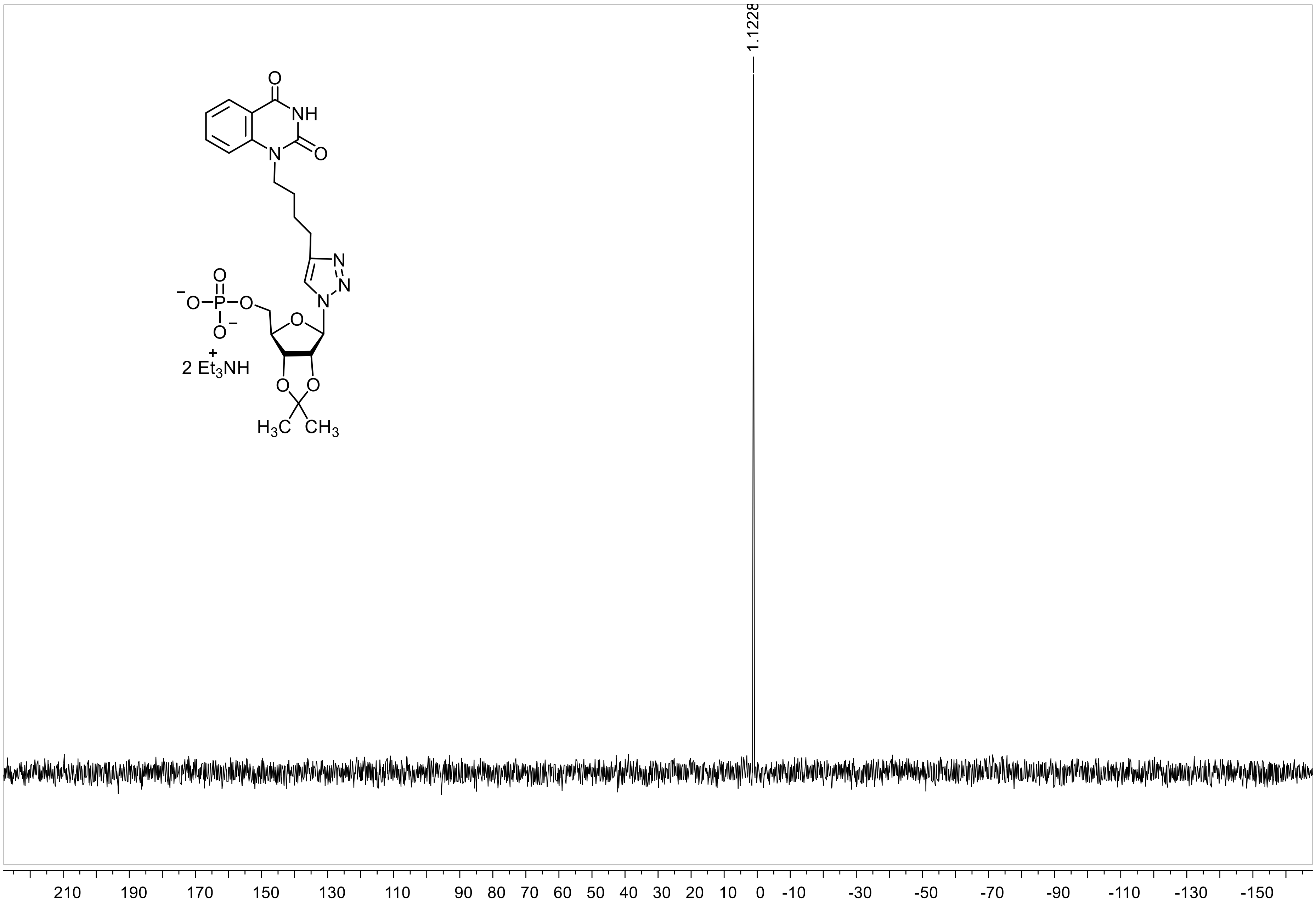

Figure S89. ^{31}P NMR spectrum of **32b** in CD_3OD

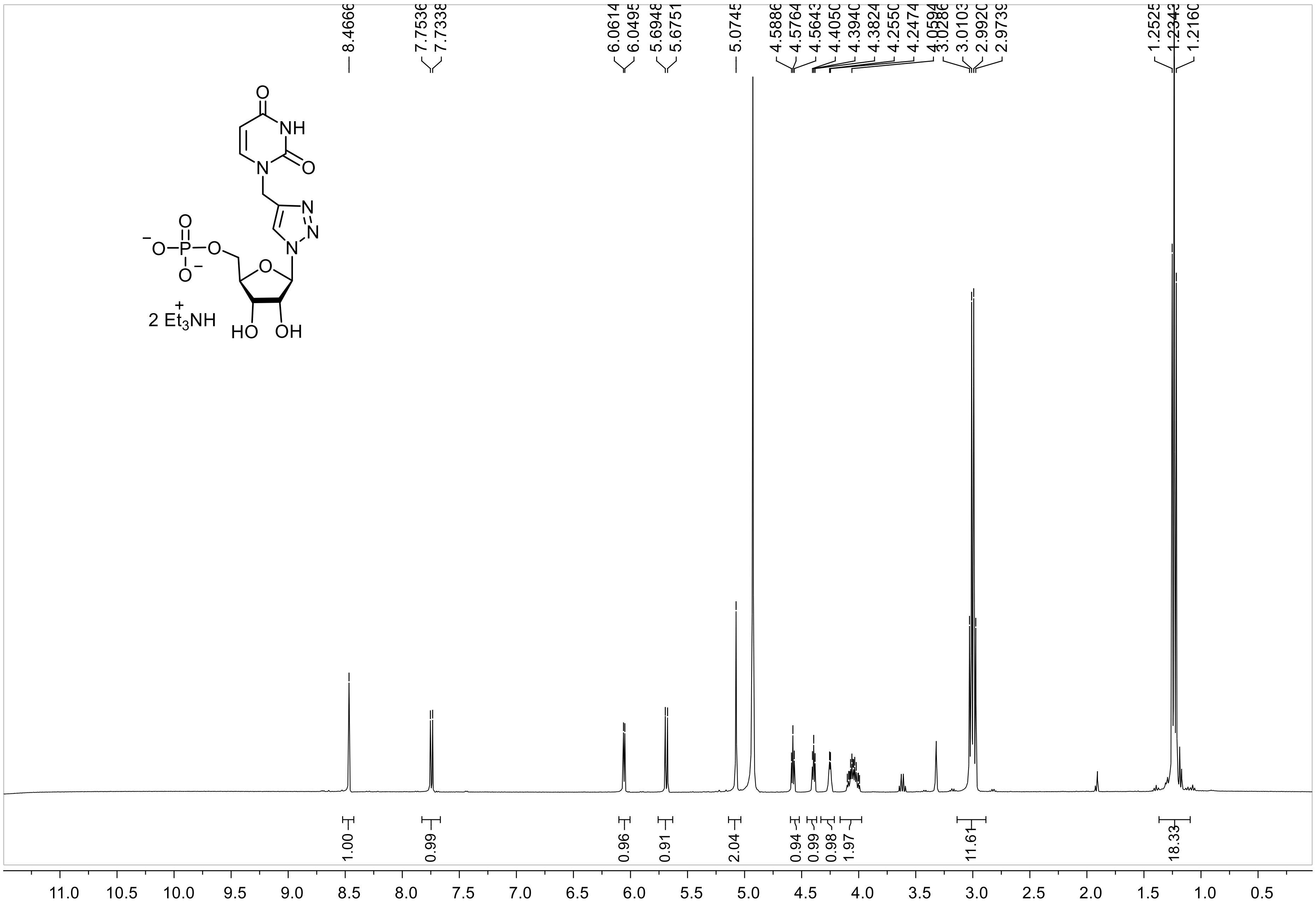

Figure S90. ^{1}H NMR spectrum of **25a** in CD_3OD

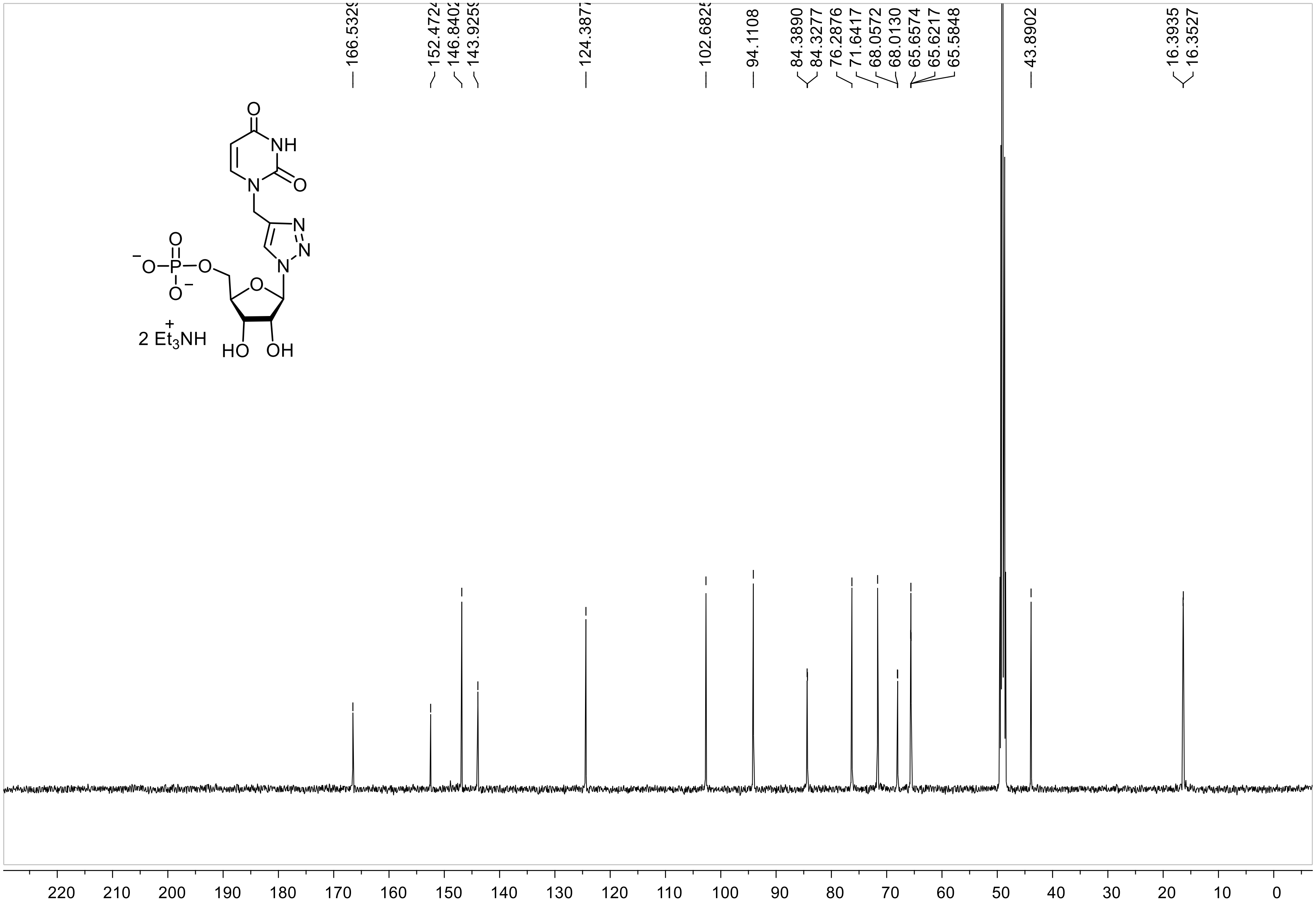

Figure S91. ^{13}C NMR spectrum of **25a** in CD_3OD

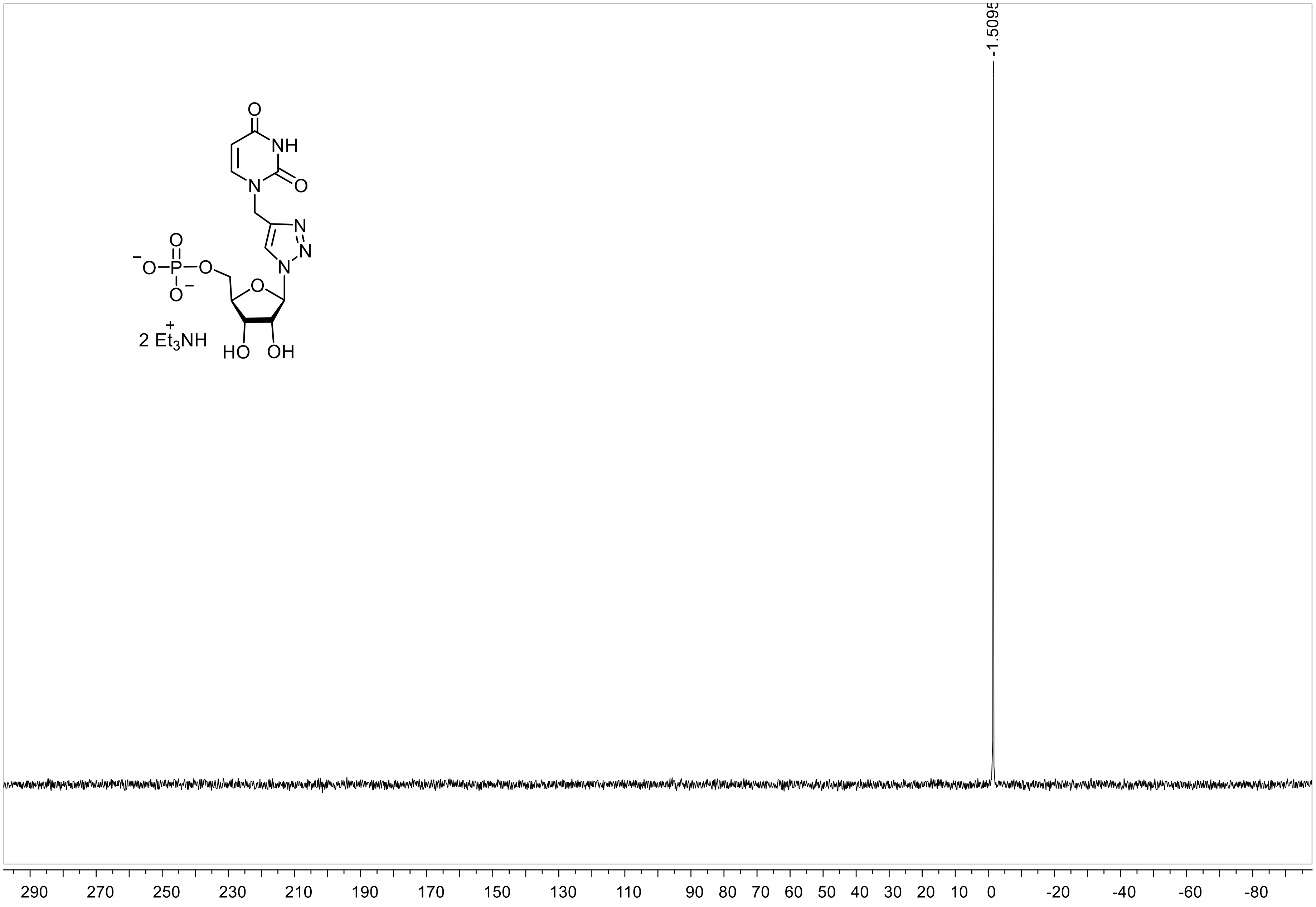

Figure S92. ^{31}P NMR spectrum of **25a** in CD_3OD

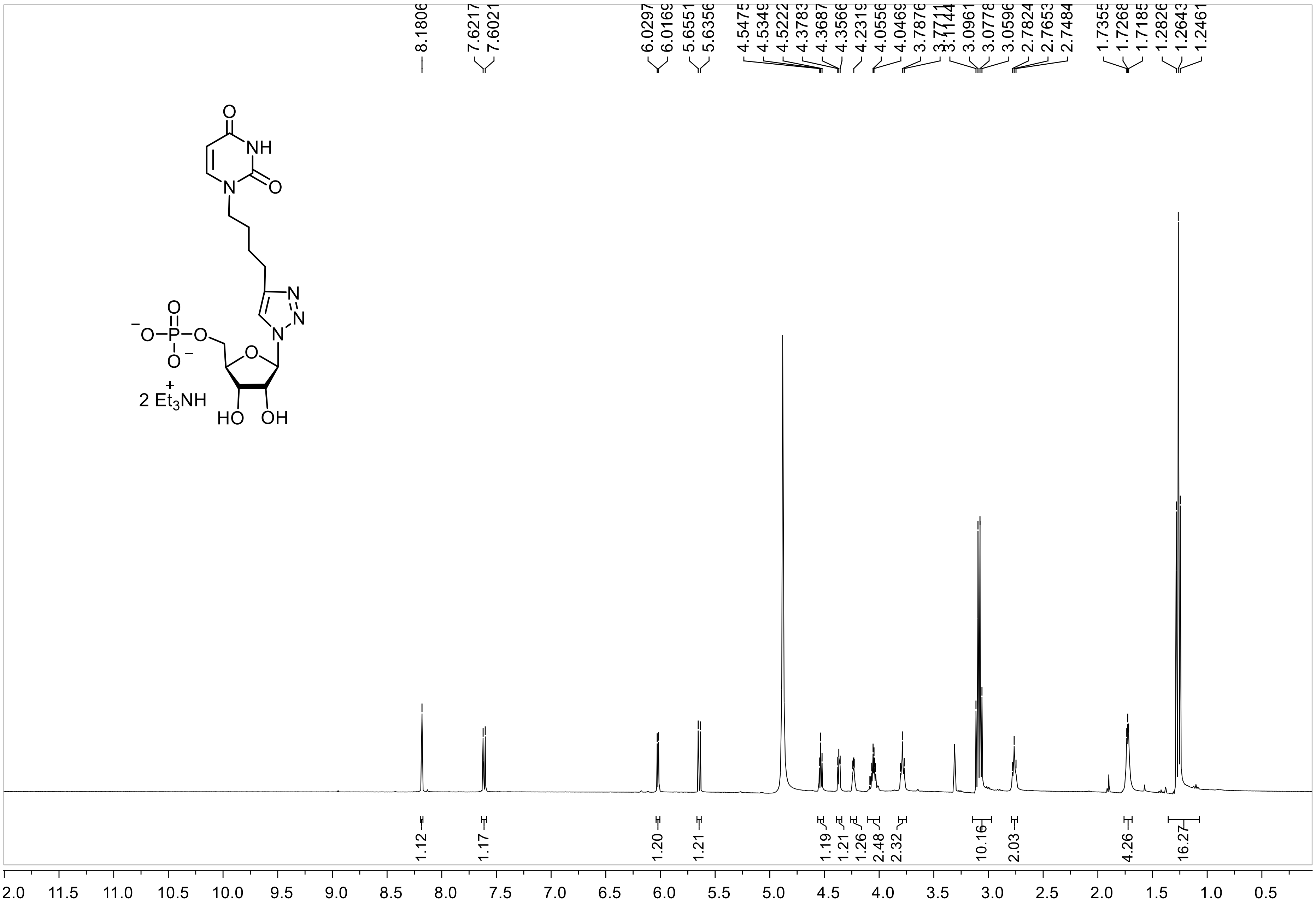

Figure S93. ^{1}H NMR spectrum of **26a** in CD_3OD

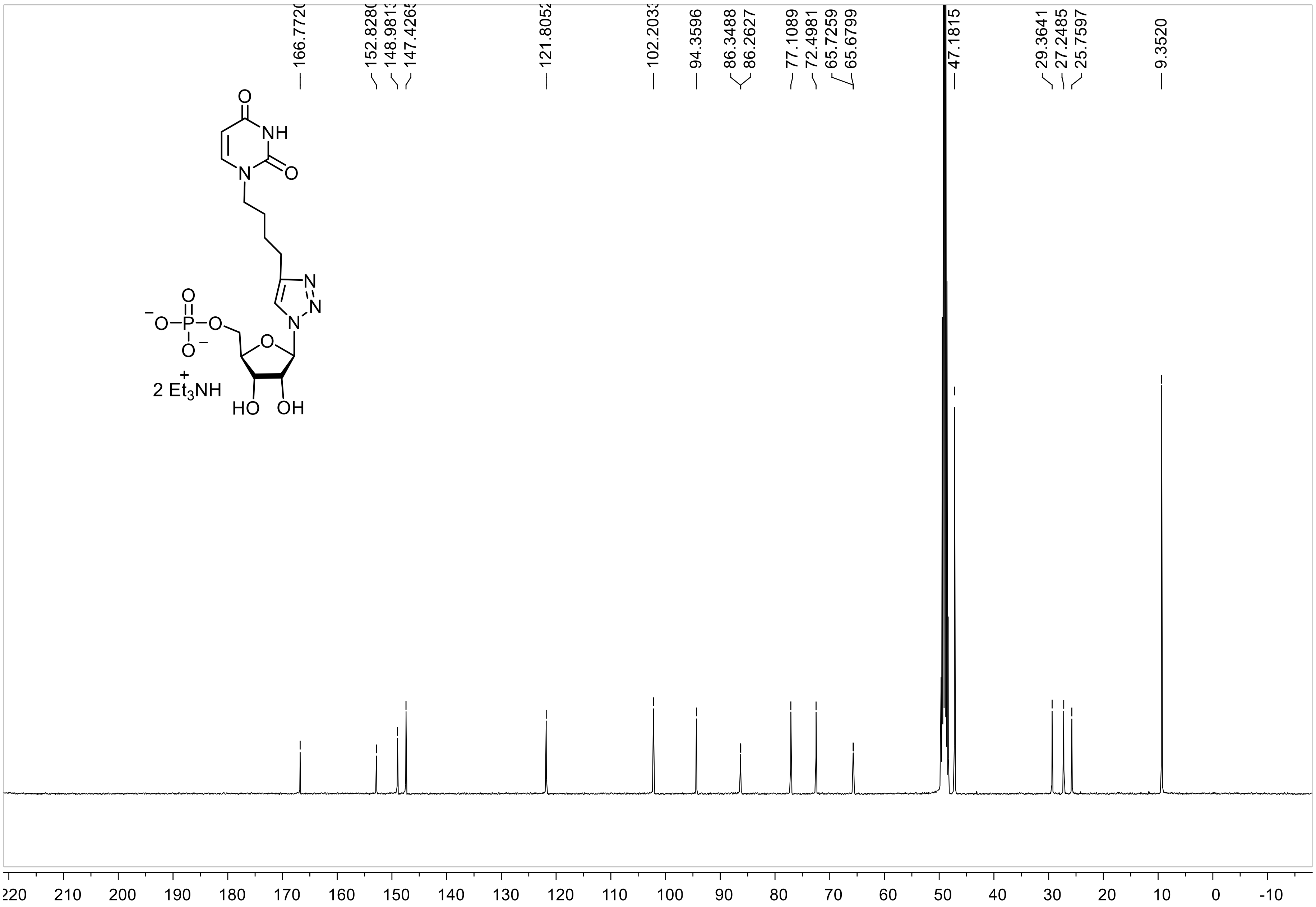

Figure S94. ^{13}C NMR spectrum of **26a** in CD_3OD

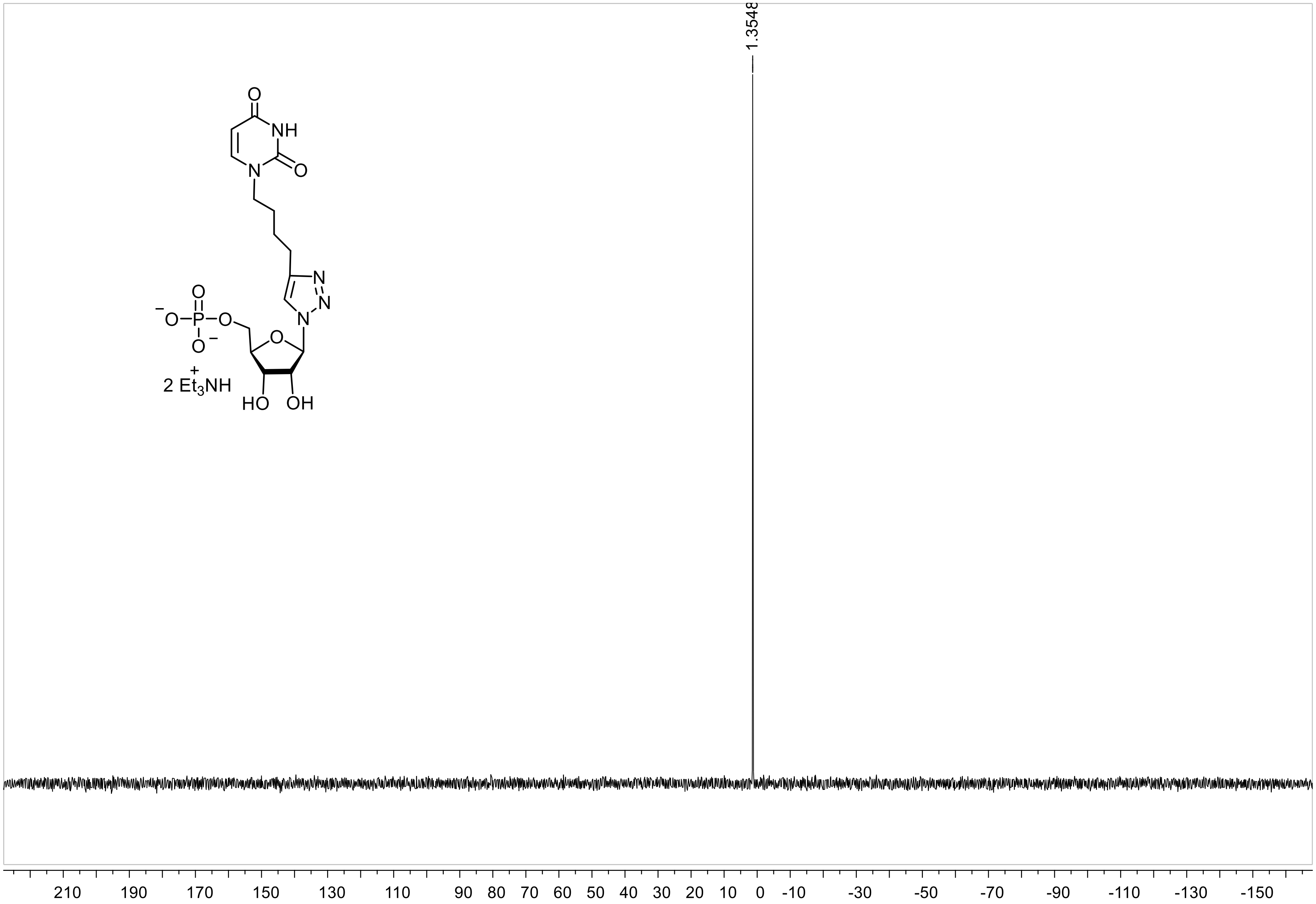

Figure S95. ^{31}P NMR spectrum of **26a** in CD_3OD

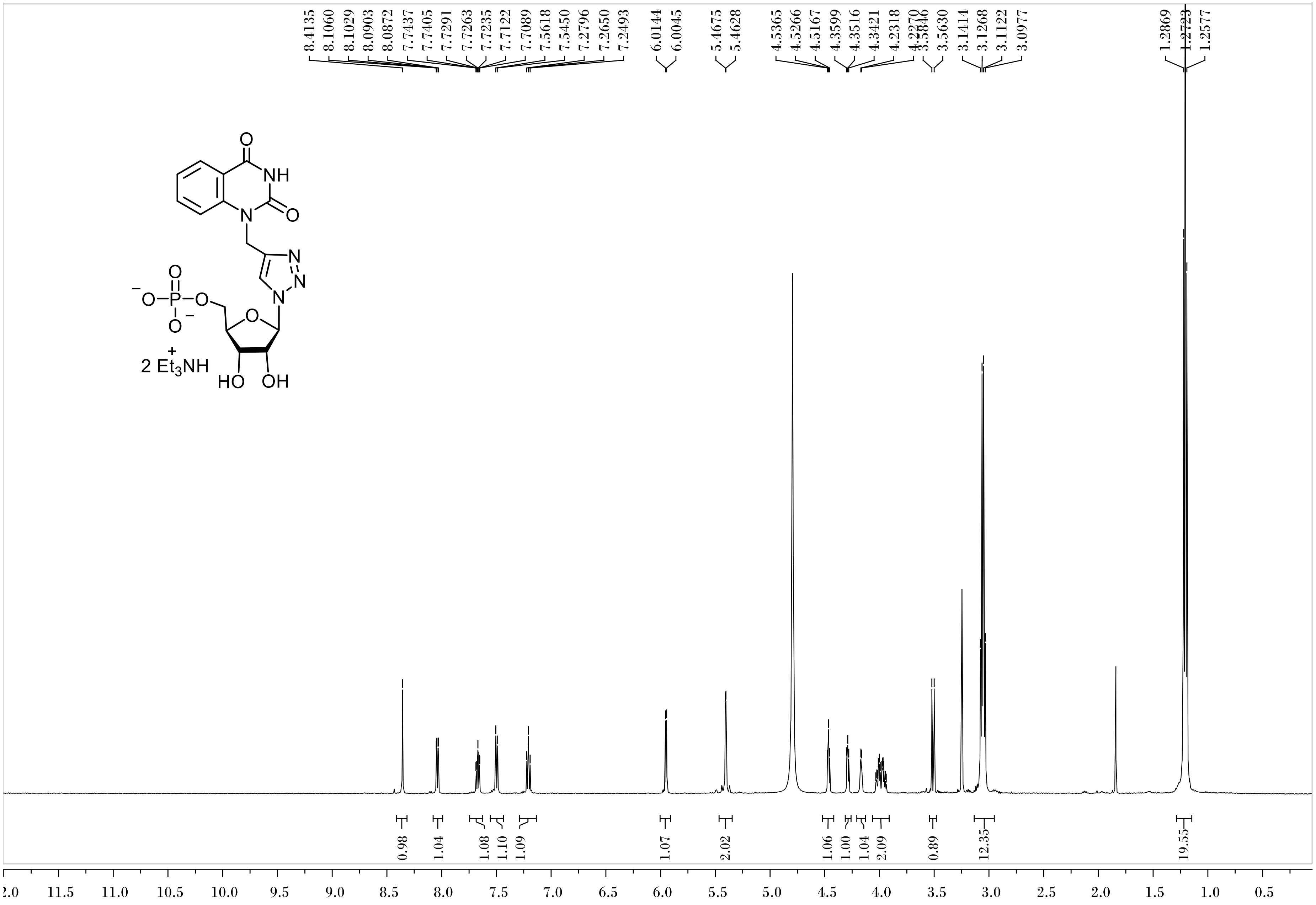

Figure S96. ^{1}H NMR spectrum of **25b** in CD_3OD

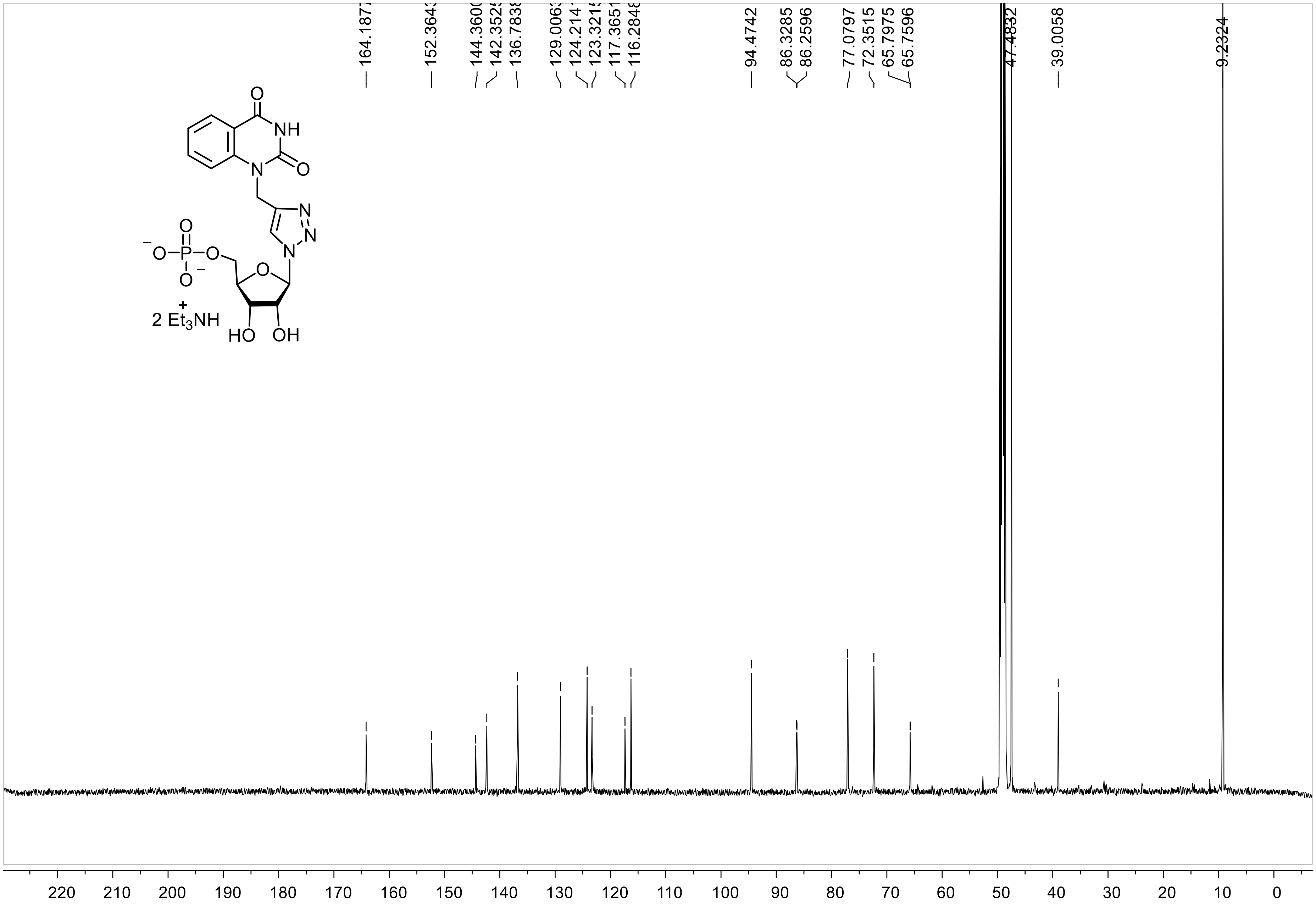

Figure S97. ^{13}C NMR spectrum of **25b** in CD_3OD

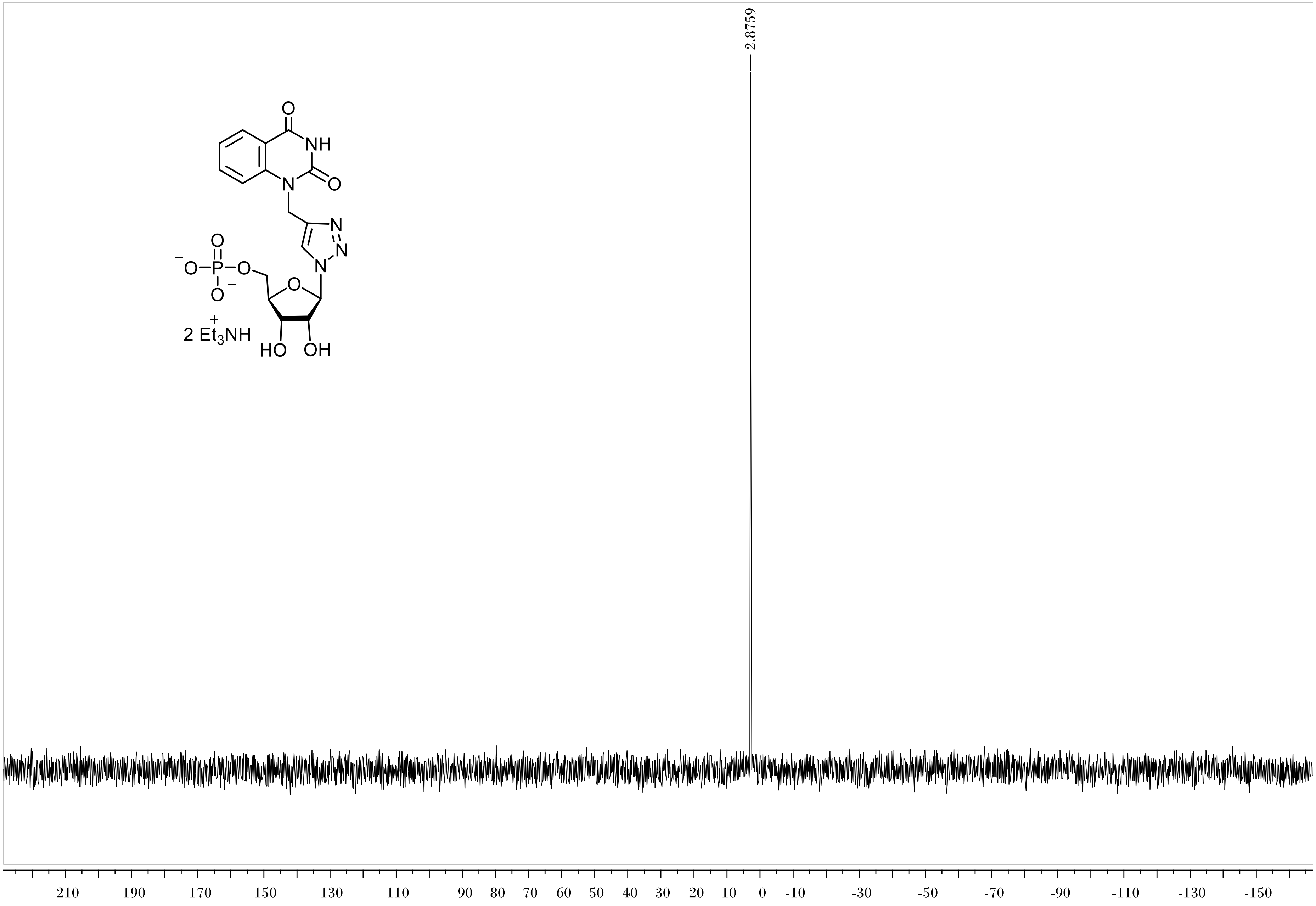

Figure S98. ^{31}P NMR spectrum of **25b** in CD_3OD

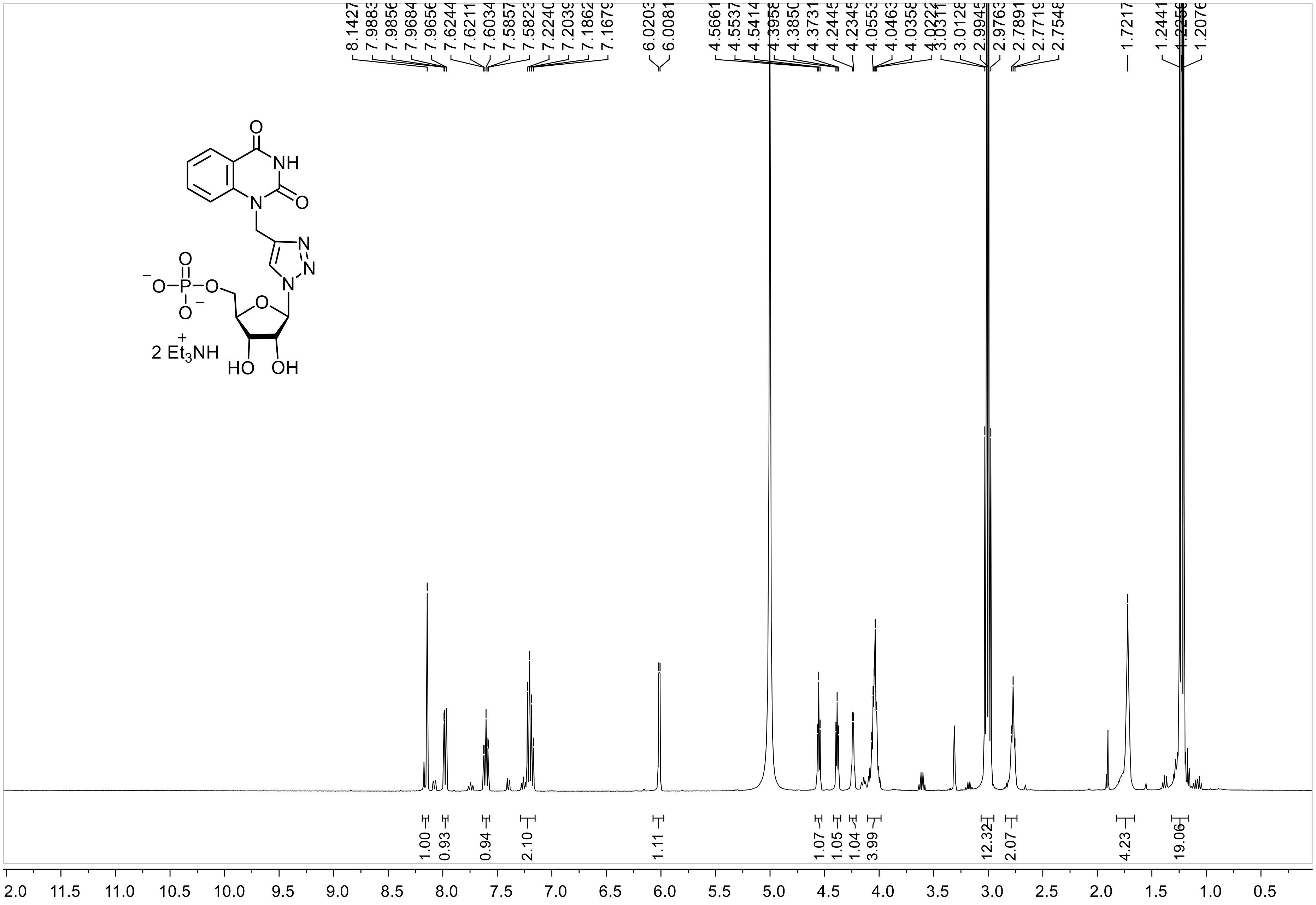

Figure S99. ^{1}H NMR spectrum of **26b** in CD_3OD

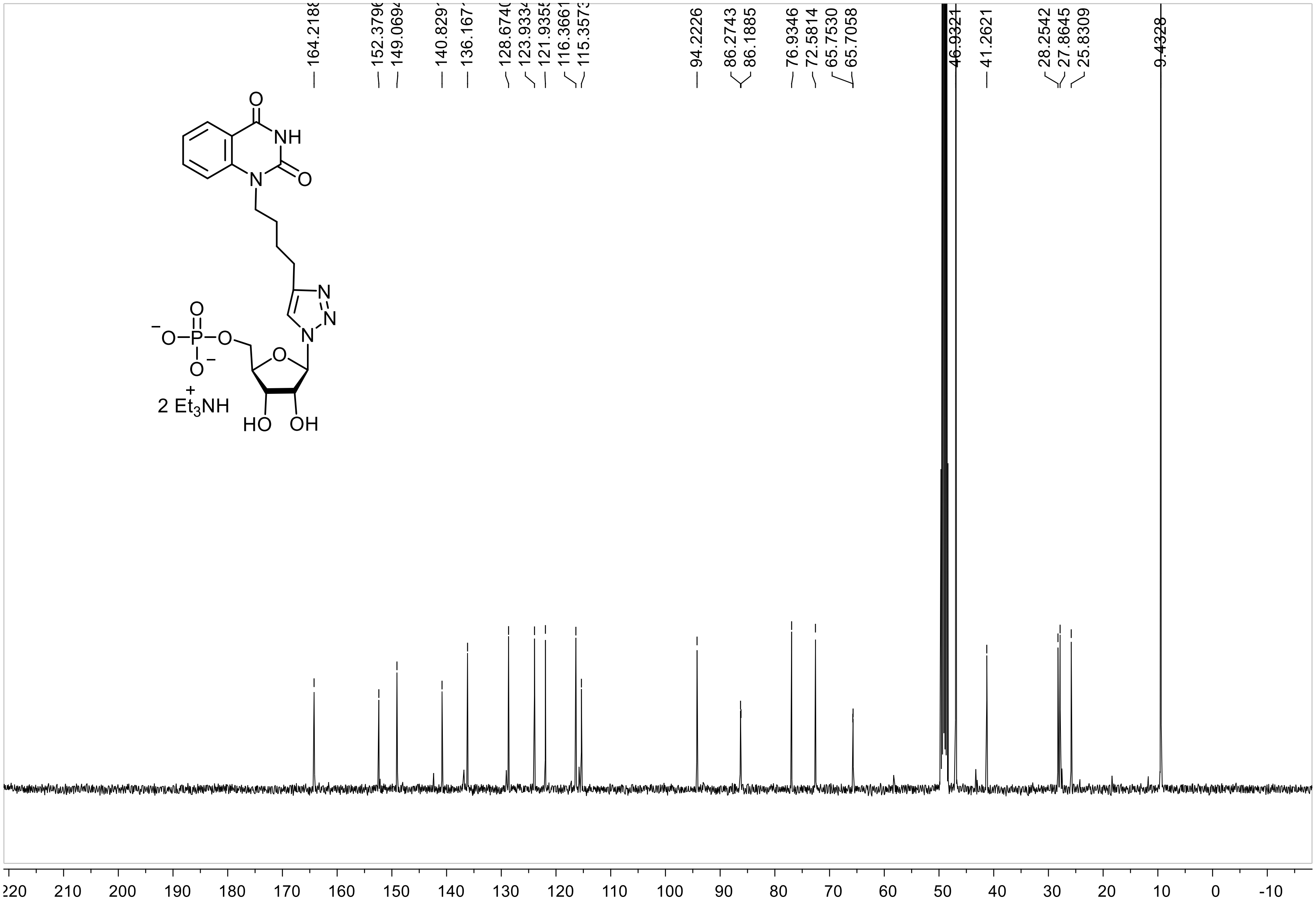

Figure S100. ^{13}C NMR spectrum of **26b** in CD_3OD

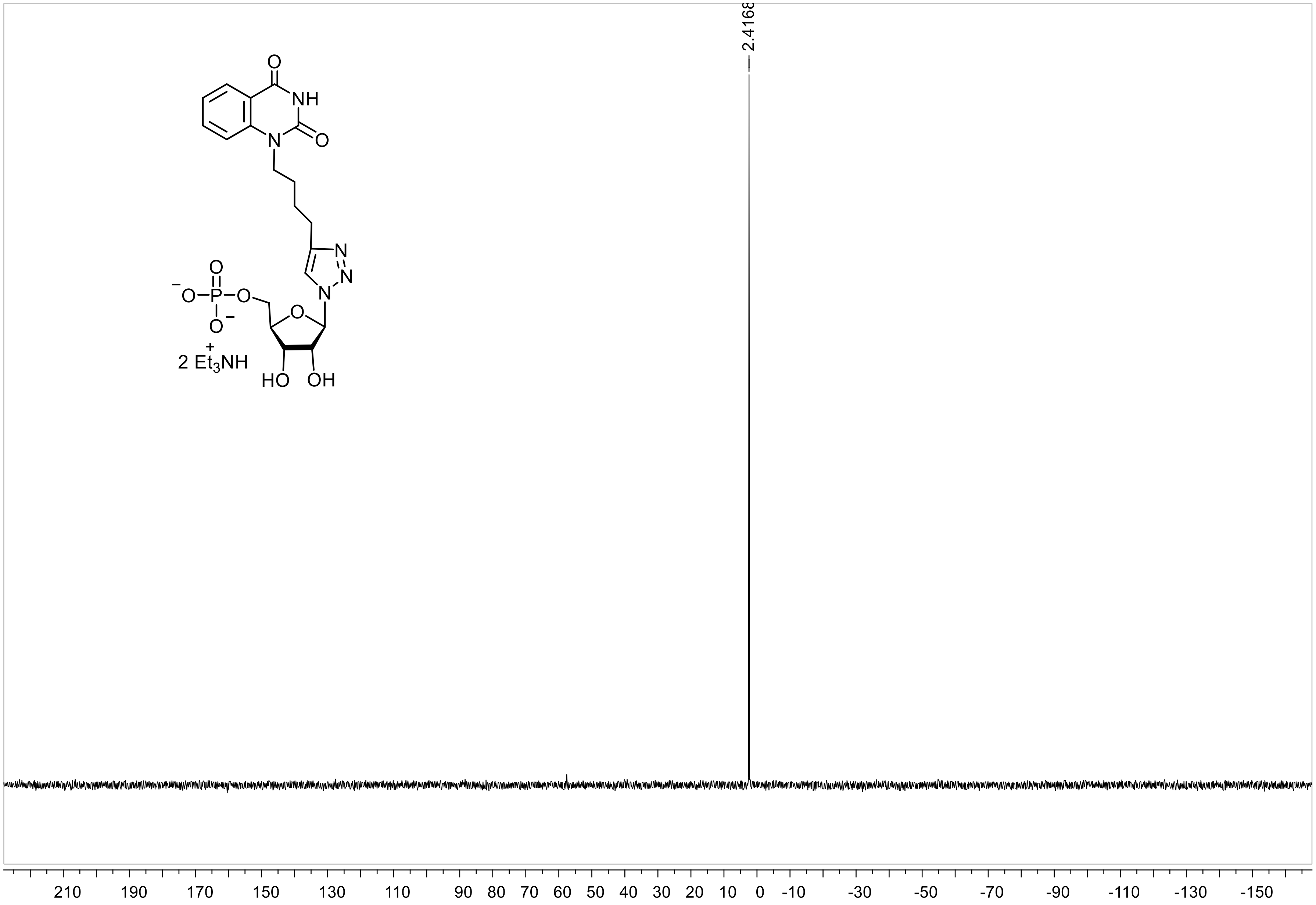

Figure S101. ^{31}P NMR spectrum of **26b** in CD_3OD

8. Antiviral assay

MDCK (ATCC CCL-34) cells initially obtained from American Type Culture Collection (Rockville, MD, USA) were seeded into 96-well plates and incubated for 24 h at 36 °C at 5% CO_2 until confluent monolayer is formed. Three-fold dilutions (400 - 4 μg/mL) were prepared on Eagle's minimal essential medium (MEM) from the compounds under investigation, added to the cells and incubated for 24 h at 36 °C at 5% CO_2. The cell monolayer was washed twice with saline (0.9% NaCl) and 100 μL of MTT solution [3-(4,5-dimethylthiazole-2)-2,5-diphenyltetrazolium bromide], 0.5 μg/mL in MEM, were added into each well. The plates were incubated for 1 h at 36 °C, then the medium was removed and formazan pellets were dissolved in dimethyl sulfoxide (0.1 mL per well). The optical density in the wells was measured on a spectrophotometer Thermo Multiskan FC at the wavelength of 540 nm. The results obtained were used for calculating the concentration of the compound resulting in death of 50% cells in the culture (CC_{50}) using GraphPad Prism software employing the four-parameter logistic curve model. The values of CC_{50} were then converted from μg/mL to μM.

The compounds in appropriate concentrations were added to cells (0.1 mL per well). Cells were further infected with A/Puerto Rico/8/34 (H1N1) influenza virus (for MDCK cells) and incubated for 48 hours at 36°C at 5% CO_2. After that, cell viability was assessed by MTT test (see above). The cytoprotective activity of compounds was considered as their ability to increase the values of OD comparing to control wells (with virus only, no drugs). Based on the results obtained, the values of IC_{50}, *i.e.* concentration of compounds that result in 50% cells protection were calculated using GraphPad Prism software.

9. Molecular docking study

Molecular docking was carried out using the Autodock 4.2 Vina software and AutoDock Tools (ADT 1.5.6) [6s]. The three-dimensional (3D) crystal structure of N-terminal endonuclease domain of polymerase acidic protein (PA) of RNA-dependent RNA polymerase (PDB code 4AWK) [7s] was obtained from the RCSB Protein Data Bank [8s]. The standard 3D structures of **29a,b**, **30a,b** were constructed using the HyperChem 8.0 [9s] and converted into a pdb file by Open Babel [10s]. The cubic grid box of 22 × 20 × 20 Å (x, y, z) with a spacing of 1.000 Å and grid maps were generated. The docking parameters were used as the default settings.

10. References

1s. Andreeva, O.V.; Belenok, M.G.; Saifina, L.F.; Shulaeva, M.M.; Dobrynin, A.B.; Sharipova, R.R.; Voloshina, A.D.; Saifina, A.F., Gubaidullin, A.T.; Khairutdinov, B.I.; Zuev, Y.F.; Semenov, V.E.; Kataev, V.E. Synthesis of novel 1,2,3-triazolyl nucleoside analogues bearing uracil, 6-methyluracil, 3,6-dimethyluracil, thymine, and quinazoline-2,4-dione moieties. *Tetrahedron Lett*. **2019**, *60*, 151276.

2s. Singh, U.S.; Chung K. Chu, C.K. Synthesis of 2′-deoxy-2′-fluoro-2′-C-methyl spiro cyclopentyl carbocyclic uridine analog as potential inhibitors of HCV NS5B polymerase. *Nucl. Nucl. Nucl. Acids* **2020**, *30* (1-3), 52-68.

3s. Koizumi, T.; Arai, Y.; Yoshii, E. The Reaction of *o*-Phenylenediamine with Phenyl Phosphorodichloridate. Synthesis and Reactions of 2-Phenoxy-1,3-Dihydro-2-H-1,3,2-Benzodiazaphosphole-2-Oxide and Related Compounds. *Chem. Pharm. Bull.* **1973**, *21*, 202–206, doi:10.1248/cpb.21.202.

4s. McGuigan, C.; Pathirana, R.N.; Balzarini, J.; De Clercq, E. Intracellular Delivery of Bioactive AZT Nucleotides by Aryl Phosphate Derivatives of AZT. *J. Med. Chem.* **1993**, *36*, 1048–1052,

5s. Young, R.W. A Re-Examination of the Reaction Between Phosphorus Trichloride and Salicylic Acid. *J. Am. Chem. Soc.* **1952**, *74*, 1672–1673.

6s. Trott, O.; Olson, A.J. AutoDock Vina: improving the speed and accuracy of docking with a new scoring function, efficient optimization and multithreading. *J. Comput. Chem*. **2010**, *31*, 455–461.

7s. Kowalinski E., Zubieta Ch., Wolkerstorfer A., Szolar O. H. J., Ruigrok R. W. H., Cusack S. Structural Analysis of Specific Metal Chelating Inhibitor Binding to the Endonuclease Domain of Influenza pH1N1 (2009) Polymerase. PLoS Pathogens **2012**, *8*, e1002831.

8s. Berman, H.M.; Westbrook, J.; Feng, Z.; Gilliland, G.; Bhat, T.N.; Weissig, H.; Shindyalov, I.N.; Bourne, P.E. The protein data bank. *Nucleic Acids Research* **2000**, *28*, 235–242.

9s. HyperChem Professional 8.0 (2007). Hypercube, Inc. http://www.hyper .com/?tabid =360. Accessed 26 July 2022.

10s. O’Boyle, N.M.; Banck, M.; James, C.A.; Morley, C.; Vandermeersch, T.; Hutchison, G.R. Open babel: An open chemical toolbox. *J. Cheminform* **2011**, *3*, 33.
